# Highly‐Selective Electrochemical Decarboxylative Late‐Stage Functionalization of Amino Acids

**DOI:** 10.1002/chem.202501287

**Published:** 2025-04-24

**Authors:** Adrija Ghosh, Vishal Kumar Parida, Tristan von Münchow, Lutz Ackermann, Debasis Banerjee

**Affiliations:** ^1^ Department of Chemistry Indian Institute of Technology Roorkee Roorkee Uttarakhand 247667 India; ^2^ Institute for Organic and Biomolecular Chemistry Georg‐August‐Universität Göttingen Tammannstr. 2 37077 Göttingen Germany

**Keywords:** alcohols, C─X bond constructions, decarboxylation, electrocatalysis, late‐stage functionalization, metal‐free

## Abstract

A unified electrochemical decarboxylative strategy for the site‐selective construction of carbon‐heteroatom bonds is disclosed herein. The metal‐ and catalyst‐free decarboxylation provides access to the functionalization of C‐ and N‐terminus from the simple amino acid feedstock. A wide variety of primary, secondary, and tertiary acids or alcohols were well tolerated. Late‐stage functionalization using *α*‐D‐galactopyranose, di‐peptide, steroid derivatives, and bio‐active drug molecules established the robustness and synthesis potential of our approach.

## Graphical abstract

Electrochemical decarboxylative late‐stage functionalization of the C‐ or N‐terminus of natural amino acids is presented. Metal and catalyst‐free protocol widely explored the synthesis of hindered ethers, amines, or di‐peptide.

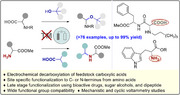

## 1 Introduction

**Scheme 1:**
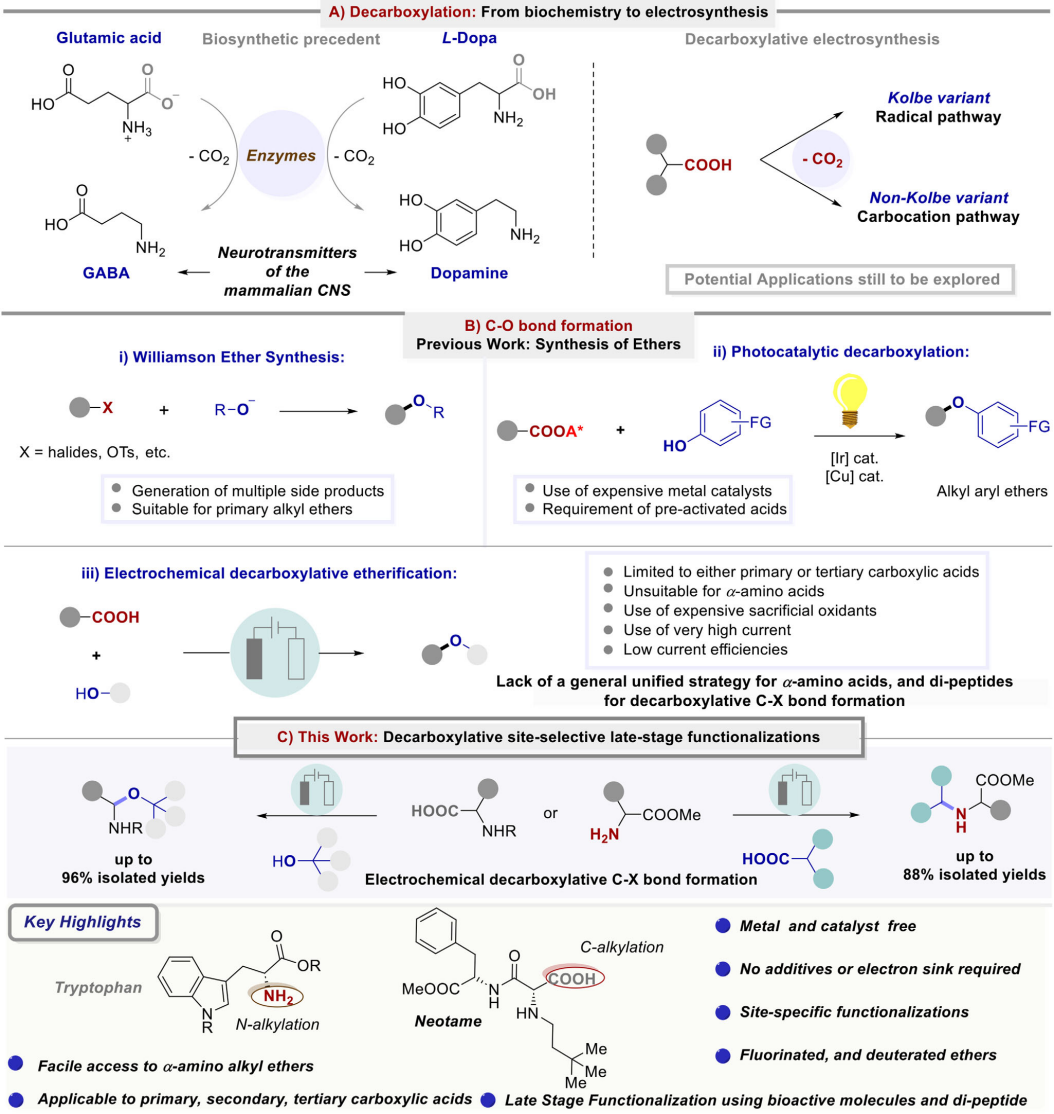
A) Bio‐chemical or electro‐chemical precedence on decarboxylation approaches. B) State‐of‐the‐art on decarboxylative C─O bond construction. C) Present strategy: Electrocatalytic decarboxylation for site‐selective access to C‐ and N‐terminus of amino acids for late‐stage functionalization.

The controlled and site‐specific decarboxylation from free amino acids is a fundamental biochemical transformation. For instance, GABA (*γ*‐aminobutyric acid),^[^ ^1^ ^]^ and Dopamine,^[^ ^2^ ^]^ two key neurotransmitters of the human central nervous system, produced by natural enzymatic decarboxylation pathways from glutamic acid, and *L*‐dopa (Scheme 1A). Considering the high natural abundance of carboxylic acids, significant applications of Kolbe,^[^ ^3^ ^]^ Hunsdiecker,^[^ ^4^ ^]^ and Barton decarboxylation,^[^ ^5^ ^]^ protocols have widely been used by synthesis chemists and enabled novel strategies for the construction of complex organic molecules.

Decarboxylative approaches have witnessed rapid growth during the past decades based on transition metals, and photo‐redox strategies (Scheme 1B). Radical decarboxylation has widely been explored using photo‐redox catalysis and has overcome several limitations of the conventional transition‐metal catalyzed pathways.^[^ ^6^ ^]^ However, the pre‐activation of carboxylic acids as redox‐active esters or expensive photo‐redox catalysts typically translates into considerable challenges.^[^ ^7^ ^]^ Hence, the use of un‐activated carboxylic acid offers an improved atom and resource efficiency and enables productive engagement of electro‐synthetic concepts.^[^ ^8^ ^]^ Employing electrons as the sole redox reagents has proven beneficial in a plethora of organic transformations and eliminated the requirement of chemical oxidants or the need for pre‐activation of carboxylic acids.^[^ ^8b–c^ ^]^

Pioneered by Kolbe and Faraday, the anodic oxidation of carboxylic acids led to the formation of carbon‐centred radicals, which undergo homo‐coupling to alkanes.^[^ ^3^, ^9^ ^]^ However, often such in situ‐generated radicals can undergo further anodic oxidation to a carbocation, which can be trapped with suitable nucleophiles, typically referred to as the Hofer–Moest decarboxylative transformation.^[^ ^8^, ^10^ ^]^ Notably, Hofer–Moest reactions continue to be associated with considerable challenges; for instance, in situ generated carbocations are highly reactive and prone to undergo rearrangement, elimination or often coupled with undesired nucleophiles leading to major issues on product selectivity.^[^ ^11^ ^]^ Therefore, C─O nucleophiles are required in large excess. Recently, Baran,^[^ ^12^ ^]^ elegantly established Hofer–Moest decarboxylative protocol using stoichiometric silver salts as the sacrificial oxidant. Though, an extended scope of various alcohols and carboxylic acids have been demonstrated, however, limited to tertiary, benzylic, and allylic substituted carboxylates (Scheme 1B, iii). Notably, primary and secondary carboxylic acids proved unfortunately not compatible with the optimized protocol due to the highly reactive and transient stability of the resultant carbocations. Very recently, Gooßen reported the Kolbe decarboxylative radical‐radical coupling of lithium alkyl carboxylate using very high current density in combination with platinum electrodes. Here, the application of primary and secondary acids using methanol/pyridine (12 mL) as a solvent combination along with a strong base. However, the application of tertiary carboxylic acids and *α*‐amino acids was unfortunately not met with success (Scheme 1B, iii).^[^ ^13^ ^]^

Reflecting on these studies, we wondered whether challenges related to side reactions of the in situ generated radicals via decarboxylation of the corresponding acids constitute a major pitfall.^[^ ^12^, ^13^, ^14^, ^15^ ^]^ Further, the requirement of activated substrates in addition to over‐oxidation and rearrangement of the generated carbocation are the major challenges. These limitations often confine the general and broad applications of electrochemical decarboxylative etherification, require expensive sacrificial oxidants, high current density, and the use of rather expensive Pt‐based electrodes.

*α*‐Amino acids are ubiquitous as non‐fossil carbon sources and are an integral part of living organisms.^[^ ^15^ ^]^ Despite these, broad applications of *α*‐amino acids have rarely been explored using electrochemical transformations.^[^ ^16^ ^]^ More specifically, site‐selective C‐terminus or N‐terminus functionalization of *α*‐amino acids has remained unexplored for a long time.

Herein, we wish to disclose a general and site‐selective functionalization of natural amino acids following a catalyst and additive‐free electrochemical decarboxylation strategy. A broad variety of natural amino acids and dipeptides were compatible in the presence of primary, secondary, or tertiary un‐activated aliphatic alcohols. Site‐specific functionalization to the C‐ or N‐terminus of free amino acids can be accessed using our decarboxylation approach (Scheme 1C). This versatile direct electro‐decarboxylative protocol has also proved successful in the case of un‐activated primary, secondary, or tertiary‐carboxylic acids, as well as a wide variety of hindered ethers. Further, late‐stage functionalization using a series of pharmaceutically active acids, amino acids, sugar‐derivatives, steroid hormones, vitamin analogues, and alcohols demonstrated the practical significance of the established protocol.

## 2 Results and Discussion

We began our investigations using *N*‐Boc‐DL‐*α*‐phenylglycine (**1a**) as the model substrate with methanol (**2a**) as alcohol source (Table 1).

**Table 1:** Optimization studies.^[^ ^a^ ^]^

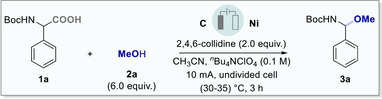		
Entry	Deviation from standard conditions	Yield of 3a [%]^[^ ^b^ ^]^
1	none^[^ ^a^ ^]^	82 (78)^[^ ^c^ ^]^
2	MeOH (3.0 mL) as solvent	99
3	2,6‐lutidine as base	80
4	1,1,3,3‐tetramethyl guanidine as base	25
5	DBU as base	30
6	Tri‐ethylamine as base	<5
7	*^n^* Bu_4_NI (0.1 M) as electrolyte	n.d.^[^ ^d^ ^]^
8	*^n^* Bu_4_NPF_6_ (0.1 M) as electrolyte	60
9	(+)C/(‐)C	51
10	(+)C/(‐)Ni‐foam	62
11	(+)C/(‐)C, AgClO_4_ as additive	53^[^ ^e^ ^]^
12	(+)C/(‐)C, AgPF_6_ as additive	47^[^ ^e^ ^]^
13	No current	n.d.^[^ ^d^ ^]^
14	Current < 10 mA	<40

^[a]^ standard conditions: **1a** (0.30 mmol), **2a** (1.8 mmol), 2,4,6‐collidine (0.60 mmol), graphite as anode, nickel as cathode, in 3 mL acetonitrile, constant current (*I*) = 10 mA, in an undivided 5 mL Electrasyn 2.0 vial at 30–35 °C for 3 hours.

^[b]^ yield based on GC and GC‐MS.

^[c]^ isolated yield.

^[d]^ not detected.

^[e]^ Ag‐salt (0.9 equiv.), CH_2_Cl_2_ (3.0 mL) as solvent.

In order to limit the use of excess alcohol as a solvent, we examined our model reaction with 6.0 equivalents of methanol in combination with 3.0 mL of acetonitrile as the solvent combination under anodic oxidation conditions and observed high product yield to **3a** (GC yield of 82% and an isolated yield of 78%) (Table 1, entry 1). Although excess methanol resulted in a quantitative yield to the desired product (Table 1, entry 2), a larger excess of alcohol might not be practical for complex alcohols. Systematic screening of bases revealed that the non‐nucleophilic and non‐oxidizable nature of the base played a crucial role in product selectivity. Weak organic bases, such as, 2,4,6‐collidine (Table 1, entry 1) and 2,6‐lutidine (Table 1, entry 3) resulted in excellent yield to the desired product **3a**. In contrast, strong organic bases, such as 1,1,3,3‐*tetra*‐methylguanidine and DBU, resulted in poor product to **3a** (Table 1, entries 4 and 5, SI Table S1). When *tri*‐ethylamine was used as a base, only a trace amount of the desired product was detected (Table 1, entry 6). Next, the influence of various electrolytes revealed that large and non‐oxidizable anions, such as, PF_6_^−^ or ClO_4_^−^ assist the reaction, whereas electrolytes comprising halide anions were incompatible with the model decarboxylation process (Table 1, entries 7 and 8, SI Table S2). A series of different electrode materials were tested for the model reaction, and metallic nickel as the cathode proved better than nickel foam or graphite for our decarboxylative transformations (Table 1, entries 9 and 10, SI Table S4). Thereafter, the addition of silver additives as the sacrificial oxidant on the standard reaction conditions did not prove beneficial while employing graphite as both the anode and cathode (Table 1, entry 11, SI Table S3).^[^ ^12^ ^]^ Control experiments for the decarboxylative etherification in the absence of base and current disclosed their key role (Table 1, entries 13 and 14, and Tables S1 and S6). To our delight, we have successfully obtained the desired *N*‐protected *α*‐amino ether **3a** under metal and additive‐free electrochemical decarboxylation (Table 1 and SI Table S1–S6).

**Scheme 2:**
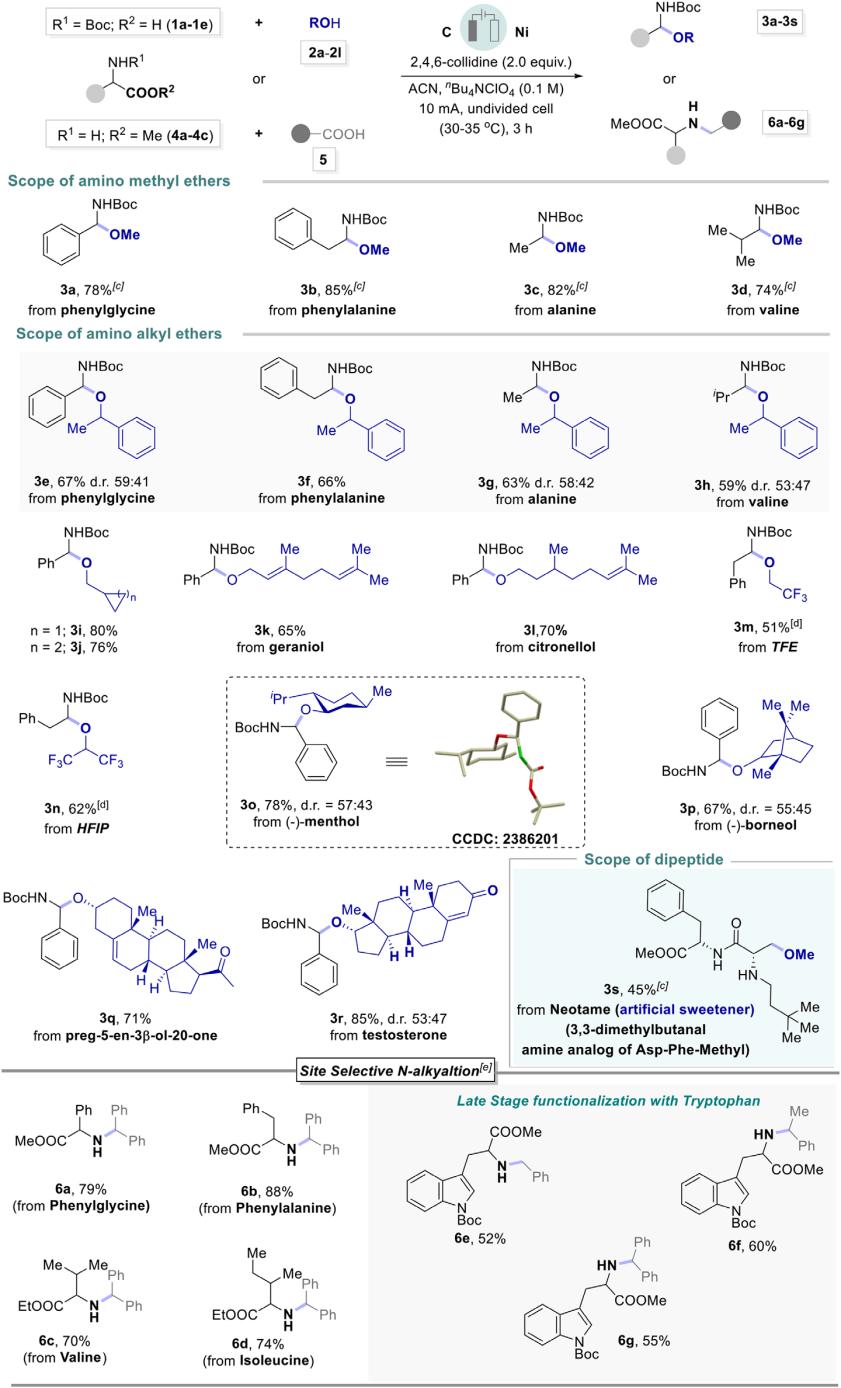
Site‐selective electrochemical decarboxylative C─C and C─N bond functionalization. a) 1 (0.3 mmol), 2 (0.9 mmol), 2,4,6‐collidine (0.6 mmol), graphite as anode, nickel as cathode, in 3 mL CH_3_CN, constant current 10 mA, in an undivided 5 mL Electrasyn 2.0 vial at room temperature for 3 h. b) isolated yields, c) 2a (1.8 mmol), d) 2 (1.5 mmol). e) CH_3_CN:CH_2_Cl_2_ (3 mL, 1:1) as solvent.

With the optimized reaction conditions in hand, we explored the scope of our approach using a series of *α*‐amino acids with alcohols as coupling partners (Scheme 2). To our delight, electrochemical decarboxylation *of α*‐amino acids, such as *DL*‐phenylalanine (**1b**), *DL*‐alanine (**1c**), and *DL*‐valine (**1d**), resulted in good to excellent yield to *α*‐amino methyl ethers (**3b**‐**3d**) respectively. Further, with a minimum alternation in the alcohol stoichiometry, a wide variety of simple and complex alcohols participated in the decarboxylation with *α*‐amino acids and exhibited good to excellent yield of *α*‐amino alkyl ethers. For instance, decarboxylation of *α*‐amino acids (**1a**‐**1d**) with 1‐phenylethyl alcohol (**2b**) resulted in *α*‐amino ethers **3e**‐**3** **h** in up to 67% yield respectively. Notably, only 3.0 equivalents of the alcohol are required as a coupling partner for such transformation (Scheme 2).^[^ ^13^ ^]^

Thereafter, the application of primary alcohols, such as cyclopropylmethanol (**2c**) and cyclobutylmethanol (**2d**) afforded the desired *α*‐amino ethers **3i** and **3j** in high yield without any notable distortion in the cycloalkane motif. Naturally occurring acyclic mono‐terpenoids, geraniol **2e**, and citronellol **2f**, were also compatible with this optimized protocol and resulted in **3k‐3l** in up to 70% yield. Interestingly, more challenging fluorinated primary and secondary aliphatic alcohols (**2g** and **2h** ) also exhibited similar reactivity for the electrochemical decarboxylation with an acceptable yield of **3** **m** and **3n** respectively. Pharmacophores having a cyclic mono‐terpenoid core, such as menthol (**2i**), and borneol (**2j**), were successfully coupled with *N*‐Boc‐DL‐*α*‐phenyl glycine **1a** and transformed into **3o** and **3p** in good to high yield. To our delight, late‐stage functionalization using steroid alcohols, 5‐pregnen‐3*β*‐ol‐20‐one (**2k**) and testosterone (**2l**), smoothly participated and resulted in *α*‐amino ethers **3q** and **3r**, in high yield without much affecting the existing functional groups on steroid core (Scheme 2).

Thereafter, we further explored the decarboxylative strategy using a di‐peptide; 3,3‐di‐methylbutanal amine analogue of Asp‐Phe‐Methyl ester (neotame). Neotame is widely used as an artificial sweetener and features an amide bond in addition to amine and carboxylic acid functionality in the same molecule. Notably, using our protocol, electrochemical decarboxylative coupling of di‐peptide with methanol (**2a**) resulted in the desired products **3s** in moderate yield (Scheme 2).

Next, we focused on site‐selective decarboxylative functionalization to the *N*‐terminus of free amino acids. The electrochemical C─N bond formation was facilitated by the decarboxylative coupling of simple acids (**5a**–**5c**) with the free amine groups of *α*‐amino acids. For instance, phenylacetic acid, 2‐phenylpropionic acid, and di‐phenyl acetic acid, smoothly coupled with free amines in *DL*‐*α*‐phenylglycine methyl ester (**4a**), *DL*‐phenylalanine methyl ester (**4b**), *DL*‐valine ethyl ester (**4c**), and *DL*‐isoleucine ethyl ester (**4d**) respectively and resulted in good to excellent yields to **6a**–**6d** (Scheme 2). Additionally, late‐stage functionalization of tryptophan methyl ester (**4e**) resulted in the desired N‐coupled products with free acids in up to 60% yield (**6e**‐**6g**).

**Scheme 3:**
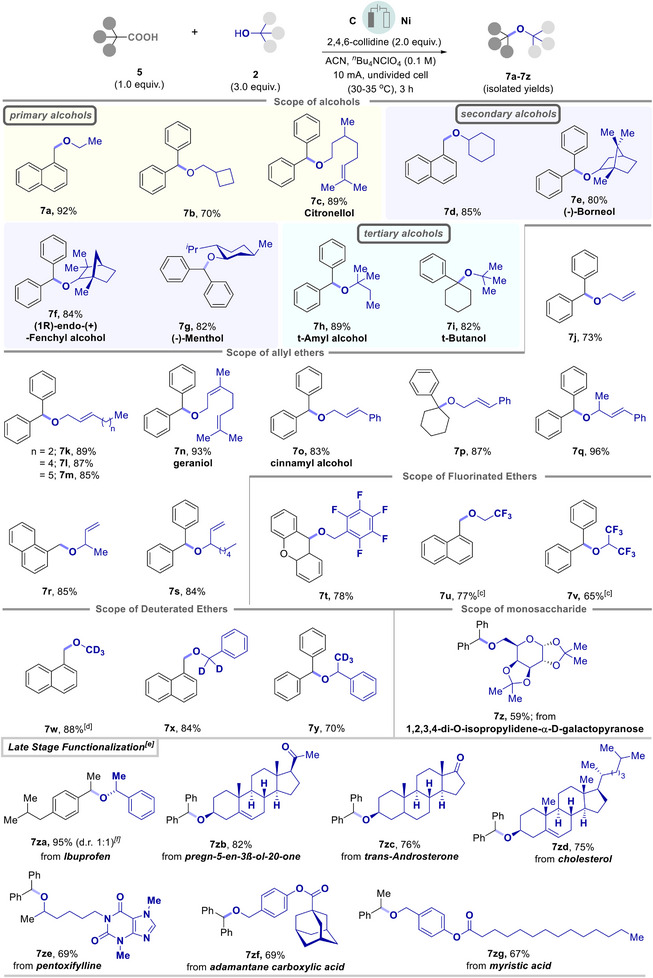
The substrate scope of simple and hindered alkyl ethers. a) 5 (0.2 mmol), 2 (0.6 mmol), 2,4,6‐collidine (0.4 mmol), graphite as anode, nickel as cathode, in 3 mL CH_3_CN, constant current 10 mA, in an undivided 5 mL Electrasyn 2.0 vial at room temperature for 3 h. b) isolated yield. c) 2 (1.0 mmol), d) CD_3_OD (0.1 mL). e) acid (0.2 mmol), alcohol (0.2 mmol), f) alcohol (0.6 mmol).

After witnessing the excellent scope of natural amino‐acids on electrochemical decarboxylation, we have established the synthesis of hindered ethers due to their prevalence in agrochemicals, pharmaceuticals, and bioactive natural products,^[^ ^17^, ^18^ ^]^ A brief literature survey revealed the lack of suitable reports on the synthesis of hindered ethers, which usually associated with harsh reaction conditions, restricted scope on functional substrates and more specifically selectivity issues.^[^ ^19^ ^]^ In this regard, direct synthesis of hindered ether using a sustainable strategy is of utmost importance and attracted potential importance. Herein, we have demonstrated a unified strategy for the synthesis of ethers from a range of primary, secondary, and tertiary carboxylic acids with suitable alkyl alcohols using an electrochemical decarboxylation strategy under metal‐ and additive‐free approach as presented in Scheme 3.

Decarboxylative etherification of primary acid, 1‐naphthylacetic acid **5d**, with primary and secondary alcohols resulted in the desired products, **7a** and **7d** in 85–92% yield respectively. Next, the application of di‐phenyl acetic acid **5a** with primary alcohols, as well as linear or mono‐cyclic terpenoids, such as citronellol, borneol, fenchol, and menthol were successfully transformed to the hindered ethers **7b**–**7c,** and **7e**–**7g** in good to excellent yield. Thereafter, more challenging and sterically hindered ethers substituted with tertiary carbons on both sides were successfully synthesized via decarboxylation of secondary and tertiary acids with *t*‐amyl alcohol and *t*‐butanol, respectively and resulted in **7h** and **7i** in up to 89% yield. To our delight, a series of linear or branched allylic alcohols smoothly participated and resulted in a variety of allylic ether **7j**–**7s** in good to excellent yields. For instance, allyl alcohol, geraniol, or cinnamyl alcohol, including branched alcohols are well tolerated with a variety of secondary and tertiary acids (Scheme 3). Electrochemical decarboxylative coupling of fluoroalkylated alcohols with primary and secondary acids, resulted in fluorinated ethers, **7t**–**7v**, in good to high yield respectively. Pleasingly, deuterated methanol, D_2_‐benzyl alcohol, and D_3_‐1‐phenylethanol also exhibited comparable reactivity leading to the formation of deuterated ethers **7w‐7y** respectively. Notably, α‐D‐galactopyranose smoothly coupled with di‐phenyl acetic acid and **7z** was obtained in 59% yield.

Next, we extended our electro‐decaroxylation towards the late‐stage functionalization and diversification of specialized chemicals as presented in Scheme 3. For instance, decarboxylative late‐stage functionalization of ibuprofen resulted in **7za**, in quantitative yield. Thereafter, the application of steroid derivatives, such as 5‐pregnen‐3*β*‐ol‐20‐one, *trans*‐androsterone, and cholesterol was efficiently coupled with di‐phenyl acetic acid to the desired ethers **7zb**‐**7zd** in 75–82% yield respectively. Pentoxifylline, a xanthine derivative widely used as a vasodilator, coupled with diphenyl acetic acid and **7ze** was obtained in 69% yield. Moreover, alcohols derived from adamantane carboxylic acid and myristic acid were well tolerated under electrochemical transformation and result in **7zf** and **7zg** in moderate yields. This late‐stage electrochemical functionalization revealed new opportunities for synthetically challenging ethers.^[^ ^19^, ^20^ ^]^

**Scheme 4:**
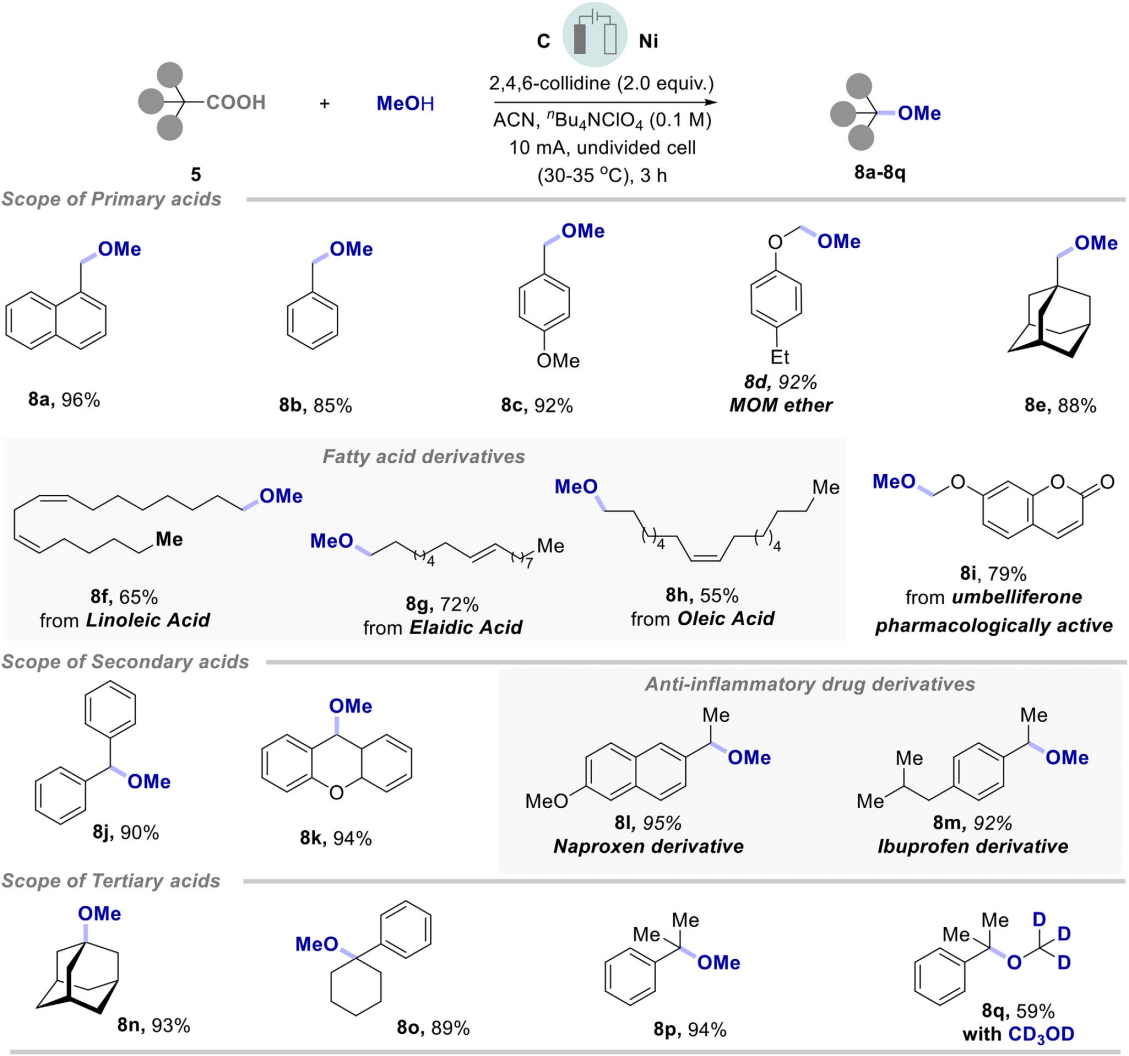
Scope of methyl ethers. a) 5 (0.3 mmol), MeOH (1.8 mmol), 2,4,6‐collidine (0.6 mmol), graphite as anode, nickel as cathode, in 3 mL CH_3_CN, constant current 10 mA, in an undivided 5 mL Electrasyn 2.0 vial at room temperature for 3 h. b) isolated yield. c) CD3OD (0.1 mL).

Furthermore, our decarboxylative electrochemical strategy proved applicable to more challenging primary alkyl acids, which are often needed in special electrochemical conditions.^[^ ^12^, ^13^ ^]^ Notably, the synthesis of primary alkyl methyl ethers finds wide applications as solvents, fuel additives, refrigerants, and spray propellants. Despite these, only a few reports on electrochemical methyl ether synthesis are known and the requirement of harsh conditions (very high current requirements), in combination with expensive electrode systems limits their broad applications.^[^ ^19^, ^20^ ^]^ Therefore, to overcome such challenges, we investigated a general and efficient approach to the synthesis of methyl ethers using a variety of primary, secondary, and tertiary carboxylic acids (Scheme 4).

To our delight, a series of primary carboxylic acids were smoothly methoxylated to **8a**–**8d**. For instance, 1‐adamantane acetic acid yielded 88% alkyl methyl ether **8e**. Further, long‐chain fatty acids derived from linoleic, elaidic, and oleic acids resulted in acceptable yields of **8f**–**8h**. Umbelliferone, a natural coumarin derivative, known for its anti‐inflammatory, anti‐oxidant, and anti‐diabetic properties, was converted to corresponding methyl ether **8i** in 79% yield. As envisioned, secondary acids were also efficiently transformed into methyl ethers **8j**–**8k**. Moreover, electrochemical decarboxylation of anti‐inflammatory NSAIDs, Naproxen and Ibuprofen led to excellent yield to methyl ethers **8l** and **8m** , respectively. Tertiary acids were also equally effective towards the electrochemical etherification and resulted in methyl ethers **8n**–**8p**. Decarboxylative etherification of 2‐methyl‐2‐phenylpropionic acid with deuterated methanol furnished the deuterated ether **8q** in acceptable yield.

**Scheme 5:**
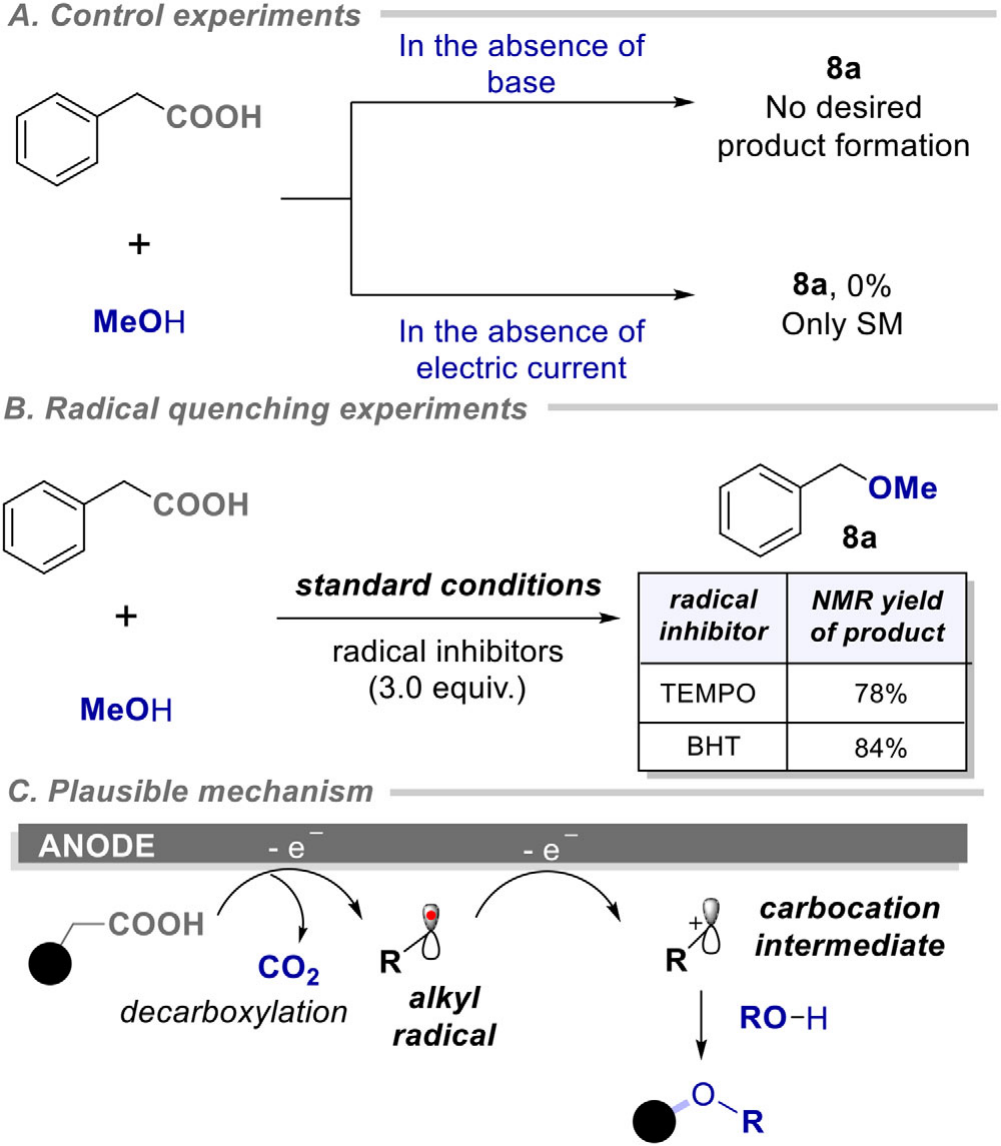
Control experiments and plausible reaction mechanism. Reactions were performed following the standard conditions of Scheme 4.

**Figure 1:**
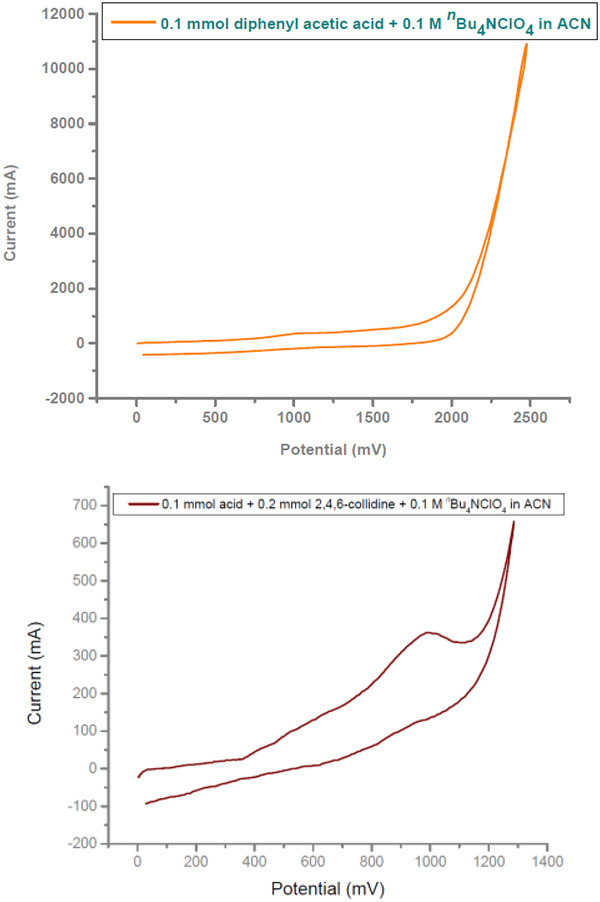
Cyclic Voltammetric Studies. Conditions: GC working /Pt counter electrode, Ag/AgCl reference electrode. A) 0.10 mmol Diphenyl acetic acid in 0.1 M *^n^* Bu_4_NClO_4_, 3.0 mL CH_3_CN solvent, Scan rate = 200 mV s^−1^. B) 0.10 mmol Diphenyl acetic acid and 0.2 mmol 2,4,6‐collidine in 0.1 M *^n^* Bu_4_NClO_4_, CH_3_CN solvent, Scan rate = 200 mV s^−1^.

Then, we studied viable mechanistic scenarios of the reaction (Scheme 5, See SI for further details). Cyclic voltammetry studies were conducted to understand the nature of oxidation of the acid. It was observed that the addition of 2,4,6‐collidine led to a slight change in the cyclic voltammogram and indicated a broad oxidation peak at around 1.05 V (Figure 1). Notably, electrochemical decarboxylation in the absence of base and electric current, did not result in any desired product (Scheme 5A). These studies evident the potential and independent role of base and current for successful product formation. Furthermore, radical quenching experiments with TEMPO and BHT as radical inhibitors ruled out the possibility of the involvement of any radical intermediate for the electrochemical transformations, and a high yield of the desired products was obtained in both cases (Scheme 5B).

Moreover, in situ HRMS studies were conducted to cross‐verify the results obtained from radical quenching experiments. However, no TEMPO or BHT adducts were detected in the HRMS analyses.

Further, we monitored the progress of the reaction via an in situ NMR study. The progress of methyl ether formation from diphenyl acetic acid was analyzed using ^1^H‐NMR spectroscopy. Careful analysis of the crude ^1^H‐NMR after regular intervals of time showed a gradual decrease in the concentration of acid and a comparable increase in the formation of the resulting methyl ether. However, the detection of any possible intermediate species was not successful using NMR studies (Figures S1 and S2). Considering a series of control experiments and literature precedence, a plausible catalytic pathway is presented in Scheme 5C. We envisioned that the electrochemical decarboxylative functionalization of natural amino acids follows the Hofer–Moest pathway, wherein amino acid undergoes sequential two‐electron oxidation and facilitates the decarboxylation process followed by the generation of the carbocation intermediate species.^[^ ^15^, ^21^ ^]^ In the next step, in situ‐generated cationic intermediate species are captured by suitable alcohol or amine nucleophiles leading to the construction of C─C or C─N bonds, respectively.

## 3 Conclusion

We have reported a unified strategy for the site‐selective decarboxylative functionalization to the C‐ and N‐terminus of amino acids and di‐peptide by electro‐oxidation. A variety of primary, secondary, and tertiary acids as well as alcohols were presented, which excludes the need for expensive catalysts, additives, ligands, and metals‐salts. Our strategy proved useful for the facile access to new C─O and C─N bonds, which shall be highly advantageous in the synthesis of complex ethers or site‐specific late‐stage functionalization from natural amino acids. The protocol is tolerant of a wide variety of functional groups, bio‐relevant compounds and peptides.

## Conflict of Interests

The authors declare no conflicts of interest.

## Supporting information



Supporting Information

## Data Availability

The data that support the findings of this study are available in the supplementary material of this article.
