# Supplementary material for: Highly‐Selective Electrochemical Decarboxylative Late‐Stage Functionalization of Amino Acids
Source: Chemistry. 2025 Apr 24;31(28):e202501287. doi: 10.1002/chem.202501287 (PMC12089904; doi:10.1002/chem.202501287)
Supplement: Supplementary file 1 — Supporting Information [file CHEM-31-e202501287-s001.pdf]

Supporting Information

Highly-selective Electrochemical Decarboxylative Late-Stage Functionalization of Amino Acids

Adrija Ghosh,[1] Vishal Kumar Parida,[1] Tristan von Münchow,[2] Lutz Ackermann*,[2] and Debasis Banerjee*[1]

[1] Department of Chemistry, Laboratory of Catalysis and Organic Synthesis
Indian Institute of Technology Roorkee, Roorkee-247667, Uttarakhand, India
E-mail: debasis.banerjee@cy.iitr.ac.in, Website: https://iitr.ac.in/dbiitrlab/

[2] Institute for Organic and Biomolecular Chemistry, Georg-August-Universität Göttingen, Tammannstr. 2, 37077 Göttingen, Germany
E-mail: Lutz.Ackermann@chemie.uni-goettingen.de;
Website: https://www.ackermann.chemie.uni-goettingen.de/

**1. General information**	**S2**
**2. General experimental procedures**	**S2**
**3. Optimization of reaction parameters for electrochemical decarboxylative etherification**	**S4**
**4. Radical Quenching Experiments**	**S6**
**5. Kinetic profile**	**S7**
**6. Cyclic Voltammetry Experiments**	**S8**
**7. Crystallographic data for compound 3o**	**S11**
**8. Analytical data**	**S13**
**9. References**	**S39**
**10. ^{1}H ,^{13}C, ^{19}F NMR spectra of compounds**	**S40**

1. General information

All solvents and reagents were used, as received from the suppliers. TLC was performed on Merck Kiesel gel 60, GF254 plates with the layer thickness of 0.25 mm. Column chromatography was performed on silica gel (100-200 mesh, 230-400 mesh) using a gradient of ethyl acetate and hexane as mobile phase. All NMR spectra (^{1}H, ^{13}C & ^{19}F) were recorded on 500 MHz (JEOL & Bruker) Avance spectrometers. ^{1}H NMR spectral data were collected using 500 MHz and ^{13}C NMR data were recorded using 125 MHz (JEOL & Bruker) and ^{19}F data were recorded using 470 MHz (JEOL). Spectral data was acquired at 298 K. The chemical shifts (δ) are given in parts per million (ppm) and referenced to residual solvent peaks for ^{1}H and ^{13}C NMR spectra: $CDCl_3$, δ_H 7.26 ppm, δ_C 77.16 ppm. Coupling constants (*J*) are reported in Hertz (Hz) to the nearest 0.1 Hz. The following multiplicity abbreviations are used: s singlet, d doublet, t triplet, m multiplet. GC were recorded using Agilent GC 7890B Spectrometer. All the reactions were performed in a closed system using 5 mL vials of Electrasyn 2.0. All electrolytes were purchased from TCI. Tetrabutylammonium chloride (Assay: >98%, CAS Number 1112-67-0; EC Number 214-195-7; Pack Size – I0366-25G). 2,4,6-collidine was purchased from Alfa Aesar (Assay: 99%, CAS Number 108-75-8; EC Number 203-613-3; Pack Size – 16195-100 mL).

2. General Experimental Procedures

General Procedure A for the Synthesis of Substrates 3a-3d:

With no precautions to exclude air or moisture, ElectraSyn 2.0 vial (5 mL) with a stir bar was charged with *N*-Boc-α-amino acid **1** (0.3 mmol, 1 equiv.), MeOH (1.8 mmol, 6 equiv.), 2,4,6-collidine (0.6 mmol, 2 equiv.), $^{n}Bu_4NClO_4$ (0.3 mmol, 1 equiv.), and acetonitrile (3.0 mL). ElectraSyn 2.0 vial cap equipped with anode (graphite) and cathode (nickel) were inserted into the mixture. The reaction mixture was electrolyzed at a constant current of 10 mA for 3 hours. Thereafter, electraSyn 2.0 vial cap was removed, and electrodes were rinsed with ethyl acetate (2 mL), which was combined with the crude mixture. Then, the crude mixture was further diluted with ethyl acetate and the resulting mixture was washed with 2N HCl (20 mL) and $NaHCO_3$ (aq) (20 mL), dried over Na_2SO_4, and concentrated in vacuo. The residue was purified by column chromatography using a gradient of hexane and ethyl acetate (eluent system) to afford the pure product.

General Procedure B for the Synthesis of Substrates 3e-3s:

With no precautions to exclude air or moisture, ElectraSyn 2.0 vial (5 mL) with a stir bar was charged with *N*-Boc-α-amino acid **1** (0.3 mmol, 1 equiv.), alcohol **2** (0.9 mmol, 3 equiv.), 2,4,6-collidine (0.6 mmol, 2 equiv.), $^{n}Bu_4NClO_4$ (0.3 mmol, 1 equiv.), and acetonitrile (3.0 mL). Thereafter, electraSyn 2.0 vial cap equipped with anode (graphite) and cathode (nickel) were inserted into the mixture. The

reaction mixture was electrolyzed at a constant current of 10 mA for 3 hours. Next, electraSyn 2.0 vial cap was removed, and electrodes were rinsed with ethyl acetate (2 mL), which was combined with the crude mixture. Then, the crude mixture was further diluted with ethyl acetate (20 mL) and the resulting mixture was washed with 2N HCl (10 mL) and $NaHCO_3$ (aq) (10 mL), dried over Na_2SO_4, and concentrated in vacuo. The residue was purified by column chromatography using a gradient of hexane and ethyl acetate (eluent system) to afford the pure product.

General Procedure C for the Synthesis of Substrates 6a-6g:

With no precautions to exclude air or moisture, ElectraSyn 2.0 vial (5 mL) with a stir bar was charged with amine **4** (0.3 mmol, 1 equiv.), carboxylic acid **5** (0.6 mmol, 2 equiv.), 2,4,6-collidine (0.6 mmol, 2 equiv.), $^{n}Bu_4NClO_4$ (0.3 mmol, 1 equiv.), and acetonitrile : DCM (3.0 mL, 1:1 ratio). ElectraSyn 2.0 vial cap equipped with anode (graphite) and cathode (nickel) were inserted into the mixture. The reaction mixture was electrolyzed at a constant current of 10 mA for 3 hours. Next, vial cap was removed, and electrodes were rinsed with ethyl acetate (2 mL), which was combined with the crude mixture. Then, the crude mixture was further diluted with ethyl acetate (20 mL) and the resulting mixture was washed with $NaHCO_3$ (aq) (10 mL), dried over Na_2SO_4, and concentrated in vacuo. The residue was purified by column chromatography using a gradient of hexane and ethyl acetate (eluent system) to afford the pure product.

General Procedure D for the Synthesis of Substrates 7a-7z:

With no precautions to exclude air or moisture, ElectraSyn 2.0 vial (5 mL) with a stir bar was charged with carboxylic acid **5** (0.3 mmol, 1 equiv.), alcohol **2** (0.9 mmol, 3 equiv.), 2,4,6-collidine (0.6 mmol, 2 equiv.), $^{n}Bu_4NClO_4$ (0.3 mmol, 1 equiv.), and acetonitrile (3.0 mL). ElectraSyn 2.0 vial cap equipped with anode (graphite) and cathode (nickel) were inserted into the mixture. The reaction mixture was electrolyzed at a constant current of 10 mA for 3 hours. Next, vial cap was removed, and electrodes were rinsed with ethyl acetate (2 mL), which was combined with the crude mixture. Then, the crude mixture was further diluted with ethyl acetate (20 mL) and the resulting mixture was washed with 2N HCl (10 mL) and $NaHCO_3$ (aq) (10 mL), dried over Na_2SO_4, and concentrated in vacuo. The residue was purified by column chromatography using a gradient of hexane and ethyl acetate (eluent system) to afford the pure product.

General Procedure E for the Synthesis of Substrates 7za-7zg:

With no precautions to exclude air or moisture, ElectraSyn 2.0 vial (5 mL) with a stir bar was charged with carboxylic acid **5** (0.3 mmol, 1 equiv.), alcohol **2** (0.3 mmol, 1 equiv.), 2,4,6-collidine (0.6 mmol, 2 equiv.), $^{n}Bu_4NClO_4$ (0.3 mmol, 1 equiv.), and acetonitrile (3.0 mL). ElectraSyn 2.0 vial cap equipped

with anode (graphite) and cathode (nickel) were inserted into the mixture. The reaction mixture was electrolyzed at a constant current of 10 mA for 3 hours. Next, vial cap was removed, and electrodes were rinsed with ethyl acetate (2 mL), which was combined with the crude mixture. Then, the crude mixture was further diluted with ethyl acetate (20 mL) and the resulting mixture was washed with 2N HCl (10 mL) and $NaHCO_3$ (aq) (10 mL), dried over Na_2SO_4, and concentrated in vacuo. The residue was purified by column chromatography using a gradient of hexane and ethyl acetate (eluent system) to afford the pure product.

General Procedure F for the Synthesis of Substrates 8a-8q:

With no precautions to exclude air or moisture, ElectraSyn 2.0 vial (5 mL) with a stir bar was charged with carboxylic acid **5** (0.3 mmol, 1 equiv.), MeOH (1.8 mmol, 6 equiv.), 2,4,6-collidine (0.6 mmol, 2 equiv.), $^{n}Bu_4NClO_4$ (0.3 mmol, 1 equiv.), and acetonitrile (3.0 mL). ElectraSyn 2.0 vial cap equipped with anode (graphite) and cathode (nickel) were inserted into the mixture. The reaction mixture was electrolyzed at a constant current of 10 mA for 3 hours. Next, cap was removed, and electrodes were rinsed with ethyl acetate (2 mL), which was combined with the crude mixture. Then, the crude mixture was further diluted with ethyl acetate (20 mL) and the resulting mixture was washed with 2N HCl (10 mL) and $NaHCO_3$ (aq) (10 mL), dried over Na_2SO_4, and concentrated in vacuo. The residue was purified by column chromatography using a gradient of hexane and ethyl acetate (eluent system) to afford the pure product.

3. Optimization of reaction parameters for electrochemical decarboxylative etherification

Table S1. Screening of bases[a]

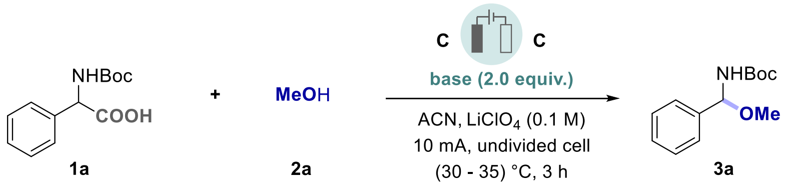

Entry	Base	Yield of **3a** (%)
1	triethylamine	n.d.
2	DBU	10
3	1,1,3,3-tetramethylguanidine	25
4	2,4,6-collidine	40
5	2,6-lutidine	36
6	No base	n.d.

Reaction conditions: [a]**1a** (0.30 mmol), **2a** (1.8 mmol), **base** (0.60 mmol), $LiClO_4$ (0.30 mmol), graphite as anode and cathode, in 3 mL acetonitrile, constant current (I) = 10 mA, in an undivided 5 mL Electrasyn 2.0 vial at (30-35) °C for 3 hours, Yield was analysed by GC-MS using mesitylene as internal standard. n.d. = not detected.

Table S2. Screening of electrolytes[a]

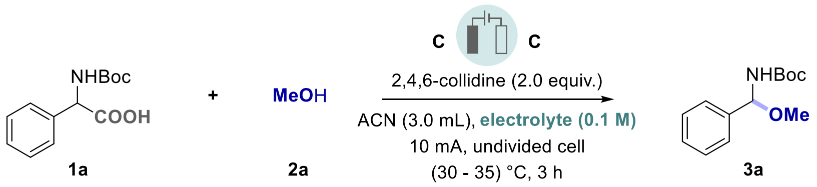

Entry	Electrolyte	Yield of **3a** (%)
1	$^{n}Bu_4NPF_6$	50
2	$^{n}Bu_4NBF_4$	40
3	$^{n}Bu_4NClO_4$	52 (46%)[b]
4	$^{n}Bu_4NBr$	12
5	$^{n}Bu_4NCl$	10
6	$^{n}Bu_4NI$	<5

Reaction conditions: [a]**1a** (0.30 mmol), **2a** (1.8 mmol), 2,4,6-collidine (0.60 mmol), **electrolyte** (0.30 mmol), graphite as anode and cathode, in 3 mL acetonitrile, constant current (I) = 10 mA, in an undivided 5 mL Electrasyn 2.0 vial at (30-35) °C for 3 hours, Yield was analysed by GC-MS using mesitylene as internal standard, [b]isolated yield.

Table S3. Effect of additives[a]

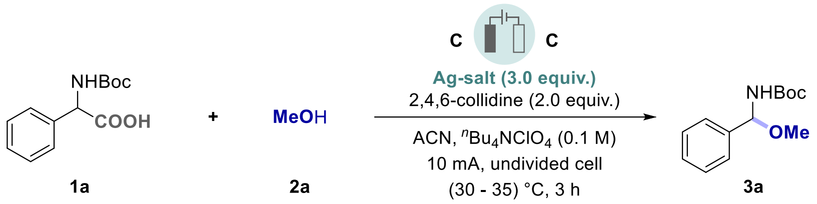

Entry	Additives	Yield of **3a** (%)
1	$AgClO_4$	53
2	$AgPF_6$	47

Reaction conditions: [a]**1a** (0.30 mmol), **2a** (1.8 mmol), 2,4,6-collidine (0.60 mmol), **additive** (0.9 mmol), graphite as anode and cathode, in 3 mL acetonitrile, constant current (I) = 10 mA, in an undivided 5 mL Electrasyn 2.0 vial at (30-35) °C for 3 hours, Yield was analysed by GC-MS using mesitylene as internal standard, n.d. = not detected.

Table S4. Screening of electrode material[a]

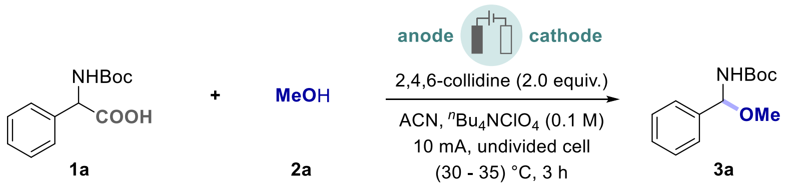

Entry	Anode	Cathode	Yield of **3a** (%)
1	Graphite	Graphite	51
2	Graphite	Nickel	82 (78)[b]
3	Graphite	Nickel foam	62
4	Graphite	Platinum	59
5	Graphite	Stainless steel	15

Reaction conditions: [a]**1a** (0.30 mmol), **2a** (1.8 mmol), 2,4,6-collidine (0.60 mmol), $^{n}Bu_4NClO_4$ (0.30 mmol), in 3 mL acetonitrile, constant current (*I*) = 10 mA, in an undivided 5 mL Electrasyn 2.0 vial at (30-35) °C for 3 hours, Yield was analysed by GC-MS using mesitylene as internal standard, [b]isolated yield.

Table S5. Screening of solvents[a]

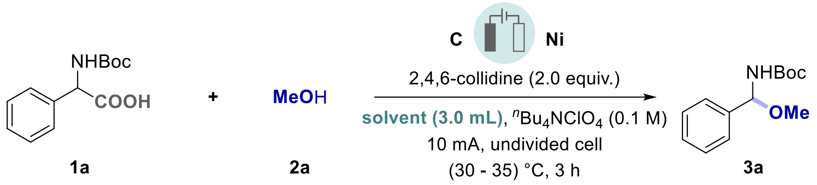

Entry	Solvent	Yield of **3a** (%)
1	DMF	n.d.
2	DMA	<5
3	DCM	68
4	Acetonitrile (ACN)	82 (78)[b]
5	DCM:ACN (1:1)	75

Reaction conditions: [a]**1a** (0.30 mmol), **2a** (1.8 mmol), 2,4,6-collidine (0.60 mmol), graphite as anode and nickel as cathode, in 3 mL **solvent**, constant current (*I*) = 10 mA, in an undivided 5 mL Electrasyn 2.0 vial at (30-35) °C for 3 hours, Yield was analysed by GC-MS using mesitylene as internal standard, n.d. = not detected.

Table S6. Screening of electric current[a]

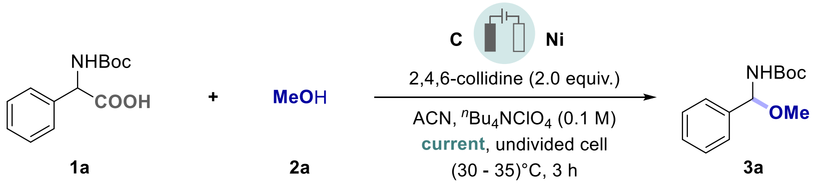

Entry	Current	Yield of **3a** (%)
1	< 10 mA	< 40
2	No current	n.d. (Only S.M.)

Reaction conditions: [a]**1a** (0.30 mmol), **2a** (1.8 mmol), 2,4,6-collidine (0.60 mmol), graphite as anode and nickel as cathode, in 3 mL acetonitrile, in an undivided 5 mL Electrasyn 2.0 vial at (30-35) °C for 3 hours, Yield was analysed by GC-MS using mesitylene as internal standard, n.d. = not detected.

4. Radical Quenching Experiments:

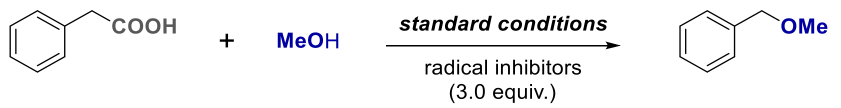

radical inhibitor	NMR yield of product
TEMPO	78%
BHT	84%

We conducted radical quenching experiments for the proposed electrochemical decarboxylation reaction. In an oven-dried Electrasyn 2.0 vial (5 mL) was added tetrabutylammonium perchlorate (100 mg, 0.1 M), phenylacetic acid (41 mg, 0.3 mmol), methanol (58 mg, 1.8 mmol), 2,4,6-collidine (73 mg, 0.6 mmol), radical inhibitors (TEMPO and BHT, 3.0 equiv.) and dissolved in 3.0 mL of acetonitrile. After that, the tube was sealed with the Electrasyn 2.0 cap containing the electrodes and stirred at constant current of 10 mA at room temperature (30-35) °C. The NMR yield of both the reactions displayed good amounts of product formation. As a result, we can easily rule out the possibility of radical formation during the progress of the reaction. We have also cross-checked our observations using HRMS studies, wherein we failed to observe any TEMPO or BHT adducts, which strongly rouled out the involvement of radical pathway.

5. Kinetic Profile

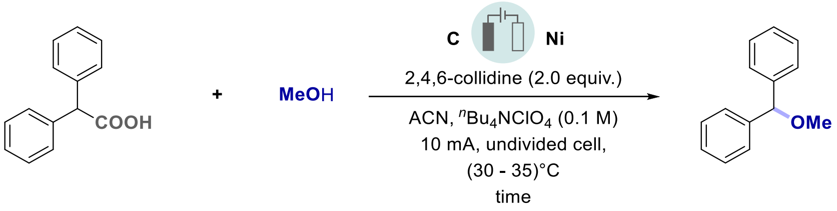

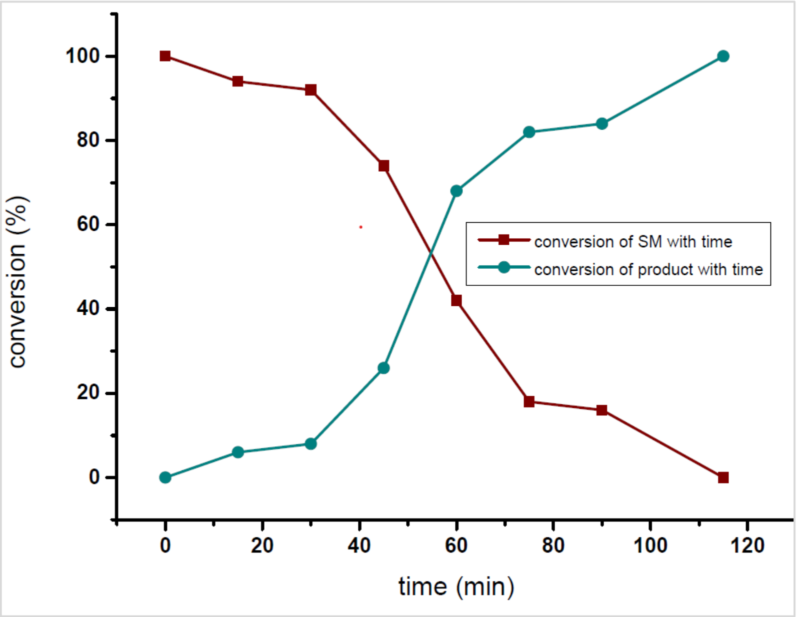

Figure S1: Time conversion plot of starting material and product with time (NMR analysis)

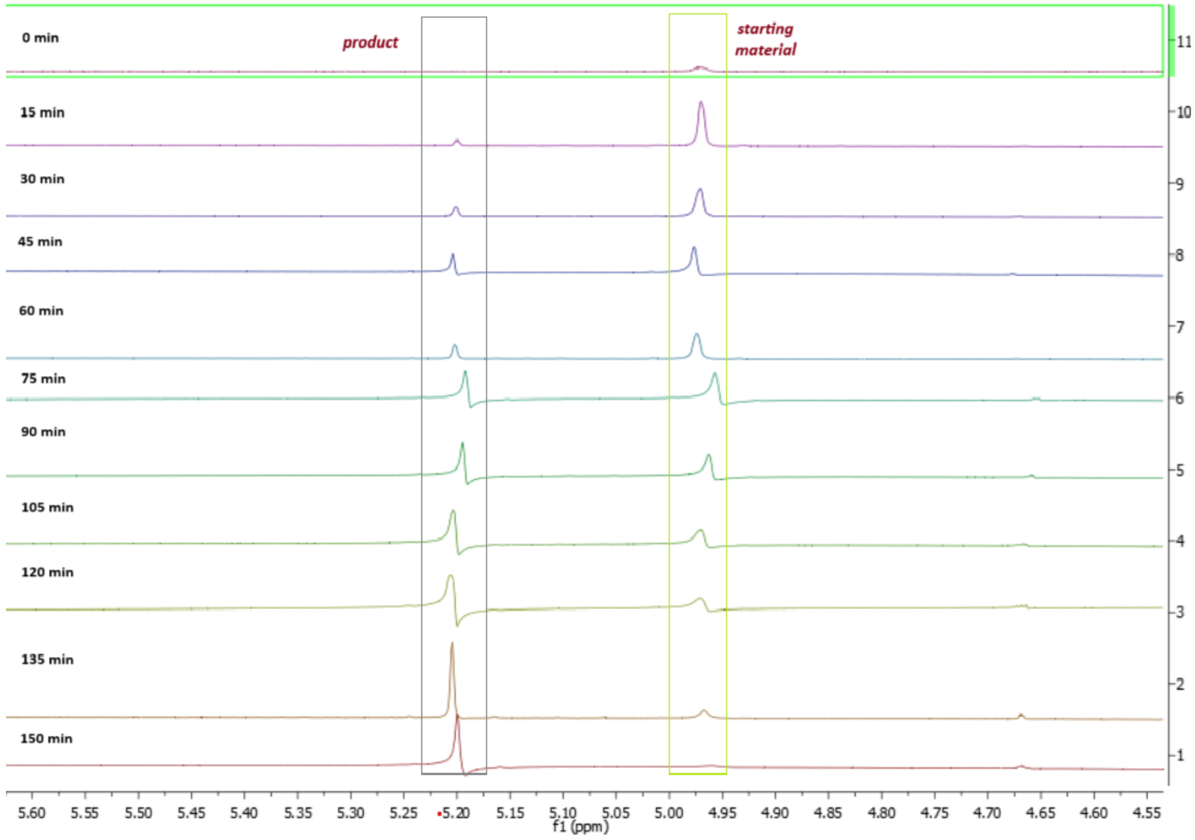

Figure S2: Time dependent in-situ NMR experiment.

The electrochemical decarboxylation was performed using 0.5 mmol of diphenyl acetic acid and 3.0 mmol of methanol in 3 mL acetonitrile as solvent under the standard reaction conditions. In an oven-dried Electrasyn 2.0 vial (5 mL) was added tetrabutylammonium perchlorate (100 mg, 0.1 M), di-phenylacetic acid (106 mg, 0.5 mmol), methanol (96 mg, 3.0 mmol), 2,4,6-collidine (122 mg, 1.0 mmol) and dissolved in 3.0 mL of acetonitrile. After that, the tube was sealed with the Electrasyn 2.0 cap containing the electrodes and stirred at constant current of 10 mA at room temperature (30-35) °C. At indicated time intervals, 100 µL of aliquot was taken out. The aliquots were analysed in NMR, plotting the conversion against time. From the plot, we observed that with time, the amounts of di-phenylacetic acid gradually decreased, and the amount of product ether increased simultaneously. The product formation reached the maximum after 120 minutes, and then quantitative conversion was observed. We did not detect any intermediates generated from the starting acid.

6. Cyclic voltammetry (CV) studies:

Cyclic voltammetry was performed in a three-electrode cell under air at room temperature (30-35) °C. A steady glassy carbon disk electrode (3 mm in diameter) was used as the working electrode, a platinum plate was used as the counter electrode, and an Ag/AgCl was used as the reference electrode in (0.1 M) KCl in acetonitrile (3.0 mL).

Acetonitrile solvent containing (0.01 M) ⁿBu4NClO4 was used as the blank. The spectrums were recorded with the scan rate of 200 mV s^{-1}, from 0 V to 2.5 V (starting from 0 V). The CV of di-phenylacetic acid and N-Boc Phenylalanine were conducted and the voltammograms are shown below. An irreversible oxidation peak for diphenyl acetic acid was observed at around 1.0 V, and irreversible oxidation peak for N-Boc phenylalanine was observed at around 1.65 V.

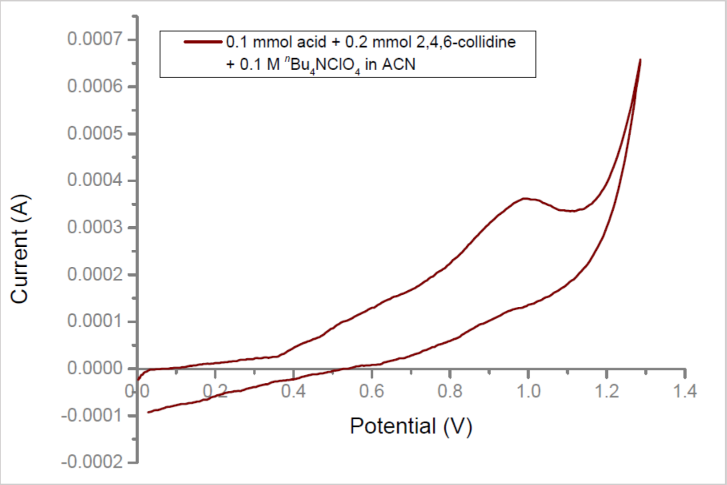

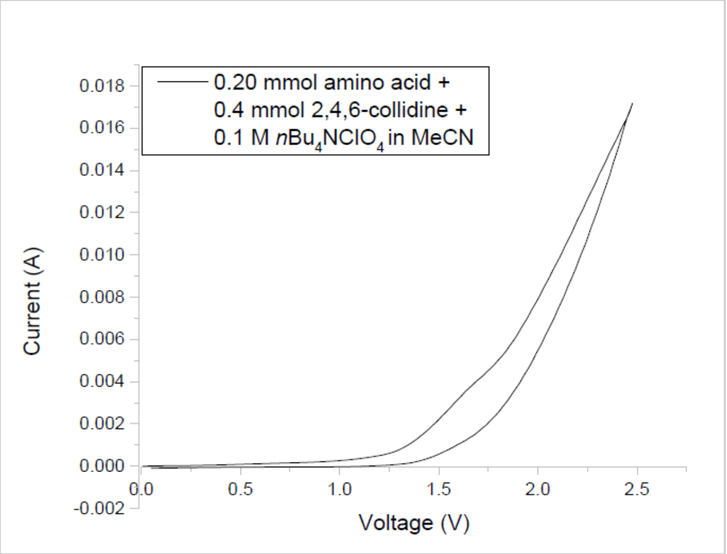

Figure S3. Cyclic voltammogram recorded in (0.1M) $^{n}Bu_4NClO_4$ in MeCN solution: scan rate: 200 mV s-1; starting potential: 0 V; glass carbon (3 mm diameter, Working Electrode); platinum plate (Counter Electrode); Ag/AgCl (0.01 M) KCl in MeCN, Reference Electrode); Concentrations: diphenyl acetic acid (0.30 mmol / 3 ml MeCN), N-Boc phenylalanine (0.3 mmol / 3 ml MeCN).

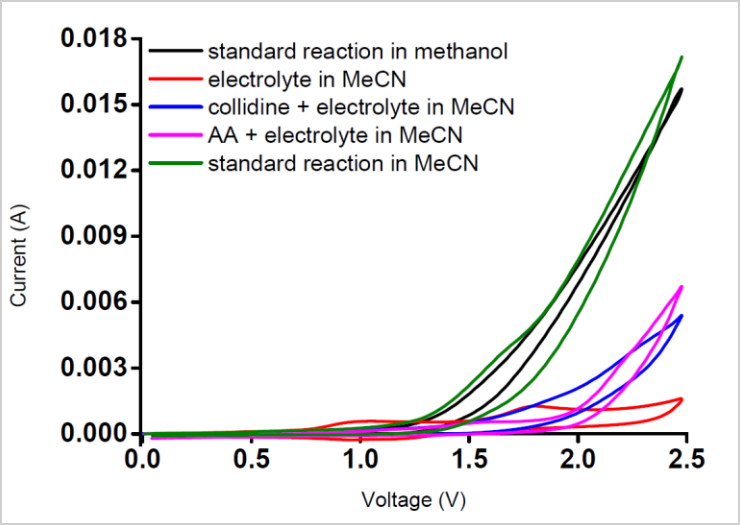

Figure S4. Control experiments for cyclic voltametric studies.

7. Crystallographic data for compound 3o

Identification code:	db_ag_24s_1337_Om		
CCDC:	2386201		
Bond Precision:	C-C 0.0026 A		Wavelength = 0.71073
Cell:	a = 9.8767(2)	b = 10.1350(2)	c = 21.8700(5)
	Alpha = 90	beta = 90	gamma = 90
Temperature:	100 K		

	**Calculated**	**Reported**
Volume	2189.20(8)	2189.19(8)
Space Group	P 21 21 21	P 21 21 21
Hall Group	P 2ac 2ab	P 2ac 2ab
Moiety Formula	C22 H35 N O3	4(C22 H35 N O3)
Sum formula	C22 H35 N O3	C88 H140 N4 O12
Mr	361.51	1446.03
Dx, g cm-3	1.097	1.097
Z	4	1
Mu (mm-1)	0.072	0.072
F000	792.0	792.0
F000’	792.34	
h,k,lmax	13,13,29	13,13,29
Nref	5438[3080]	5436
Tmin,Tmax	0.995,0.993	0.617,0.746
Tmin’	0.978	

Correction method = # Reported T Limits: Tmin = 0.617 Tmax = 0.746

AbsCorr = MULTI-SCAN

Data completeness = 1.76/1.00 Theta (max) = 28.287

R (reflections) = 0.0374 (4828) wR2 (reflections) = 0.0843

S = 1.024 Npar = 245

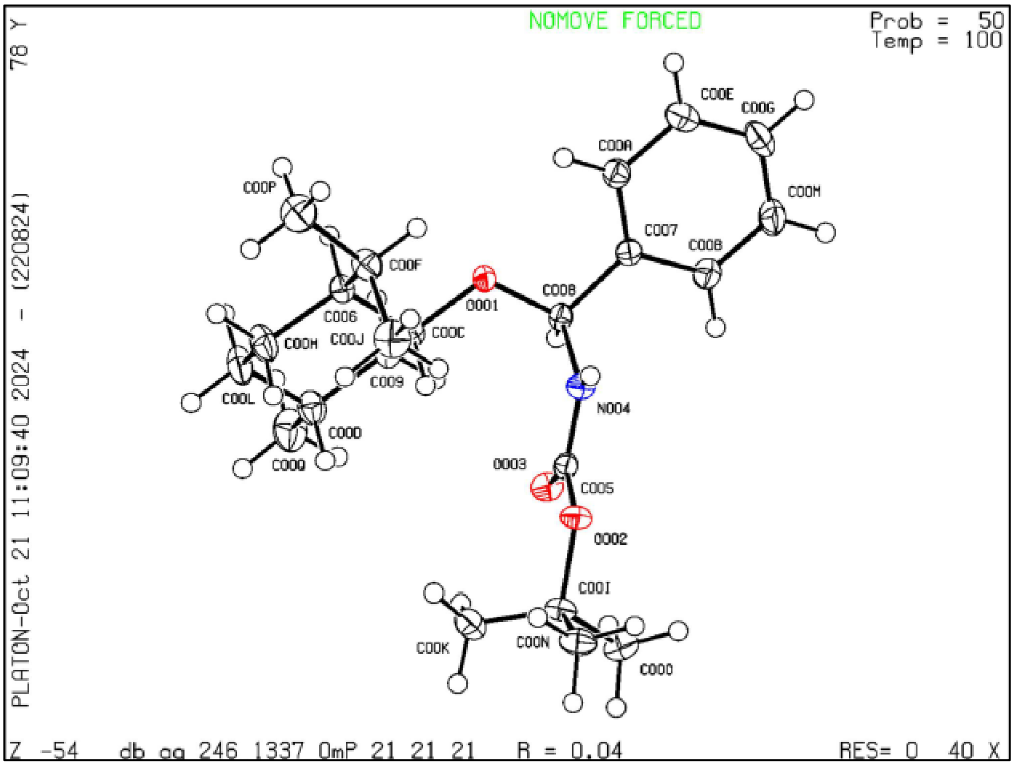

Figure S5. Platon-ellipsoid plot for compound **3o**

8. Analytical Data:

***tert*-butyl-(methoxy(phenyl)methyl)carbamate (3a):**[1]

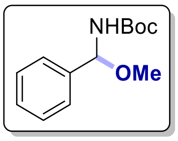

Following the general procedure **A,** the title compound was isolated as a colourless viscous liquid using silica-gel column chromatography eluting with hexane and ethyl acetate (93:7) with an isolated yield of 78% (56 mg). ^{1}H NMR (500 MHz, $CDCl_3$) δ 7.27 – 7.21 (m, 2H), 7.20 – 7.11 (m, 3H), 5.65 (d, J = 10.5 Hz, 1H), 5.03 (s, 1H), 3.27 (s, 3H), 1.30 (s, 9H). ^{13}C{^{1}H} NMR (125 MHz, $CDCl_3$) δ 154.3, 138.5, 127.5, 127.4, 124.9, 82.5, 79.1, 54.5, 27.3.

***tert*-butyl-(1-methoxy-2-phenylethyl)carbamate (3b):**

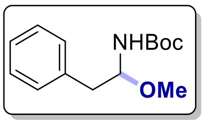

Following the general procedure **A,** the title compound was isolated as a colourless viscous liquid using silica-gel column chromatography eluting with hexane and ethyl acetate (93:7) with an isolated yield of 85% (64 mg). ^{1}H NMR (500 MHz, $CDCl_3$) δ 7.32 – 7.26 (m, 2H), 7.22 (ddd, J = 6.1, 5.4, 3.9 Hz, 3H), 5.13 – 5.04 (m, 1H), 4.80 (d, J = 11.6 Hz, 1H), 3.32 (s, 3H), 2.90 (d, J = 6.1 Hz, 2H), 1.40 (s, 9H). ^{13}C{^{1}H} NMR (125 MHz, $CDCl_3$) δ 155.4, 136.3, 129.8, 128.4, 126.7, 82.9, 79.8, 55.6, 41.8, 28.3.

HRMS (ESI-TOF): m/z $[M+H]^+$ calcd for $C_{14}H_{22}NO_3$: 252.1594; found: 252.1597.

tert-butyl(1-methoxyethyl)carbamate (3c):

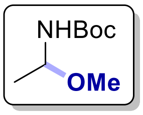

Following the general procedure **A,** the title compound was isolated as a colourless liquid using silica-gel column chromatography eluting with hexane and ethyl acetate (93:7) with an isolated yield of 82% (43 mg). ^{1}H NMR (500 MHz, $CDCl_3$) δ 4.94 (dd, J = 10.5, 5.8 Hz, 1H), 4.89 (s, 1H), 3.29 (s, 3H), 1.42 (s, 9H), 1.28 (d, J = 7.7 Hz, 3H). ^{13}C{^{1}H} NMR (125 MHz, $CDCl_3$) δ 155.3, 79.7, 79.5, 55.2, 28.4, 21.7.

HRMS (ESI-TOF): m/z $[M+H]^+$ calcd for $C_8H_{18}NO_3$: 176.1281; found: 176.1285.

tert-butyl (1-methoxy-2-methylpropyl)carbamate (3d):[1]

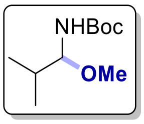

Following the general procedure **A,** the title compound was isolated as a colourless liquid using silica-gel column chromatography eluting with hexane and ethyl acetate (93:7) with an isolated yield of 74% (45 mg). ^{1}H NMR (500 MHz, $CDCl_3$) δ 4.77 (s, 1H), 4.53 – 4.51 (m, 1H), 3.28 (s, 3H), 1.77 – 1.73 (m, 1H), 1.41 (s, 9H), 0.87 (d, J = 10 Hz, 6H). ^{13}C{^{1}H} NMR (125 MHz, $CDCl_3$) δ 155.9, 87.2, 79.6, 55.6, 33.11, 28.3, 17.8, 17.3.

tert-butyl (phenyl(1-phenylethoxy)methyl)carbamate (3e):

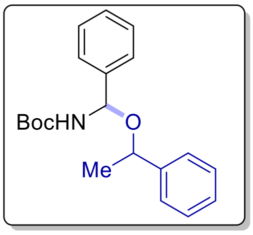

Following the general procedure **A,** the title compound was isolated as a colourless liquid using silica-gel column chromatography eluting with hexane and ethyl acetate (95:5) with an isolated yield of 67% (65 mg). **Diastereomer 1: Diastereomer 2:** 59:41.

Diastereomer 1: ^{1}H NMR (500 MHz, $CDCl_3$) δ 7.47-7.46 (m, 1H), 7.39-7.36 (m, 5H), 7.35-7.30 (m, 3H), 7.29-7.25 (m, 1H), 6.08 (d, J = 5.6 Hz, 1H), 5.20 – 5.18 (m, 1H), 4.87 (dd, J = 12.9, 6.5 Hz, 1H), 1.52 (d, J = 5.0 Hz, 3H), 1.41 (s, 9H). ^{13}C{^{1}H} NMR (125 MHz, $CDCl_3$), δ 155.3, 144.4, 140.3, 128.7, 128.44, 128.2, 127.3, 126.2, 80.8, 80.0, 75.2, 28.4, 24.4.

Diastereomer 2: ^{1}H NMR (500 MHz, $CDCl_3$) δ 7.47-7.46 (m, 1H), 7.39-7.36 (m, 5H), 7.35-7.30 (m, 3H), 7.29-7.25 (m, 1H), 5.85 (d, J = 5.6 Hz, 1H), 5.06 – 5.04 (m, 1H), 4.76-4.75 (m, 1H), 1.52 (d, J = 10 Hz, 3H), 1.51 (s, 9H). ^{13}C{^{1}H} NMR (125 MHz, $CDCl_3$), δ 154.9, 143.3, 140.2, 128.5, 128.41, 127.7, 126.8, 126.1, 80.8, 79.8, 75.0, 28.3, 23.6.

HRMS (ESI-TOF): m/z $[M+H]^+$ calcd for $C_{20}H_{26}NO_3$: 328.1913; found: 328.1910.

***tert*-butyl (2-phenyl-1-(1-phenylethoxy)ethyl)carbamate (3f):**

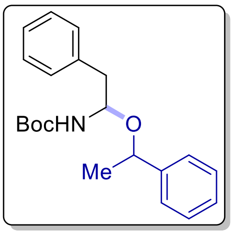

Following the general procedure **B,** the title compound was isolated as a colourless liquid using silica-gel column chromatography eluting with hexane and ethyl acetate (95:5) with an isolated yield of 66% (67 mg). ^{1}H NMR (500 MHz, $CDCl_3$) δ 7.23 – 7.22 (m, 1H), 7.20 – 7.19 (m, 4H), 7.18 – 7.15 (m, 3H), 7.14 – 7.12 (m, 2H), 5.33 – 5.24 (m, 1H), 4.58 (dd, *J* = 12.9, 6.5 Hz, 1H), 2.86 (d, *J* = 5.6 Hz, 2H), 1.23 (s, 9H), 1.21 (d, *J* = 6.4 Hz, 3H). ^{13}C{^{1}H} NMR (125 MHz, $CDCl_3$) *δ* 155.0, 144.9, 136.7, 129.9, 128.3, 127.1, 126.6, 125.9, 80.7, 79.7, 75.4, 42.5, 28.3, 22.9.

HRMS (ESI-TOF): m/z $[M+H]^+$ calcd for $C_{21}H_{28}NO_3$: 342.2069; found: 342.2060.

***tert*-butyl (1-(1-phenylethoxy)ethyl)carbamate (3g):**

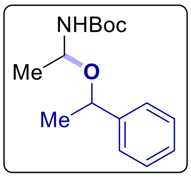

Following the general procedure **B,** the title compound was isolated as a colourless liquid using silica-gel column chromatography eluting with hexane and ethyl acetate (95:5) with an isolated yield of 63% (50 mg). **Diastereomer 1**: **Diastereomer 2** = 58:42.

Diastereomer 1: ^{1}H NMR (500 MHz, $CDCl_3$) *δ* 7.33-7.29 (m, 4H), 7.28 – 7.26 (m, 1H), 5.04 (s, 1H), 4.70 (dd, *J* = 13.8, 6.9 Hz, 1H), 1.45 (s, 9H), 1.39 (d, *J* = 6.4 Hz, 3H), 1.23 (d, *J* = 5.8 Hz, 3H). ^{13}C{^{1}H} NMR (125 MHz, $CDCl_3$) *δ* 155.2, 143.8, 128.6, 127.6, 126.6, 125.5, 81.7, 79.7, 74.7, 28.4, 25.3, 19.0.

Diastereomer 2: ^{1}H NMR (500 MHz, $CDCl_3$) *δ* 7.33-7.29 (m, 4H), 7.28 – 7.26 (m, 1H), 5.07 (d, *J* = 20.1 Hz, 1H), 4.96 – 4.83 (m, 1H), 1.43 (s, 9H), 1.32 (d, *J* = 7.3 Hz, 3H), 1.23 (d, *J* = 5.8 Hz, 3H). ^{13}C{^{1}H} NMR (125 MHz, $CDCl_3$) *δ* 155.2, 143.8, 128.4, 127.5, 126.6, 125.5, 81.7, 79.7, 76.2, 28.0, 24.6, 22.5.

HRMS (ESI-TOF): m/z $[M+H]^+$ calcd for $C_{15}H_{24}NO_3$: 266.1756; found: 266.1750.

tert-butyl (2-methyl-1-(1-phenylethoxy)propyl)carbamate (3h):

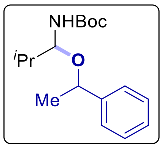

Following the general procedure **B,** the title compound was isolated as a colourless liquid using silica-gel column chromatography eluting with hexane and ethyl acetate (95:5) with an isolated yield of 59% (46 mg). **Diastereomer 1**: **Diastereomer 2** = 53:47.

Diastereomer 1: ^{1}H NMR (500 MHz, $CDCl_3$) δ 7.31 (dd, J = 6.2, 2.3 Hz, 4H), 7.24 (d, J = 7.9 Hz, 1H), 4.79 (d, J = 10.4 Hz, 1H), 4.61 (dd, J = 7.4, 5.6 Hz, 1H), 1.71 – 1.67 (m, 1H), 1.46 (s, 9H), 1.39 (d, J = 6.4 Hz, 3H), 0.82 (t, J = 6.3 Hz, 6H). ^{13}C{^{1}H} NMR (125 MHz, $CDCl_3$) δ 155.6, 143.5, 128.6, 128.3, 127.5, 127.0, 125.5, 83.4, 79.5, 74.3, 33.5, 28.42, 25.3, 18.0, 17.5.

Diastereomer 1: ^{1}H NMR (500 MHz, $CDCl_3$) δ 7.31 (dd, J = 6.2, 2.3 Hz, 4H), 7.24 (d, J = 7.9 Hz, 1H), 4.79 (d, J = 10.4 Hz, 1H), 4.48 – 4.45 (m, 1H), 1.71 – 1.67 (m, 1H), 1.46 (s, 9H), 1.39 (d, J = 6.4 Hz, 3H), 0.82 (t, J = 6.3 Hz, 6H). ^{13}C{^{1}H} NMR (125 MHz, $CDCl_3$) δ 155.6, 143.5, 128.6, 128.3, 127.5, 127.0, 125.5, 83.4, 79.5, 70.5, 31.0, 28.40, 24.3, 18.0, 17.5.

HRMS (ESI-TOF): m/z $[M+H]^+$ calcd for $C_{17}H_{28}NO_3$: 294.2069; found: 294.2060.

tert-butyl ((cyclopropylmethoxy)(phenyl)methyl)carbamate (3i):

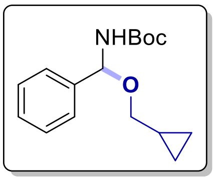

Following the general procedure **B,** the title compound was isolated as a colourless liquid using silica-gel column chromatography eluting with hexane and ethyl acetate (95:5) with an isolated yield of 80% (66 mg). ^{1}H NMR (500 MHz, $CDCl_3$) δ 7.44 – 7.41 (m, 2H), 7.37 – 7.30 (m, 2H), 7.28 (dd, J = 6.7, 1.9 Hz, 1H), 5.93 (d, J = 10.6 Hz, 1H), 5.17 (d, J = 11.1 Hz, 1H), 3.51 (dd, J = 10.6, 7.3 Hz, 1H), 3.38 (dd, J = 10.4, 7.0 Hz, 1H), 1.45 (s, 9H), 1.14 – 1.09 (m, 1H), 0.54 – 0.50 (m, 2H), 0.25 – 0.18 (m, 2H). ^{13}C{^{1}H} NMR (125 MHz, $CDCl_3$) δ 155.3, 139.9, 128.6, 128.4, 126.1, 81.9, 80.1, 72.9, 28.4, 10.7, 3.3, 3.0. HRMS (ESI-TOF): m/z $[M+H]^+$ calcd for $C_{16}H_{24}NO_3$: 278.1756; found: 278.1706.

***tert*-butyl ((cyclobutylmethoxy)(phenyl)methyl)carbamate (3j):**

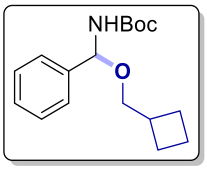

Following the general procedure **B,** the title compound was isolated as a colourless liquid using silica-gel column chromatography eluting with hexane and ethyl acetate (95:5) with an isolated yield of 76% (66 mg). ^{1}H NMR (500 MHz, $CDCl_3$) δ 7.44 – 7.40 (m, 2H), 7.34 (ddd, J = 6.4, 2.7, 1.0 Hz, 2H), 7.31 – 7.28 (m, 1H), 5.90 (d, J = 10.5 Hz, 1H), 5.13 (d, J = 11.0 Hz, 1H), 3.67 (dd, J = 9.9, 7.0 Hz, 1H), 3.51 (dd, J = 9.9, 7.3 Hz, 1H), 2.63 (qd, J = 7.6, 3.7 Hz, 1H), 2.09 – 2.02 (m, 2H), 1.92 – 1.84 (m, 2H), 1.81 – 1.75 (m, 2H), 1.47 (s, 9H). ^{13}C{^{1}H} NMR (125 MHz, $CDCl_3$) δ 155.4, 140.0, 128.6, 128.4, 126.1, 82.2, 80.1, 72.7, 35.1, 28.4, 25.2, 25.0, 18.7.
HRMS (ESI-TOF): m/z $[M+H]^+$ calcd for $C_{17}H_{26}NO_3$: 292.1913; found: 292.1910.

***tert*-butyl (E)-(((3,7-dimethylocta-2,6-dien-1-yl)oxy)(phenyl)methyl)carbamate (3k):**

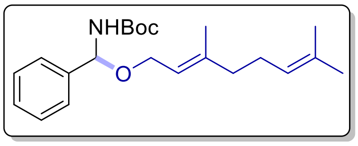

Following the general procedure **B,** the title compound was isolated as a colourless liquid using silica-gel column chromatography eluting with hexane and ethyl acetate (95:5) with an isolated yield of 65% (70 mg). ^{1}H NMR (500 MHz, $CDCl_3$) δ 7.43 (d, J = 7.3 Hz, 2H), 7.34 (dd, J = 8.1, 6.6 Hz, 2H), 7.29 (t, J = 7.2 Hz, 1H), 5.95 (d, J = 9.8 Hz, 1H), 5.44 – 5.38 (m, 1H), 5.09 (tdd, J = 4.2, 3.4, 1.4 Hz, 1H), 4.22 (dd, J = 11.5, 6.6 Hz, 1H), 4.11 (dd, J = 11.4, 7.0 Hz, 1H), 2.10 – 2.01 (m, 4H), 1.66 – 1.65 (m, 6H), 1.59 (s, 3H), 1.47 (s, 9H). ^{13}C{^{1}H} NMR (125 MHz, $CDCl_3$) δ 155.3, 140.6, 139.9, 128.6, 128.3, 126.1, 124.1, 120.5, 81.8, 80.0, 64.8, 39.7, 28.4, 26.5, 25.7, 17.7, 16.6.
HRMS (ESI-TOF): m/z $[M+Na]^+$ calcd for $C_{22}H_{33}NaNO_3$: 382.2358; found: 382.2351.

***tert*-butyl (((3,7-dimethyloct-6-en-1-yl)oxy)(phenyl)methyl)carbamate (3l):**

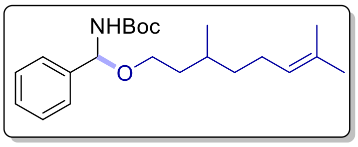

Following the general procedure **B,** the title compound was isolated as a colourless liquid using silica-gel column chromatography eluting with hexane and ethyl acetate (96:4) with an isolated yield of 70% (76 mg). ^{1}H NMR (500 MHz, $CDCl_3$) δ 7.45 – 7.40 (m, 2H), 7.34 (t, J = 7.7 Hz, 2H), 7.29 (t, J = 7.4

Hz, 1H), 5.91 (d, J = 10.9 Hz, 1H), 5.11 – 5.08 (m, 1H), 3.74 – 3.55 (m, 2H), 1.98 – 1.96 (m, 2H), 1.67 (s, 3H), 1.61 (dd, J = 11.1, 3.8 Hz, 1H), 1.59 (s, 3H), 1.54 – 1.50 (m, 1H), 1.47 (s, 9H), 1.40 – 1.27 (m, 2H), 1.25 – 1.06 (m, 1H), 0.89 (t, J = 7.2 Hz, 3H). $^{13}C\{^1H\}$ NMR (125 MHz, $CDCl_3$) δ 155.4, 140.1, 131.2, 128.6, 128.3, 126.0, 124.9, 82.2, 82.1, 80.1, 66.5, 66.4, 55.3, 37.3, 37.2, 36.7, 36.6, 29.6, 28.5, 28.45, 28.43, 28.40, 25.8, 25.5, 19.62, 19.59, 17.7.

HRMS (ESI-TOF): m/z $[M+H]^+$ calcd for $C_{22}H_{36}NO_3$: 362.2695; found: 362.2691.

***tert*-butyl-(2-phenyl-1-(2,2,2-trifluoroethoxy)ethyl)carbamate (3m):**

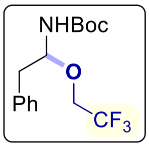

Following the general procedure **B,** the title compound was isolated as a white solid using silica-gel column chromatography eluting with hexane and ethyl acetate (96:4) with an isolated yield of 51% (49 mg). ^1H NMR (500 MHz, $CDCl_3$) δ 7.32 – 7.27 (m, 3H), 7.24 (ddd, J = 8.4, 6.8, 2.4 Hz, 2H), 5.29 (d, J = 5.2 Hz, 1H), 4.98 (d, J = 10.8 Hz, 1H), 3.94 – 3.91 (m, 2H), 2.96 (d, J = 5.9 Hz, 2H), 1.40 (s, 9H). $^{13}C\{^1H\}$ NMR (125 MHz, $CDCl_3$) δ 155.4, 135.4, 129.9, 128.6, 127.0, 125.1, 122.9, 82.9, 80.6, 65.9, 65.7, 41.4, 28.3. ^{19}F NMR (470 MHz, $CDCl_3$) δ -74.34.

HRMS (ESI-TOF): m/z $[M+H]^+$ calcd for $C_{15}H_{21}F_3NO_3$: 320.1474; found: 320.1472.

***tert*-butyl -(1-((1,1,1,3,3,3-hexafluoropropan-2-yl)oxy)-2-phenylethyl)carbamate (3n):**

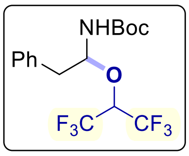

Following the general procedure **B,** the title compound was isolated as a white solid using silica-gel column chromatography eluting with hexane and ethyl acetate (93:7) with an isolated yield of 62% (71 mg). ^1H NMR (500 MHz, $CDCl_3$) δ 7.34 – 7.27 (m, 3H), 7.23 (dd, J = 8.4, 1.5 Hz, 2H), 5.54 – 5.50 (m, 1H), 5.17 (s, 1H), 3.03 – 3.00 (m, 2H), 1.41 (s, 9H). $^{13}C\{^1H\}$ NMR (125 MHz, $CDCl_3$) δ 155.9, 134.5, 129.9, 128.7, 127.3, 84.7, 81.3, 73.9, 40.9, 28.1. ^{19}F NMR (470 MHz, $CDCl_3$) δ -74.2, -74.4.

HRMS (ESI-TOF): m/z $[M+H]^+$ calcd for $C_{16}H_{20}F_6NO_3$: 388.1347; found: 388.1339.

***tert*-butyl (((2-isopropyl-5-methylcyclohexyl)oxy)(phenyl)methyl)carbamate (3o):**

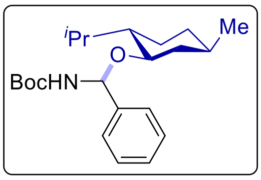

Following the general procedure **B,** the title compound was isolated as a colourless liquid using silica-gel column chromatography eluting with hexane and ethyl acetate (93:7) with an isolated yield of 78% (84 mg). **Diastereomer 1: Diastereomer 2** = 57:43.

Diastereomer 1. ^{1}H NMR (500 MHz, $CDCl_3$) δ 7.31 – 7.24 (m, 2H), 7.23 – 7.16 (m, 2H), 7.15 – 7.11 (m, 2H), 5.95 (d, J = 10.7 Hz, 1H), 3.38 (ddd, J = 15.3, 11.1, 3.7 Hz, 1H), 2.16 – 2.01 (m, 2H), 1.52 – 1.46 (m, 2H), 1.32 (s, 9H), 1.14 – 1.04 (m, 2H), 0.96 – 0.82 (m, 2H), 0.76 (d, J = 7.2 Hz, 6H), 0.65 (d, J = 7.1 Hz, 3H). ^{13}C{^{1}H} NMR (125 MHz, $CDCl_3$) δ 155.6, 140.9, 128.5, 128.3, 126.1, 82.2, 78.9, 76.0, 49.0, 42.8, 34.6, 31.9, 28.4, 25.5, 23.2, 22.4, 21.4, 16.0. **Diastereomer 2.** ^{1}H NMR (500 MHz, $CDCl_3$) δ 7.31 – 7.24 (m, 2H), 7.23 – 7.16 (m, 2H), 7.15 – 7.11 (m, 2H), 5.87 (d, J = 11.1 Hz, 1H), 3.38 (ddd, J = 15.3, 11.1, 3.7 Hz, 1H), 2.16 – 2.01 (m, 2H), 1.52 – 1.46 (m, 2H), 1.32 (s, 9H), 1.14 – 1.04 (m, 2H), 0.96 – 0.82 (m, 2H), 0.71 (d, J = 7.3 Hz, 6H), 0.51 (d, J = 7.4 Hz, 3H). ^{13}C{^{1}H} NMR (125 MHz, $CDCl_3$) δ 155.1, 140.4, 128.5, 128.1, 126.1, 79.9, 78.4, 76.0, 48.0, 40.5, 34.5, 31.5, 28.4, 25.4, 23.0, 22.4, 21.2, 15.9.

***tert*-butyl (phenyl(1,7,7-trimethylbicyclo[2.2.1]heptan-2-yl)oxy)methyl)carbamate (3p):**

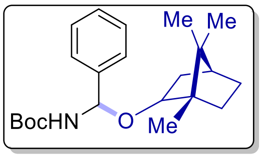

Following the general procedure **B,** the title compound was isolated as a colourless liquid using silica-gel column chromatography eluting with hexane and ethyl acetate (93:7) with an isolated yield of 67% (72 mg). **Diastereomer 1: Diastereomer 2** = 55:45.

Diastereomer 1. ^{1}H NMR (500 MHz, $CDCl_3$) δ 7.31 (d, J = 7.5 Hz, 2H), 7.19 (t, J = 7.3 Hz, 2H), 7.15 (d, J = 7.2 Hz, 1H), 5.84 (d, J = 10.3 Hz, 1H), 3.86 – 3.84 (m, 1H), 2.10 – 2.02 (m, 1H), 1.62 – 1.48 (m, 2H), 1.31 (s, 9H), 1.14 – 1.10 (m, 2H), 1.09 – 1.08 (m, 1H), 0.92 (dd, J = 13.1, 3.3 Hz, 1H), 0.73 (s, 3H), 0.70 (s, 3H), 0.69 (s, 3H). ^{13}C{^{1}H} NMR (125 MHz, $CDCl_3$) δ 155.8, 140.9, 134.6, 128.4, 128.2, 126.2, 83.9, 81.0, 80.0, 49.7, 47.8, 45.2, 37.2, 28.4, 28.4, 27.0, 19.8, 19.0, 14.0.

Diastereomer 2. ^{1}H NMR (500 MHz, $CDCl_3$) δ 7.31 (d, J = 7.5 Hz, 2H), 7.19 (t, J = 7.3 Hz, 2H), 7.15 (d, J = 7.2 Hz, 1H), 5.81 (d, J = 10.4 Hz, 1H), 3.78 – 3.76 (m, 1H), 1.96 – 1.90 (m, 1H), 1.62 – 1.48 (m, 2H), 1.31 (s, 9H), 1.14 – 1.10 (m, 2H), 1.09 – 1.08 (m, 1H), 0.92 (dd, J = 13.1, 3.3 Hz, 1H), 0.72 (s, 3H), 0.69 (s, 3H), 0.67 (s, 3H). ^{13}C{^{1}H} NMR (125 MHz, $CDCl_3$) δ 155.1, 140.4, 134.6, 128.4, 128.1, 126.2, 82.8, 80.2, 79. 9, 49.1, 47.4, 45.1, 35.9, 28.4, 28.3, 26.9, 19.8, 18.9, 13.7.

HRMS (ESI-TOF) *m/z:* $[M+H]^+$ Calcd for $[C_{22}H_{34}NO_3]^+$ 360.2533; Found 360.2538.

tert-butyl(17-acetyl-10,13-dimethyl-2,3,4,7,8,9,10,11,12,13,14,15,16,17-tetradecahydro-1H-cyclopenta[a]phenanthren-3-yl)oxy)(phenyl)methyl)carbamate (3q):

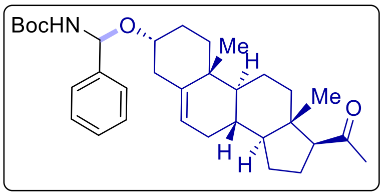

Following the general procedure **B,** the title compound was isolated as a colourless liquid using silica-gel column chromatography eluting with hexane and ethyl acetate (93:7) with an isolated yield of 71% (110 mg). ^{1}H NMR (500 MHz, $CDCl_3$) δ 7.28 (d, J = 7.4 Hz, 2H), 7.22 – 7.17 (m, 2H), 7.15 (dd, J = 6.8, 0.6 Hz, 1H), 5.91 (d, J = 9.1 Hz, 1H), 5.23 – 5.11 (m, 1H), 3.42 – 3.28 (m, 1H), 2.36 (t, J = 8.8 Hz, 1H), 2.31 – 2.17 (m, 1H), 2.15 – 2.06 (m, 1H), 2.04 – 2.02 (m, 1H), 1.96 (s, 3H), 1.92 – 1.77 (m, 3H), 1.70 (ddd, J = 13.5, 8.1, 3.1 Hz, 1H), 1.58 – 1.40 (m, 6H), 1.31 (s, 9H), 1.15 – 1.02 (m, 3H), 1.01 – 0.94 (m, 1H), 0.90 (dd, J = 13.7, 3.0 Hz, 1H), 0.84 (s, 3H), 0.75 – 0.67 (m, 1H), 0.46 (s, 3H). ^{13}C{^{1}H} NMR (125 MHz, $CDCl_3$) δ 209.8, 155.1, 140.7, 128.6, 128.3, 126.1, 121.6, 114.4, 80.4, 80.1, 71.8, 63.8, 57.0, 55.7, 50.1, 44.1, 42.3, 39.8, 38.9, 37.4, 37.3, 36.9, 31.9, 31.6, 29.3, 28.4, 28.4, 24.6, 22.9, 21.1, 19.5, 13.3.

HRMS (ESI-TOF) *m/z:* $[M+H]^+$ Calcd for $[C_{33}H_{48}NO_4]^+$ 522.3578; Found 522.3580.

tert-butyl(10,13-dimethyl-3-oxo-2,3,6,7,8,9,10,11,12,13,14,15,16,17-tetradecahydro-1H-cyclopenta[a]phenanthren-17-yl)oxy)(phenyl)methyl)carbamate (3r):

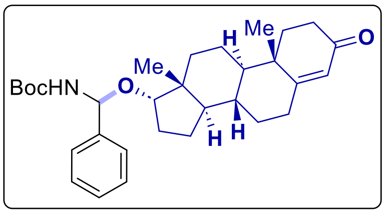

Following the general procedure **B,** the title compound was isolated as a colourless liquid using silica-gel column chromatography eluting with hexane and ethyl acetate (92:8) with an isolated yield of 85% (125 mg). **Diastereomer 1: Diastereomer 2** = 53:47.

Diastereomer 1. ^{1}H NMR (500 MHz, $CDCl_3$) δ 7.32 – 7.25 (m, 2H), 7.23 – 7.12 (m, 3H), 6.62 (s, 1H), 5.57 (s, 1H), 3.51 (ddd, J = 19.4, 15.3, 6.7 Hz, 1H), 2.28 – 2.12 (m, 2H), 2.03 – 1.82 (m, 2H), 1.80 – 1.65 (m, 2H), 1.63 – 1.37 (m, 7H), 1.29 (s, 9H), 1.33 – 1.26 (m, 4H), 1.04 (s, 3H), 0.91 – 0.82 (m, 3H), 0.75 (s, 3H). ^{13}C{^{1}H} NMR (125 MHz, $CDCl_3$) δ 199.7, 171.5, 157.4, 147.6, 140.6, 128.5, 126.1, 123.9, 121.3, 87.3, 82.4, 80.7, 54.0, 50.6, 43.2, 38.7, 37.1, 35.8, 35.5, 34.0, 32.9, 31.6, 28.5, 28.3, 27.7, 24.3, 23.6, 20.9, 20.7, 17.5, 12.0.

Diastereomer 2. ^{1}H NMR (500 MHz, $CDCl_3$) δ 7.32 – 7.25 (m, 2H), 7.23 – 7.12 (m, 3H), 5.60 – 5.52 (s, 1H), 5.02 – 4.99 (m, 1H), 3.51 (ddd, J = 19.4, 15.3, 6.7 Hz, 1H), 2.28 – 2.12 (m, 2H), 2.03 – 1.82 (m, 2H), 1.80 – 1.65 (m, 2H), 1.63 – 1.37 (m, 7H), 1.29 (s, 9H), 1.33 – 1.26 (m, 4H), 1.04 (s, 3H), 0.91 – 0.82 (m, 3H), 0.74 (s, 3H). ^{13}C{^{1}H} NMR (125 MHz, $CDCl_3$) δ 199.7, 171.4, 155.4, 147.6, 140.4, 128.2, 126.1, 123.9, 121.3, 85.2, 82.4, 80.0, 53.9, 50.2, 42.8, 38.7, 37.0, 35.7, 35.5, 34.0, 32.9, 31.6, 28.4, 28.3, 27.0, 24.3, 23. 4, 20.9, 20.7, 17.5, 11.8.
HRMS (ESI-TOF) *m/z:* $[M+H]^+$ Calcd for $[C_{31}H_{44}NO_4]^+$ 494.3265; Found 494.3269.

methyl N-(3,3-dimethylbutyl)-O-methyl-L-seryl-L-phenylalaninate (3s):

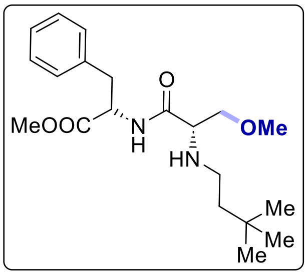

Following the general procedure **B,** the title compound was isolated as a colourless liquid using silica-gel column chromatography eluting with hexane and ethyl acetate (60:40) with an isolated yield of 45% (49 mg). 1H NMR (500 MHz, $CDCl_3$) δ 9.32 (d, J = 8.2 Hz, 1H), 7.25 (d, J = 1.8 Hz, 3H), 7.19 (d, J = 6.9 Hz, 2H), 4.77 – 4.72 (m, 1H), 3.81 (td, J = 4.2, 2.1 Hz, 1H), 3.77 (s, 3H), 3.64 (s, 3H), 3.19 – 3.13 (m, 1H), 2.98 (dd, J = 14.1, 9.2 Hz, 1H), 2.88 – 2.65 (m, 3H), 2.57 – 2.53 (m, 1H), 1.59 – 1.46 (m, 2H), 0.86 (s, 9H). ^{1}H NMR (125 MHz, $CDCl_3$) δ 171.7, 136.6, 130.3, 129.4, 129.3, 128.6, 127.1, 74.4, 58.8, 57.9, 55.3, 54.1, 52.4, 42.5, 40.6, 37.6, 29.9, 29.3.
HRMS (ESI-TOF) *m/z:* $[M+H]^+$ Calcd for $[C_{20}H_{33}N_2O_4]^+$ 365.2440; Found 365.2410.

methyl 2-(benzhydrylamino)-2-phenylacetate (6a):[2]

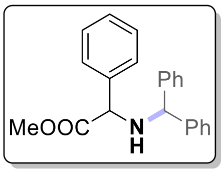

Following the general procedure **C,** the title compound was isolated as a colourless liquid using silica-gel column chromatography eluting with hexane and ethyl acetate (96:4) with an isolated yield of 79% (78 mg). ^{1}H NMR (500 MHz, $CDCl_3$) δ 7.47 – 7.26 (m, 11H), 7.23 – 7.09 (m, 4H), 4.72 (s, 1H), 4.32 (s, 1H), 3.99 – 3.98 (m, 1H), 3.68 (s, 3H). NMR (125 MHz, $CDCl_3$) δ 173.7, 143.2, 138.2, 129.0, 128.8, 128.7, 128.6, 128.5, 128.2, 127.9, 127.68, 127.65, 127.6, 127.5, 127.4, 127.3, 127.2, 64.4, 62.9, 52.3.

methyl benzhydrylphenylalaninate (6b):

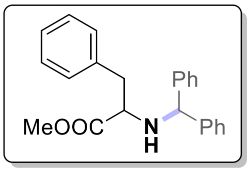

Following the general procedure **C,** the title compound was isolated as a colourless liquid using silica-gel column chromatography eluting with hexane and ethyl acetate (97:3) with an isolated yield of 88% (91 mg). ^{1}H NMR (500 MHz, $CDCl_3$) δ 7.34 – 7.25 (m, 8H), 7.24 – 7.20 (m, 2H), 7.20 – 7.16 (m, 5H), 4.79 (s, 1H), 3.67 (s, 3H), 3.47 (dd, *J* = 8.0, 5.9 Hz, 1H), 3.01 (dd, *J* = 13.7, 6.0 Hz, 1H), 2.92 (dd, *J* = 13.7, 7.9 Hz, 1H). ^{13}C{^{1}H} NMR (125 MHz, $CDCl_3$) δ 175.4, 144.3, 142.7, 137.7, 129.6, 128.6, 128.5, 128.4, 127.5, 127.4, 127.3, 127.2, 126.7, 65.5, 60.7, 51.7, 40.3.
HRMS (ESI-TOF): m/z $[M+H]^+$ calcd for $C_{23}H_{24}NO_2$: 346.1807; found: 346.1832.

ethyl benzhydrylvalinate (6c):

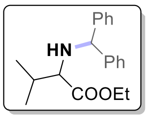

Following the general procedure **C,** the title compound was isolated as a colourless liquid using silica-gel column chromatography eluting with hexane and ethyl acetate (98:2) with an isolated yield of 70% (65 mg). ^{1}H NMR (500 MHz, $CDCl_3$) δ 7.46 (d, *J* = 7.8 Hz, 2H), 7.38 (d, *J* = 7.9 Hz, 2H), 7.33 – 7.26 (m, 4H), 7.23 – 7.17 (m, 2H), 4.74 (s, 1H), 4.20 (dd, *J* = 7.1, 3.8 Hz, 2H), 2.97 (d, *J* = 6.0 Hz, 1H), 1.94 (dd, *J* = 13.3, 6.6 Hz, 1H), 1.27 (d, *J* = 7.1 Hz, 3H), 0.98 (dd, *J* = 10.4, 6.8 Hz, 6H). ^{13}C{^{1}H} NMR (125 MHz, $CDCl_3$) δ 175.5, 128.6, 128.5, 127.8, 127.4, 127.2, 65.9, 64.9, 60.4, 31.9, 19.7, 18.6, 14.5.

HRMS (ESI-TOF): m/z $[M+Na]^+$ calcd for $C_{20}H_{25}NNaO_2$: 334.1783; found: 334.1772.

ethyl benzhydrylleucinate (6d):

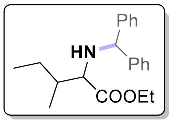

Following the general procedure **C,** the title compound was isolated as a colourless liquid using silica-gel column chromatography eluting with hexane and ethyl acetate (98:2) with an isolated yield of 74% (65 mg). ^{1}H NMR (125 MHz, $CDCl_3$) δ 7.45 – 7.42 (m, 2H), 7.36 (dd, *J* = 8.1, 0.9 Hz, 2H), 7.29 – 7.25 (m, 4H), 7.20 (ddd, *J* = 13.5, 12.0, 7.4 Hz, 2H), 4.73 (s, 1H), 4.19 (dd, *J* = 7.1, 4.1 Hz, 2H), 3.01 (d, *J*

= 6.2 Hz, 1H), 2.15 – 2.04 (m, 1H), 1.66 (dtdd, *J* = 19.3, 15.0, 7.1, 4.2 Hz, 2H), 1.27 (t, *J* = 7.1 Hz, 3H), 0.90 (d, *J* = 6.8 Hz, 3H), 0.85 (t, *J* = 7.4 Hz, 3H). $^{13}C\{^1H\}$ NMR (125 MHz, $CDCl_3$) δ 175.5, 144.7, 142.9, 128.6, 128.5, 127.8, 127.4, 127.2, 127.2, 65.9, 63.9, 60.4, 38.6, 25.4, 15.9, 14.5, 11.5.

HRMS (ESI-TOF): m/z $[M+H]^+$ calcd for $C_{21}H_{28}NO_2$: 326.2120.1807; found: 326.2109.

tert-butyl 3-(2-(benzylamino)-3-methoxy-3-oxopropyl)-1H-indole-1-carboxylate (6e):

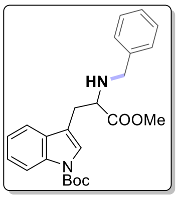

Following the general procedure **C,** the title compound was isolated as a colourless solid using silica-gel column chromatography eluting with hexane and ethyl acetate (90:10) with an isolated yield of 52% (32 mg). ^1H NMR (500 MHz, $CDCl_3$) δ 7.47-7.45 (m, 1H), 7.39-7.38 (m, 4H), 7.35-7.32 (m, 1H), 7.16-7.09 (m. 2H), 6.78 (s, 1H), 4.59 (d, *J* = 10.0 Hz, 1H), 3.93 (s, 3H), 3.60 (s, 3H), 3.11 (d, *J* = 10.0 Hz, 2H), 1.40 (s, 9H). $^{13}C\{^1H\}$ NMR (125 MHz, $CDCl_3$) δ 169.3, 162.5, 131.2, 129.5, 129.0, 128.9, 127.8, 122.1, 121.3, 120.4, 112.3, 82.2, 74.5, 41.2, 28.4, 26.2.
HRMS (ESI-TOF): m/z $[M+H]^+$ calcd for $C_{24}H_{29}N_2O_4$: 409.2127; found: 409.2110.

tert-butyl 3-(3-methoxy-3-oxo-2-((1-phenylethyl)amino)propyl)-1H-indole-1-carboxylate (6f):

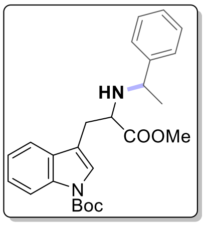

Following the general procedure **C,** the title compound was isolated as a colourless solid using silica-gel column chromatography eluting with hexane and ethyl acetate (90:10) with an isolated yield of 60% (58 mg). ^1H NMR (500 MHz, $CDCl_3$) δ 7.46 – 7.38 (m, 6H), 7.33 (d, *J* = 6.8 Hz, 1H), 7.23 – 7.21 (m, 1H), 7.13 – 7.09 (m, 2H), 5.01 (dd, *J* = 9.3, 2.6 Hz, 1H), 4.55 – 4.49 (m, 1H), 4.02 (dt, *J* = 8.6, 6.4 Hz, 1H), 3.57 (d, *J* = 2.2 Hz, 3H), 3.00 (t, *J* = 6.1 Hz, 2H), 1.66 (d, *J* = 7.2 Hz, 3H), 1.40 (d, *J* = 5.9 Hz, 9H). $^{13}C\{^1H\}$ NMR (125 MHz, $CDCl_3$) δ 172.8, 155.3, 139.1, 139.1, 131.3, 129.1, 127.9, 127.73, 127.71, 122.0, 120.4, 110.9, 79.9, 53.7, 52.3, 45.7, 28.4, 26.1, 18.2.
HRMS (ESI-TOF): m/z $[M+H]^+$ calcd for $C_{25}H_{31}N_2O_4$: 423.2284; found: 423.2240.

tert-butyl 3-(2-(benzhydrylamino)-3-methoxy-3-oxopropyl)-1H-indole-1-carboxylate (6g):

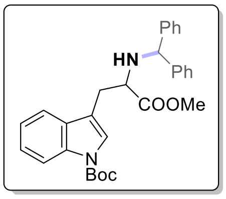

Following the general procedure **C,** the title compound was isolated as a colourless solid using silica-gel column chromatography eluting with hexane and ethyl acetate (90:10) with an isolated yield of 55% (58 mg). ^{1}H NMR (500 MHz, $CDCl_3$) δ 8.72 (s, 1H), 7.46 (d, J = 1.2 Hz, 1H), 7.42 – 7.36 (m, 11H), 7.33 (d, J = 4.9 Hz, 1H), 7.16 – 7.10 (m, 2H), 5.32 (s, 1H), 4.97 (d, J = 8.4 Hz, 1H), 4.51 (dt, J = 8.3, 6.1 Hz, 1H), 3.52 (s, 3H), 3.01 (dd, J = 7.6, 6.2 Hz, 2H), 1.38 (s, 9H). ^{13}C{^{1}H} NMR (125 MHz, $CDCl_3$) δ 172.8, 170.4, 155.3, 140.9, 137.4, 131.3, 129.0, 128.7, 127.9, 122.2, 120.5, 118.8, 111.0, 94.9, 79.9, 57.0, 53.8, 52.3, 28.4, 26.1.
HRMS (ESI-TOF): m/z [M+H]$^+$ calcd for $C_{30}H_{33}N_2O_4$: 485.2440; found: 485.2439.

1-(methoxymethyl)naphthalene (7a):[3]

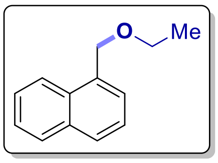

Following the general procedure **D,** the title product was obtained as a colourless oil using silica-gel column chromatography eluting with hexane and ethyl acetate (97:3) with an isolated yield of isolated yield of 92% (51 mg). ^{1}H NMR (500 MHz, $CDCl_3$) δ 8.16 – 8.14 (m, 1H), 7.89 – 7.87 (m, 1H), 7.84 - 7.81 (dd, J = 11.4, 3.1 Hz, 1H), 7.57 – 7.51 (m, 2H), 7.50 – 7.44 (dd, J = 8.4, 6.9 Hz, 2H), 4.97 (s, 2H), 3.65 (q, J = 7.1 Hz, 2H), 1.30 (t, J = 5 Hz, 3H). ^{13}C{^{1}H} NMR (125 MHz, $CDCl_3$) δ 134.1, 133.9, 131.9, 128.63, 128.61, 126.4, 126.3, 125.8, 125.3, 124.1, 71.3, 65.9, 15.4.

((cyclobutylmethoxy)methylene)dibenzene (7b):

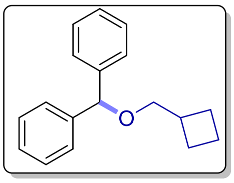

Following the general procedure **D,** the title product was obtained as a colourless oil using silica-gel column chromatography eluting with 3% ethyl acetate in hexane (Yield: 70%, 53 mg). ^{1}H NMR (500 MHz, $CDCl_3$) δ 7.40 – 7.37 (m, 4H), 7.36 – 7.32 (m, 4H), 7.28 – 7.25 (m, 2H), 5.37 – 5.36 (d, J = 4.2

Hz, 1H), 3.48 – 3.46 (m, 2H), 2.72 – 2.66 (m, 1H), 2.12 - 2.07 (m, 2H), 1.97 – 1.87 (m, 2H), 1.85 – 1.79 (m, 2H). $^{13}C\{^1H\}$ NMR (125 MHz, $CDCl_3$) δ 142.8, 128.5, 127.4, 127.1, 83.6, 73.8, 35.4, 25.3, 18.8.

HRMS (ESI-TOF): m/z $[M+H]^+$ calcd for $C_{18}H_{21}O$: 253.1592; found: 253.1598.

(((3,7-dimethyloct-6-en-1-yl)oxy)methylene)dibenzene (7c):

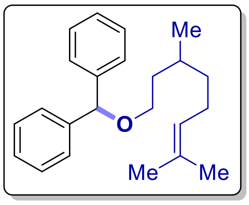

Following the general procedure **D,** the title product was obtained as a colourless oil using silica-gel column chromatography eluting with 3% ethyl acetate in hexane (Yield: 89%, 86 mg). ^1H NMR (500 MHz, $CDCl_3$) δ 7.26 – 7.24 (m, 4H), 7.22 – 7.19 (m, 4H), 7.14 – 7.11 (m, 2H), 5.23 (s, 1H), 5.02 – 4.98 (m, 1H), 3.42 – 3.34 (m, 2H), 1.95 – 1.81 (m, 2H), 1.64 – 1.60 (m, 1H), 1.58 (d, *J* = 0.8 Hz, 3H), 1.54 (dd, *J* = 13.1, 6.6 Hz, 1H), 1.50 (s, 3H), 1.40 – 1.33 (td, *J* = 13.4, 6.9 Hz, 1H), 1.27 – 1.21 (m, 1H), 1.09 – 1.01 (m, 1H), 0.78 – 0.77 (m, 3H). $^{13}C\{^1H\}$ NMR (125 MHz, $CDCl_3$) δ 142.73, 142.71, 131.1, 128.4, 127.3, 127.0, 126.9, 124.9, 83.7, 67.5, 37.2, 36.9, 29.6, 25.8, 25.5, 19.7, 17.7.

HRMS (ESI-TOF): m/z $[M+H]^+$ calcd for $C_{23}H_{31}O$: 323.2375; found: 323.2366.

1-((cyclohexyloxy)methyl)naphthalene (7d):

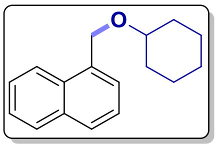

Following the general procedure **D,** the title product was obtained as a colourless oil using silica-gel column chromatography eluting with 1% ethyl acetate in hexane (Yield: 85%, 61 mg). ^1H NMR (500 MHz, $CDCl_3$) δ 8.16 (d, *J* = 8.4 Hz, 1H), 7.90 – 7.82 (m, 2H), 7.57 – 7.50 (m, 3H), 7.48 – 7.45 (m, 1H), 5.03 (s, 2H), 3.52 – 3.47 (m, 1H), 2.05 (dd, *J* = 9.1, 3.6 Hz, 2H), 1.83 – 1.80 (m, 2H), 1.59 (dd, *J* = 9.4, 3.8 Hz, 1H), 1.49 – 1.42 (m, 1H), 1.36 – 1.23 (m, 4H). $^{13}C\{^1H\}$ NMR (125 MHz, $CDCl_3$) δ 136.6, 135.3, 134.6, 133.8, 131.8, 128.5, 128.2, 126.0, 125.9, 125.6, 125.3, 124.0, 77.4, 68.3, 32.3, 25.8, 24.2.

HRMS (ESI-TOF): m/z $[M+H]^+$ calcd for $C_{17}H_{21}O$: 241.1592; found: 241.1587.

2-(benzhydryloxy)-1,7,7-trimethylbicyclo[2.2.1]heptane (7e):

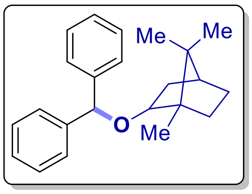

Following the general procedure **D,** the title product was obtained as a colourless oil using silica-gel column chromatography eluting with 3% ethyl acetate in hexane (Yield: 80%, 76 mg). ^{1}H NMR (500 MHz, $CDCl_3$) δ 7.41 (d, J = 7.9 Hz, 4H), 7.38 – 7.33 (m, 3H), 7.33 – 7.22 (m, 3H), 5.44 (d, J = 2.6 Hz, 1H), 3.78 – 3.73 (m, 1H), 2.34 (td, J = 9.1, 4.4 Hz, 1H), 2.13 – 2.04 (m, 1H), 1.84 – 1.73 (m, 1H), 1.69 (d, J = 3.9 Hz, 1H), 1.39 – 1.30 (m, 2H), 1.18 (dd, J = 12.8, 3.1 Hz, 1H), 0.94 – 0.89 (m, 6H), 0.79 (d, J = 2.5 Hz, 3H). ^{13}C{^{1}H} NMR (125 MHz, $CDCl_3$) δ 143.9, 142.8, 128.9, 128.5, 128.3, 128.1, 127.4, 127.3, 126.99, 126.93, 82.0, 81.6, 49.5, 47.8, 45.2, 36.1, 28.4, 27.1, 19.8, 18.9, 13.9.
HRMS (ESI-TOF): m/z $[M+Na]^+$ calcd for $C_{23}H_{28}ONa$: 343.2038; found: 343.2026.

2-(benzhydryloxy)-1,3,3-trimethylbicyclo[2.2.1]heptane (7f):

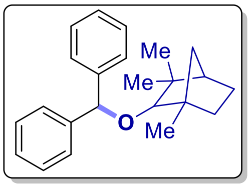

Following the general procedure **D,** the title product was obtained as a colourless oil using silica-gel column chromatography eluting with 3% ethyl acetate in hexane (Yield: 84%, 81 mg). ^{1}H NMR (500 MHz, $CDCl_3$) δ 7.42 – 7.37 (m, 4H), 7.34 – 7.31 (m, 4H), 7.23 (d, J = 1.4 Hz, 2H), 5.39 (s, 1H), 3.10 (d, J = 1.7 Hz, 1H), 2.06 – 1.99 (m, 1H), 1.78 (dd, J = 12.2, 9.2 Hz, 1H), 1.65 – 1.61 (m, 1H), 1.48 – 1.38 (m, 2H), 1.08 – 1.03 (m, 2H), 1.00 (d, J = 3.4 Hz, 6H), 0.92 (s, 3H). ^{13}C{^{1}H} NMR (125 MHz, $CDCl_3$) δ 143.6, 143.0, 141.1, 128.9, 128.5, 128.1, 128.0, 127.7, 127.3, 126.97, 126.94, 126.1, 88.7, 82.8, 49.3, 48.9, 41.9, 41.4, 39.5, 31.2, 26.3, 21.4, 20.1.
HRMS (ESI-TOF): m/z $[M+H]^+$ calcd for $C_{23}H_{29}O$: 321.2218; found: 321.2210.

(((2-isopropyl-5-methylcyclohexyl)oxy)methylene)dibenzene (7g):[4]

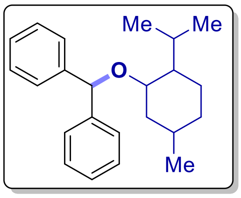

Following the general procedure **D,** the title product was obtained as a colourless oil using silica-gel column chromatography eluting with 3% ethyl acetate in hexane (Yield: 82%, 76 mg). ^{1}H NMR (500 MHz, $CDCl_3$) δ 7.26 (dd, J = 11.1, 4.1 Hz, 4H), 7.23 – 7.18 (m, 4H), 7.16 – 7.14 (m, 1H), 7.13 – 7.09 (m, 1H), 5.44 (s, 1H), 3.05 (td, J = 10.5, 4.2 Hz, 1H), 2.28 (m, J = 14.0, 7.0, 2.4 Hz, 1H), 2.07 (m, J = 12.1, 5.4, 3.8 Hz, 1H), 1.53 – 1.49 (m, 2H), 1.25 (m, J = 7.3, 5.9, 3.3 Hz, 1H), 1.20 – 1.13 (m, 1H), 0.85 – 0.82 (m, 1H), 0.79 (dd, J = 6.8, 4.8 Hz, 6H), 0.77 – 0.73 (m, 2H), 0.34 (d, J = 6.9 Hz, 3H). ^{13}C{^{1}H} NMR (125 MHz, $CDCl_3$) δ 143.9, 142.6, 128.3, 128.1, 127.9, 127.4, 126.9, 126.7, 79.9, 75.9, 48.8, 40.4, 34.6, 31.5, 25.1, 22.9, 22.4, 21.4, 15.7.

((*tert*-pentyloxy)methylene)dibenzene (7h):[5]

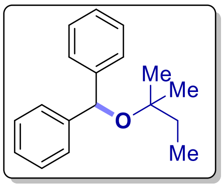

Following the general procedure **D,** the title product was obtained as a colourless oil using silica-gel column chromatography eluting with 3% ethyl acetate in hexane (Yield: 89%, 68 mg). ^{1}H NMR (500 MHz, $CDCl_3$) δ 7.19 (d, J = 7.3 Hz, 4H), 7.10 (dd, J = 10.4, 4.8 Hz, 4H), 7.01 (t, J = 7.3 Hz, 2H), 5.40 (s, 1H), 1.42 (q, J = 7.5 Hz, 2H), 0.97 (s, 6H), 0.70 (t, J = 7.5 Hz, 3H). ^{13}C{^{1}H} NMR (125 MHz, $CDCl_3$) δ 145.4, 128.1, 126.8, 126.7, 77.3, 75.4, 34.6, 25.9, 8.7.

(1-(*tert*-butoxy)cyclohexyl)benzene (7i):

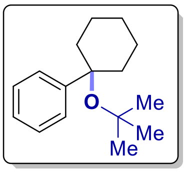

Following the general procedure **D,** the title product was obtained as a colourless oil using silica-gel column chromatography eluting with 3% ethyl acetate in hexane (Yield: 82%, 68 mg). ^{1}H NMR (500 MHz, $CDCl_3$) δ 7.25 – 7.22 (m, 2H), 7.19 – 7.14 (m, 2H), 7.07 – 7.05 (m, 1H), 2.31 – 2.10 (m, 2H), 1.96 – 1.66 (m, 4H), 1.59 – 1.44 (m, 4H), 1.10 (s, 9H). ^{13}C{^{1}H} NMR (125 MHz, $CDCl_3$) δ 139.1, 128.2, 127.1, 126.9, 126.2, 125.5, 73.8, 66.4, 35.5, 31.5, 28.5, 27.4, 22.0.
HRMS (ESI-TOF): m/z $[M+H]^+$ calcd for $C_{16}H_{25}O$: 233.1905; found: 233.1912.

((allyloxy)methylene)dibenzene (7j):[6]

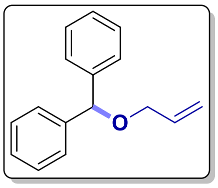

Following the general procedure **D,** the title product was obtained as a colourless oil using silica-gel column chromatography eluting with 1% ethyl acetate in hexane (Yield: 73%, 49 mg). ^{1}H NMR (500 MHz, $CDCl_3$) δ 7.28 – 7.25 (m, 4H), 7.24 – 7.20 (m, 4H), 7.15 (tt, J = 6.4, 1.4 Hz, 2H), 5.88 (ddt, J = 17.2, 10.8, 5.5 Hz, 1H), 5.33 (s, 1H), 5.22 (dq, J = 17.2, 1.7 Hz, 1H), 5.10 (ddd, J = 10.4, 3.0, 1.4 Hz, 1H), 3.92 (dt, J = 5.5, 1.5 Hz, 2H). ^{13}C{^{1}H} NMR (125 MHz, $CDCl_3$) δ 142.2, 134.8, 128.4, 127.5, 127.0, 116.9, 82.6, 69.7.

(E)-((hex-2-en-1-yloxy)methylene)dibenzene (7k):

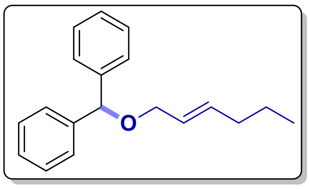

Following the general procedure **D,** the title product was obtained as a colourless oil using silica-gel column chromatography eluting with 1% ethyl acetate in hexane (Yield: 89%, 71 mg). ^{1}H NMR (500 MHz, $CDCl_3$) δ 7.19 – 7.17 (m, 4H), 7.14 (dd, J = 10.3, 4.9 Hz, 4H), 7.08 – 7.04 (m, 2H), 5.55 – 5.40 (m, 2H), 5.25 (s, 1H), 3.79 (dd, J = 5.8, 0.6 Hz, 2H), 1.87 (dd, J = 14.0, 6.8 Hz, 2H), 1.28 – 1.19 (m, 2H), 0.74 (t, J = 7.4 Hz, 3H). ^{13}C{^{1}H} NMR (125 MHz, $CDCl_3$) δ 142.4, 134.6, 128.4, 127.4, 127.1, 126.5, 82.3, 69.6, 34.4, 22.3, 13.7.

HRMS (ESI-TOF): m/z $[M+H]^+$ calcd for $C_{19}H_{23}O$: 267.1749; found: 267.1752.

(E)-((oct-2-en-1-yloxy)methylene)dibenzene (7l):

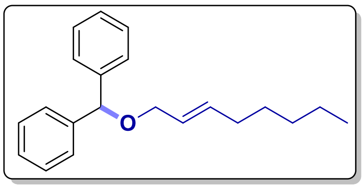

Following the general procedure **D,** the title product was obtained as a colourless oil using silica-gel column chromatography eluting with 1% ethyl acetate in hexane (Yield: 87%, 76.7 mg). ^{1}H NMR (500 MHz, $CDCl_3$) δ 7.44 – 7.42 (m, 4H), 7.38 (t, J = 7.5 Hz, 4H), 7.30 (dd, J = 11.0, 3.8 Hz, 2H), 5.78 – 5.66 (m, 2H), 5.50 (s, 1H), 4.04 (d, J = 5.9 Hz, 2H), 2.12 (q, J = 6.9 Hz, 2H), 1.49 – 1.41 (m, 2H), 1.41

– 1.34 (m, 4H), 0.97 (t, *J* = 6.9 Hz, 3H). $^{13}C\{^{1}H\}$ NMR (125 MHz, $CDCl_3$) δ 142.4, 134.9, 128.4, 127.4, 127.1, 126.3, 82.3, 69.6, 32.3, 31.5, 28.8, 22.6, 14.1.

HRMS (ESI-TOF): m/z $[M+H]^+$ calcd for $C_{21}H_{27}O$: 295.2062; found: 295.2059.

(E)-((non-2-en-1-yloxy)methylene)dibenzene (7m):

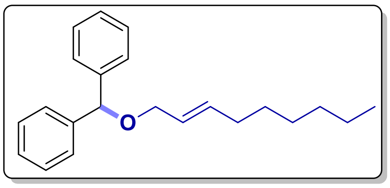

Following the general procedure **D,** the title product was obtained as a colourless oil using silica-gel column chromatography eluting with 1% ethyl acetate in hexane (Yield: 85%, 65 mg). ^{1}H NMR (500 MHz, $CDCl_3$) δ 7.23 (d, *J* = 7.7 Hz, 4H), 7.18 (t, *J* = 7.5 Hz, 4H), 7.11 (t, *J* = 7.2 Hz, 2H), 5.58 – 5.44 (m, 2H), 5.30 (s, 1H), 3.84 (d, *J* = 5.8 Hz, 2H), 1.93 (q, *J* = 6.9 Hz, 2H), 1.28 – 1.23 (m, 2H), 1.21 – 1.15 (m, 6H), 0.77 (t, *J* = 6.6 Hz, 3H). $^{13}C\{^{1}H\}$ NMR (125 MHz, $CDCl_3$) δ 142.4, 134.9, 128.4, 127.4, 127.1, 126.2, 82.3, 69.6, 32.4, 31.8, 29.1, 28.9, 22.7, 14.1.

HRMS (ESI-TOF): m/z $[M+H]^+$ calcd for $C_{22}H_{29}O$: 309.2218; found: 309.2211.

(Z)-(((3,7-dimethylocta-2,6-dien-1-yl)oxy)methylene)dibenzene (7n):

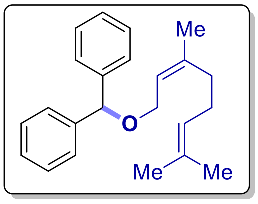

Following the general procedure **D,** the title product was obtained as a colourless oil using silica-gel column chromatography eluting with 1% ethyl acetate in hexane (Yield: 93%, 89 mg). ^{1}H NMR (500 MHz, $CDCl_3$) δ 7.19 (d, *J* = 7.1 Hz, 4H), 7.14 (t, *J* = 7.4 Hz, 4H), 7.07 (dd, *J* = 8.9, 5.3 Hz, 2H), 5.28 (d, *J* = 5.8 Hz, 1H), 5.24 (s, 1H), 4.94 (s, 1H), 3.86 (d, *J* = 6.5 Hz, 2H), 1.98 – 1.91 (m, 2H), 1.88 (d, *J* = 7.4 Hz, 2H), 1.52 (s, 3H), 1.44 (s, 3H), 1.39 (s, 3H). $^{13}C\{^{1}H\}$ NMR (125 MHz, $CDCl_3$) δ 142.5, 140.3, 131.7, 128.4, 127.4, 127.2, 124.1, 121.0, 82.3, 65.4, 39.7, 26.4, 25.7, 17.7, 16.5.

HRMS (ESI-TOF): m/z $[M+H]^+$ calcd for $C_{23}H_{29}O$: 321.2218; found: 321.2213.

((cinnamyloxy)methylene)dibenzene (7o):[7]

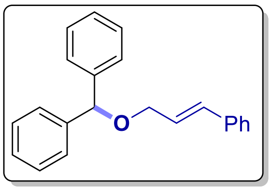

Following the general procedure **D,** the title product was obtained as a colourless oil using silica-gel column chromatography eluting with 1% ethyl acetate in hexane (Yield: 83%, 75 mg). ^{1}H NMR (500 MHz, $CDCl_3$) δ 7.32 – 7.28 (m, 6H), 7.23 (dd, J = 16.1, 8.0 Hz, 6H), 7.19 – 7.14 (m, 3H), 6.51 (d, J = 15.9 Hz, 1H), 6.26 (dt, J = 15.9, 5.9 Hz, 1H), 5.39 (s, 1H), 4.09 (d, J = 5.9 Hz, 2H). ^{13}C{^{1}H} NMR (125 MHz, $CDCl_3$) δ 142.2, 136.8, 132.4, 128.6, 128.5, 127.7, 127.5, 127.1, 126.5, 126.2, 82.7, 69.4.

(1-(cinnamyloxy)cyclohexyl)benzene (7p):[8]

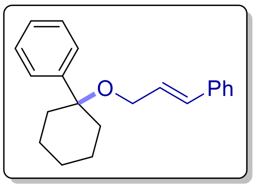

Following the general procedure **D,** the title product was obtained as a colourless oil using silica-gel column chromatography eluting with 3% ethyl acetate in hexane (Yield: 87%, 76 mg). ^{1}H NMR (500 MHz, $CDCl_3$) δ 7.33 – 7.29 (m, 2H), 7.19 (dd, J = 14.1, 7.3 Hz, 4H), 7.14 – 7.08 (m, 3H), 7.04 (t, J = 7.3 Hz, 1H), 6.41 (dd, J = 16.0, 5.9 Hz, 1H), 6.10 (dt, J = 15.9, 5.7 Hz, 1H), 3.58 (dd, J = 5.7, 1.4 Hz, 2H), 1.92 (d, J = 12.8 Hz, 2H), 1.64 (ddd, J = 12.5, 7.9, 3.3 Hz, 2H), 1.60 – 1.54 (m, 3H), 1.45 – 1.40 (m, 2H), 1.16 – 1.08 (m, 1H). ^{13}C{^{1}H} NMR (125 MHz, $CDCl_3$) δ 146.3, 137.2, 130.8, 128.5, 128.3, 127.3, 126.9, 126.4, 126.1, 77.6, 62.7, 35.9, 25.7, 22.1.

(E)-(((4-phenylbut-3-en-2-yl)oxy)methylene)dibenzene (7q):

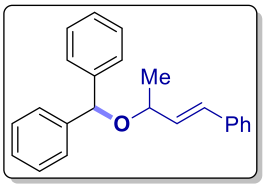

Following the general procedure **D,** the title product was obtained as a colourless oil using silica-gel column chromatography eluting with 3% ethyl acetate in hexane (Yield: 96%, 90 mg). ^{1}H NMR (500 MHz, $CDCl_3$) δ 7.23 – 7.18 (m, 5H), 7.17 – 7.13 (m, 5H), 7.12 – 7.08 (m, 3H), 7.08 – 7.05 (m, 1H), 7.04 – 7.00 (m, 1H), 6.29 (d, J = 16.0 Hz, 1H), 6.01 (dd, J = 15.9, 7.8 Hz, 1H), 5.37 (s, 1H), 3.91 (p, J = 6.6 Hz, 1H), 1.22 (d, J = 6.4 Hz, 3H). ^{13}C{^{1}H} NMR (125 MHz, $CDCl_3$) δ 143.0, 142.3, 136.7, 131.7, 131.6, 128.6, 128.5, 128.3, 127.7, 127.5, 127.4, 127.2, 127.0, 126.6, 115.0, 80.1, 73.9, 21.9.
HRMS (ESI-TOF): m/z $[M+H]^+$ calcd for $C_{23}H_{23}O$: 315.1749; found: 315.1744.

((but-3-en-2-yloxy)methylene)dibenzene (7r):

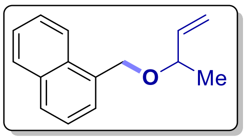

Following the general procedure **D,** the title product was obtained as a colourless oil using silica-gel column chromatography eluting with 3% ethyl acetate in hexane (Yield: 85%, 61 mg). ^{1}H NMR (500 MHz, $CDCl_3$) δ 8.02 (d, J = 8.3 Hz, 1H), 7.78 – 7.70 (m, 2H), 7.45 – 7.38 (m, 3H), 7.35 – 7.32 (dd, J = 8.1, 7.1 Hz, 1H), 5.82 – 5.75 (ddd, J = 17.6, 10.3, 7.5 Hz, 1H), 5.20 – 5.13 (m, 2H), 4.95 (d, J = 11.9 Hz, 1H), 4.72 (d, J = 11.9 Hz, 1H), 3.93 (dq, J = 12.9, 6.4 Hz, 1H), 1.22 (d, J = 6.4 Hz, 3H). ^{13}C{^{1}H} NMR (125 MHz, $CDCl_3$) δ 140.2, 134.2, 133.8, 128.5, 128.4, 126.3, 126.1, 125.7, 125.3, 124.1, 116.4, 115.0, 76.5, 68.5, 21.5.

HRMS (ESI-TOF): m/z $[M+H]^+$ calcd for $C_{17}H_{19}O$: 239.1436; found: 239.1406.

((oct-1-en-3-yloxy)methylene)dibenzene (7s):

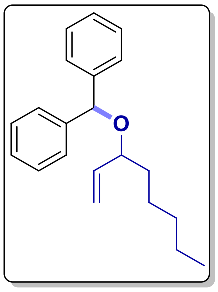

Following the general procedure **D,** the title product was obtained as a colourless oil using silica-gel column chromatography eluting with 3% ethyl acetate in hexane (Yield: 84%, 74 mg). ^{1}H NMR (500 MHz, $CDCl_3$) δ 7.21 – 7.14 (m, 6H), 7.10 (t, J = 7.5 Hz, 3H), 7.02 (t, J = 6.8 Hz, 1H), 5.57 (ddd, J = 10.1, 9.1, 4.5 Hz, 1H), 5.32 (s, 1H), 5.07 – 4.91 (m, 2H), 3.54 (dd, J = 13.8, 6.8 Hz, 1H), 1.62 – 1.45 (m, 1H), 1.37 – 1.31 (m, 1H), 1.28 – 1.18 (m, 1H), 1.15 – 1.08 (m, 3H), 1.07 – 0.99 (m, 2H), 0.72 – 0.68 (m, 3H). ^{13}C{^{1}H} NMR (125 MHz, $CDCl_3$) δ 143.3, 142.3, 139.3, 128.4, 128.2, 127.6, 127.5, 127.0, 126.8, 117.2, 79.7, 78.3, 35.6, 31.8, 24.9, 22.6, 14.0.

HRMS (ESI-TOF): m/z $[M+H]^+$ calcd for $C_{21}H_{27}O$: 295.2062; found: 295.2059.

9-((perfluorophenyl)methoxy)-9H-xanthene (7t):

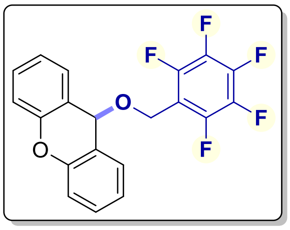

Following the general procedure **D,** the title product was obtained as a colourless oil using silica-gel column chromatography eluting with 5% ethyl acetate in hexane (Yield: 78%, 88 mg). ^{1}H NMR (500 MHz, $CDCl_3$) δ 7.69 (dd, J = 7.7, 1.7 Hz, 1H), 7.41 (dd, J = 8.1, 1.7 Hz, 1H), 7.36 (ddd, J = 8.5, 7.1, 1.7 Hz, 1H), 7.27 – 7.23 (m, 1H), 7.17 – 7.13 (m, 2H), 7.04 (d, J = 1.3 Hz, 2H), 5.85 (s, 1H), 3.93 (s,

2H). $^{13}C\{^{1}H\}$ NMR (125 MHz, $CDCl_3$) δ 153.2, 152.3, 132.5, 131.3, 130.1, 130.0, 129.6, 127.4, 124.0, 123.5, 118.6, 117.2, 116.8, 116.7, 115.7, 115.5, 115.0, 96.9, 70.4, 53.7. ^{19}F NMR (470 MHz, $CDCl_3$) δ -144.3 (q, J = 9.4 Hz), -153.9 (t, J = 23.5 Hz), -161.7 (sextet, J = 9.4 Hz).
HRMS (ESI-TOF): m/z $[M+H]^+$ calcd for $C_{20}H_{11}F_5O_2$: 379.0757; found: 379.0750.

1-((2,2,2-trifluoroethoxy)methyl)naphthalene (7u):

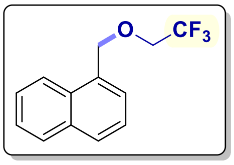

Following the general procedure **D,** the title product was obtained as a colourless oil using silica-gel column chromatography eluting with 3% ethyl acetate in hexane (Yield: 77%, 55 mg). ^{1}H NMR (500 MHz, $CDCl_3$) δ 7.76 – 7.67 (m, 3H), 7.43 – 7.35 (m, 2H), 7.35 – 7.26 (m, 2H), 4.97 (s, 2H), 3.68 (dt, J = 8.7, 5.6 Hz, 2H). $^{13}C\{^{1}H\}$ NMR (125 MHz, $CDCl_3$) δ 133.8, 131.8, 131.7, 130.5, 129.5, 128.6, 127.2, 126.6, 126.1, 125.1, 123.8, 72.7, 66.8 (q, J = 33.65 Hz). ^{19}F NMR (470 MHz, $CDCl_3$) δ -73.6, -73.9.
HRMS (ESI-TOF): m/z $[M+H]^+$ calcd for $C_{13}H_{12}F_3O$: 241.0840; found: 241.0852.

(((1,1,1,3,3,3-hexafluoropropan-2-yl)oxy)methylene)dibenzene (7v):[9]

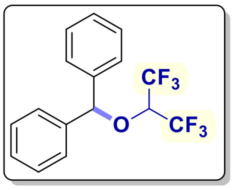

Following the general procedure **D,** the title product was obtained as a colourless oil using silica-gel column chromatography eluting with 3% ethyl acetate in hexane (Yield: 65%, 65 mg). ^{1}H NMR (500 MHz, $CDCl_3$) δ 7.47 – 7.42 (m, 8H), 7.40 – 7.37 (m, 2H), 7.34 – 7.24 (m, 1H), 5.88 (s, 1H). $^{13}C\{^{1}H\}$ NMR (125 MHz, $CDCl_3$) δ 138.9, 128.8, 128.7, 127.7, 126.2–118.4 (m), 85.7, 72.5 (p, J = 31.3 Hz). ^{19}F NMR (470 MHz, $CDCl_3$) δ -72.6 (d, J = 9.4 Hz).

1-((methoxy-d3)methyl)naphthalene (7w):

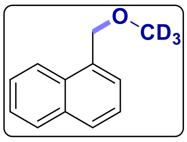

Following the general procedure **D,** the title product was obtained as a colourless oil using silica-gel column chromatography eluting with 1% ethyl acetate in hexane (Yield: 88%, 46 mg). ^{1}H NMR (500 MHz, $CDCl_3$) δ 8.13 – 8.11 (m, 1H), 7.88 (dd, J = 8.6, 1.3 Hz, 1H), 7.83 (d, J = 8.5 Hz, 1H), 7.57 –

7.43 (m, 4H), 4.92 (m, 2H). $^{13}C\{^1H\}$ NMR (125 MHz, $CDCl_3$) δ 133.9, 133.7, 131.8, 128.8, 128.7, 126.6, 126.3, 125.9, 125.3, 124.1, 73.2.

HRMS (ESI-TOF): m/z $[M+Na]^+$ calcd for $C_{12}H_9D_3NaO$: 198.0974; found: 198.0950.

1-((phenylmethoxy-d2)methyl)naphthalene (7x):

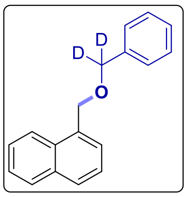

Following the general procedure **D,** the title product was obtained as a colourless oil using silica-gel column chromatography eluting with 1% ethyl acetate in hexane (Yield: 84%, 63 mg). ^1H NMR (500 MHz, $CDCl_3$) δ 7.97 – 7.93 (m, 1H), 7.89 – 7.82 (m, 3H), 7.72 – 7.67 (m, 1H), 7.52 – 7.41 (m, 6H), 7.29 (s, 1H), 5.57 (s, 1H). $^{13}C\{^1H\}$ NMR (125 MHz, $CDCl_3$) δ 129.4, 128.8, 128.7, 128.6, 128.3, 128.2, 128.1, 127.6, 126.6, 126.4, 125.9, 125.8, 125.6, 125.3, 123.9, 123.7, 77.3, 65.3.

HRMS (ESI-TOF): m/z $[M+Na]^+$ calcd for $C_{18}H_{14}D_2NaO$: 273.1224; found: 273.1219.

((1-phenylethoxy-2,2,2-d3)methylene)dibenzene (7y):

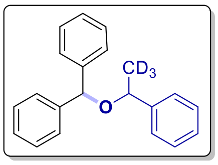

Following the general procedure **D,** the title product was obtained as a colourless oil using silica-gel column chromatography eluting with 1% ethyl acetate in hexane (Yield: 70%, 61 mg). ^1H NMR (500 MHz, $CDCl_3$) δ 7.36 (dd, J = 5.1, 2.3 Hz, 5H), 7.34 – 7.33 (m, 2H), 7.32 – 7.29 (m, 3H), 7.27 – 7.25 (m, 5H), 5.26 (s, 1H), 4.45 (s, 1H). $^{13}C\{^1H\}$ NMR (125 MHz, $CDCl_3$) δ 143.8, 142.9, 142.1, 128.6, 128.5, 128.3, 127.7, 127.6, 127.2, 127.0, 126.6, 80.1, 74.9, 14.2.

HRMS (ESI-TOF): m/z $[M+H]^+$ calcd for $C_{21}H_{18}D_3O$: 292.1781; found: 292.1772.

5-((benzhydryloxy)methyl)-2,2,7,7-tetramethyltetrahydro-5H-bis([1,3]dioxolo)[4,5-b:4',5'-d]pyran (7z):

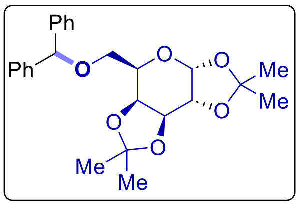

Following the general procedure **D,** the title product was obtained as a colourless oil using silica-gel column chromatography eluting with 7% ethyl acetate in hexane (Yield: 59%, 75 mg). ^{1}H NMR (500 MHz, $CDCl_3$) δ 7.37 (d, J = 0.7 Hz, 4H), 7.33 (s, 3H), 7.32 (s, 1H), 7.28 (d, J = 1.5 Hz, 1H), 7.26 (s, 1H), 5.52 (d, J = 5.0 Hz, 1H), 5.47 (s, 1H), 4.60 (dd, J = 7.9, 2.4 Hz, 1H), 4.34 – 4.31 (m, 1H), 4.30 – 4.29 (m, 1H), 4.13 – 4.06 (m, 1H), 3.65 (dd, J = 16.4, 6.4 Hz, 2H), 1.55 (s, 3H), 1.41 (s, 3H), 1.33 (s, 6H). 13C{1H} NMR (125 MHz, $CDCl_3$) δ 143.9, 142.2, 128.6, 127.7, 126.6, 109.3, 108.7, 96.4, 83.9, 76.3, 71.2, 70.8, 67.7, 67.1, 26.2, 26.1, 25.1, 24.5.
HRMS (ESI-TOF): m/z $[M+H]^+$ calcd for $C_{25}H_{30}O_6$: 426.2042; found: 426.2034.

1-isobutyl-4-[1-(1-phenylethoxy)ethyl]benzene (7za):[10]

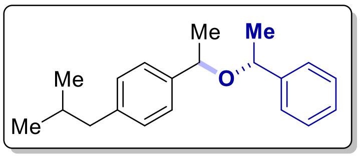

Following the general procedure **E,** the title product was obtained as a colourless oil using silica-gel column chromatography eluting with 5% ethyl acetate in hexane (Yield: 95%, 76 mg). **Diasteromer 1: Diastereomer 2: 50:50.** ^{1}H NMR (500 MHz, $CDCl_3$, for both diastereomers) (the integration at 1.92 ppm, 1.88 ppm indicated the ratio of the two isomers of **7za** to be 50:50) δ 7.39 (dd, J = 9.5, 5.3 Hz, 2H), 7.36 – 7.28 (m, 7H), 7.24 (ddd, J = 10.9, 8.3, 3.6 Hz, 5H), 7.17 (d, J = 8.0 Hz, 2H), 7.10 (d, J = 8.0 Hz, 2H), 4.56 (dq, J = 12.7, 6.4 Hz, 2H), 4.28 (dq, J = 16.2, 6.5 Hz, 2H), 2.53 (d, J = 7.2 Hz, 2H), 2.48 (d, J = 7.2 Hz, 2H), 1.93 (dd, J = 13.6, 6.8 Hz, 1H), 1.90 – 1.84 (m, 1H), 1.50 (d, J = 6.4 Hz, 6H), 1.42 (dd, J = 6.5, 1.1 Hz, 6H), 0.97 (d, J = 6.6 Hz, 6H), 0.94 (d, J = 6.6 Hz, 6H). ^{13}C{^{1}H} NMR (125 MHz, $CDCl_3$, for both diastereomers) δ 144.8, 144.7, 141.8, 141.7, 141.2, 140.9, 129.6, 129.4, 128.8, 128.6, 127.7, 127.5, 126.8, 126.7, 126.5, 126.4, 74.9, 74.8, 45.6, 45.5, 30.7, 30.6, 25.2, 25.1, 23.5, 23.3, 22.9, 22.88, 22.83.

1-(3-(benzhydryloxy)-10,13-dimethyl-2,3,4,7,8,9,10,11,12,13,14,15,16,17-tetradecahydro-1H-cyclopenta[a]phenanthren-17-yl)ethan-1-one (7zb):

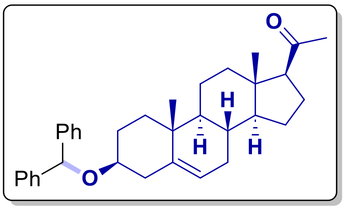

Following the general procedure **E,** the title product was obtained as a colourless oil using silica-gel column chromatography eluting with 3% ethyl acetate in hexane (Yield: 82%, 118 mg). ^{1}H NMR (500 MHz, $CDCl_3$) δ 7.27 – 7.22 (m, 6H), 7.22 – 7.18 (dd, J = 9.8, 4.5 Hz, 2H), 7.15 (dt, J = 4.1, 1.8 Hz,

2H), 5.47 (s, 1H), 5.21 – 5.20 (m, 1H), 3.23 – 3.17 (m, 1H), 2.42 (t, *J* = 8.9 Hz, 1H), 2.35 – 2.24 (m, 2H), 2.02 (s, 3H), 1.95 – 1.85 (m, 3H), 1.74 (dt, *J* = 13.3, 3.4 Hz, 1H), 1.58 – 1.50 (m, 3H), 1.45 – 1.32 (m, 5H), 1.16 (s, 2H), 1.05 – 0.99 (m, 1H), 0.92 (s, 3H), 0.86 – 0.78 (m, 2H), 0.53 (s, 3H). $^{13}C\{^1H\}$ NMR (125 MHz, $CDCl_3$) δ 209.5, 142.9, 141.0, 139.9, 138.4, 128.8, 128.5, 128.4, 128.35, 128.34, 127.9, 127.3, 127.2, 127.1, 121.2, 115.0, 80.4, 77.6, 63.7, 57.3, 56.9, 50.0, 44.0, 39.3, 38.9, 37.2, 36.9, 31.9, 31.8, 31.5, 28.6, 24.5, 22.8, 21.0, 19.4, 13.2.

HRMS (ESI-TOF): m/z $[M+H]^+$ calcd for $C_{34}H_{43}O_2$: 483.3263; found: 483.3259.

3-(benzhydryloxy)-10,13-dimethylhexadecahydro-17H-cyclopenta[a]phenanthren-17-one (7zc):

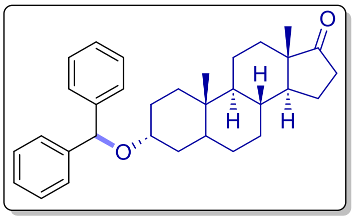

Following the general procedure **E,** the title product was obtained as a white solid using silica-gel column chromatography eluting with 3% ethyl acetate in hexane (Yield: 76%, 103 mg). ^1H NMR (500 MHz, $CDCl_3$) *δ* 7.37 – 7.32 (m, 6H), 7.31 – 7.27 (m, 2H), 7.24 – 7.19 (m, 2H), 5.56 (s, 1H), 3.32 (ddd, *J* = 16.5, 10.8, 4.9 Hz, 1H), 2.44 – 2.38 (m, 1H), 2.08 – 2.00 (m, 1H), 1.94 – 1.87 (m, 2H), 1.77 – 1.74 (m, 2H), 1.72 – 1.67 (m, 1H), 1.63 – 1.60 (m, 1H), 1.56 – 1.40 (m, 4H), 1.32 – 1.17 (m, 5H), 1.04 – 0.85 (m, 3H), 0.84 (s, 6H). $^{13}C\{^1H\}$ NMR (125 MHz, $CDCl_3$) *δ* 221.6, 143.0, 143.0, 128.6, 128.4, 127.7, 127.4, 127.2, 127.2, 126.6, 80.3, 76.2, 54.5, 51.5, 47.9, 44.9, 37.1, 36.0, 35.1, 31.6, 30.9, 28.6, 21.9, 20.6, 13.9, 12.4.
HRMS (ESI-TOF): m/z $[M+H]^+$ calcd for $C_{32}H_{41}O_2$: 457.3107; found: 457.3102.

3-(benzhydryloxy)-10,13-dimethyl-17-(6-methylheptan-2-yl)-2,3,4,7,8,9,10,11,12,13,14,15,16,17-tetradecahydro-1H-cyclopenta[a]phenanthrene (7zd):[11]

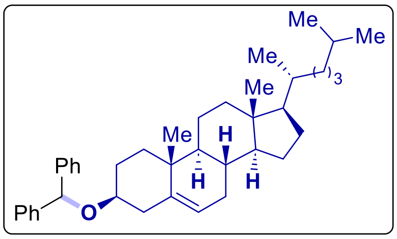

Following the general procedure **E**, the title product was obtained as a white solid using silica-gel column chromatography eluting with 3% ethyl acetate in hexane (Yield: 75%, 110 mg). ^1H NMR (500 MHz, $CDCl_3$) *δ* 7.22 (d, *J* = 8.1 Hz, 4H), 7.18 (t, *J* = 7.5 Hz, 4H), 7.10 (t, *J* = 7.1 Hz, 2H), 5.44 (s, 1H), 5.17 (d, *J* = 5.0 Hz, 1H), 3.23 – 3.08 (m, 1H), 2.31 – 2.21 (m, 2H), 1.84 (dd, *J* = 23.3, 12.4 Hz, 3H),

1.76 – 1.63 (m, 3H), 1.44 (s, 3H), 1.39 – 1.31 (m, 5H), 1.22 (dd, J = 20.1, 16.2 Hz, 6H), 1.04 – 0.96 (m, 6H), 0.88 (s, 3H), 0.78 (d, J = 6.5 Hz, 3H), 0.74 (dd, J = 6.6, 2.2 Hz, 6H), 0.54 (s, 3H). $^{13}C\{^1H\}$ NMR (125 MHz, $CDCl_3$) δ 143.0, 141.1, 128.4, 127.4, 127.2, 121.6, 80.4, 75.7, 56.9, 56.2, 50.2, 42.4, 39.6, 36.3, 35.9, 31.9, 29.8, 28.7, 28.3, 28.1, 24.4, 23.9, 22.9, 22.7, 21.1, 19.5, 18.8, 11.9.

1-(5-(benzhydryloxy)hexyl)-3,7-dimethyl-3,7-dihydro-1H-purine-2,6-dione (7ze):

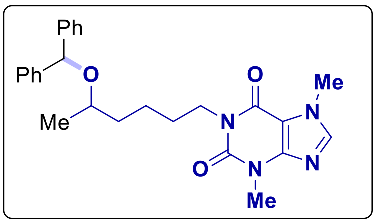

Following the general procedure **E,** the title product was obtained as a colourless oil using silica-gel column chromatography eluting with 15% ethyl acetate in hexane (Yield: 69%, 92 mg). ^1H NMR (500 MHz, $CDCl_3$) δ 7.44 (t, J = 3.1 Hz, 1H), 7.37 – 7.27 (m, 4H), 7.27 – 7.20 (m, 4H), 7.18 – 7.11 (m, 2H), 5.43 (s, 1H), 3.98 – 3.92 (m, 2H), 3.91 (s, 3H), 3.52 (s, 3H), 3.49 – 3.43 (m, 1H), 1.83 – 1.53 (m, 3H), 1.50 – 1.20 (m, 3H), 1.12 (d, J = 6.2 Hz, 3H). $^{13}C\{^1H\}$ NMR (125 MHz, $CDCl_3$) δ 155.4, 151.5, 148.8, 143.4, 142.8, 141.4, 128.4, 128.3, 127.39, 127.36, 127.0, 80.5, 80.4, 72.6, 67.9, 41.4, 36.5, 33.7, 29.8, 28.2, 23.1, 19.7.

HRMS (ESI-TOF): m/z $[M+H]^+$ calcd for $C_{26}H_{31}N_4O_3$: 447.2396; found: 447.2388.

4-((benzhydryloxy)methyl)phenyl adamantane-1-carboxylate (7zf):

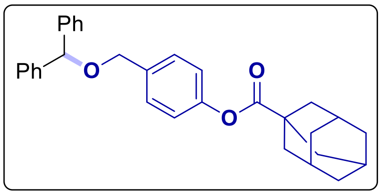

Following the general procedure **E,** the title product was obtained as a colourless oil using silica-gel column chromatography eluting with 3% ethyl acetate in hexane (Yield: 69%, 84 mg). ^1H NMR (500 MHz, $CDCl_3$) δ 7.43 – 7.36 (m, 5H), 7.33 (t, J = 7.7 Hz, 2H), 7.28 – 7.18 (m, 5H), 7.03 (dd, J = 8.8, 0.7 Hz, 2H), 5.44 (s, 1H), 4.53 (s, 2H), 2.14 – 2.07 (m, 3H), 2.06 (d, J = 3.3 Hz, 6H), 1.77 (d, J = 3.0 Hz, 6H). $^{13}C\{^1H\}$ NMR (125 MHz, $CDCl_3$) δ 176.4, 171.5, 150.6, 142.1, 139.9, 138.5, 135.8, 135.7, 128.9, 128.8, 128.7, 128.54, 128.51, 127.9, 127.6, 127.4, 127.2, 127.1, 121.6, 82.5, 69.9, 41.1, 38.8, 36.59, 36.55, 28.0, 27.9.

HRMS (ESI-TOF): m/z $[M+Na]^+$ calcd for $C_{31}H_{32}NaO_3$: 475.2249; found: 475.2220.

4-((benzhydryloxy)methyl)phenyl tetradecanoate (7zg):

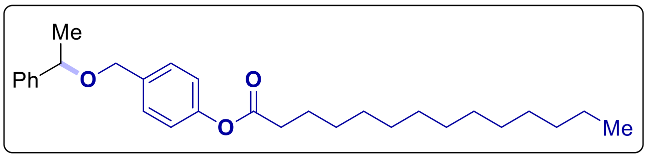

Following the general procedure **E,** the title product was obtained as a colourless oil using silica-gel column chromatography eluting with 3% ethyl acetate in hexane (Yield: 67%, 100 mg). ^{1}H NMR (500 MHz, $CDCl_3$) δ 7.24 – 7.17 (m, 5H), 7.17 – 7.11 (m, 2H), 6.92 – 6.84 (m, 2H), 4.37 – 4.30 (m, 1H), 4.28 – 4.11 (m, 2H), 2.42 – 2.35 (m, 2H), 1.63 – 1.53 (m, 2H), 1.32 (d, J = 6.5 Hz, 3H), 1.16 – 1.05 (m, 20H), 0.72 (t, J = 7.1 Hz, 3H). ^{13}C{^{1}H} NMR (125 MHz, $CDCl_3$) δ 172.5, 150.1, 143.7, 136.2, 128.8, 128.6, 127.6, 126.4, 121.6, 77.3, 69.7, 34.5, 32.0, 29.8, 29.7, 29.6, 29.5, 29.4, 29.3, 29.2, 25.0, 24.3, 22.8, 14.2.

HRMS (ESI-TOF): m/z $[M+H]^+$ calcd for $C_{34}H_{45}O_3$: 501.3369; found: 501.3358.

1-(methoxymethyl)naphthalene (8a):[12]

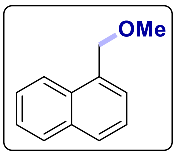

Following the general procedure **F,** the title product was obtained as a colourless oil using silica-gel column chromatography eluting with 1% ethyl acetate in hexane (Yield: 96%, 50 mg). ^{1}H NMR (500 MHz, $CDCl_3$) δ 7.93 (d, J = 8.3 Hz, 1H), 7.66 (dd, J = 25.5, 7.9 Hz, 2H), 7.40 – 7.30 (m, 3H), 7.28 – 7.23 (m, 1H), 4.73 (s, 2H), 3.28 (s, 3H). ^{13}C{^{1}H} NMR (125 MHz, $CDCl_3$) δ 133.8, 133.7, 131.8, 128.7, 128.6, 126.5, 126.3, 125.8, 125.2, 124.0, 73.2, 58.2.

(methoxymethyl)benzene (8b):

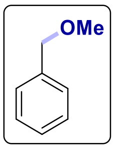

Following the general procedure **F,** the title product was obtained as a colourless oil using silica-gel column chromatography eluting with 5% ethyl acetate in hexane (Yield: 85%, 32 mg). ^{1}H NMR (500 MHz, $CDCl_3$) δ 7.38 – 7.26 (m, 5H), 4.46 (s, 2H), 3.39 (s, 3H). ^{13}C{^{1}H} NMR (125 MHz, $CDCl_3$) δ 138.3, 128.5, 127.8, 127.7, 74.8, 58.2.

HRMS (ESI-TOF): m/z $[M+H]^+$ calcd for $C_8H_{11}O$: 123.0810; found: 123.0819.

1-methoxy-4-(methoxymethyl)benzene (8c):[13]

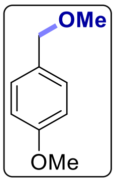

Following the general procedure **F,** the title product was obtained as a colourless oil using silica-gel column chromatography eluting with 5% ethyl acetate in hexane (Yield: 92%, 42 mg). ^{1}H NMR (500 MHz, $CDCl_3$) δ 7.25 (dd, *J* = 5.0, 4.4 Hz, 2H), 6.88 (d, *J* = 8.9 Hz, 2H), 4.38 (s, 2H), 3.80 (s, 3H), 3.35 (s, 3H).

1-ethyl-4-(methoxymethoxy)benzene (8d):[14]

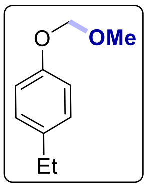

Following the general procedure **F,** the title product was obtained as a colourless oil using silica-gel column chromatography eluting with 1% ethyl acetate in hexane (Yield: 92%, 46 mg). ^{1}H NMR (500 MHz, $CDCl_3$) *δ* 6.96 – 6.95 (m, 2H), 6.81 – 6.79 (m, 2H), 4.99 (s, 2H), 3.32 (s, 3H), 2.46 – 2.41 (m, 2H), 1.05 (t, *J* = 7.6 Hz, 3H). ^{13}C NMR (125 MHz, $CDCl_3$) *δ* 155.3, 137.8, 128.8, 116.2, 94.7, 55.9, 28.0, 15.8.

1-(methoxymethyl)adamantine (8e):

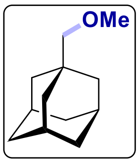

Following the general procedure **F,** the title product was obtained as a colourless oil using silica-gel column chromatography eluting with 1% ethyl acetate in hexane (Yield: 88%, 47 mg). ^{1}H NMR (500 MHz, $CDCl_3$) δ 3.18 (s, 2H), 3.14 (s, 3H), 1.85 – 1.75 (m, 1H), 1.67 – 1.60 (m, 1H), 1.57 (d, *J* = 7.1 Hz, 2H), 1.41 (d, *J* = 12.1 Hz, 1H), 1.36 (dd, *J* = 14.5, 7.3 Hz, 4H), 0.93 (t, *J* = 7.3 Hz, 6H). ^{13}C{^{1}H} NMR (125 MHz, $CDCl_3$) δ 76.9, 58.8, 48.4, 43.2, 37.7, 36.1, 35.9, 31.2, 29.8, 27.6, 23.9, 19.8, 13.7. HRMS (ESI-TOF): m/z $[M+H]^+$ calcd for $C_{12}H_{21}O$: 181.1592; found: 181.1586.

(6Z,9Z)-17-methoxyheptadeca-6,9-diene (8f):

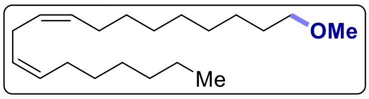

Following the general procedure **F,** the title product was obtained as a colourless oil using silica-gel column chromatography eluting with 1% ethyl acetate in hexane (Yield: 65%, 52 mg). ^{1}H NMR (500 MHz, $CDCl_3$) δ 5.41 – 5.35 (m, 4H), 3.34 (s, 3H), 3.33 – 3.07 (m, 2H), 2.80 (t, J = 6.6 Hz, 2H), 2.08 (dd, J = 6.8, 3.4 Hz, 4H), 1.60 – 1.45 (m, 3H), 1.40 – 1.30 (m, 15H), 1.14 (d, J = 6.1 Hz, 2H), 0.91 (s, 3H).^{13}C{^{1}H} NMR (125 MHz, $CDCl_3$) δ 130.3, 130.1, 128.1, 127.9, 82.1, 55.9, 36.4, 32.9, 31.6, 29.7, 29.5, 29.4, 27.3, 25.7, 25.4, 22.6, 19.1, 14.1.

HRMS (ESI-TOF): m/z $[M+H]^+$ calcd for $C_{20}H_{38}O$: 295.3001 ; found: 295.3022.

(*E*)-1-methoxyheptadec-8-ene (8g):

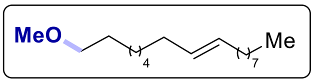

Following the general procedure **F**, the title product was obtained as a colourless oil using silica-gel column chromatography eluting with 1% ethyl acetate in hexane (Yield: 72%, 57 mg). ^{1}H NMR (500 MHz, $CDCl_3$) δ 5.39 – 5.37 (m, 2H), 3.31 (s, 3H), 3.30 – 3.22 (m, 2H), 1.96 (s, 4H), 1.38 – 1.27 (m, 22H), 0.88 (d, J = 2.0 Hz, 3H). ^{13}C NMR (125 MHz, $CDCl_3$) δ 130.5, 130.2, 82.1, 55.9, 36.3, 32.6, 31.9, 29.7, 29.5, 29.3, 29.2, 25.8, 25.3, 24.8, 22.7, 19.0, 14.1.

HRMS (ESI-TOF): m/z $[M+H]^+$ calcd for $C_{18}H_{37}O$: 269.2844 ; found: 269.2840.

(Z)-1-methoxyheptadec-8-ene (8h):

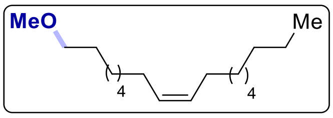

Following the general procedure **F**, the title product was obtained as a colourless oil using silica-gel column chromatography eluting with 1% ethyl acetate in hexane (Yield: 55%, 44 mg). ^{1}H NMR (500 MHz, $CDCl_3$) δ 5.35 – 5.33 (m, 2H), 3.35 (t, J = 6.7 Hz, 1H), 3.31 (s, 3H), 2.01 (ddd, J = 17.7, 10.3, 5.9 Hz, 4H), 1.47 (dddd, J = 12.4, 11.2, 5.7, 1.7 Hz, 4H), 1.35 – 1.09 (m, 19H), 0.87 (dd, J = 7.3, 4.3 Hz, 3H). ^{13}C{^{1}H} NMR (125 MHz, $CDCl_3$) δ 130.1, 129.7, 82.1, 56.5, 32.9, 31.9, 30.1, 29.9, 29.6, 29.41, 29.40, 29.3, 27.3, 25.9, 25.1, 22.8, 14.2, 9.4.

HRMS (ESI-TOF): m/z $[M+H]^+$ calcd for $C_{18}H_{37}O$: 269.2844; found: 269.2871.

7-(methoxymethoxy)-2H-chromen-2-one (8i):[15]

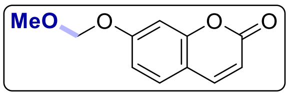

Following the general procedure **F**, the title product was obtained as a colourless oil using silica-gel column chromatography eluting with 1% ethyl acetate in hexane (Yield: 79%, 49 mg). ^{1}H NMR (500 MHz, $CDCl_3$) δ 7.63 (dd, J = 9.6, 0.9 Hz, 1H), 7.37 (d, J = 8.6 Hz, 1H), 6.97 (ddd, J = 11.0, 5.5, 1.6 Hz, 2H), 6.26 (t, J = 5.4 Hz, 1H), 5.22 (s, 2H), 3.48 (s, 3H). ^{13}C{^{1}H} NMR (125 MHz, $CDCl_3$) δ 160.0, 159.2, 154.5, 142.2, 127.7, 112.7, 112.4, 102.8, 93.3, 55.3.
HRMS (ESI-TOF): m/z [M+H]$^+$ calcd for $C_{11}H_{11}O_4$: 207.0657 ; found: 207.0651.

(methoxymethylene)dibenzene (8j):[16]

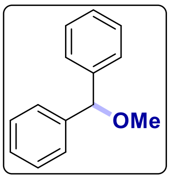

Following the general procedure **F,** the title product was obtained as a colourless oil using silica-gel column chromatography eluting with 1% ethyl acetate in hexane (Yield: 90%, 53 mg). ^{1}H NMR (500 MHz, $CDCl_3$) δ 7.44 – 7.38 (m, 8H), 7.33 – 7.29 (m, 2H), 5.32 (s, 1H), 3.46 (s, 3H). ^{13}C{^{1}H} NMR (125 MHz, $CDCl_3$) δ 142.2, 128.5, 127.5, 126.9, 85.5, 57.1.

9-methoxy-9H-xanthene (8k):

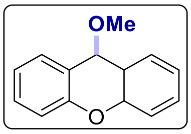

Following the general procedure **F,** the title product was obtained as a colourless oil using silica-gel column chromatography eluting with 3% ethyl acetate in hexane (Yield: 94%, 60 mg). ^{1}H NMR (500 MHz, $CDCl_3$) δ 7.37 – 7.35 (m, 2H), 7.23 – 7.19 (m, 2H), 7.04 – 6.99 (m, 4H), 5.57 (s, 1H), 2.80 (s, 3H). ^{13}C{^{1}H} NMR (125 MHz, $CDCl_3$) δ 152.6, 130.1, 129.7, 123.3, 119.7, 116.7, 70.9, 51.6.
HRMS (ESI-TOF): m/z [M+Na]$^+$ calcd for $C_{14}H_{14}NaO_2$: 237.0891 ; found: 237.0886.

2-methoxy-6-(1-methoxyethyl)naphthalene (8l):[17]

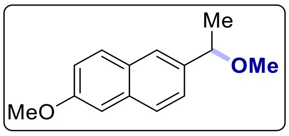

Following the general procedure **F,** the title product was obtained as a colourless oil using silica-gel column chromatography eluting with 7% ethyl acetate in hexane (Yield: 95%, 62 mg). ^{1}H NMR (500

MHz, $CDCl_3$) δ 7.59 – 7.55 (m, 2H), 7.50 (d, *J* = 1.8 Hz, 1H), 7.27 (dd, *J* = 8.4, 1.8 Hz, 1H), 7.04 – 6.99 (m, 1H), 6.98 (s, 1H), 4.27 (q, *J* = 6.5 Hz, 1H), 3.76 (s, 3H), 3.08 (s, 3H), 1.35 - 1.34 (d, *J* = 6.5 Hz, 3H). $^{13}C\{^1H\}$ NMR (125 MHz, $CDCl_3$) δ 157.6, 138.6, 134.2, 129.3, 128.7, 127.2, 125.1, 124.7, 118.9, 105.8, 79.7, 56.4, 55.3, 23.8.

1-isobutyl-4-(1-methoxyethyl)benzene (8m):[17]

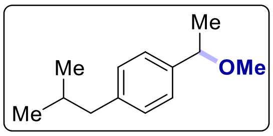

Following the general procedure **F,** the title product was obtained as a colourless oil using silica-gel column chromatography eluting with 3% ethyl acetate in hexane (Yield: 92%, 53 mg). ^1H NMR (500 MHz, $CDCl_3$) δ 7.25 (d, *J* = 8.0 Hz, 2H), 7.16 (d, *J* = 8.0 Hz, 2H), 4.30 (q, *J* = 6.5 Hz, 1H), 3.25 (s, 3H), 2.50 (d, *J* = 7.2 Hz, 2H), 1.89 (dt, *J* = 13.5, 6.8 Hz, 1H), 1.47 (d, *J* = 6.5 Hz, 3H), 0.94 (d, *J* = 6.6 Hz, 6H). $^{13}C\{^1H\}$ NMR (125 MHz, $CDCl_3$) δ 140.9, 140.7, 129.2, 126.0, 79.5, 56.4, 45.2, 30.3, 23.8, 22.4.

(3s,5s,7s)-1-methoxyadamantane (8n):[18]

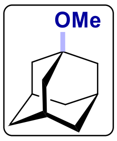

Following the general procedure **F,** the title product was obtained as a colourless oil using silica-gel column chromatography eluting with 1% ethyl acetate in hexane (Yield: 93%, 46 mg). ^1H NMR (500 MHz, $CDCl_3$) δ 3.26 (s, 3H), 2.19 – 2.16 (m, 3H), 1.76 (d, *J* = 2.9 Hz, 6H), 1.68 – 1.61 (m, 6H). $^{13}C\{^1H\}$ NMR (125 MHz, $CDCl_3$) δ 71.9, 47.8, 40.9, 36.5, 30.5.

(1-methoxycyclohexyl)benzene (8o):[19]

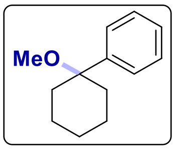

Following the general procedure **F,** the title product was obtained as a colourless oil using silica-gel column chromatography eluting with 3% ethyl acetate in hexane (Yield: 89%, 51 mg). ^1H NMR (500 MHz, $CDCl_3$) δ 7.25 (dt, *J* = 8.2, 1.6 Hz, 2H), 7.20 – 7.16 (m, 2H), 7.10 – 7.07 (m, 1H), 2.80 (s, 3H), 1.87 – 1.84 (m, 2H), 1.59 – 1.49 (m, 5H), 1.44 – 1.40 (m, 2H), 1.13 – 1.08 (ddd, *J* = 11.4, 6.1, 2.2 Hz, 1H). $^{13}C\{^1H\}$ NMR (125 MHz, $CDCl_3$) δ 146.0, 128.2, 126.8, 126.1, 77.4, 49.6, 35.4, 25.7, 21.9.

(2-methoxypropan-2-yl)benzene (8p):[17]

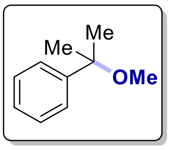

Following the general procedure **F,** the title product was obtained as a colourless oil using silica-gel column chromatography eluting with 3% ethyl acetate in hexane (Yield: 94%, 42 mg). ^{1}H NMR (500 MHz, $CDCl_3$) *δ* 7.37 – 7.21 (m, 3H), 7.01 – 6.93 (m, 2H), 3.83 (s, 3H), 1.63 (s, 6H). ^{13}C{^{1}H} NMR (125 MHz, $CDCl_3$) δ 143.9, 129.7, 128.6, 127.1, 125.9, 114.0, 55.2, 26.3.

(2-(methoxy-d3)propan-2-yl)benzene (8q):[20]

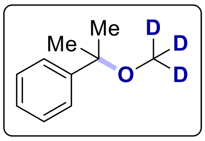

Following the general procedure **F,** the title product was obtained as a colourless oil using silica-gel column chromatography eluting with 3% ethyl acetate in hexane (Yield: 59%, 27 mg). ^{1}H NMR (500 MHz, $CDCl_3$) δ 7.40 – 7.29 (m, 3H), 7.29 – 7.22 (m, 2H), 1.44 (s, 6H).

6. References

1. W. K. Xu, J. M. Guo, C. H. Liu, J. T. Sun, M. Lv, B. G. Wei, *Org. Biomol. Chem.* **2022**, *20*, 5086–5094.
2. P. Xu, W. W. Liu, T. Z. Hao, Y. Q. Liu, H. X. Jiang, J. Xu, J. Y. Li, L. Yin, S. L. Zhu, X. Zhu, *J. Org. Chem.* **2024**, DOI 10.1021/acs.joc.3c02887.
3. X. Zhao, X. Zhu, K. Wang, J. Lv, S. Chen, G. Yao, J. Lang, F. Lv, Y. Pu, R. Yang, B. Zhang, Z. Jiang, Y. Wan, *Nat. Commun.* **2022**, *13*, 1–11.
4. J. H. Youn, S. Y. Go, H. Chung, H. Lee, T. D. Chung, P. H. Y. Cheong, H. G. Lee, *Org. Lett.* **2024**, *26*, 198–203.
5. K. T. Howard, B. C. Duffy, M. R. Linaburg, J. D. Chisholm, *Org. Biomol. Chem.* **2016**, *14*, 1623–1628.
6. A. G. Singh, A. A. Alawaed, P. V. Ramachandran, *RSC Adv.* **2024**, *14*, 24236–24239.
7. C. Empel, C. Pei, F. He, S. Jana, R. M. Koenigs, *Chem. - A Eur. J.* **2022**, *28*, 1–7.
8. P. Das, M. DeSpain, A. Ethridge, J. D. Weaver, *Org. Lett.* **2023**, *25*, 7316–7321.
9. S. Guo, Y. Li, Q. H. Li, K. Zheng, *Chem. Commun.* **2024**, *60*, 2501–2504.

10. J. Xiang, M. Shang, Y. Kawamata, H. Lundberg, S. H. Reisberg, M. Chen, P. Mykhailiuk, G. Beutner, M. R. Collins, A. Davies, M. Del Bel, G. M. Gallego, J. E. Spangler, J. Starr, S. Yang, D. G. Blackmond, P. S. Baran, *Nature* **2019**, *573*, 398–402.
11. S. Y. Go, H. Chung, S. J. Shin, S. An, J. H. Youn, T. Y. Im, J. Y. Kim, T. D. Chung, H. G. Lee, *J. Am. Chem. Soc.* **2022**, *144*, 9149–9160.
12. G. A. Molander, F. Beaumard, T. K. Niethamer, *J. Org. Chem.* **2011**, *76*, 8126–8130.
13. F. G. Delolo, J. Fessler, H. Neumann, K. Junge, E. N. dos Santos, E. V. Gusevskaya, M. Beller, *Chem. - A Eur. J.* **2022**, *28*, 1–5.
14. Y. Zhang, P. K. Sahoo, P. Ren, Y. Qin, R. Cauwenbergh, P. Nimmegeers, G. SivaRaman, S. Van Passel, A. Guidetti, S. Das, *Chem. Commun.* **2022**, *58*, 11454–11457.
15. M. Asada, T. Obitsu, A. Kinoshita, T. Nagase, T. Yoshida, Y. Yamaura, H. Takizawa, K. Yoshikawa, K. Sato, M. Narita, H. Nakai, M. Toda, Y. Tobe, *Bioorg. Med. Chem.* **2010**, *18*, 3212–3223.
16. Y. Morii, T. Watanabe, Y. Saga, T. Kambe, M. Kondo, S. Masaoka, *ChemElectroChem* **2024**, *11*, 1–7.
17. Q. Y. Li, S. N. Gockel, G. A. Lutovsky, K. S. Deglopper, N. J. Baldwin, M. W. Bundesmann, J. W. Tucker, S. W. Bagley, T. P. Yoon, *Nat. Chem.* **2022**, *14*, 94–99.
18. M. Bonsir, C. Davila, A. R. Kennedy, Y. Geerts, *Eur. J. Org. Chem*. **2021**, 5227–5237.
19. C. Yin, W. Liu, Q. Wu, M. Wang, P. Hu, *Adv. Synth. Catal*. **2023**, *365*, 1072– 1081.
20. T. Mitsuhashi, *J. Am. Chem. Soc.* **1986**, 108, 2394-2400.

7. ^{1}H, ^{13}C, ^{19}F NMR Spectra of compounds

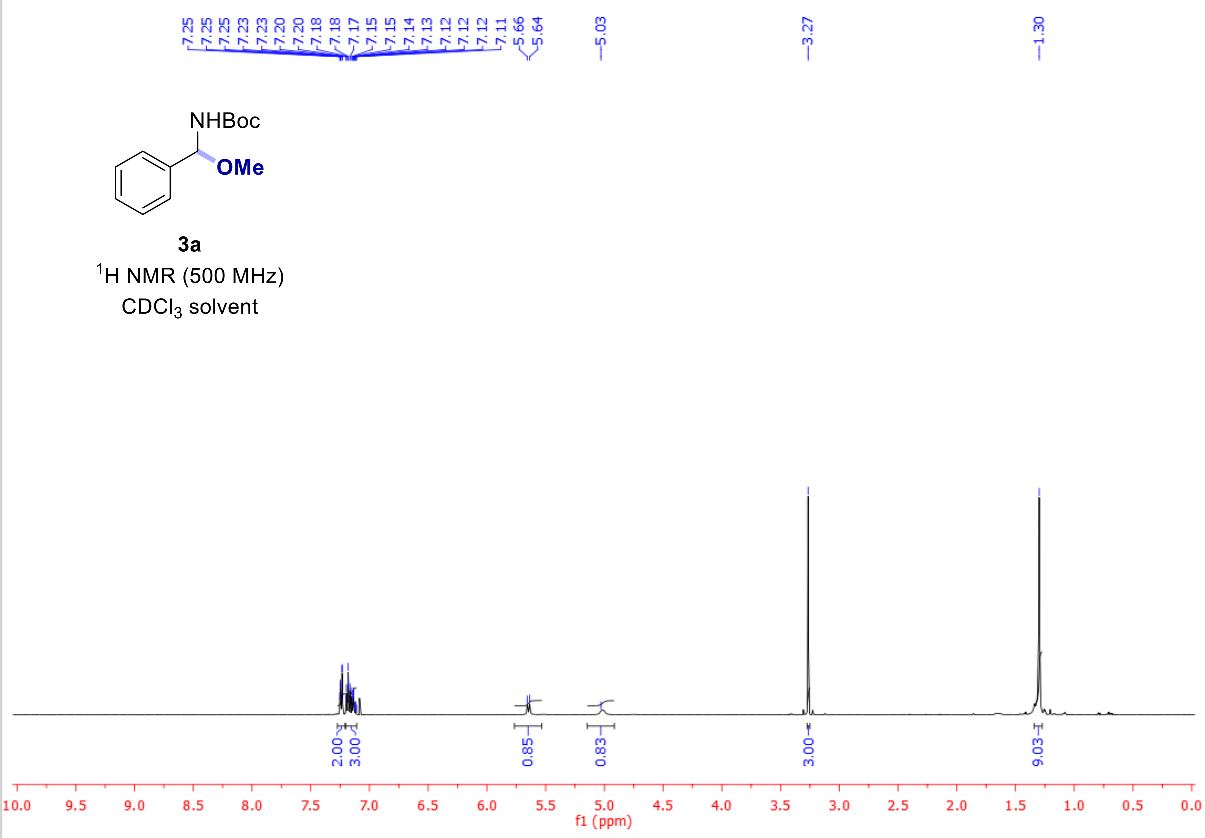

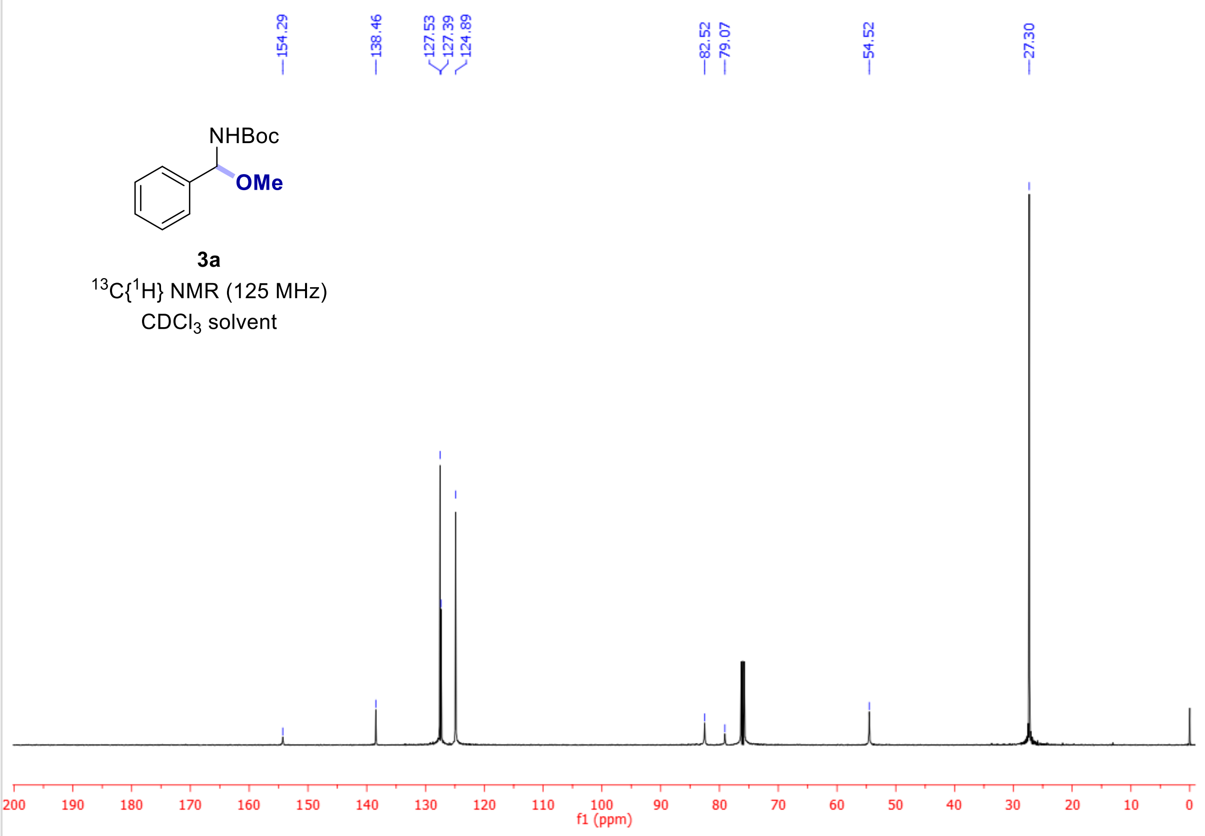

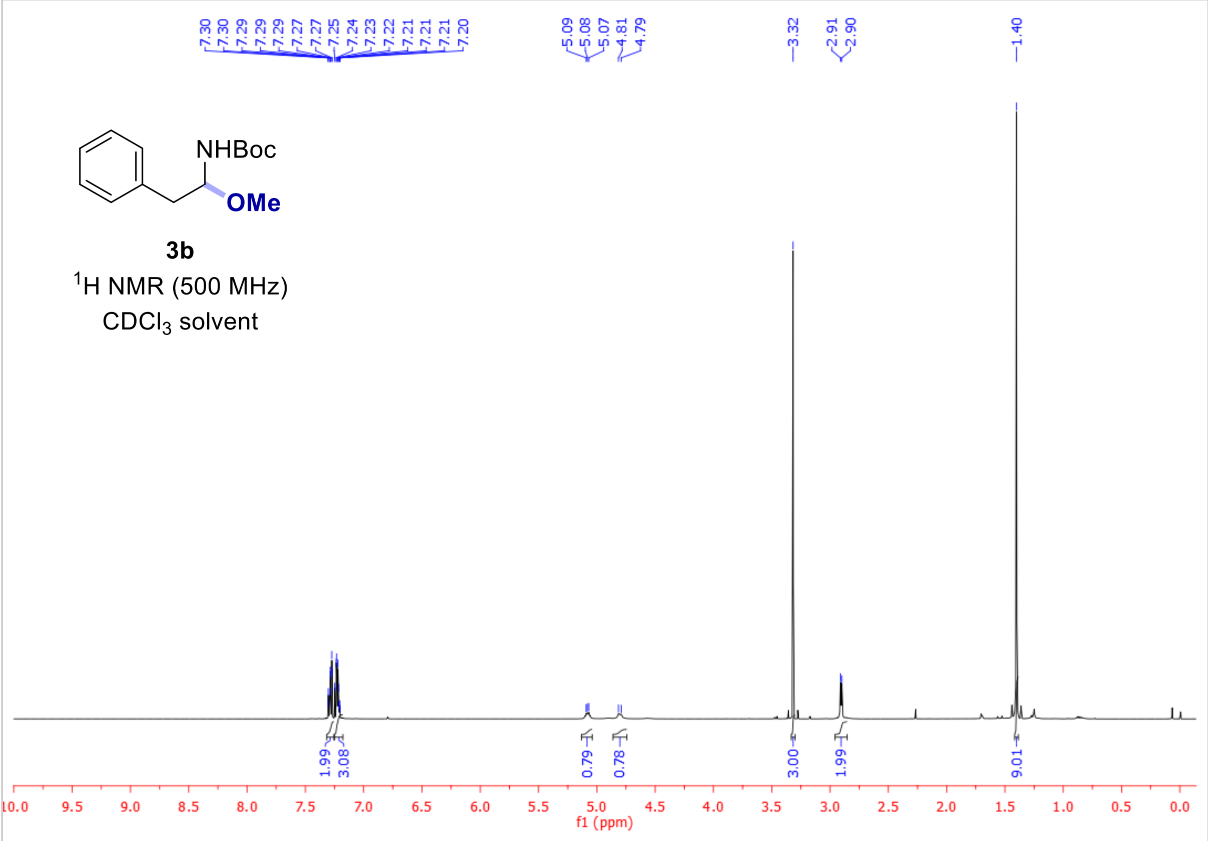
7.30 7.30 7.29 7.29 7.29 7.27 7.25 7.24 7.23 7.22 7.21 7.21 7.21 7.20
5.09 5.08 5.07 4.81 4.79
3.32
2.91 2.90
1.40
NHBoc
OMe
3b
1H NMR (500 MHz)
CDCl3 solvent
1.99 3.08 0.79 0.78 3.00 1.99 9.01
10.0 9.5 9.0 8.5 8.0 7.5 7.0 6.5 6.0 5.5 5.0 4.5 4.0 3.5 3.0 2.5 2.0 1.5 1.0 0.5 0.0
f1 (ppm)

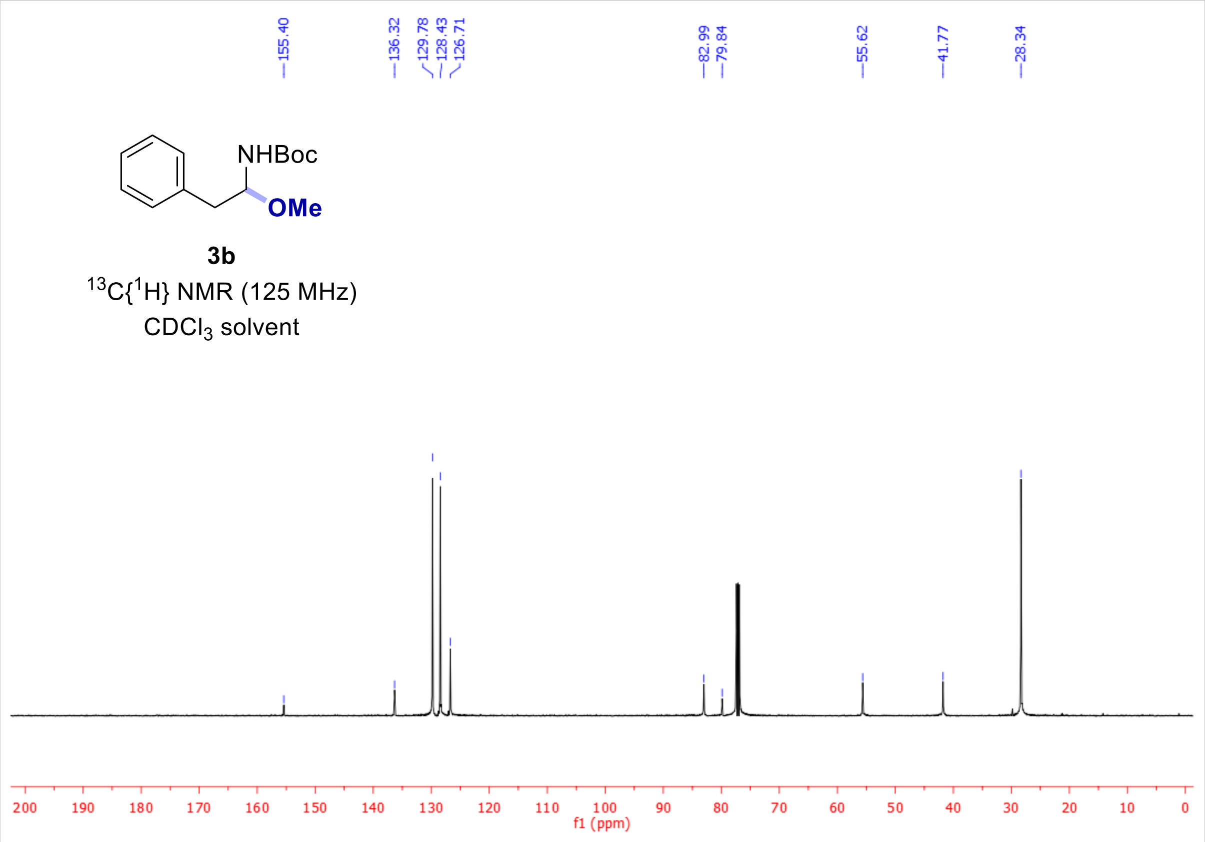
155.40
136.32 129.78 128.43 126.71
82.99 79.84
55.62
41.77
28.34
NHBoc
OMe
3b
13C{1H} NMR (125 MHz)
CDCl3 solvent
200 190 180 170 160 150 140 130 120 110 100 90 80 70 60 50 40 30 20 10 0
f1 (ppm)

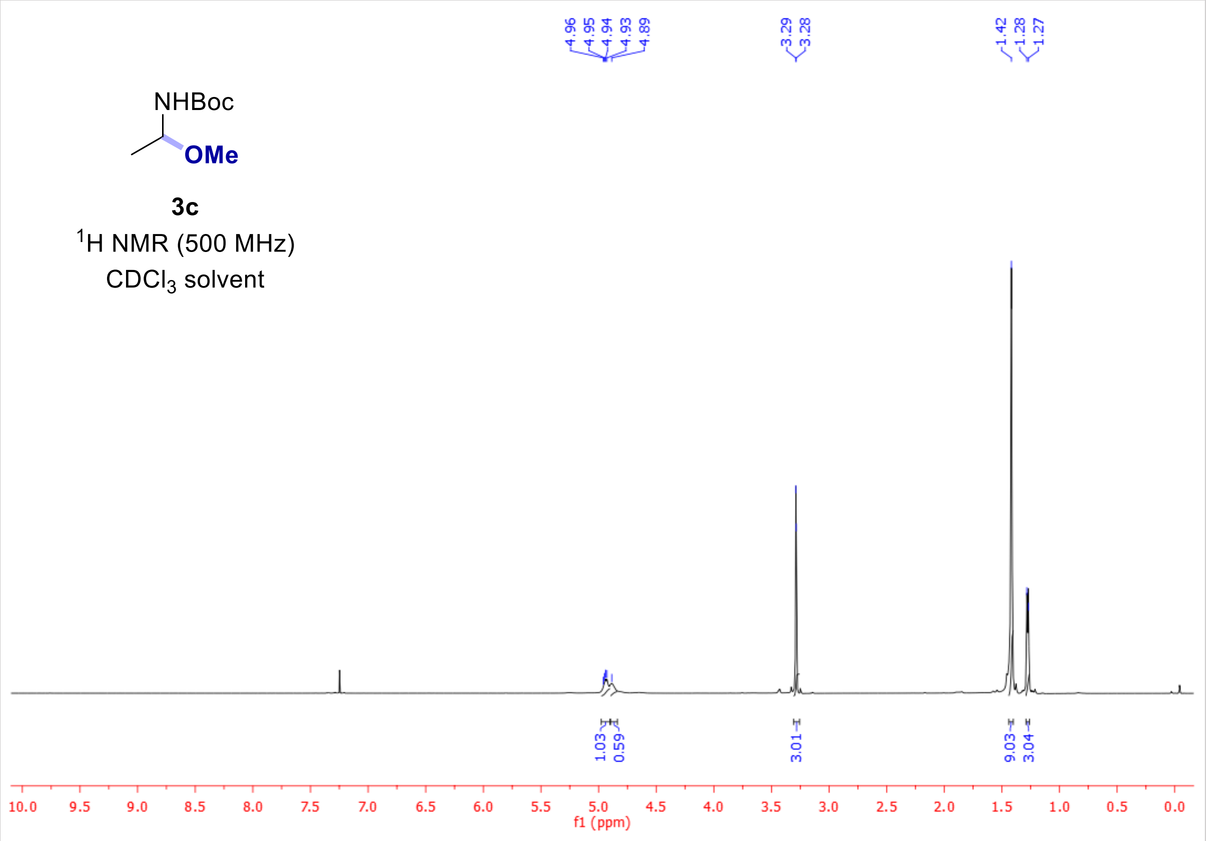
4.96
4.95
4.94
4.93
4.89
3.29
3.28
1.42
1.28
1.27
NHBoc
OMe
3c
^{1}H NMR (500 MHz)
$CDCl_3$ solvent
1.03
0.59
3.01
9.03
3.04
10.0 9.5 9.0 8.5 8.0 7.5 7.0 6.5 6.0 5.5 5.0 4.5 4.0 3.5 3.0 2.5 2.0 1.5 1.0 0.5 0.0
f1 (ppm)

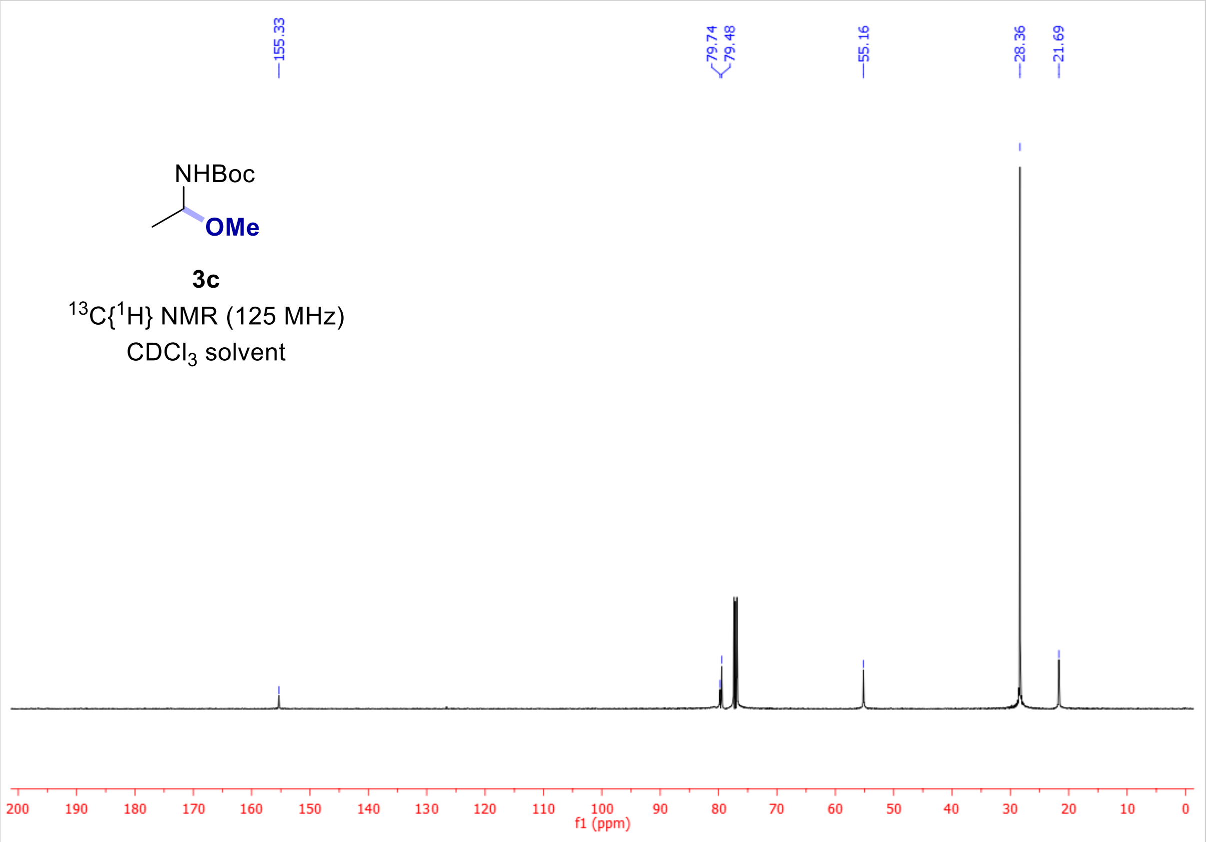
155.33
79.74
79.48
55.16
28.36
21.69
NHBoc
OMe
3c
^{13}C{^{1}H} NMR (125 MHz)
$CDCl_3$ solvent
200 190 180 170 160 150 140 130 120 110 100 90 80 70 60 50 40 30 20 10 0
f1 (ppm)

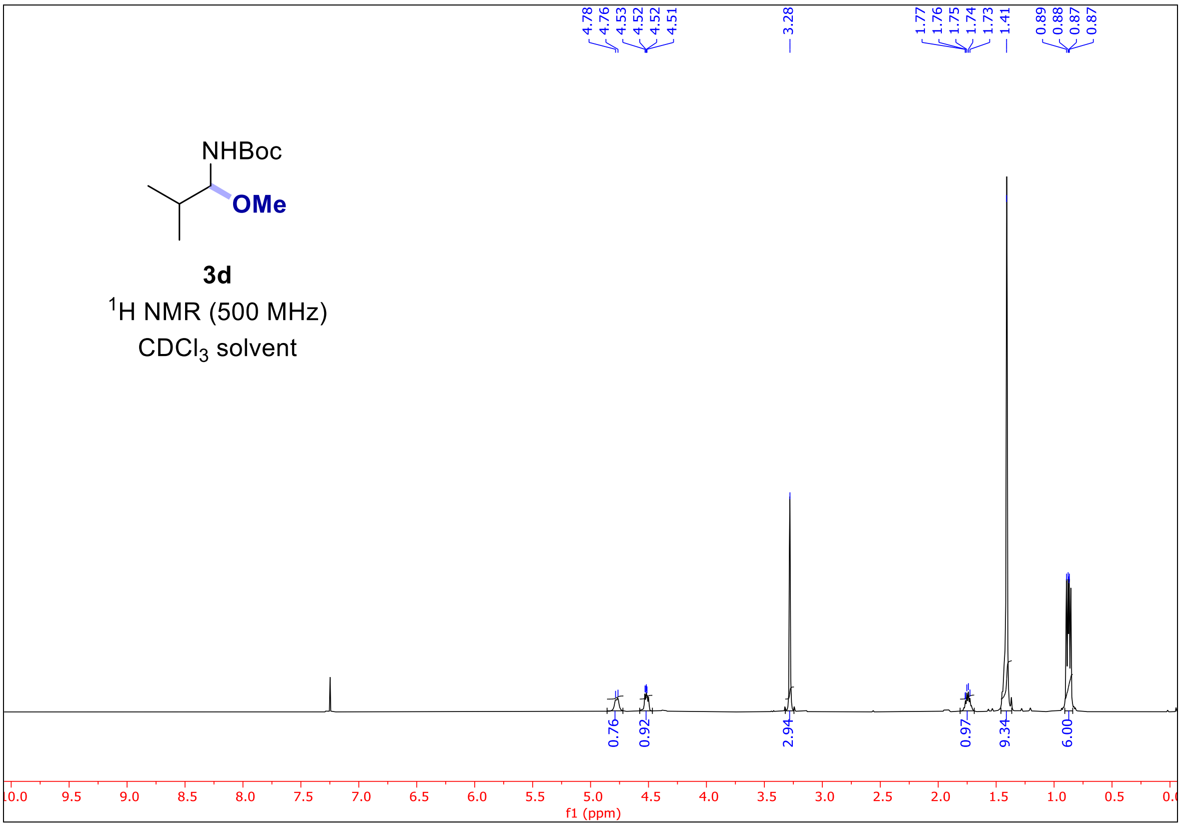
NHBoc
OMe
3d
1H NMR (500 MHz)
CDCl3 solvent
4.78 4.76 4.53 4.52 4.52 4.51
3.28
1.77 1.76 1.75 1.74 1.73 1.41
0.89 0.88 0.87 0.87
0.76 0.92 2.94 0.97 9.34 6.00
f1 (ppm)

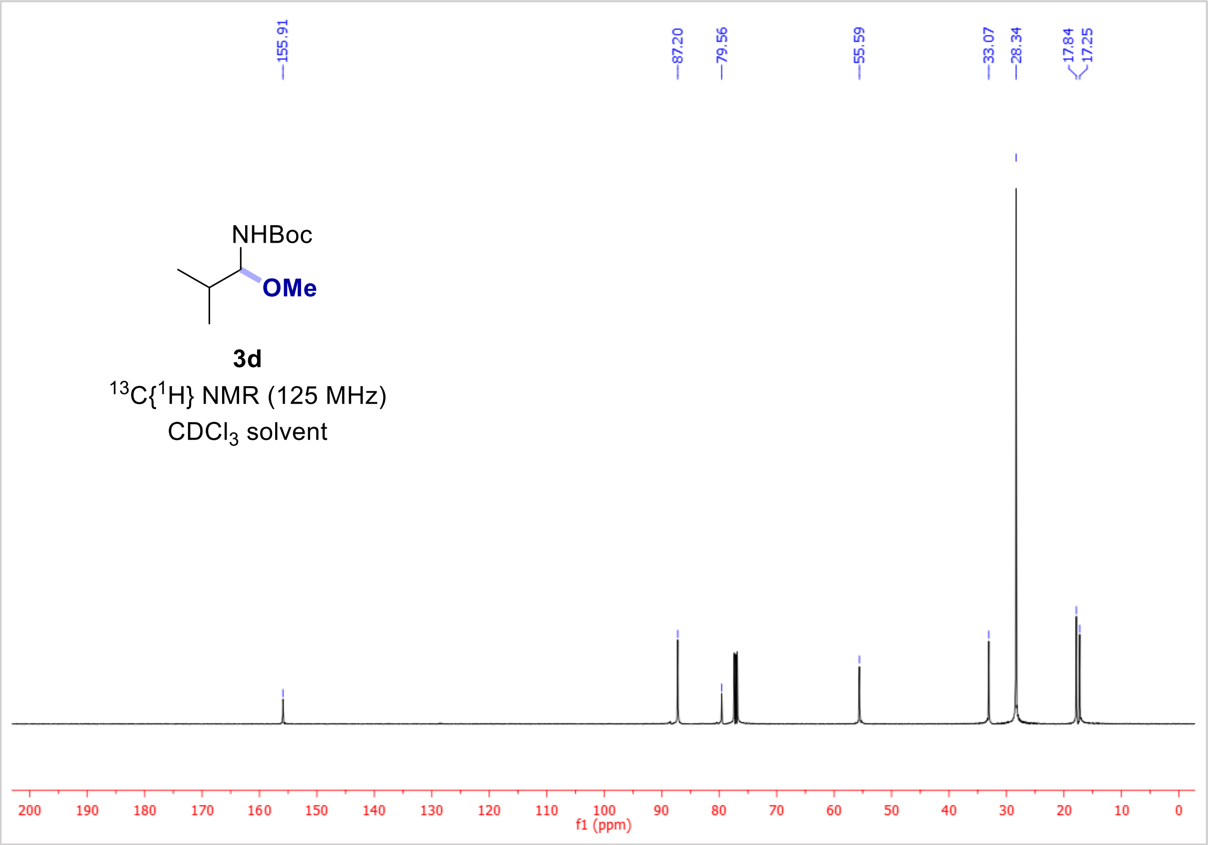
NHBoc
OMe
3d
13C{1H} NMR (125 MHz)
CDCl3 solvent
155.91
87.20
79.56
55.59
33.07
28.34
17.84
17.25
f1 (ppm)

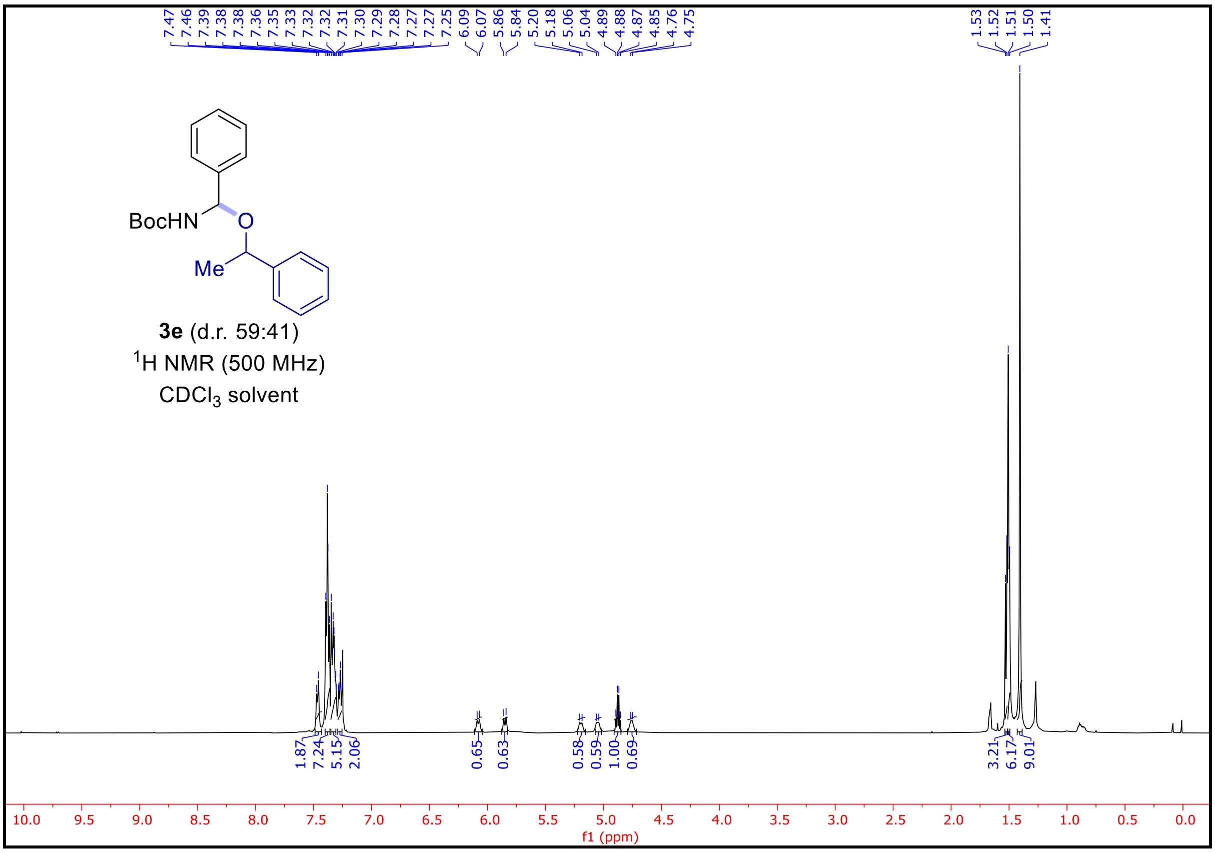
7.47
7.46
7.39
7.38
7.38
7.36
7.35
7.33
7.32
7.32
7.31
7.30
7.29
7.28
7.27
7.27
7.25
6.09
6.07
5.86
5.84
5.20
5.18
5.06
5.04
4.89
4.88
4.87
4.85
4.76
4.75
1.53
1.52
1.51
1.50
1.41
BocHN
O
Me
3e (d.r. 59:41)
1H NMR (500 MHz)
CDCl3 solvent
1.87
7.24
5.15
2.06
0.65
0.63
0.58
0.59
1.00
0.69
3.21
6.17
9.01
10.0
9.5
9.0
8.5
8.0
7.5
7.0
6.5
6.0
5.5
5.0
4.5
4.0
3.5
3.0
2.5
2.0
1.5
1.0
0.5
0.0
f1 (ppm)

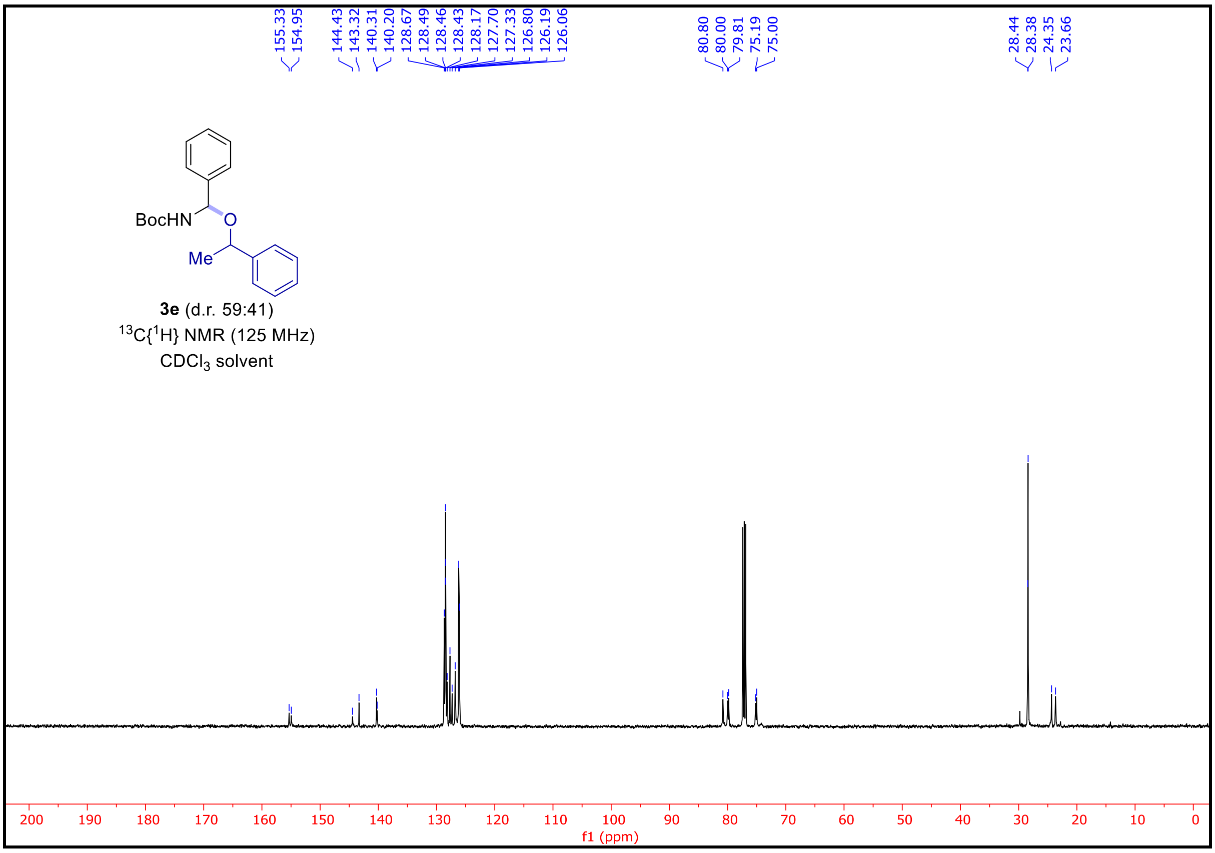
155.33
154.95
144.43
143.32
140.31
140.20
128.67
128.49
128.46
128.43
128.17
127.70
127.33
126.80
126.19
126.06
80.80
80.00
79.81
75.19
75.00
28.44
28.38
24.35
23.66
BocHN
O
Me
3e (d.r. 59:41)
13C{1H} NMR (125 MHz)
CDCl3 solvent
200
190
180
170
160
150
140
130
120
110
100
90
80
70
60
50
40
30
20
10
0
f1 (ppm)

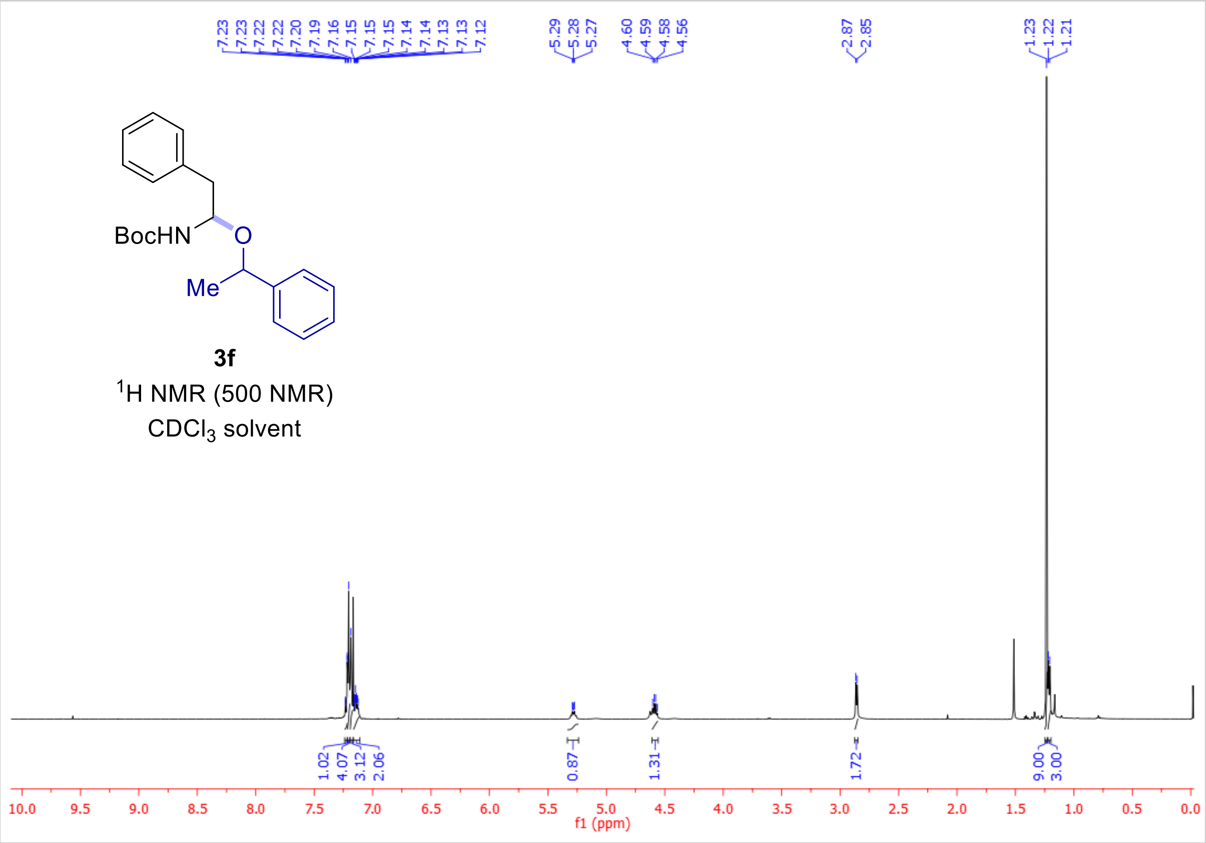
BocHN
O
Me
3f
1H NMR (500 NMR)
CDCl3 solvent
7.23 7.23 7.22 7.22 7.20 7.19 7.16 7.15 7.15 7.15 7.14 7.14 7.13 7.13 7.12
5.29 5.28 5.27
4.60 4.59 4.58 4.56
2.87 2.85
1.23 1.22 1.21
1.02 4.07 3.12 2.06
0.87
1.31
1.72
9.00 3.00
f1 (ppm)

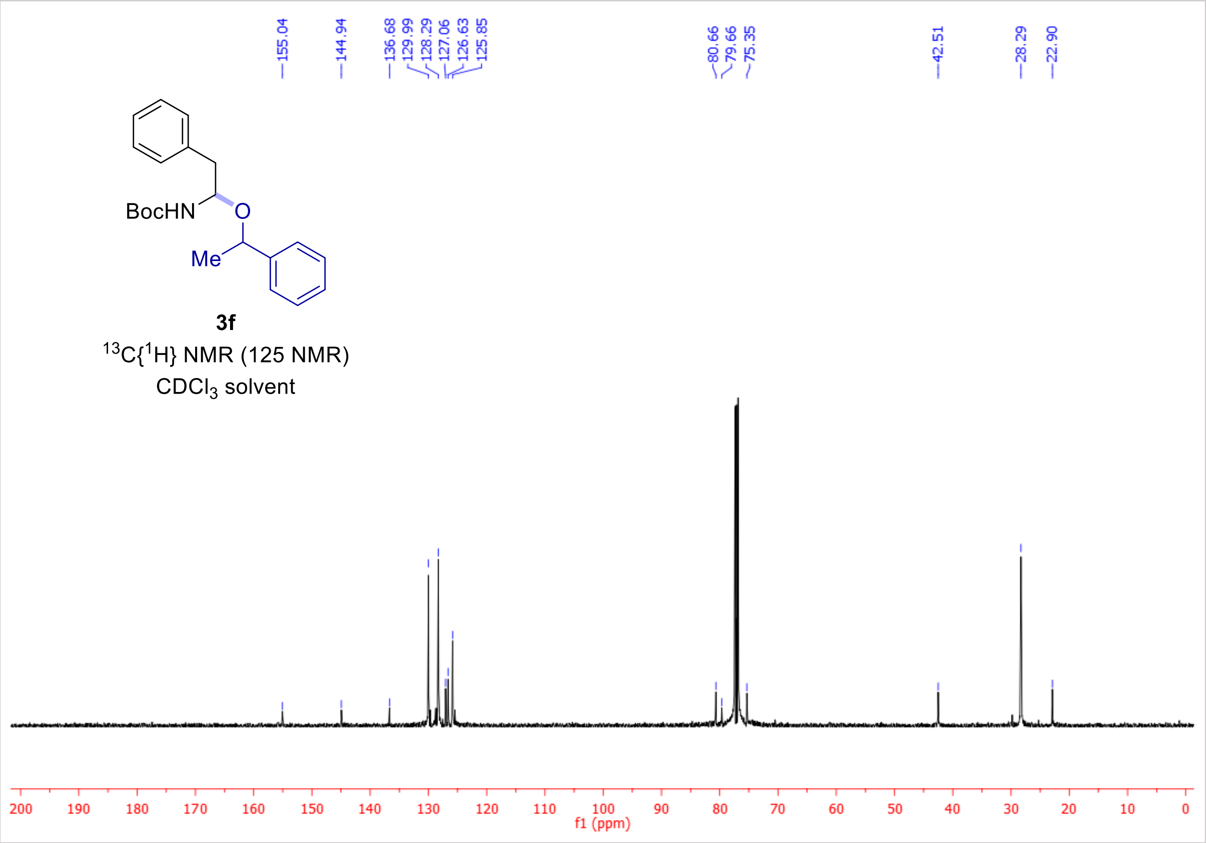
BocHN
O
Me
3f
13C{1H} NMR (125 NMR)
CDCl3 solvent
155.04
144.94
136.68 129.99 128.29 127.06 126.63 125.85
80.66 79.66 75.35
42.51
28.29
22.90
f1 (ppm)

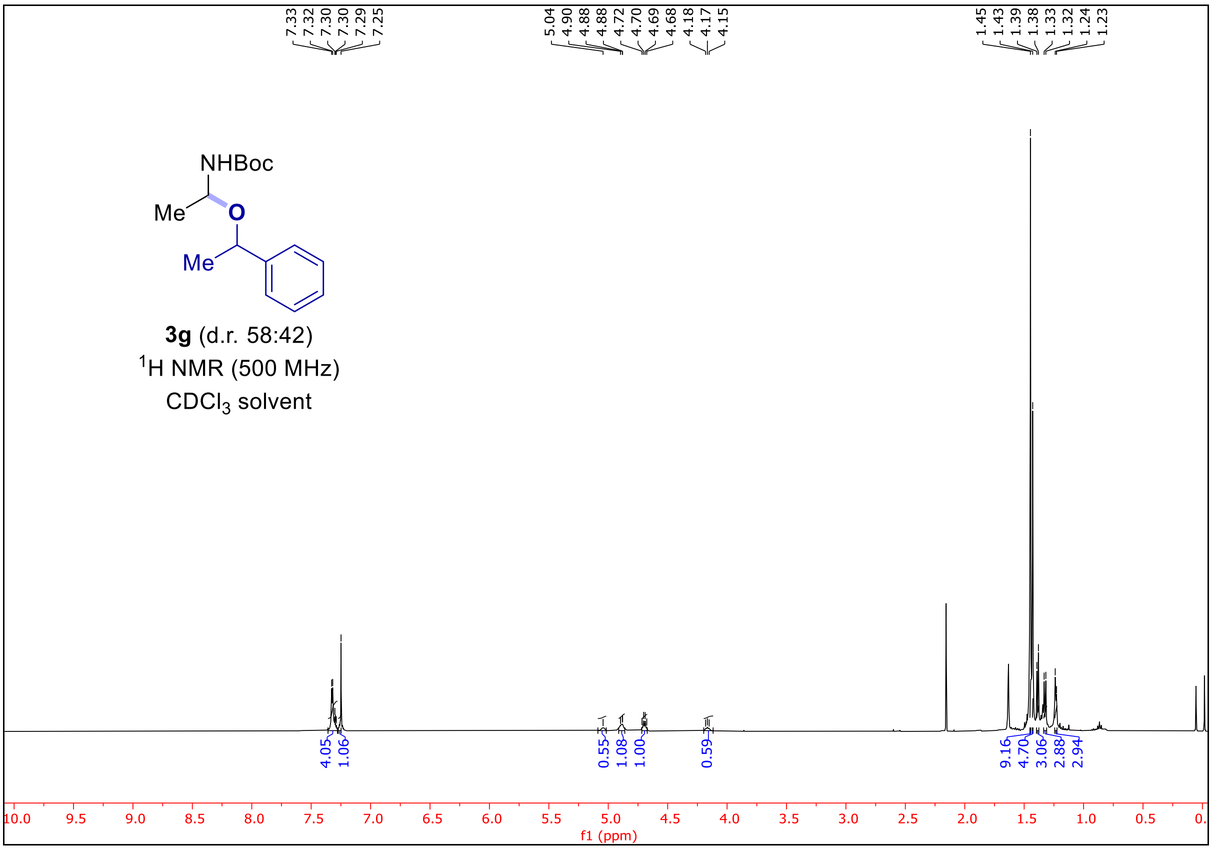

7.33
7.32
7.30
7.30
7.29
7.25
5.04
4.90
4.88
4.88
4.72
4.70
4.69
4.68
4.18
4.17
4.15
1.45
1.43
1.39
1.38
1.33
1.32
1.24
1.23
NHBoc
Me
O
Me
3g (d.r. 58:42)
1H NMR (500 MHz)
CDCl3 solvent
4.05
1.06
0.55
1.08
1.00
0.59
9.16
4.70
3.06
2.88
2.94
10.0
9.5
9.0
8.5
8.0
7.5
7.0
6.5
6.0
5.5
5.0
4.5
4.0
3.5
3.0
2.5
2.0
1.5
1.0
0.5
f1 (ppm)

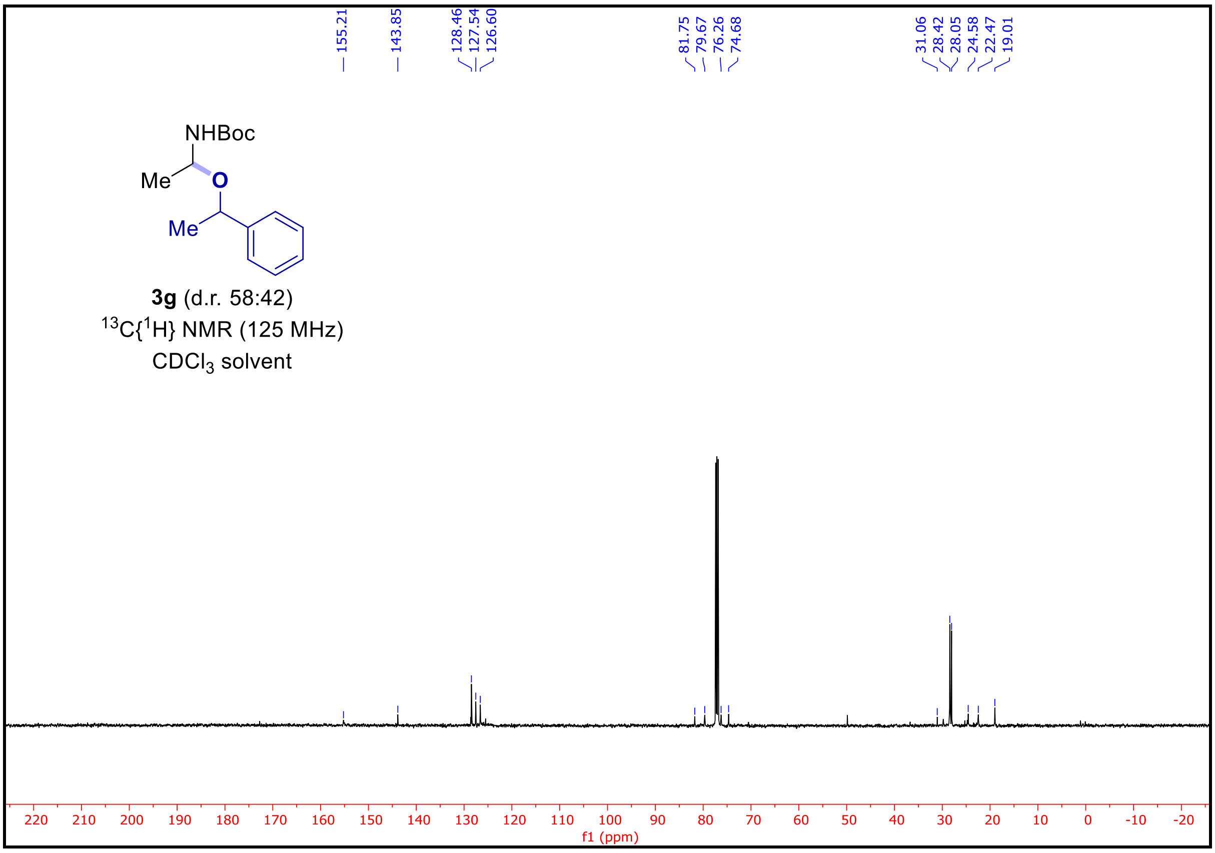

155.21
143.85
128.46
127.54
126.60
81.75
79.67
76.26
74.68
31.06
28.42
28.05
24.58
22.47
19.01
NHBoc
Me
O
Me
3g (d.r. 58:42)
13C{1H} NMR (125 MHz)
CDCl3 solvent
220
210
200
190
180
170
160
150
140
130
120
110
100
90
80
70
60
50
40
30
20
10
0
-10
-20
f1 (ppm)

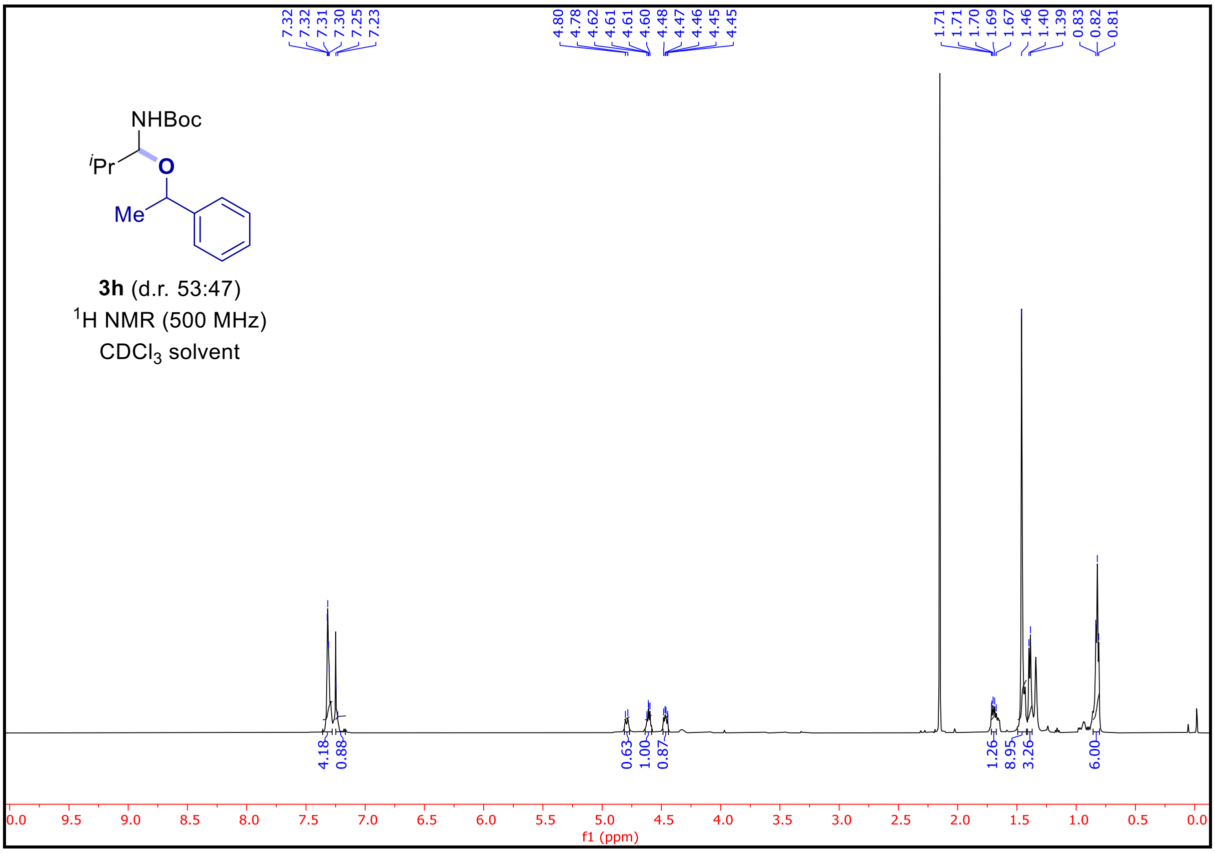
NHBoc
iPr
O
Me
3h (d.r. 53:47)
1H NMR (500 MHz)
CDCl3 solvent
7.32
7.32
7.31
7.30
7.25
7.23
4.80
4.78
4.62
4.61
4.61
4.60
4.48
4.47
4.46
4.45
4.45
1.71
1.71
1.70
1.69
1.67
1.46
1.40
1.39
0.83
0.82
0.81
4.18
0.88
0.63
1.00
0.87
1.26
8.95
3.26
6.00
f1 (ppm)

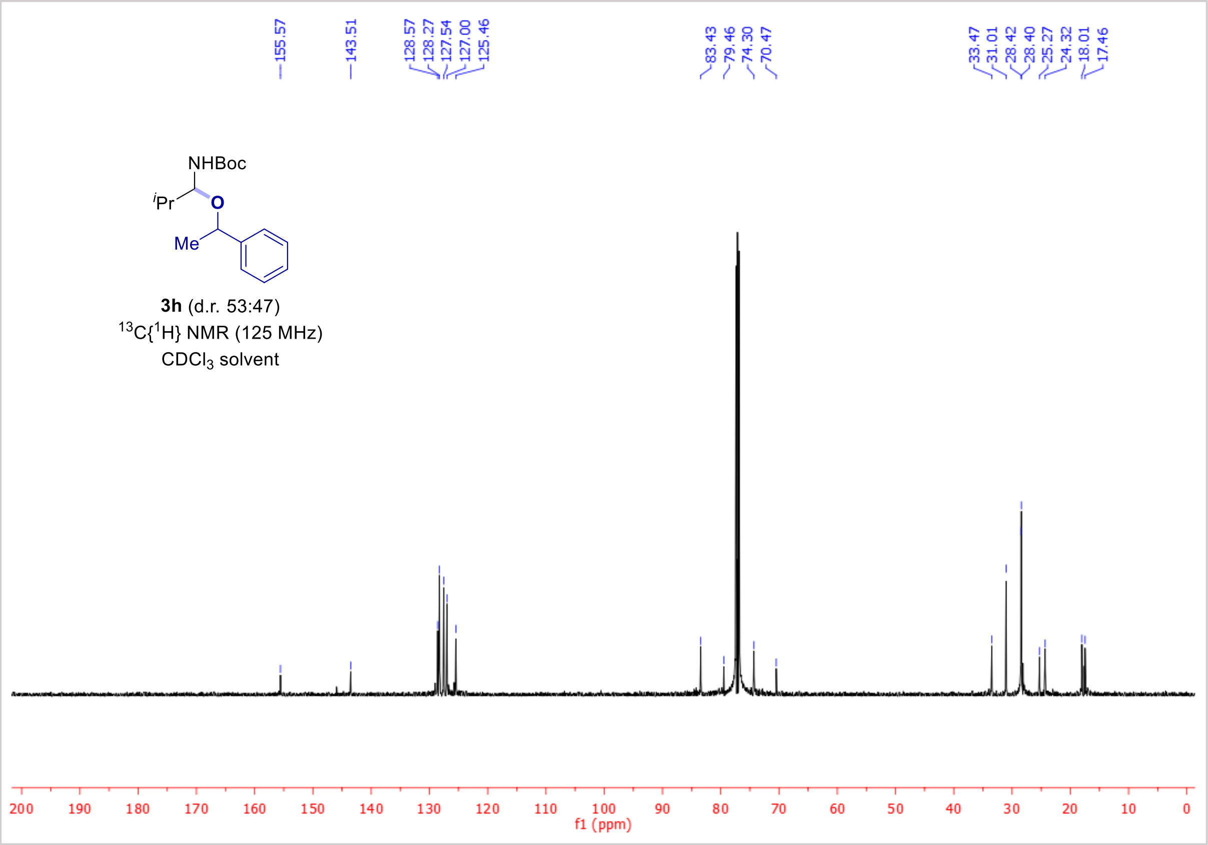
NHBoc
iPr
O
Me
3h (d.r. 53:47)
13C{1H} NMR (125 MHz)
CDCl3 solvent
155.57
143.51
128.57
128.27
127.54
127.00
125.46
83.43
79.46
74.30
70.47
33.47
31.01
28.42
28.40
25.27
24.32
18.01
17.46
f1 (ppm)

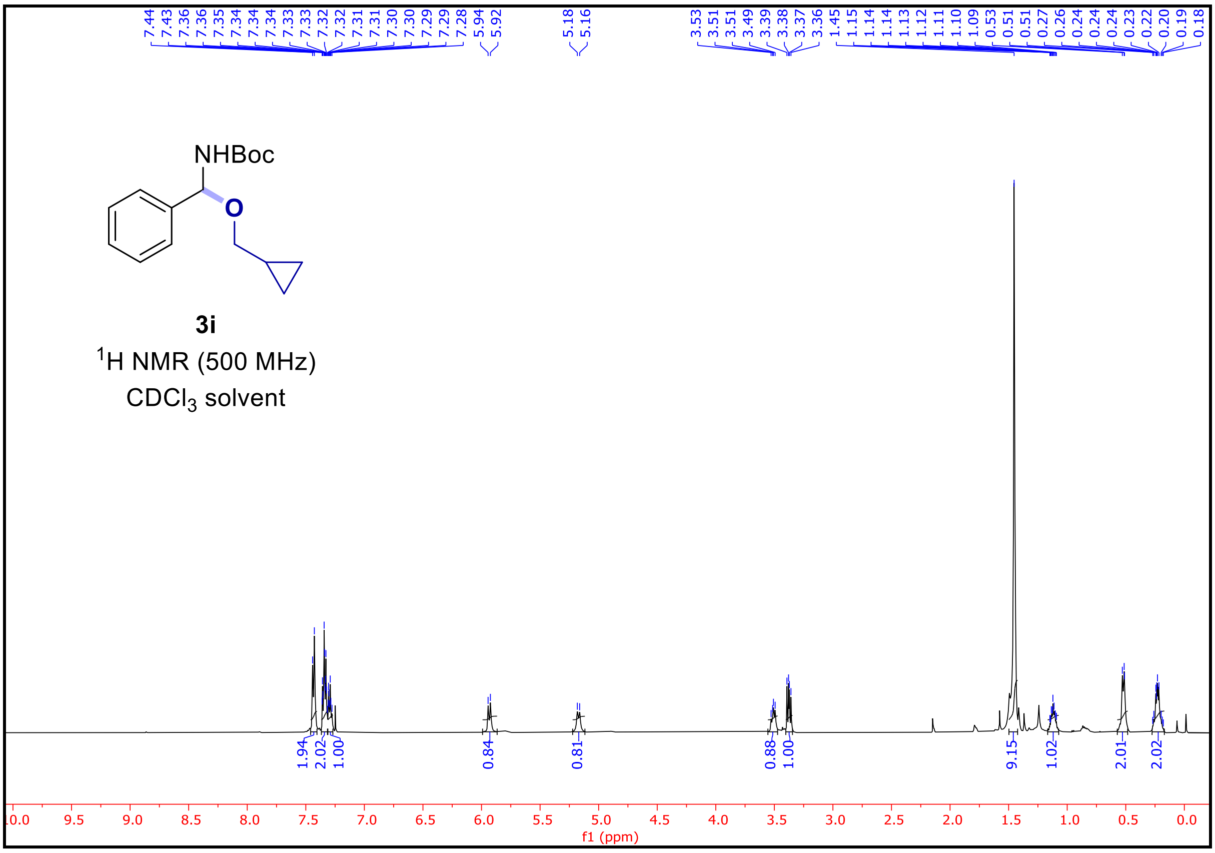
7.44 7.43 7.36 7.36 7.35 7.34 7.34 7.34 7.33 7.33 7.32 7.32 7.32 7.31 7.31 7.30 7.30 7.29 7.29 7.28 5.94 5.92 5.18 5.16 3.53 3.51 3.51 3.49 3.39 3.38 3.37 3.36 1.45 1.15 1.14 1.14 1.13 1.12 1.11 1.10 1.09 0.53 0.51 0.51 0.27 0.26 0.24 0.24 0.24 0.23 0.22 0.20 0.19 0.18
NHBoc
O
3i
1H NMR (500 MHz)
CDCl3 solvent
1.94 2.02 1.00 0.84 0.81 0.88 1.00 9.15 1.02 2.01 2.02
10.0 9.5 9.0 8.5 8.0 7.5 7.0 6.5 6.0 5.5 5.0 4.5 4.0 3.5 3.0 2.5 2.0 1.5 1.0 0.5 0.0
f1 (ppm)

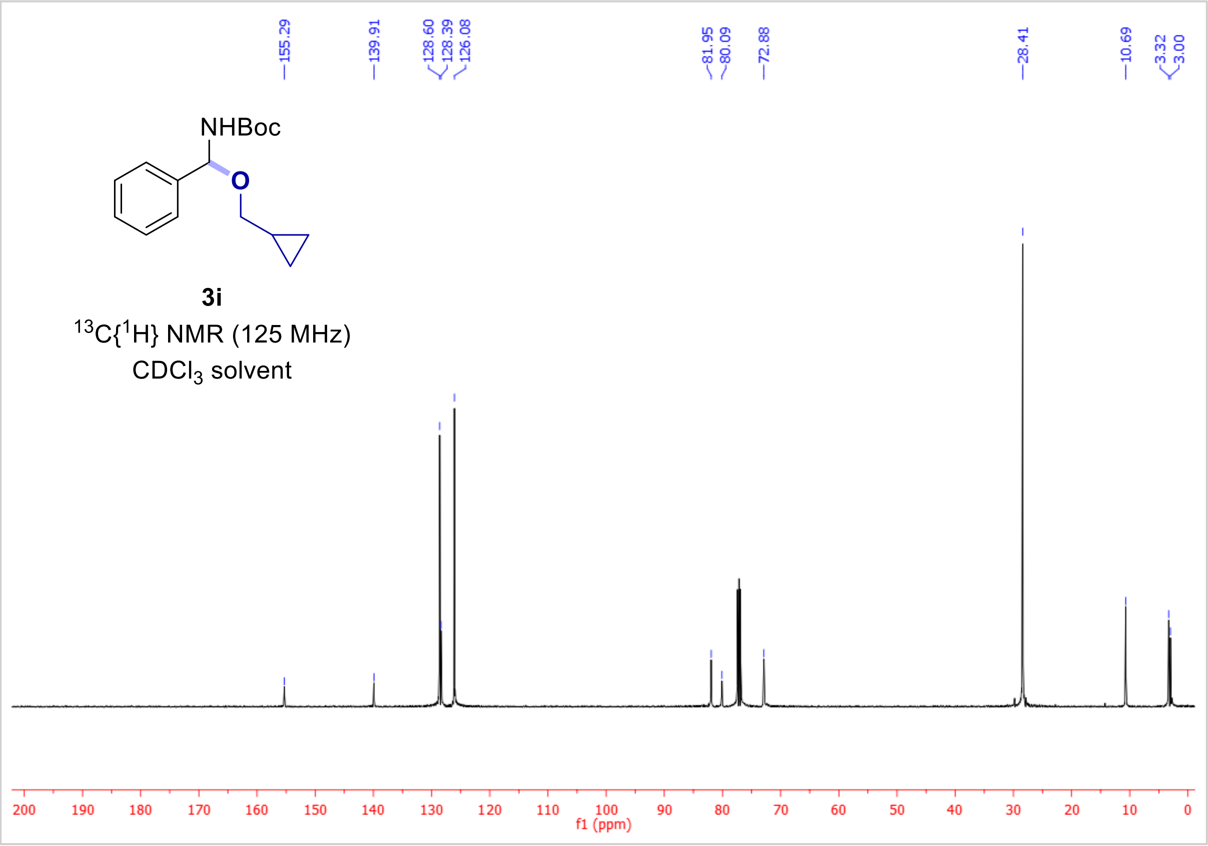
155.29 139.91 128.60 128.39 126.08 81.95 80.09 72.88 28.41 10.69 3.32 3.00
NHBoc
O
3i
13C{1H} NMR (125 MHz)
CDCl3 solvent
200 190 180 170 160 150 140 130 120 110 100 90 80 70 60 50 40 30 20 10 0
f1 (ppm)

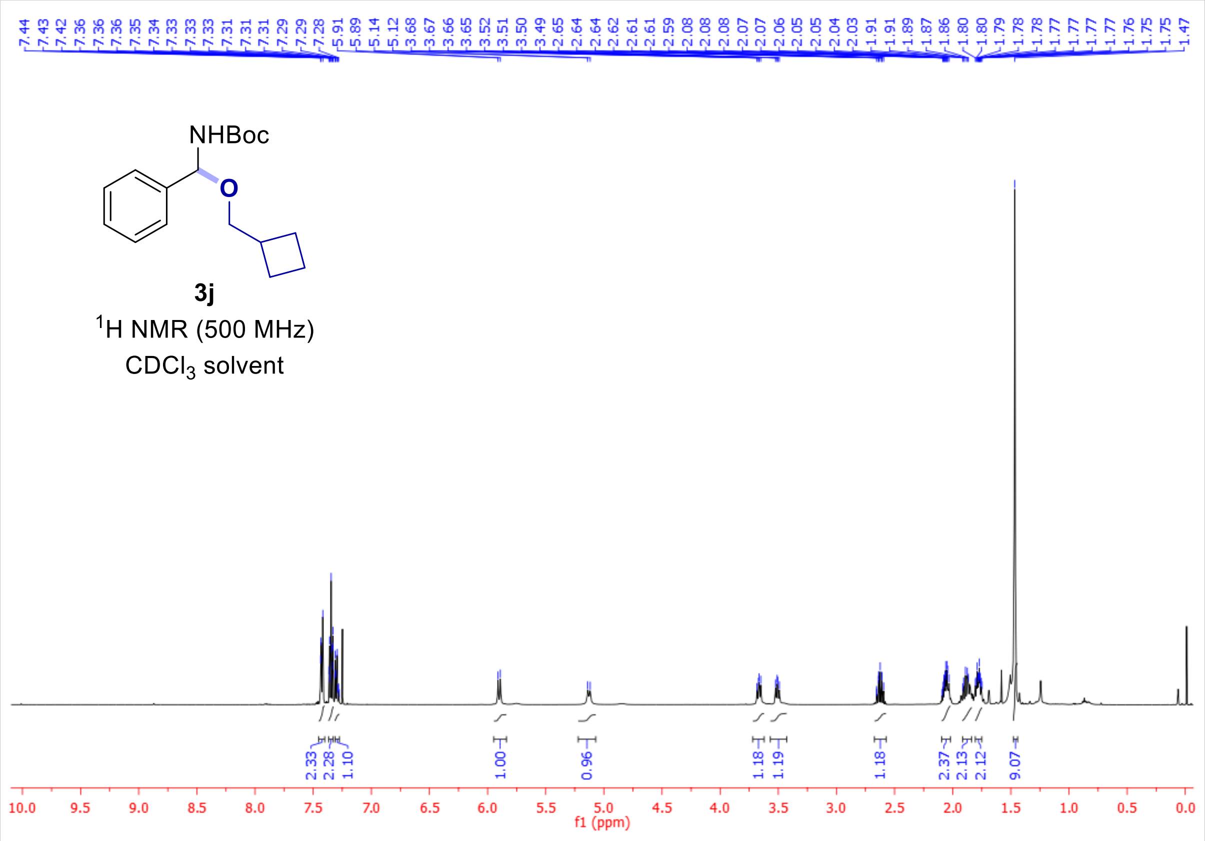
7.44 7.43 7.42 7.36 7.36 7.36 7.35 7.34 7.33 7.33 7.33 7.31 7.31 7.31 7.29 7.29 7.28 5.91 5.89 5.14 5.12 3.68 3.67 3.66 3.65 3.52 3.51 3.50 3.49 2.65 2.64 2.64 2.62 2.61 2.61 2.59 2.08 2.08 2.08 2.07 2.07 2.06 2.05 2.05 2.04 2.03 1.91 1.91 1.89 1.87 1.86 1.80 1.80 1.79 1.78 1.78 1.77 1.77 1.77 1.77 1.76 1.75 1.75 1.47
NHBoc
O
3j
1H NMR (500 MHz)
CDCl3 solvent
2.33 2.28 1.10 1.00 0.96 1.18 1.19 1.18 2.37 2.13 2.12 9.07
10.0 9.5 9.0 8.5 8.0 7.5 7.0 6.5 6.0 5.5 5.0 4.5 4.0 3.5 3.0 2.5 2.0 1.5 1.0 0.5 0.0
f1 (ppm)

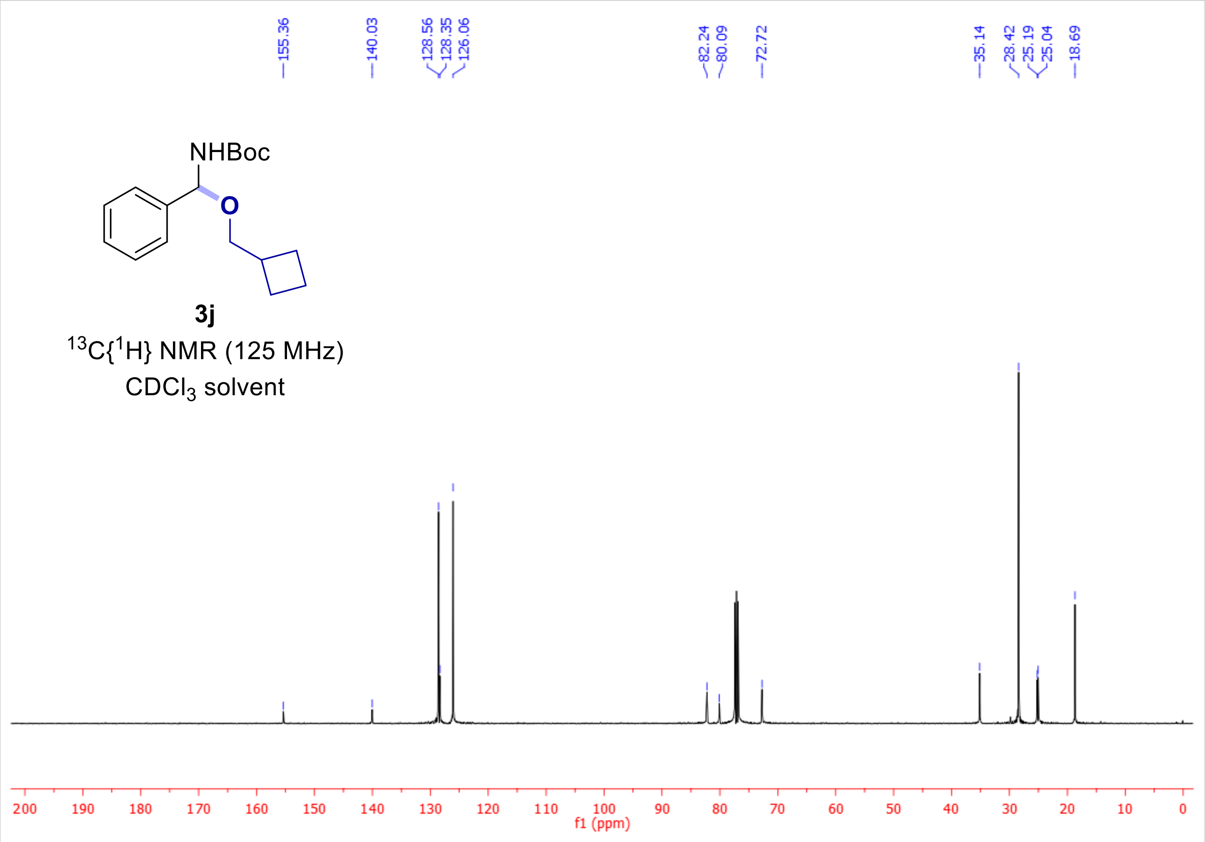
155.36 140.03 128.56 128.35 126.06 82.24 80.09 72.72 35.14 28.42 25.19 25.04 18.69
NHBoc
O
3j
13C{1H} NMR (125 MHz)
CDCl3 solvent
200 190 180 170 160 150 140 130 120 110 100 90 80 70 60 50 40 30 20 10 0
f1 (ppm)

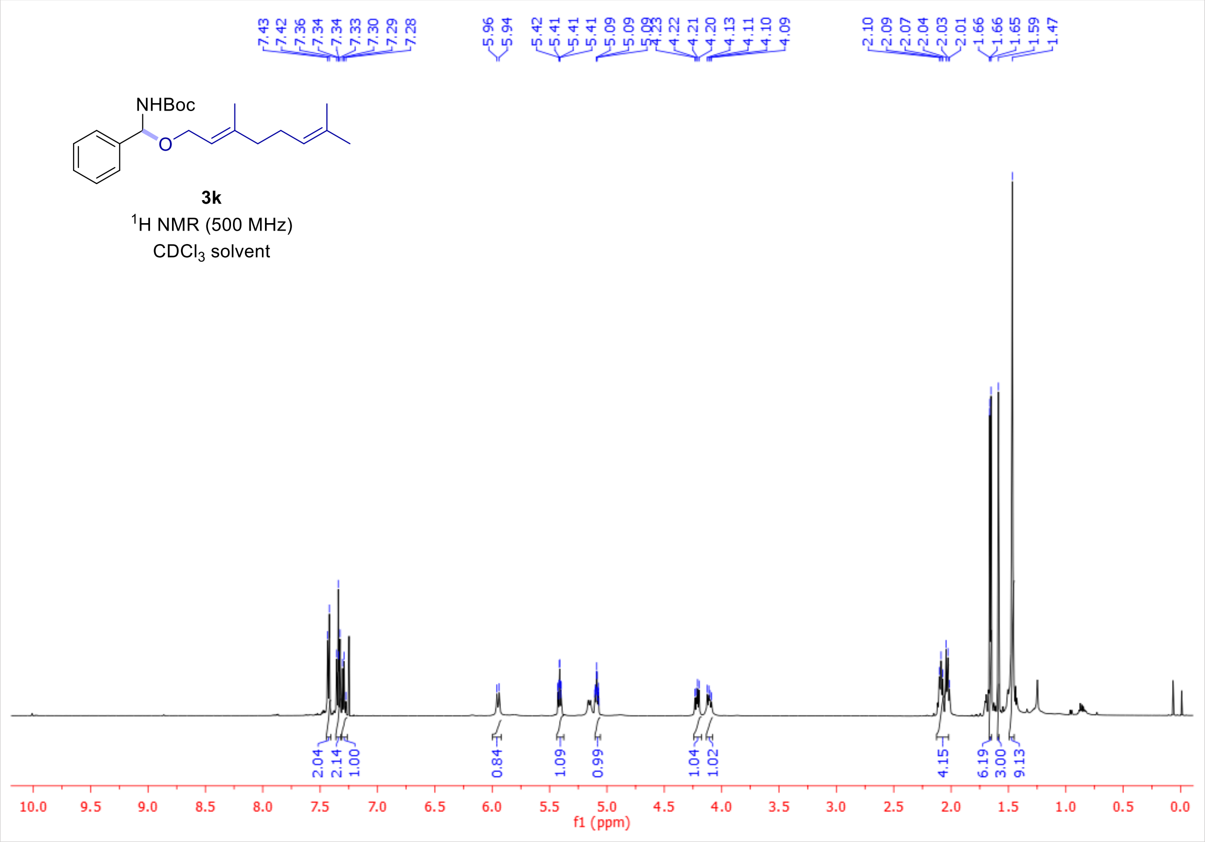

7.43
7.42
7.36
7.34
7.34
7.33
7.30
7.29
7.28
5.96
5.94
5.42
5.41
5.41
5.41
5.09
5.09
4.23
4.22
4.21
4.20
4.13
4.11
4.10
4.09
2.10
2.09
2.07
2.04
2.03
2.01
1.66
1.65
1.59
1.47
NHBoc
O
3k
^{1}H NMR (500 MHz)
$CDCl_3$ solvent
2.04
2.14
1.00
0.84
1.09
0.99
1.04
1.02
4.15
6.19
3.00
9.13
10.0 9.5 9.0 8.5 8.0 7.5 7.0 6.5 6.0 5.5 5.0 4.5 4.0 3.5 3.0 2.5 2.0 1.5 1.0 0.5 0.0
f1 (ppm)

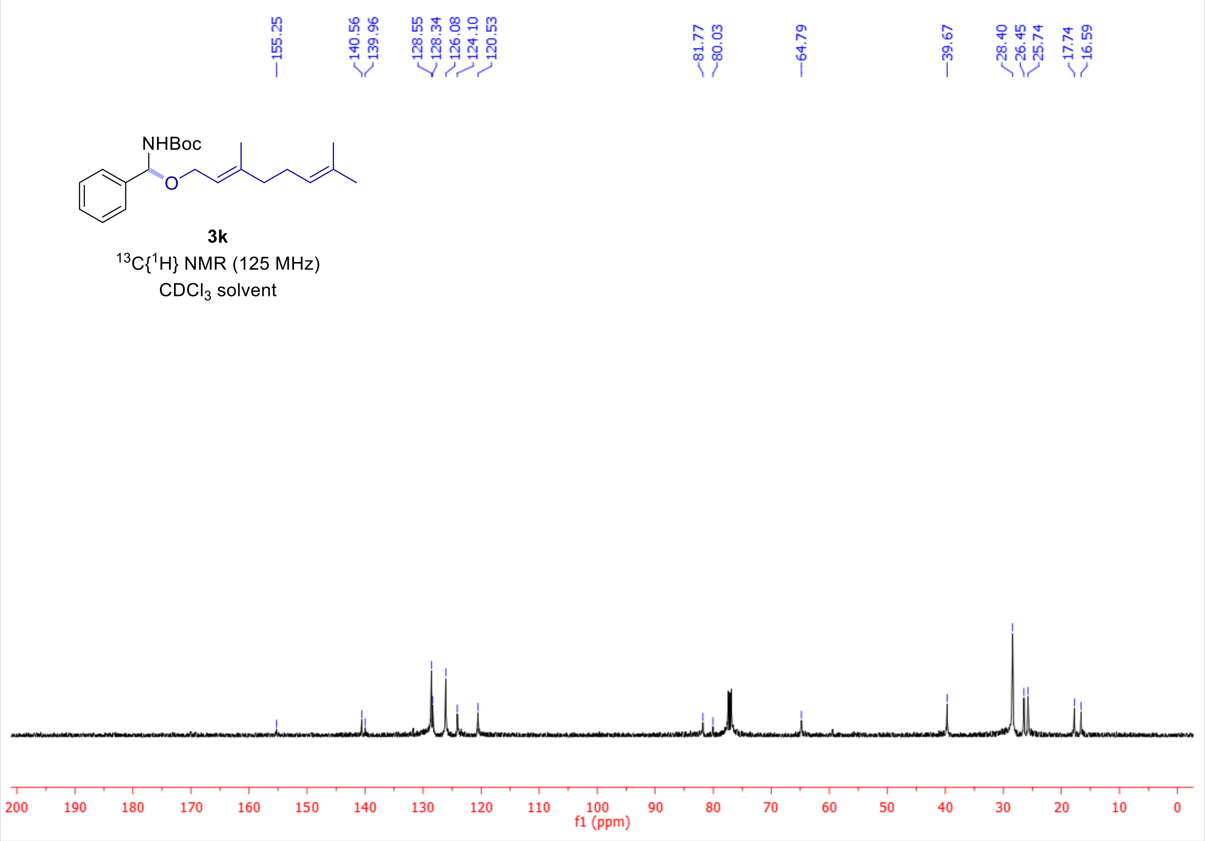

155.25
140.56
139.96
128.55
128.34
126.08
124.10
120.53
81.77
80.03
64.79
39.67
28.40
26.45
25.74
17.74
16.59
NHBoc
O
3k
$^{13}C\{^{1}H\}$ NMR (125 MHz)
$CDCl_3$ solvent
200 190 180 170 160 150 140 130 120 110 100 90 80 70 60 50 40 30 20 10 0
f1 (ppm)

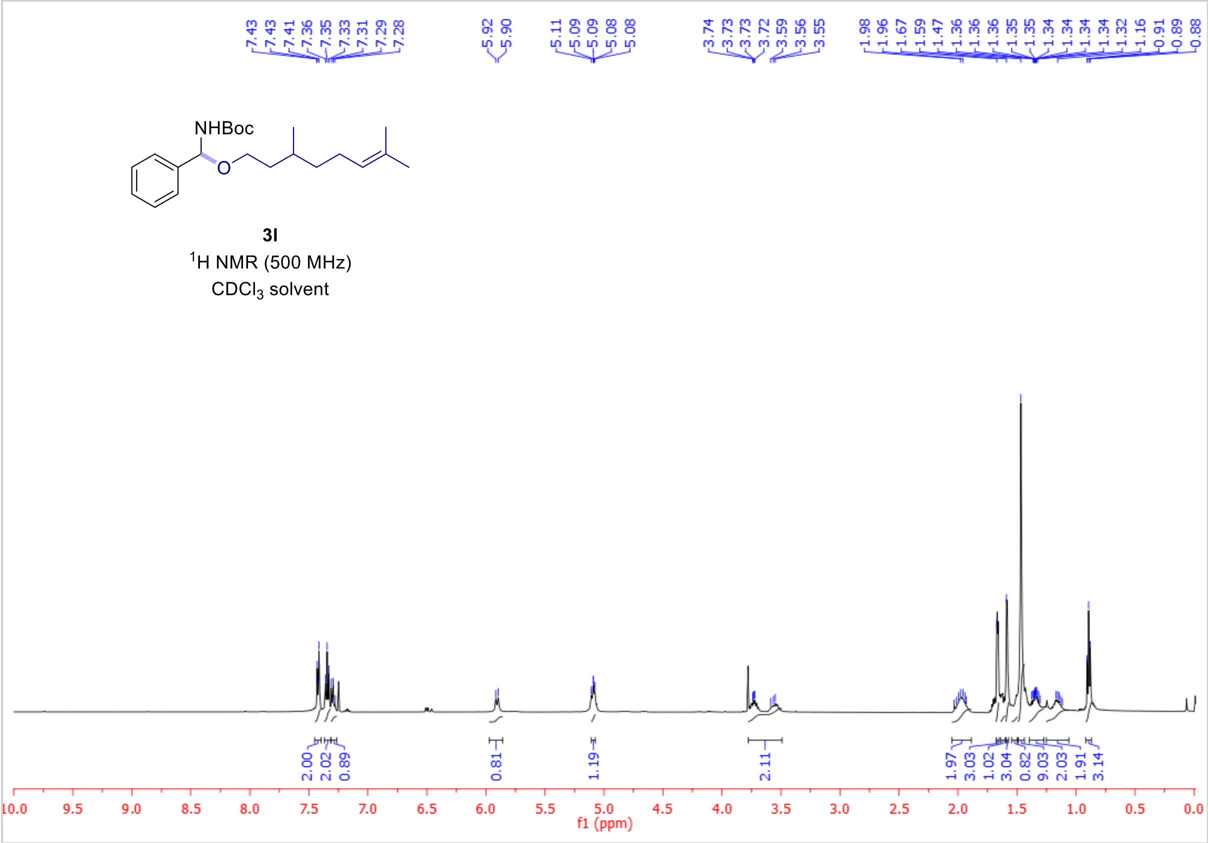
7.43 7.43 7.41 7.36 7.35 7.33 7.31 7.29 7.28
5.92 5.90
5.11 5.09 5.09 5.08 5.08
3.74 3.73 3.73 3.72 3.59 3.56 3.55
1.98 1.96 1.67 1.59 1.47 1.36 1.36 1.36 1.35 1.35 1.34 1.34 1.34 1.34 1.32 1.16 0.91 0.89 0.88
NHBoc
O
3l
1H NMR (500 MHz)
CDCl3 solvent
2.00 2.02 0.89
0.81
1.19
2.11
1.97 3.03 1.02 3.04 0.82 9.03 2.03 1.91 3.14
10.0 9.5 9.0 8.5 8.0 7.5 7.0 6.5 6.0 5.5 5.0 4.5 4.0 3.5 3.0 2.5 2.0 1.5 1.0 0.5 0.0
f1 (ppm)

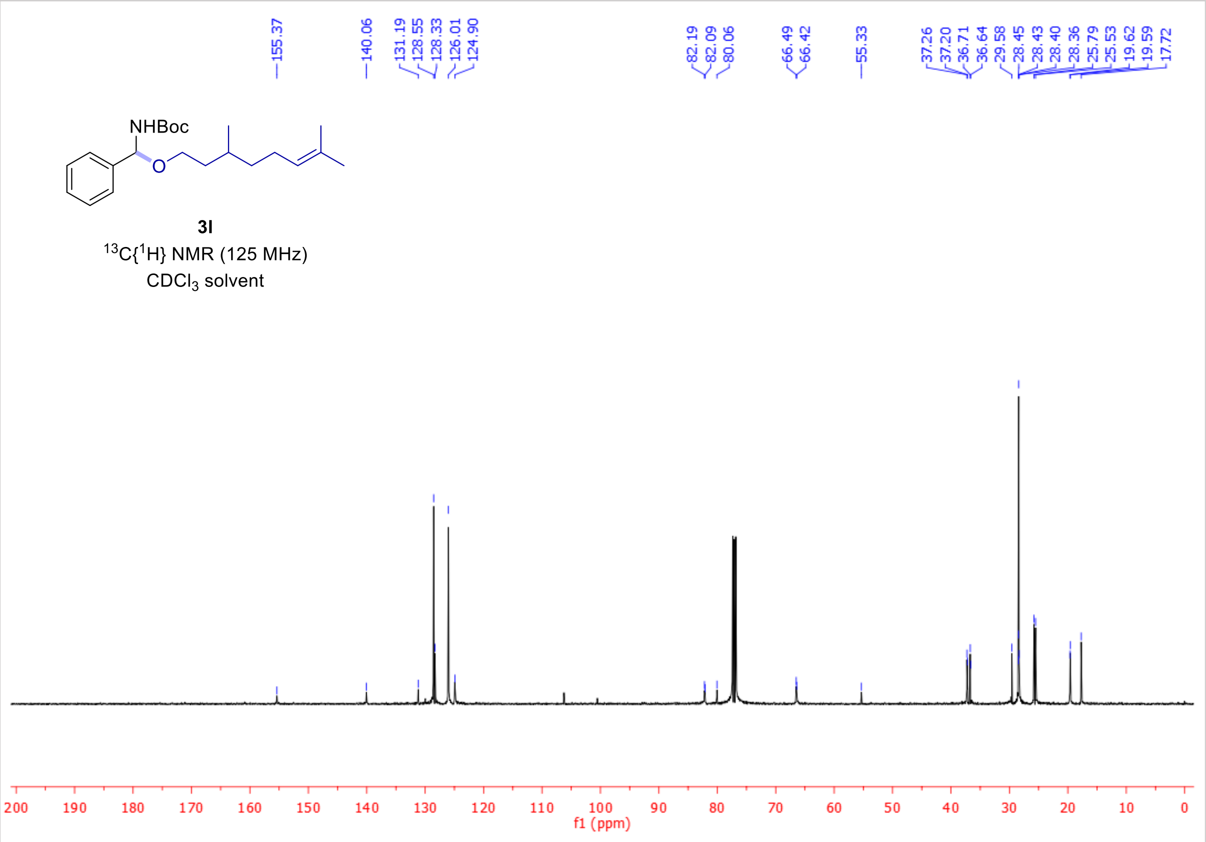
155.37
140.06
131.19 128.55 128.33 126.01 124.90
82.19 82.09 80.06
66.49 66.42
55.33
37.26 37.20 36.71 36.64 29.58 28.45 28.43 28.40 28.36 25.79 25.53 19.62 19.59 17.72
NHBoc
O
3l
13C{1H} NMR (125 MHz)
CDCl3 solvent
200 190 180 170 160 150 140 130 120 110 100 90 80 70 60 50 40 30 20 10 0
f1 (ppm)

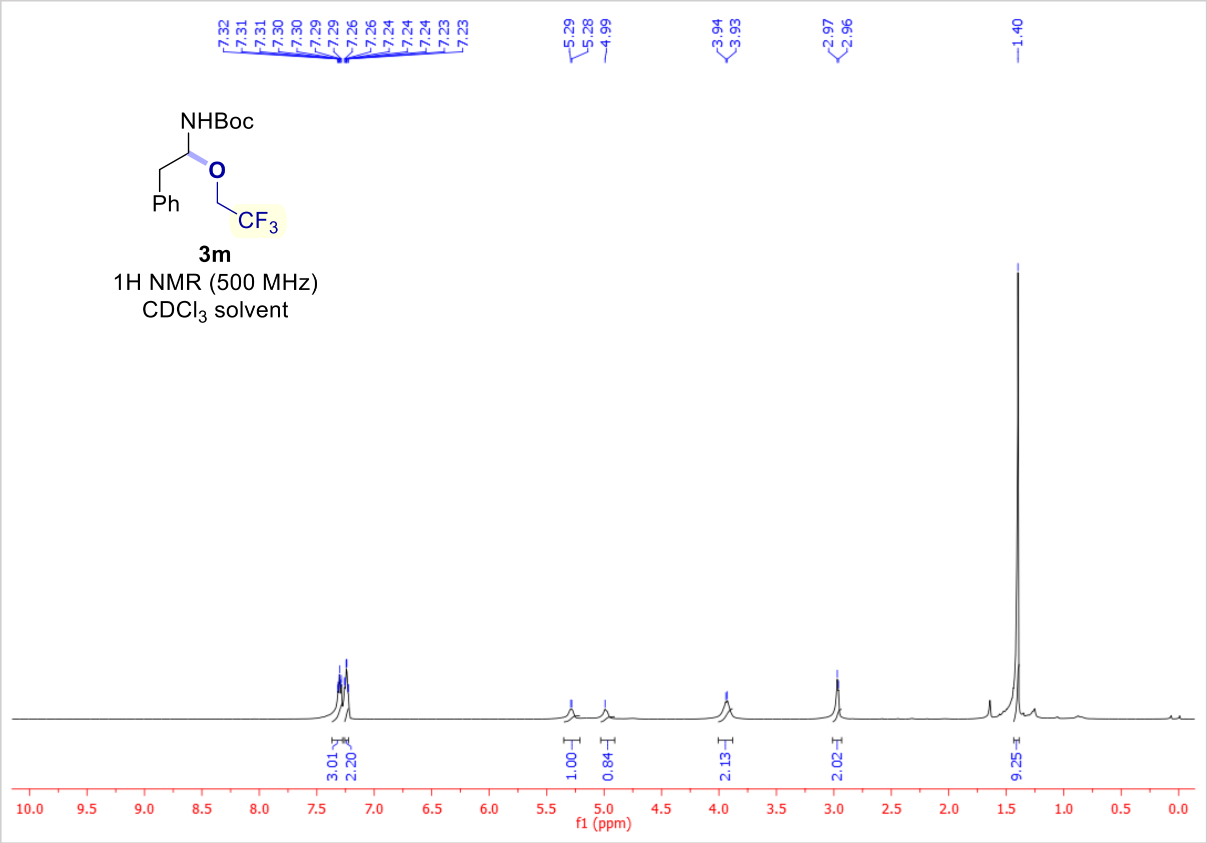
7.32
7.31
7.31
7.30
7.30
7.29
7.29
7.26
7.26
7.24
7.24
7.24
7.23
7.23
5.29
5.28
4.99
3.94
3.93
2.97
2.96
1.40
NHBoc
O
Ph
CF3
3m
1H NMR (500 MHz)
CDCl3 solvent
3.01
2.20
1.00
0.84
2.13
2.02
9.25
10.0
9.5
9.0
8.5
8.0
7.5
7.0
6.5
6.0
5.5
5.0
4.5
4.0
3.5
3.0
2.5
2.0
1.5
1.0
0.5
0.0
f1 (ppm)

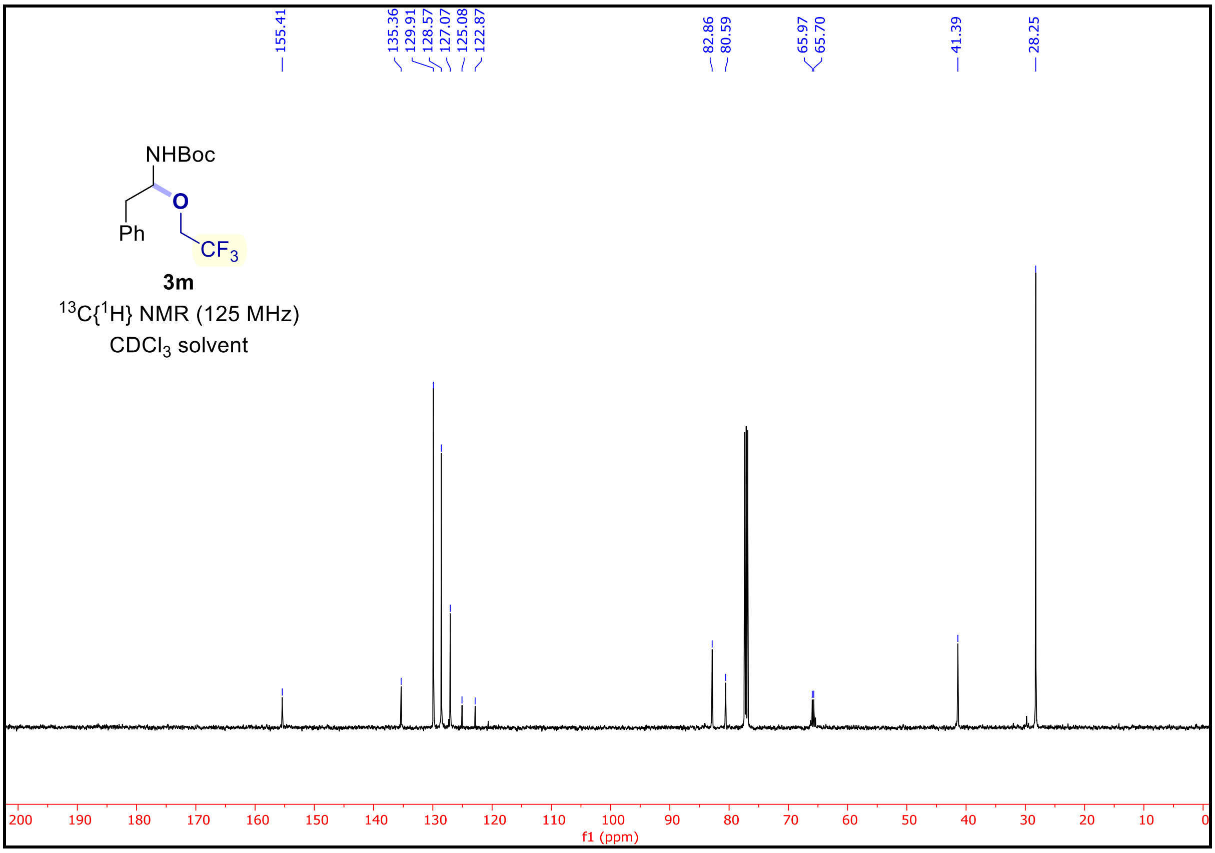
155.41
135.36
129.91
128.57
127.07
125.08
122.87
82.86
80.59
65.97
65.70
41.39
28.25
NHBoc
O
Ph
CF3
3m
13C{1H} NMR (125 MHz)
CDCl3 solvent
200
190
180
170
160
150
140
130
120
110
100
90
80
70
60
50
40
30
20
10
0
f1 (ppm)

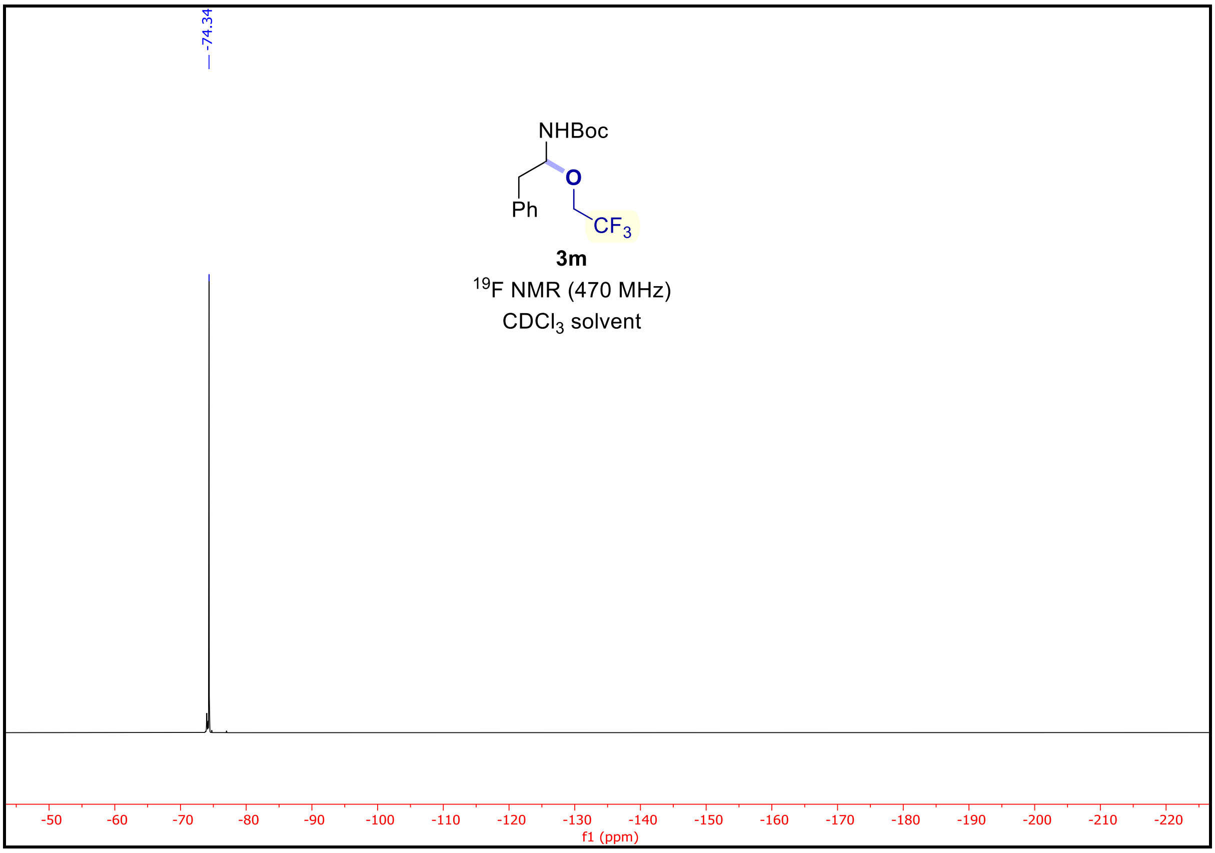

-74.34
NHBoc
O
Ph
CF3
3m
19F NMR (470 MHz)
CDCl3 solvent
f1 (ppm)

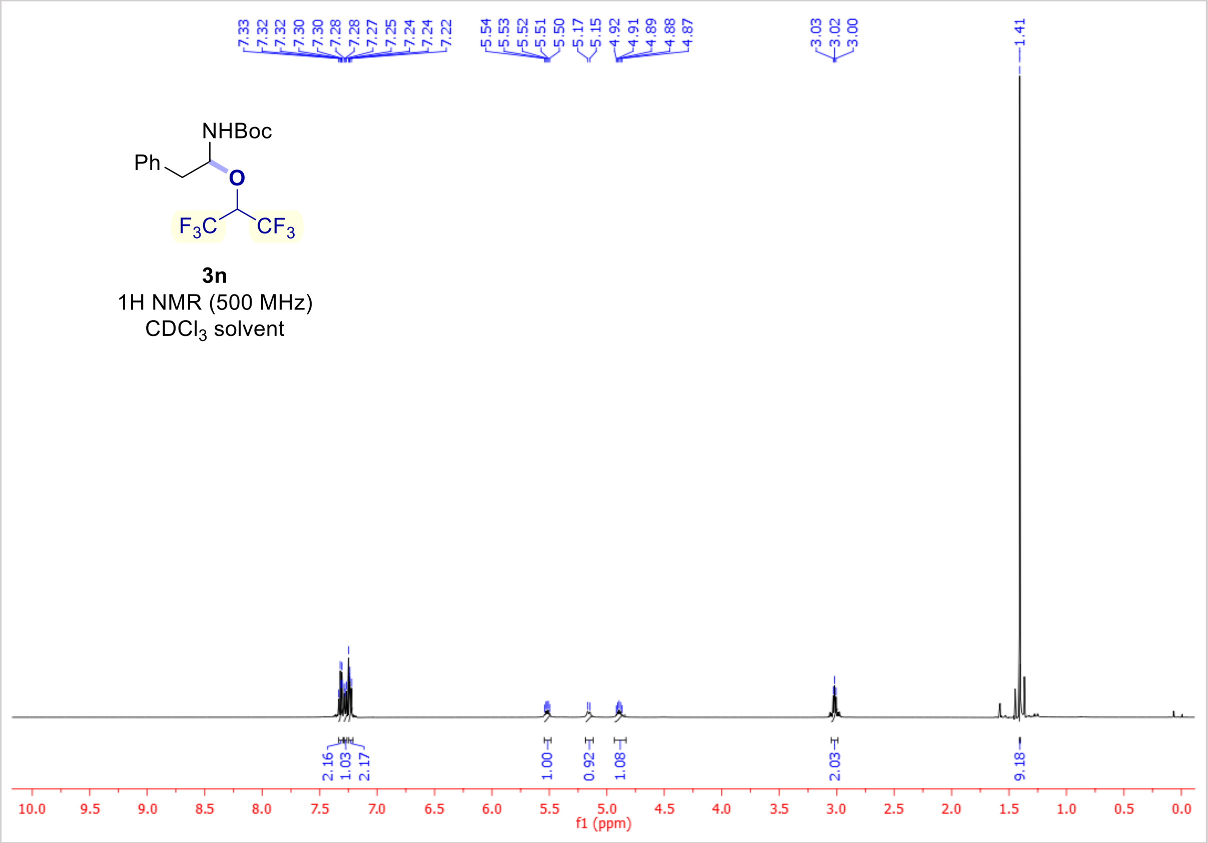

NHBoc
Ph
O
F3C
CF3
3n
1H NMR (500 MHz)
CDCl3 solvent
f1 (ppm)

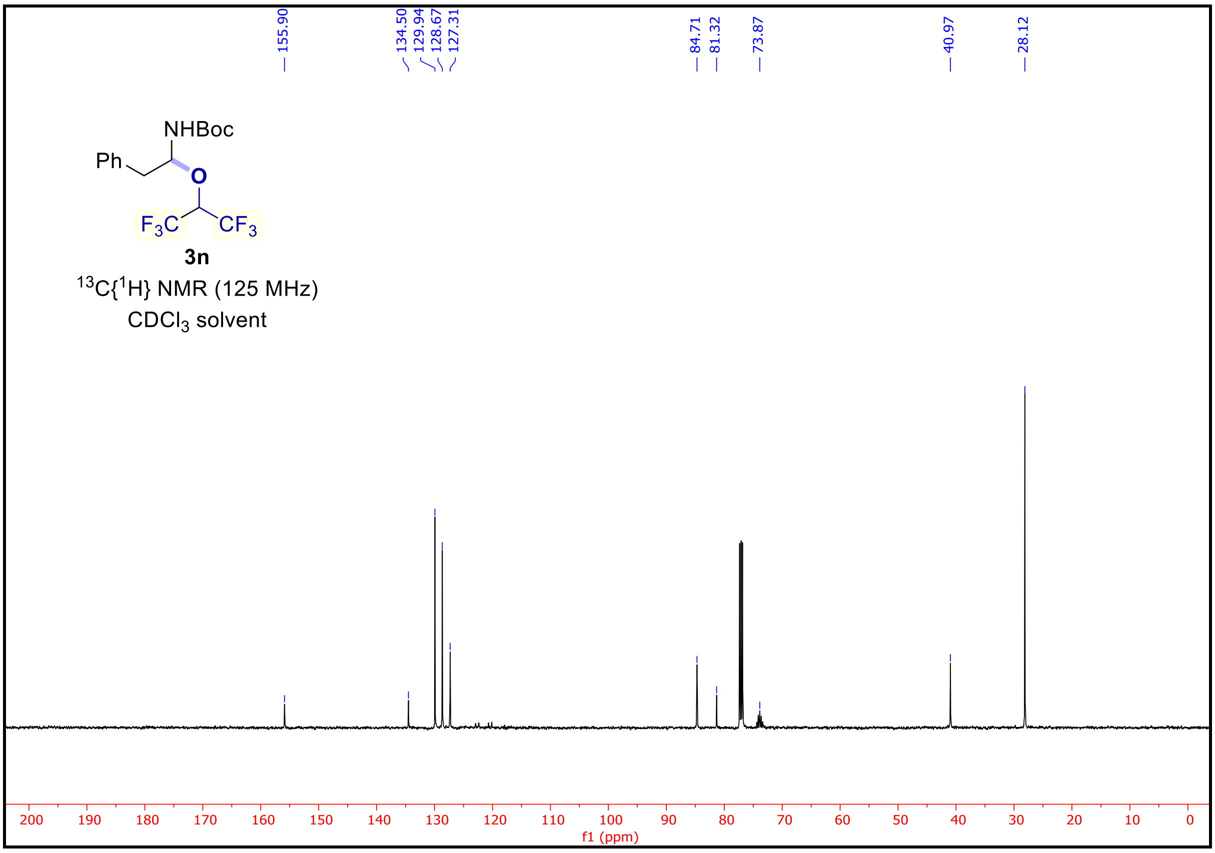
155.90
134.50
129.94
128.67
127.31
84.71
81.32
73.87
40.97
28.12
NHBoc
Ph
O
F3C
CF3
3n
13C{1H} NMR (125 MHz)
CDCl3 solvent
200
190
180
170
160
150
140
130
120
110
100
90
80
70
60
50
40
30
20
10
0
f1 (ppm)

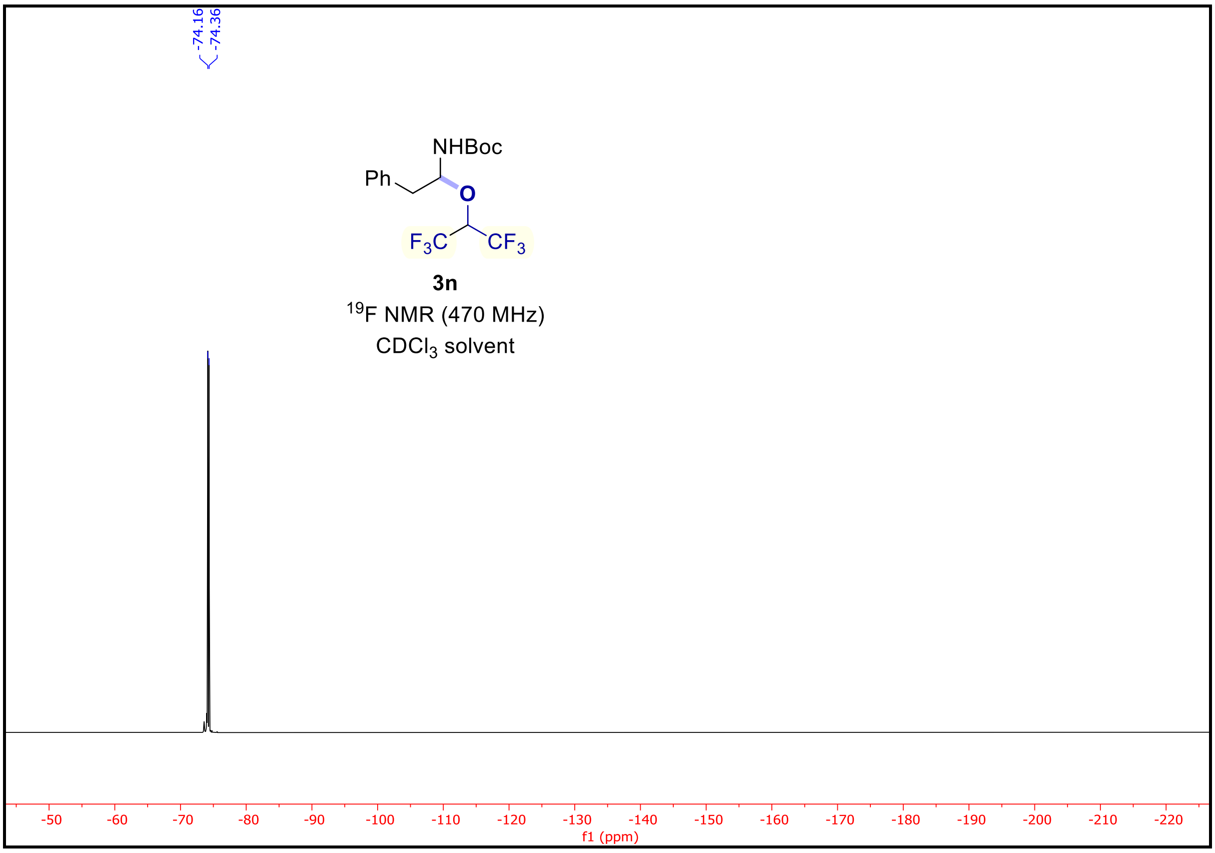
-74.16
-74.36
NHBoc
Ph
O
F3C
CF3
3n
19F NMR (470 MHz)
CDCl3 solvent
-50
-60
-70
-80
-90
-100
-110
-120
-130
-140
-150
-160
-170
-180
-190
-200
-210
-220
f1 (ppm)

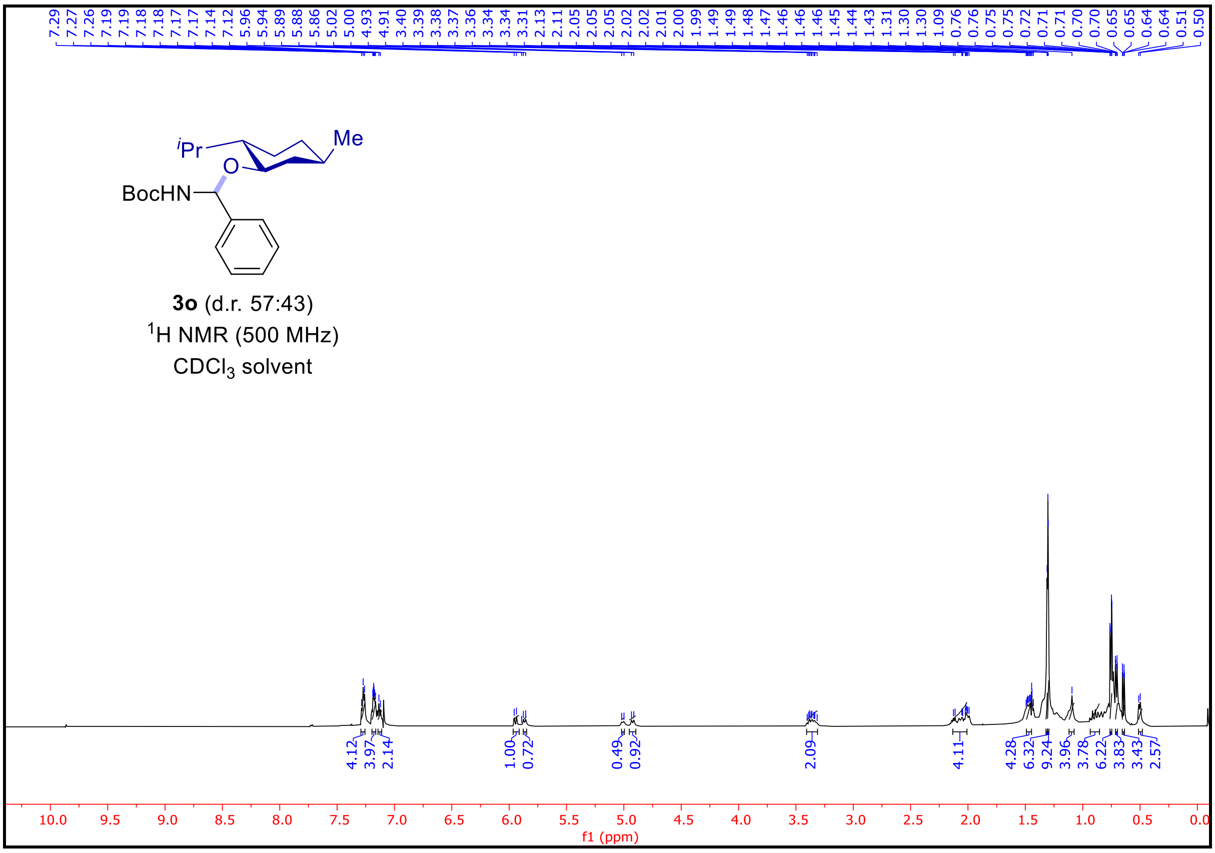

iPr
Me
O
BocHN
3o (d.r. 57:43)
1H NMR (500 MHz)
CDCl3 solvent
f1 (ppm)

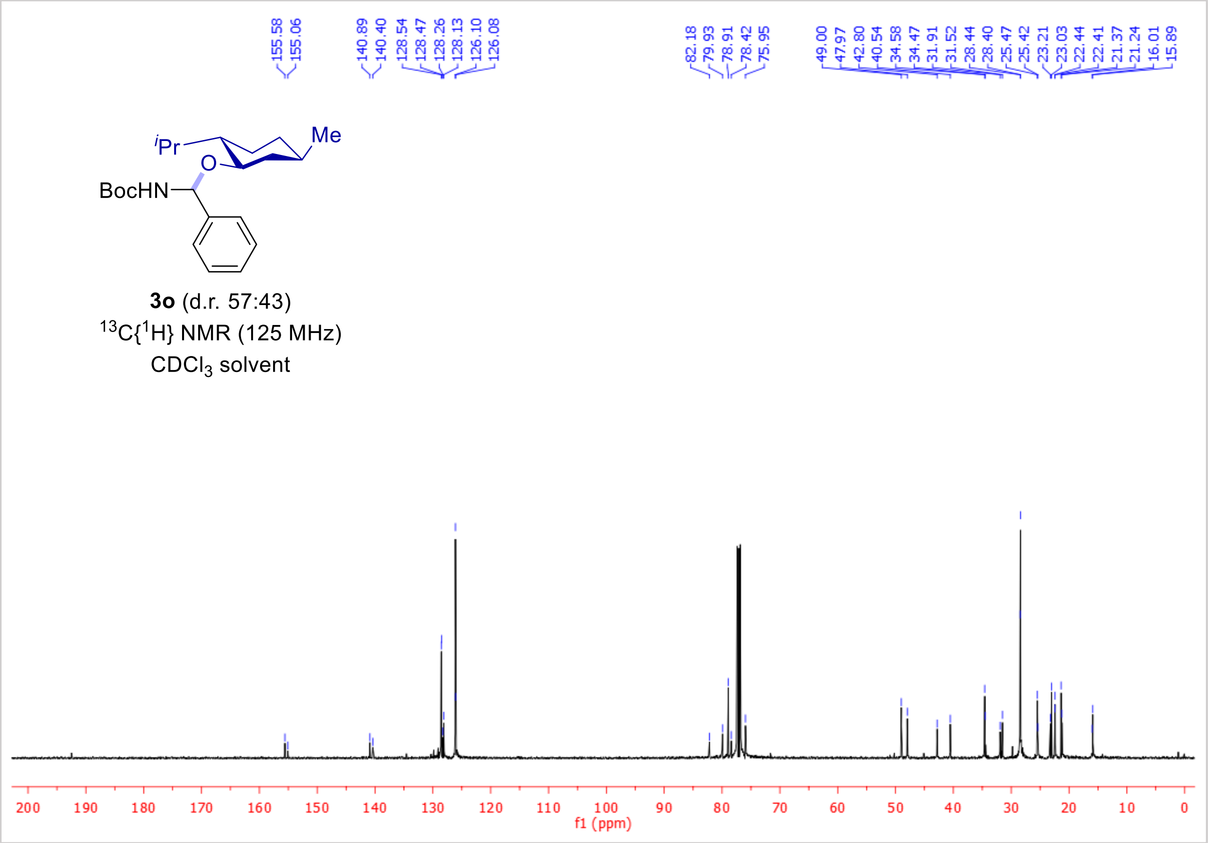

iPr
Me
O
BocHN
3o (d.r. 57:43)
13C{1H} NMR (125 MHz)
CDCl3 solvent
f1 (ppm)

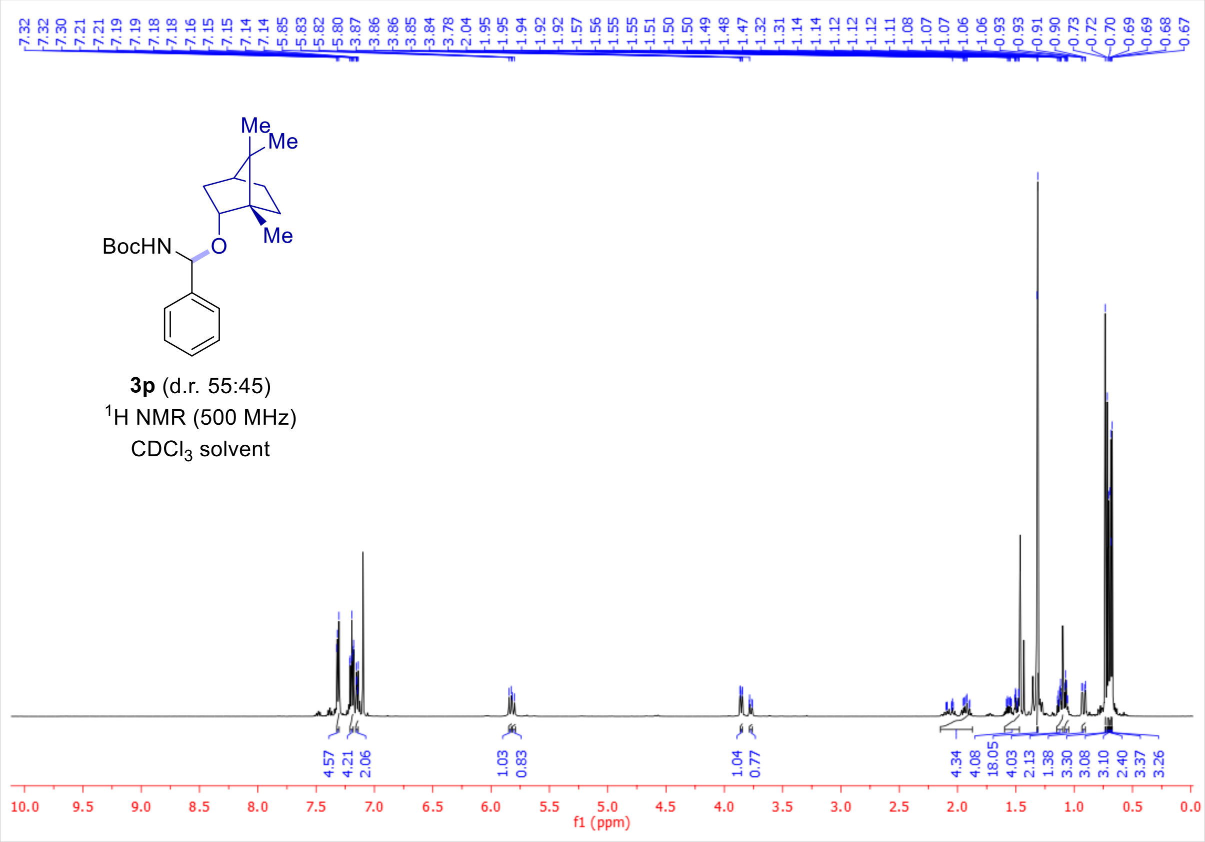

3p (d.r. 55:45)
^{1}H NMR (500 MHz)
$CDCl_3$ solvent

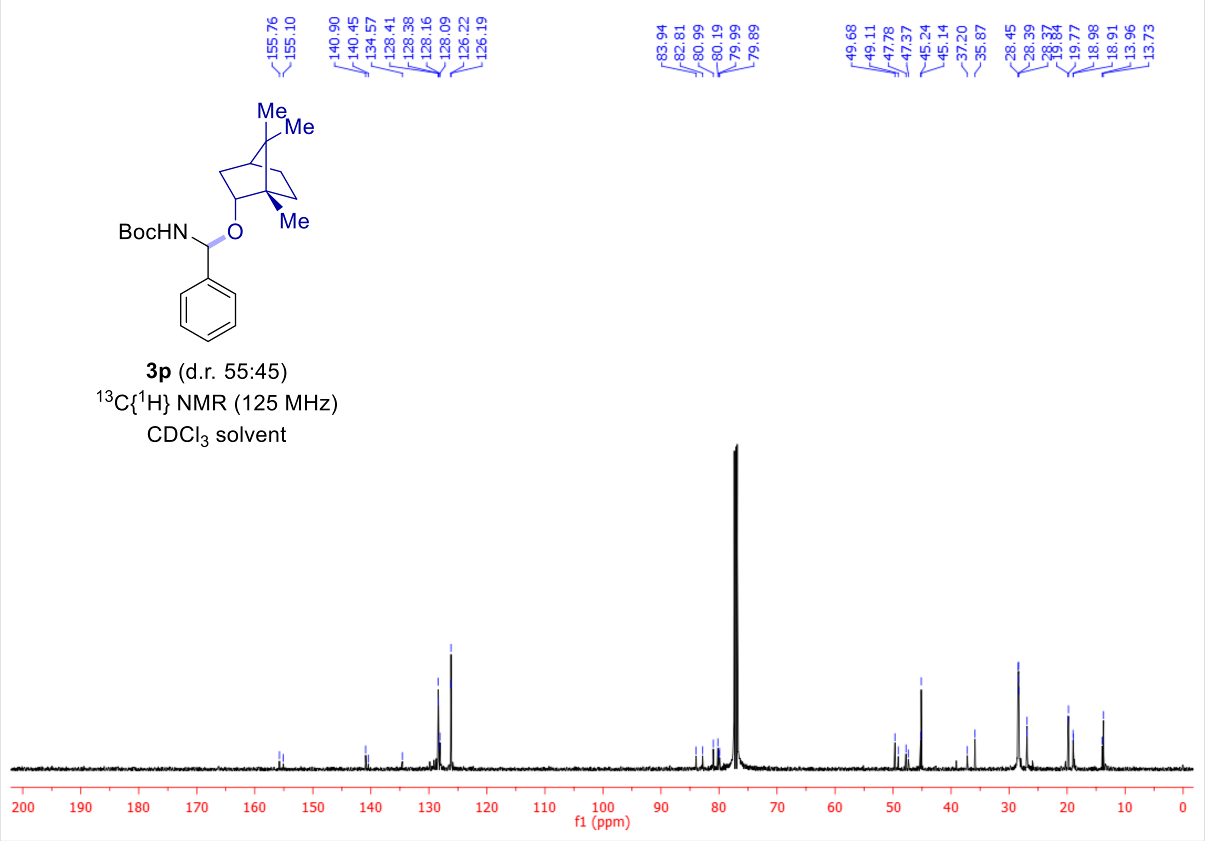

3p (d.r. 55:45)
^{13}C{^{1}H} NMR (125 MHz)
$CDCl_3$ solvent

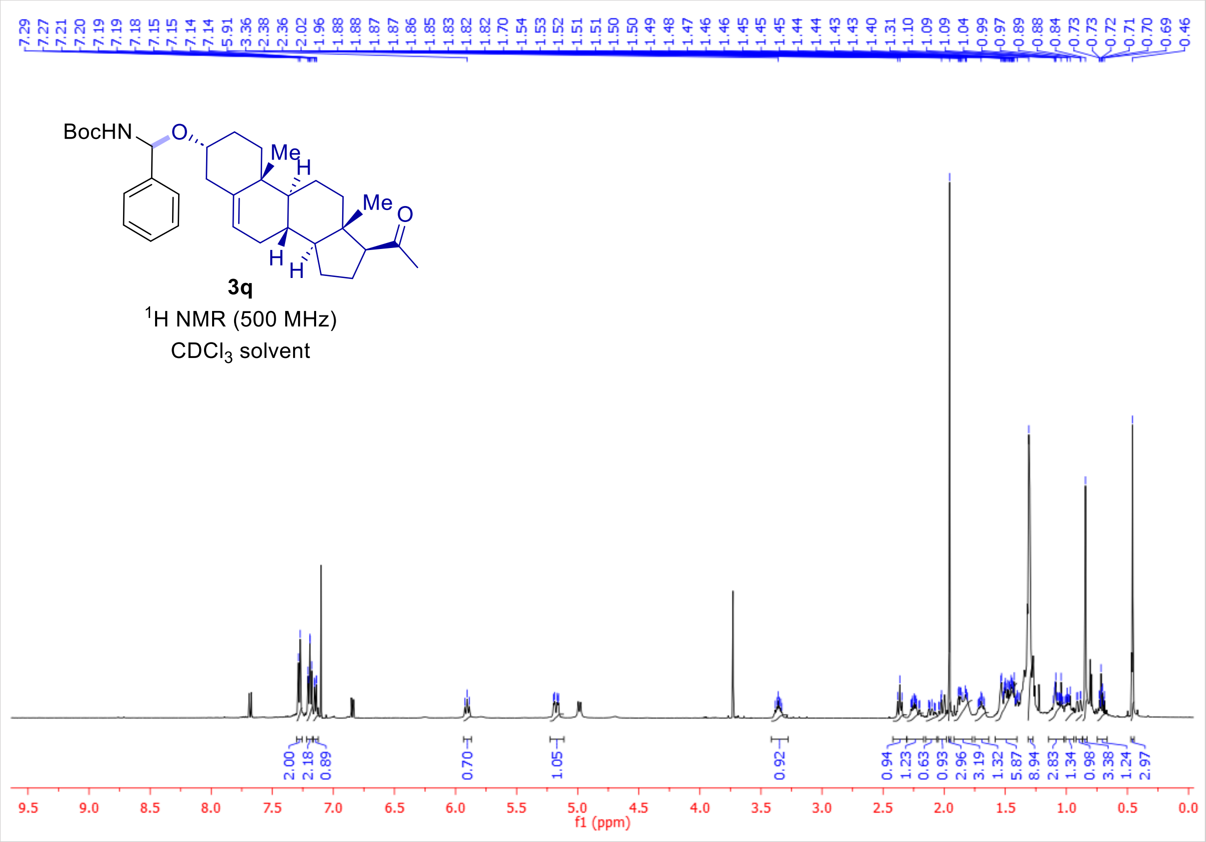
BocHN
O
Me
H
Me
O
H
H
3q
^{1}H NMR (500 MHz)
$CDCl_3$ solvent
f1 (ppm)

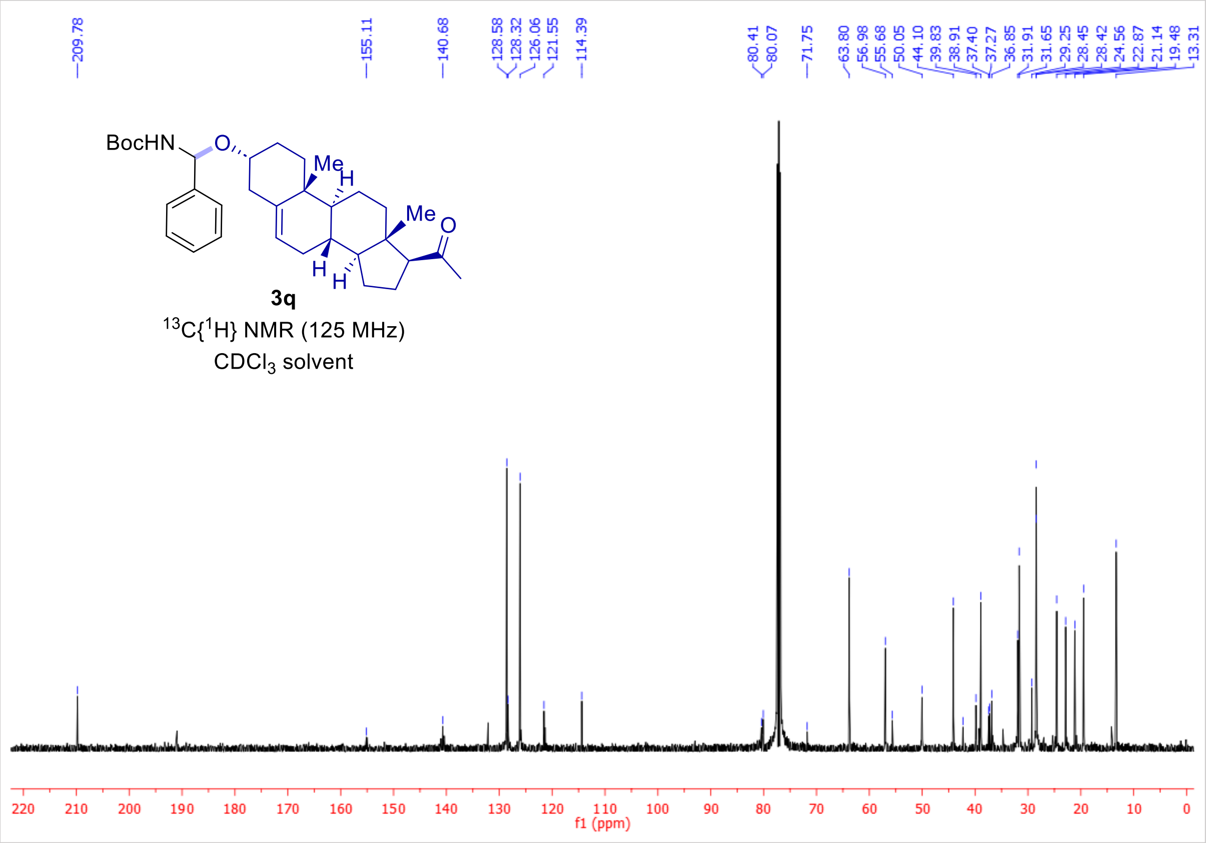
BocHN
O
Me
H
Me
O
H
H
3q
$^{13}C\{^1H\}$ NMR (125 MHz)
$CDCl_3$ solvent
f1 (ppm)

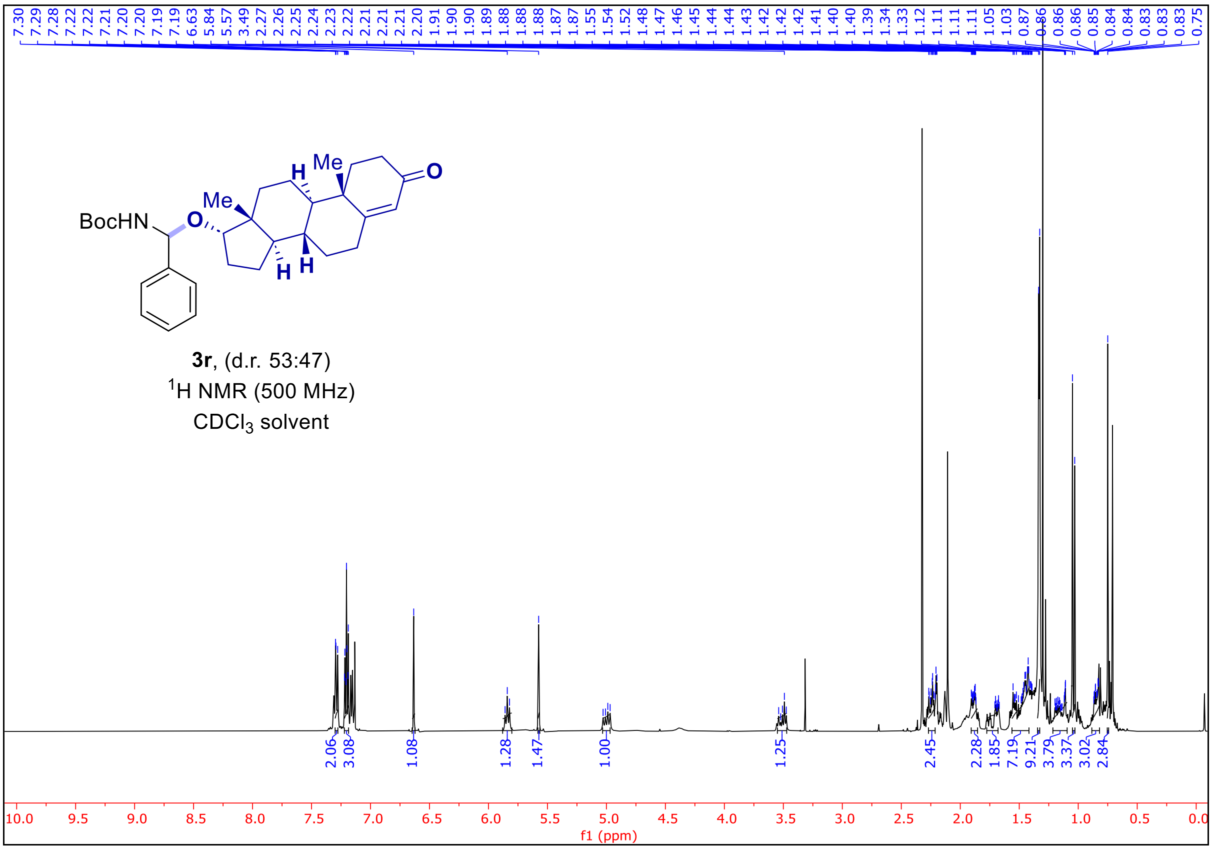
7.30 7.29 7.28 7.22 7.22 7.21 7.20 7.20 7.19 7.19 6.63 5.84 5.57 3.49 2.27 2.26 2.25 2.24 2.23 2.22 2.21 2.21 2.21 2.20 1.91 1.90 1.90 1.89 1.88 1.88 1.88 1.87 1.55 1.54 1.52 1.48 1.47 1.46 1.45 1.44 1.44 1.43 1.42 1.42 1.41 1.40 1.39 1.34 1.33 1.12 1.11 1.11 1.11 1.05 1.03 0.87 0.86 0.86 0.86 0.85 0.84 0.84 0.83 0.83 0.83 0.75
BocHN
Me
Me
H
H
H
O
O
3r, (d.r. 53:47)
^{1}H NMR (500 MHz)
$CDCl_3$ solvent
2.06 3.08 1.08 1.28 1.47 1.00 1.25 2.45 2.28 1.85 7.19 9.21 3.79 3.37 3.02 2.84
10.0 9.5 9.0 8.5 8.0 7.5 7.0 6.5 6.0 5.5 5.0 4.5 4.0 3.5 3.0 2.5 2.0 1.5 1.0 0.5 0.0
f1 (ppm)

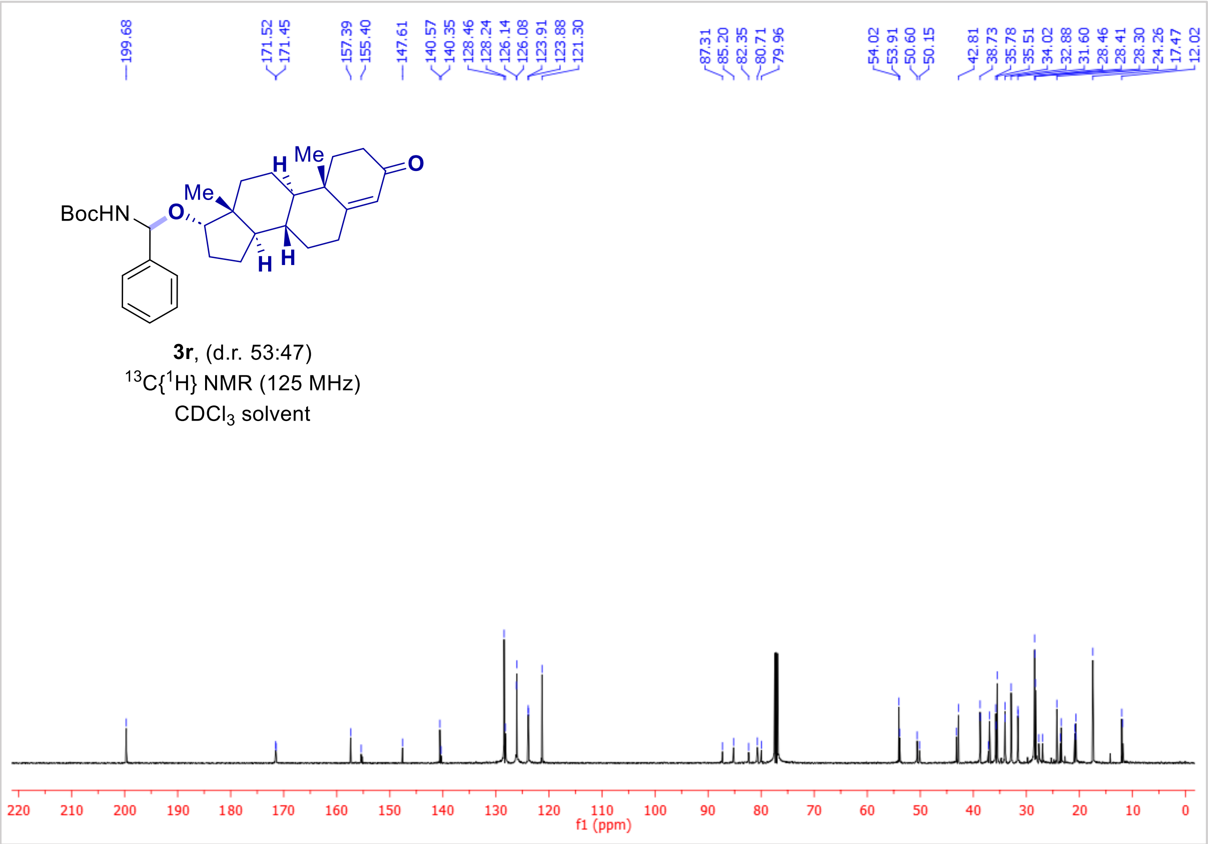
199.68 171.52 171.45 157.39 155.40 147.61 140.57 140.35 128.46 128.24 126.14 126.08 123.91 123.88 121.30 87.31 85.20 82.35 80.71 79.96 54.02 53.91 50.60 50.15 42.81 38.73 35.78 35.51 34.02 32.88 31.60 28.46 28.41 28.30 24.26 17.47 12.02
BocHN
Me
Me
H
H
H
O
O
3r, (d.r. 53:47)
^{13}C{^{1}H} NMR (125 MHz)
$CDCl_3$ solvent
220 210 200 190 180 170 160 150 140 130 120 110 100 90 80 70 60 50 40 30 20 10 0
f1 (ppm)

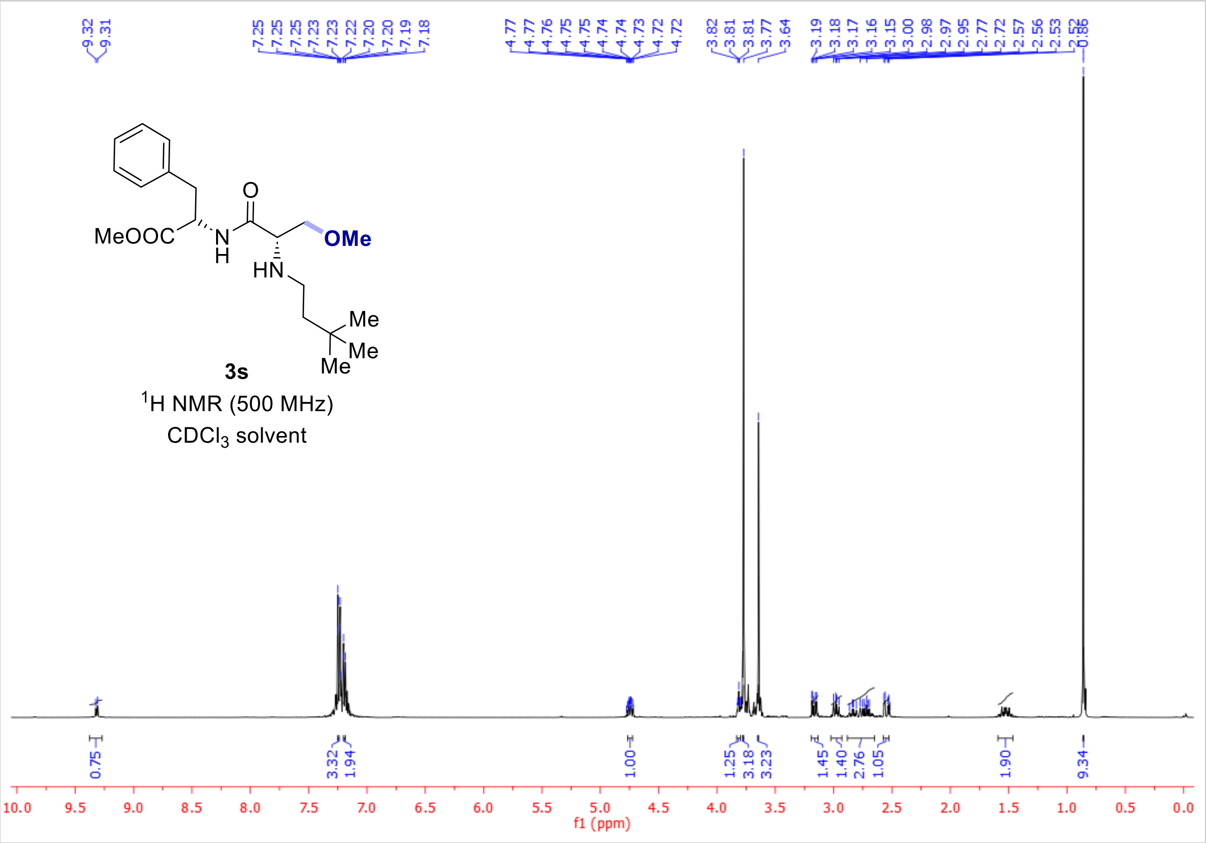
9.32
9.31
7.25
7.25
7.25
7.23
7.23
7.22
7.20
7.20
7.19
7.18
4.77
4.77
4.76
4.75
4.75
4.74
4.74
4.73
4.72
4.72
3.82
3.81
3.81
3.77
3.64
3.19
3.18
3.17
3.16
3.15
3.00
2.98
2.97
2.95
2.77
2.72
2.57
2.56
2.53
2.52
0.86
MeOOC
N
H
O
OMe
HN
Me
Me
Me
3s
^{1}H NMR (500 MHz)
CDCl$_3$ solvent
0.75
3.32
1.94
1.00
1.25
3.18
3.23
1.45
1.40
2.76
1.05
1.90
9.34
f1 (ppm)

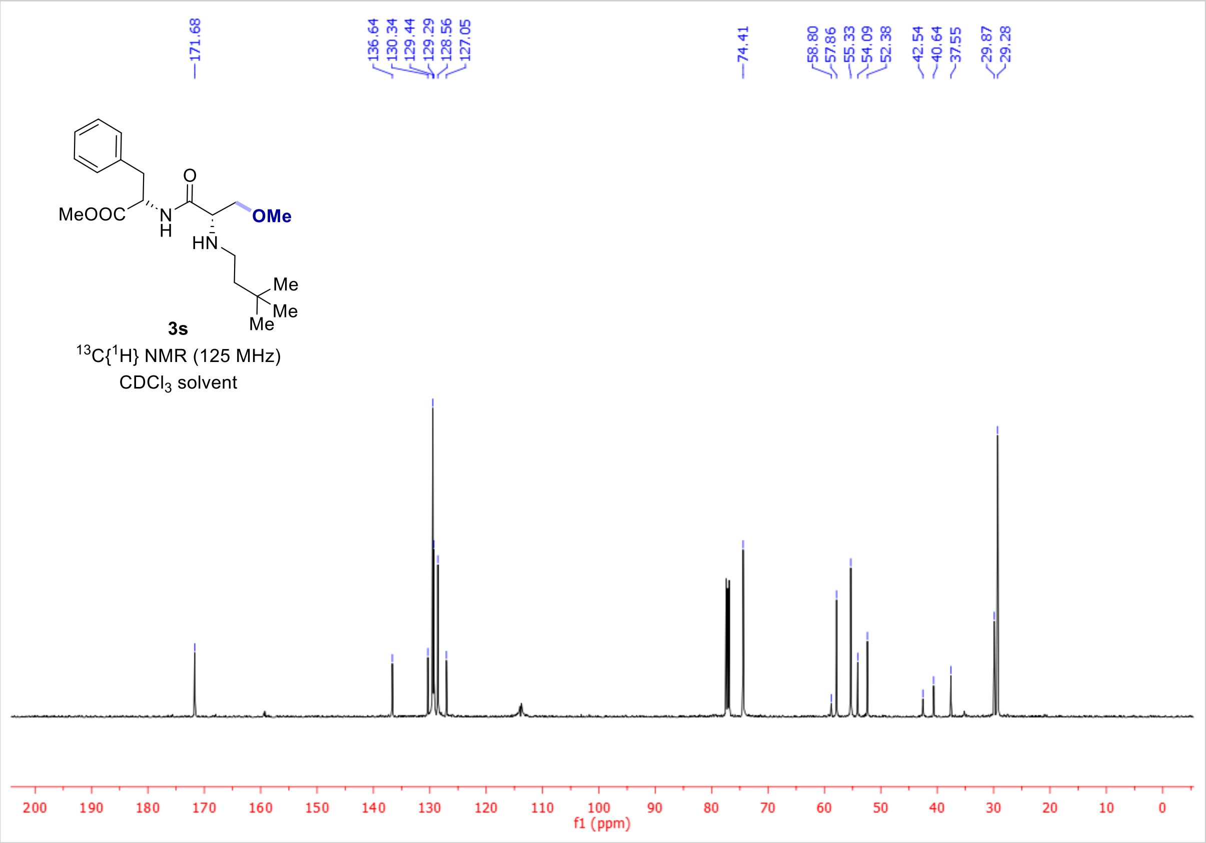
171.68
136.64
130.34
129.44
129.29
128.56
127.05
74.41
58.80
57.86
55.33
54.09
52.38
42.54
40.64
37.55
29.87
29.28
MeOOC
N
H
O
OMe
HN
Me
Me
Me
3s
^{13}C{^{1}H} NMR (125 MHz)
CDCl$_3$ solvent
f1 (ppm)

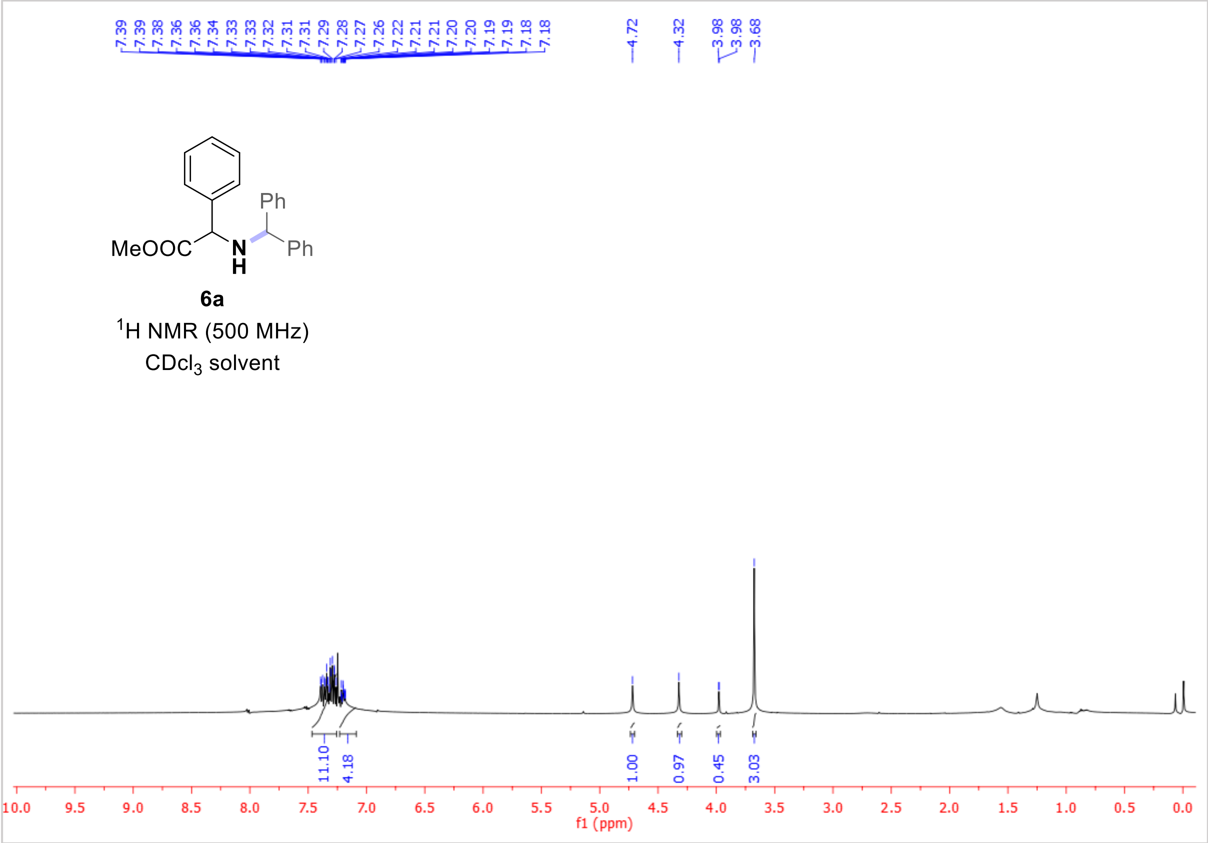
7.39 7.39 7.38 7.36 7.36 7.34 7.33 7.33 7.32 7.31 7.31 7.29 7.28 7.27 7.26 7.22 7.21 7.21 7.20 7.20 7.19 7.19 7.18 7.18
4.72
4.32
3.98 3.98
3.68
Ph
MeOOC
N
H
Ph
6a
1H NMR (500 MHz)
CDcl3 solvent
11.10 4.18 1.00 0.97 0.45 3.03
10.0 9.5 9.0 8.5 8.0 7.5 7.0 6.5 6.0 5.5 5.0 4.5 4.0 3.5 3.0 2.5 2.0 1.5 1.0 0.5 0.0
f1 (ppm)

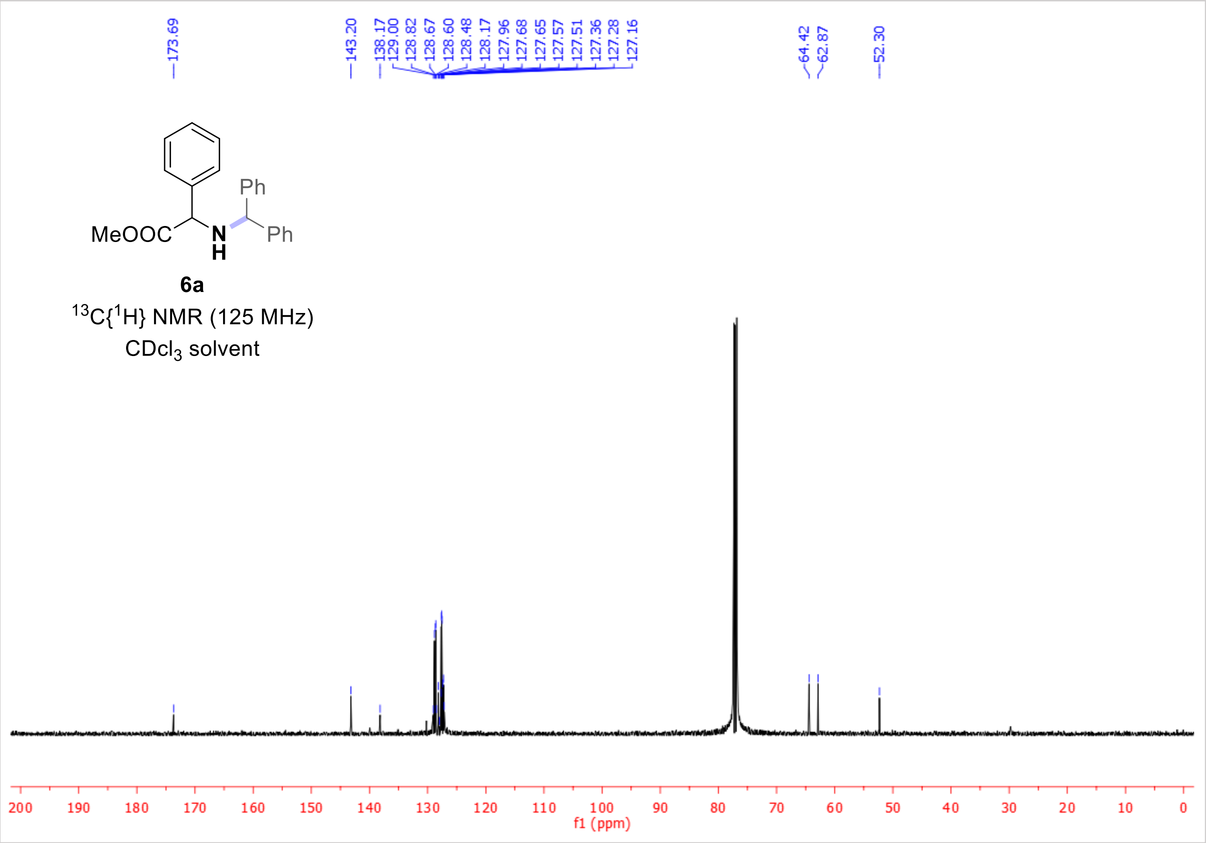
173.69
143.20
138.17 129.00 128.82 128.67 128.60 128.48 128.17 127.96 127.68 127.65 127.57 127.51 127.36 127.28 127.16
64.42 62.87
52.30
Ph
MeOOC
N
H
Ph
6a
13C{1H} NMR (125 MHz)
CDcl3 solvent
200 190 180 170 160 150 140 130 120 110 100 90 80 70 60 50 40 30 20 10 0
f1 (ppm)

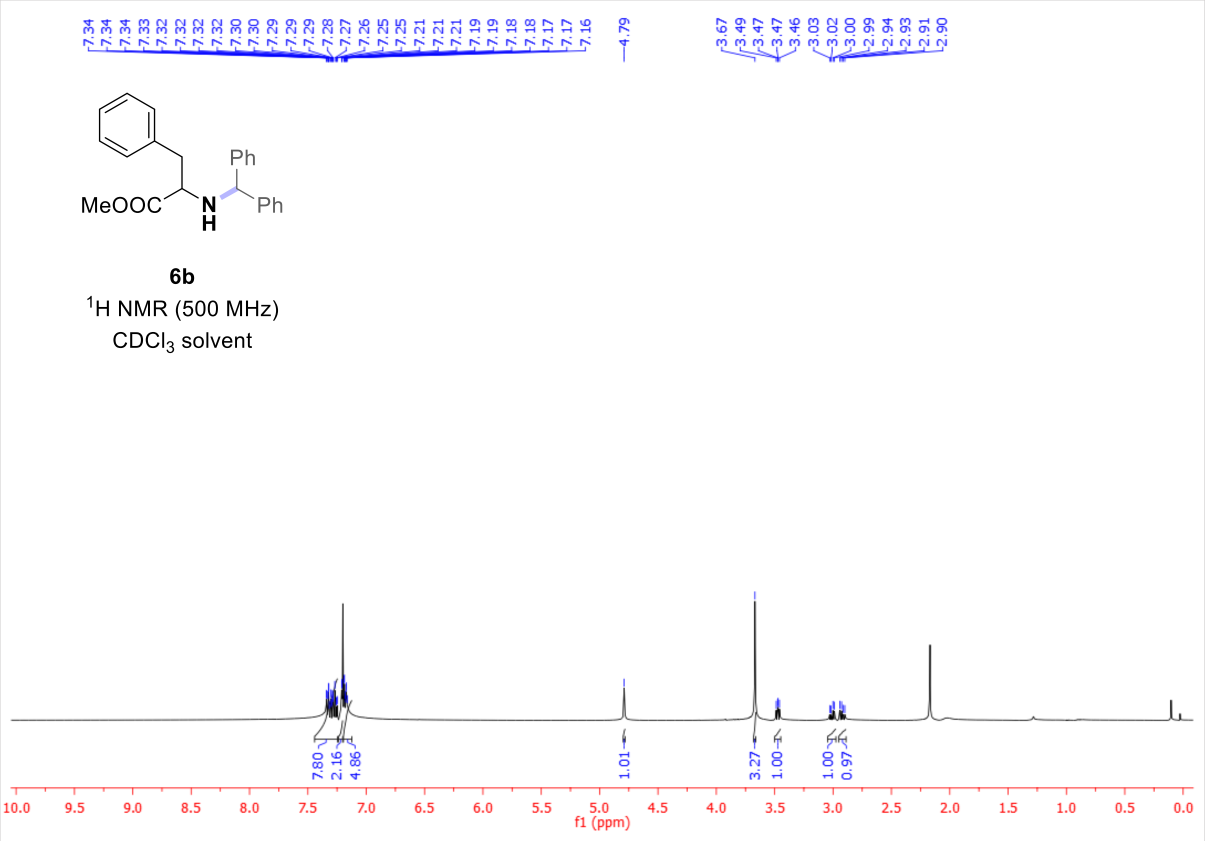
7.34 7.34 7.34 7.33 7.32 7.32 7.32 7.32 7.30 7.30 7.29 7.29 7.29 7.28 7.27 7.26 7.25 7.25 7.21 7.21 7.21 7.19 7.19 7.18 7.18 7.17 7.17 7.16
4.79
3.67 3.49 3.47 3.47 3.46 3.03 3.02 3.00 2.99 2.94 2.93 2.91 2.90
Ph
MeOOC
N
H
Ph
6b
1H NMR (500 MHz)
CDCl3 solvent
7.80 2.16 4.86
1.01
3.27 1.00
1.00 0.97
f1 (ppm)

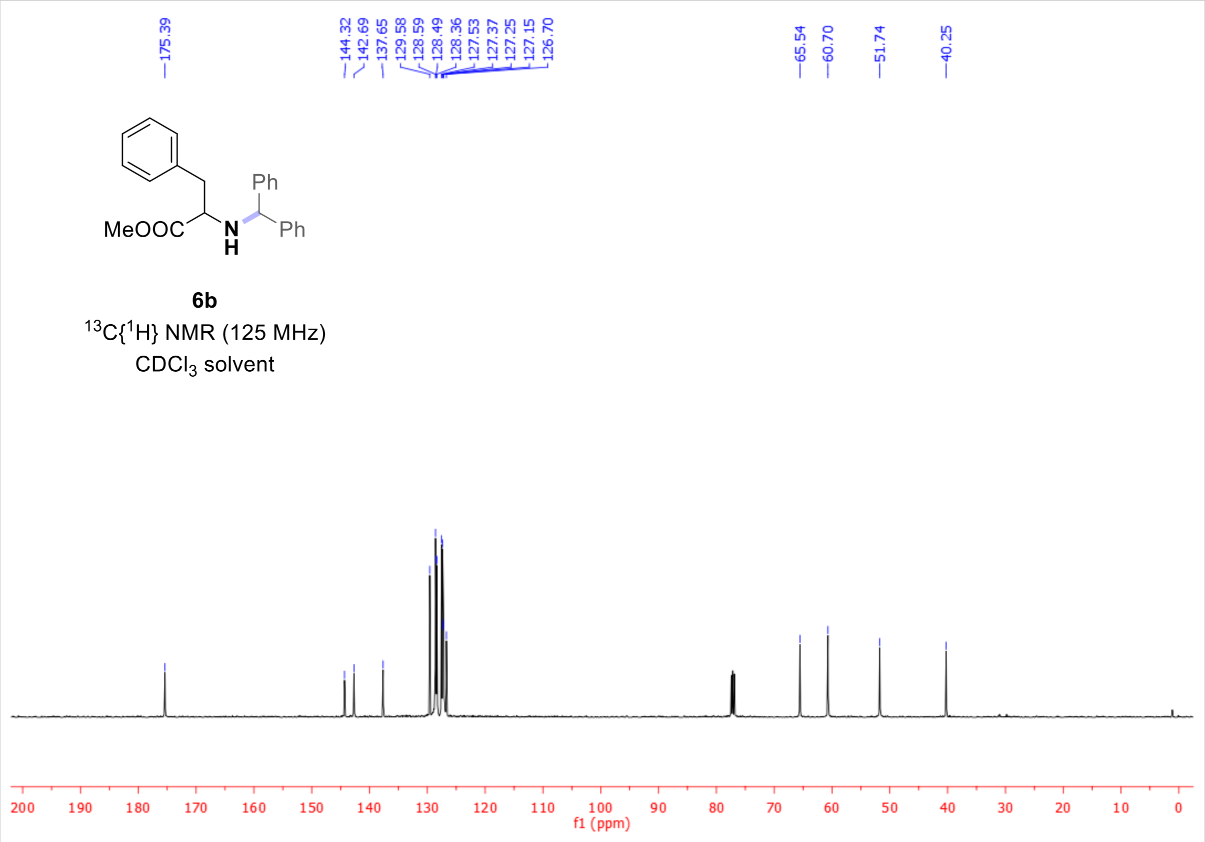
175.39
144.32 142.69 137.65 129.58 128.59 128.49 128.36 127.53 127.37 127.25 127.15 126.70
65.54 60.70 51.74 40.25
Ph
MeOOC
N
H
Ph
6b
13C{1H} NMR (125 MHz)
CDCl3 solvent
f1 (ppm)

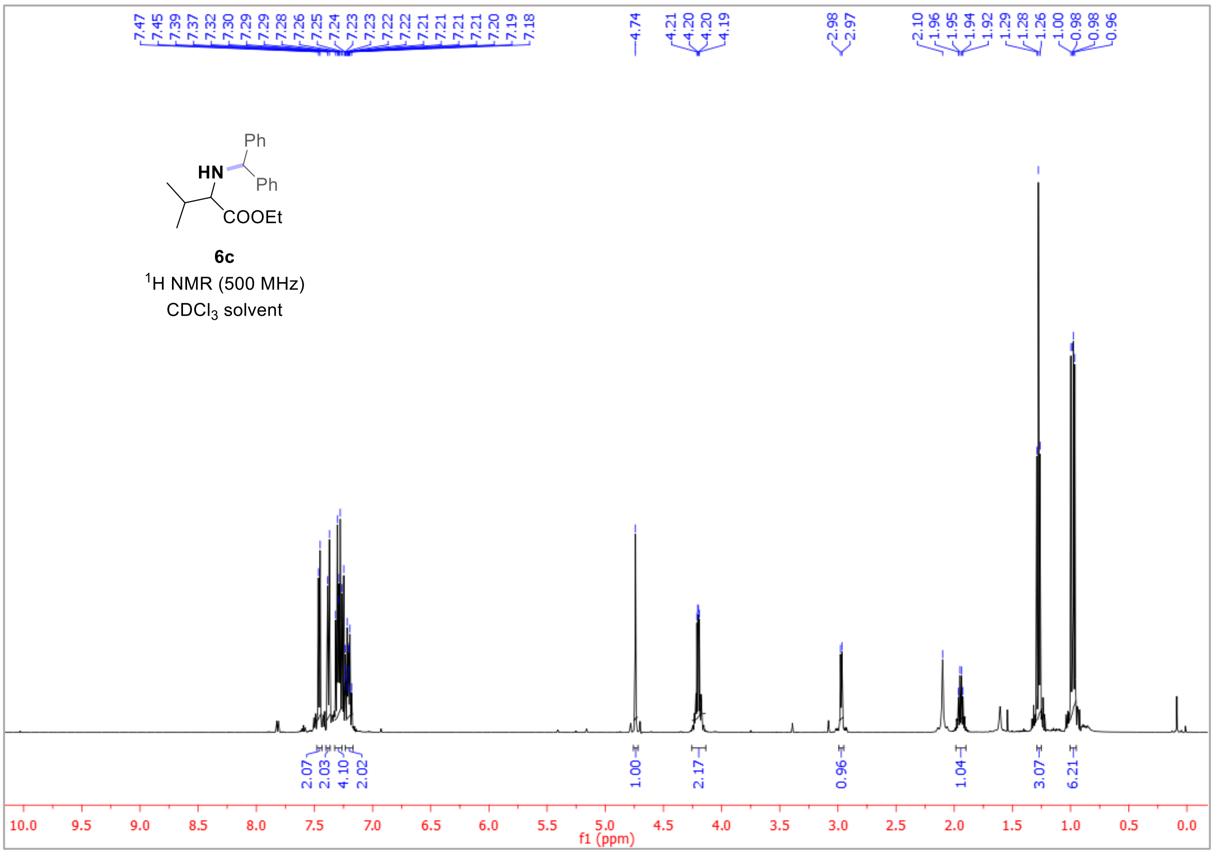
7.47 7.45 7.39 7.37 7.32 7.30 7.29 7.29 7.28 7.26 7.25 7.24 7.23 7.23 7.22 7.22 7.21 7.21 7.21 7.21 7.20 7.19 7.18
4.74
4.21 4.20 4.20 4.19
2.98 2.97
2.10 1.96 1.95 1.94 1.92 1.29 1.28 1.26 1.00 0.98 0.98 0.96
Ph
HN
Ph
COOEt
6c
1H NMR (500 MHz)
CDCl3 solvent
2.07 2.03 4.10 2.02
1.00
2.17
0.96
1.04
3.07
6.21
10.0 9.5 9.0 8.5 8.0 7.5 7.0 6.5 6.0 5.5 5.0 4.5 4.0 3.5 3.0 2.5 2.0 1.5 1.0 0.5 0.0
f1 (ppm)

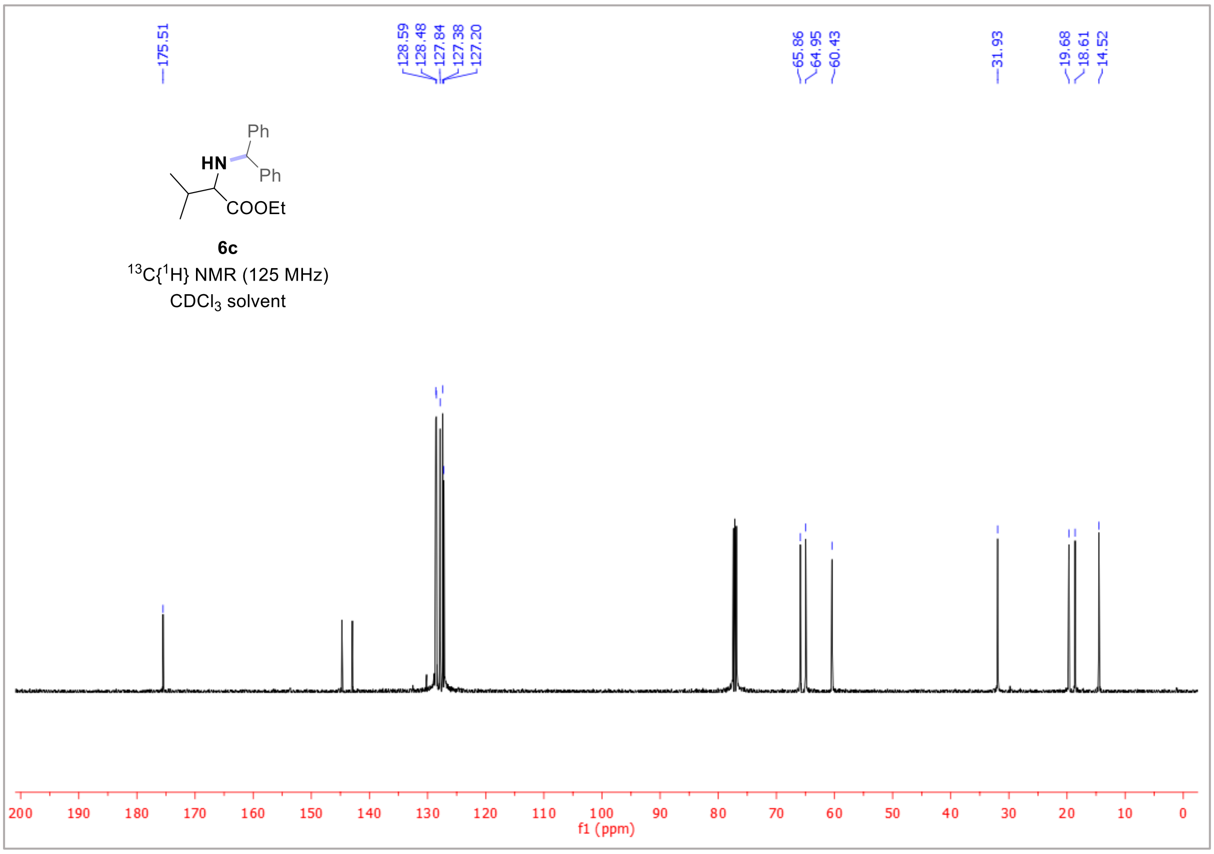
175.51
128.59 128.48 127.84 127.38 127.20
65.86 64.95 60.43
31.93
19.68 18.61 14.52
Ph
HN
Ph
COOEt
6c
13C{1H} NMR (125 MHz)
CDCl3 solvent
200 190 180 170 160 150 140 130 120 110 100 90 80 70 60 50 40 30 20 10 0
f1 (ppm)

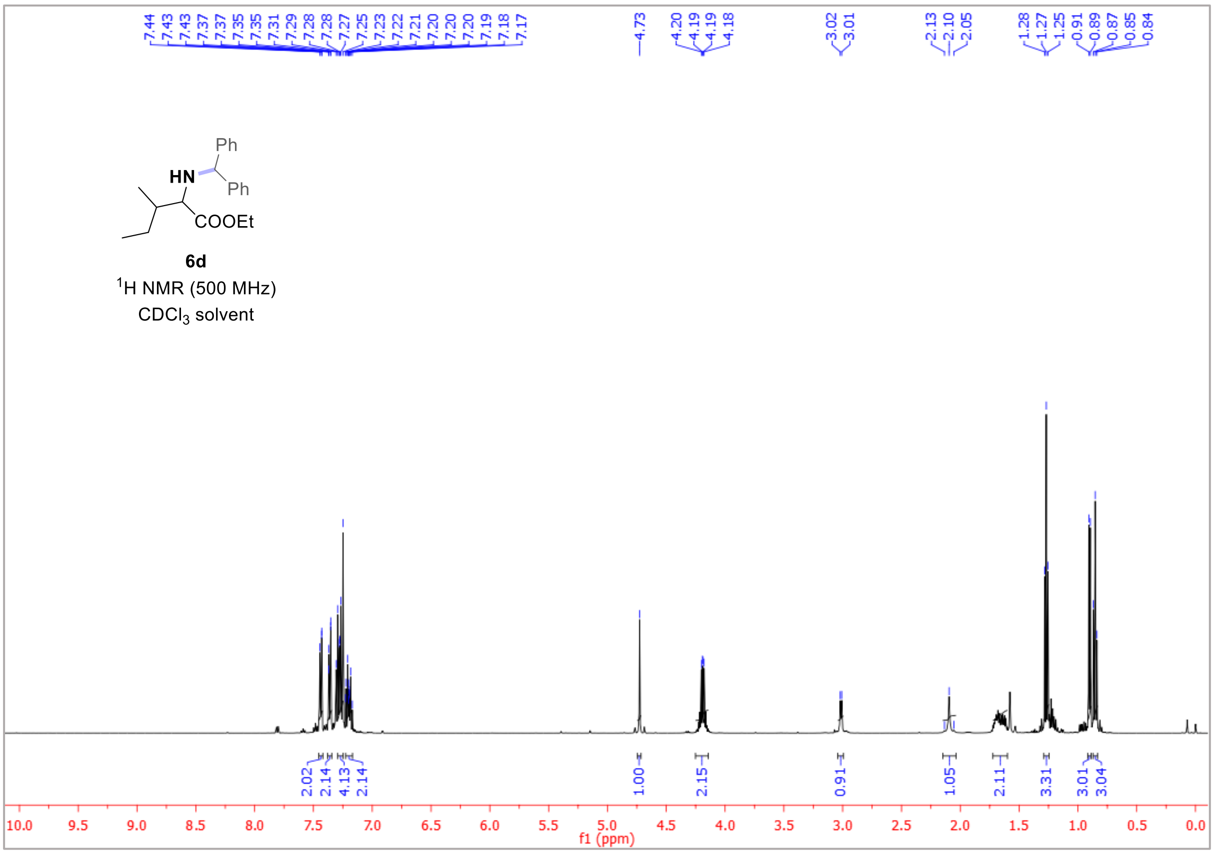

7.44 7.43 7.43 7.37 7.37 7.35 7.35 7.31 7.29 7.28 7.28 7.27 7.25 7.23 7.22 7.21 7.20 7.20 7.20 7.19 7.18 7.17
4.73
4.20 4.19 4.19 4.18
3.02 3.01
2.13 2.10 2.05
1.28 1.27 1.25 0.91 0.89 0.87 0.85 0.84
Ph
HN
Ph
COOEt
6d
1H NMR (500 MHz)
CDCl3 solvent
2.02 2.14 4.13 2.14 1.00 2.15 0.91 1.05 2.11 3.31 3.01 3.04
f1 (ppm)

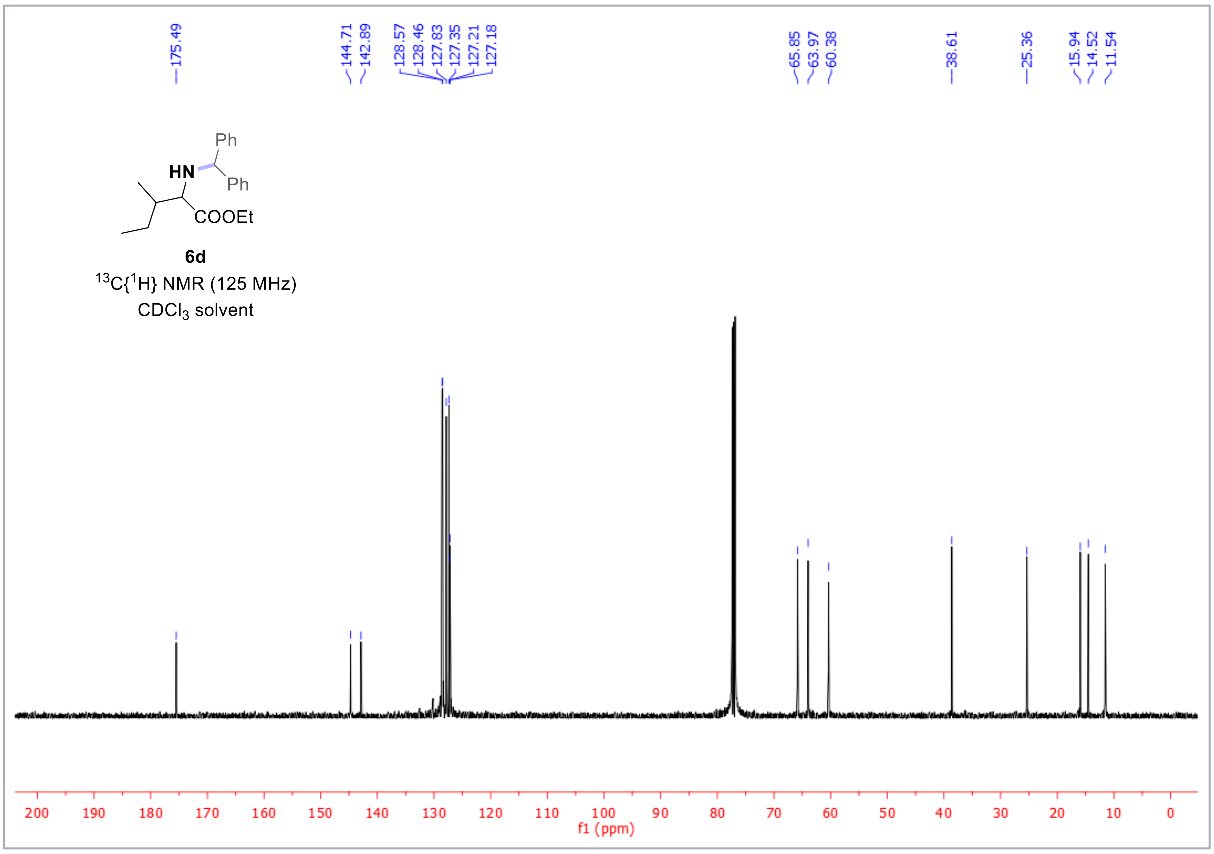

175.49
144.71 142.89
128.57 128.46 127.83 127.35 127.21 127.18
65.85 63.97 60.38
38.61
25.36
15.94 14.52 11.54
Ph
HN
Ph
COOEt
6d
13C{1H} NMR (125 MHz)
CDCl3 solvent
f1 (ppm)

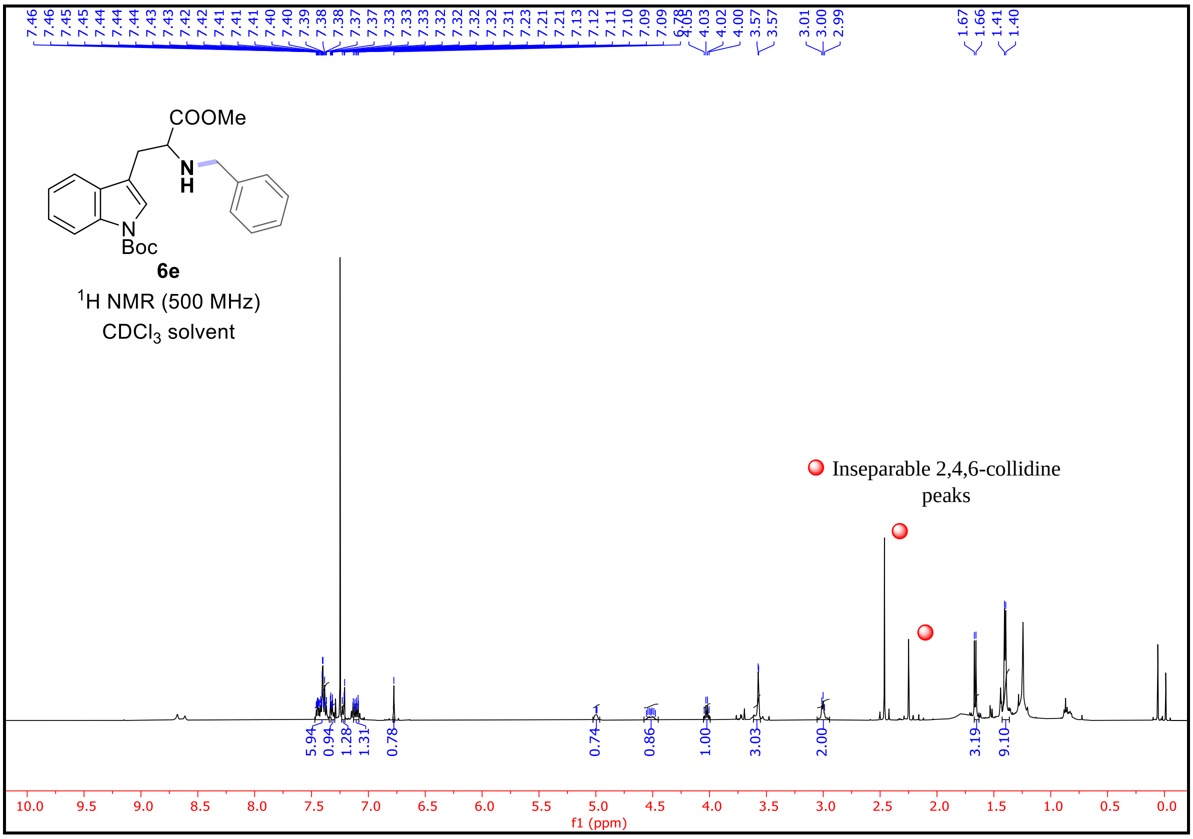

7.46
7.46
7.45
7.45
7.44
7.44
7.44
7.43
7.43
7.43
7.42
7.42
7.41
7.41
7.41
7.41
7.40
7.40
7.39
7.38
7.38
7.37
7.37
7.33
7.33
7.33
7.32
7.32
7.32
7.32
7.31
7.23
7.21
7.21
7.13
7.12
7.11
7.10
7.09
7.09
6.78
4.05
4.03
4.02
4.00
3.57
3.57
3.01
3.00
2.99
1.67
1.66
1.41
1.40
COOMe
N
H
N
Boc
6e
1H NMR (500 MHz)
CDCl3 solvent
Inseparable 2,4,6-collidine peaks
5.94
0.94
1.28
1.31
0.78
0.74
0.86
1.00
3.03
2.00
3.19
9.10
10.0
9.5
9.0
8.5
8.0
7.5
7.0
6.5
6.0
5.5
5.0
4.5
4.0
3.5
3.0
2.5
2.0
1.5
1.0
0.5
0.0
f1 (ppm)

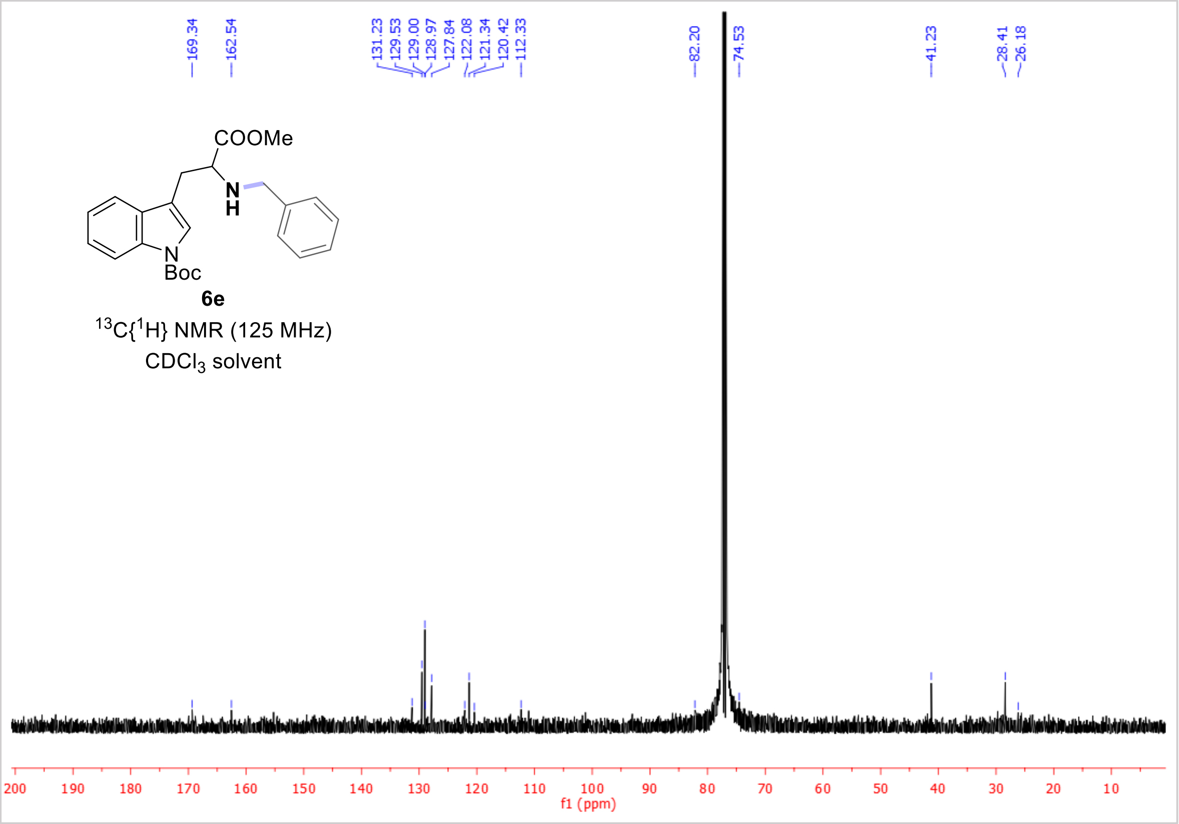

169.34
162.54
131.23
129.53
129.00
128.97
127.84
122.08
121.34
120.42
112.33
82.20
74.53
41.23
28.41
26.18
COOMe
N
H
N
Boc
6e
13C{1H} NMR (125 MHz)
CDCl3 solvent
200
190
180
170
160
150
140
130
120
110
100
90
80
70
60
50
40
30
20
10
f1 (ppm)

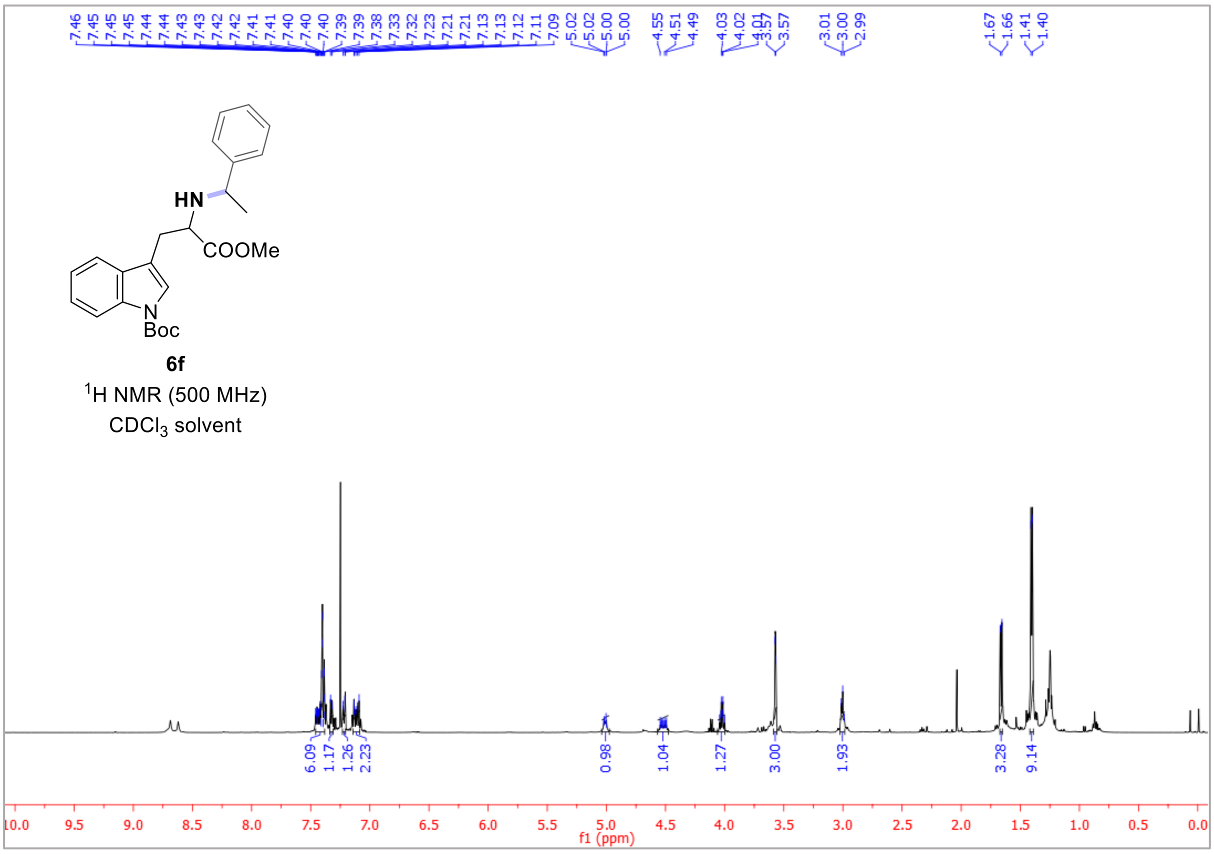
HN
COOMe
N
Boc
6f
^{1}H NMR (500 MHz)
$CDCl_3$ solvent
f1 (ppm)

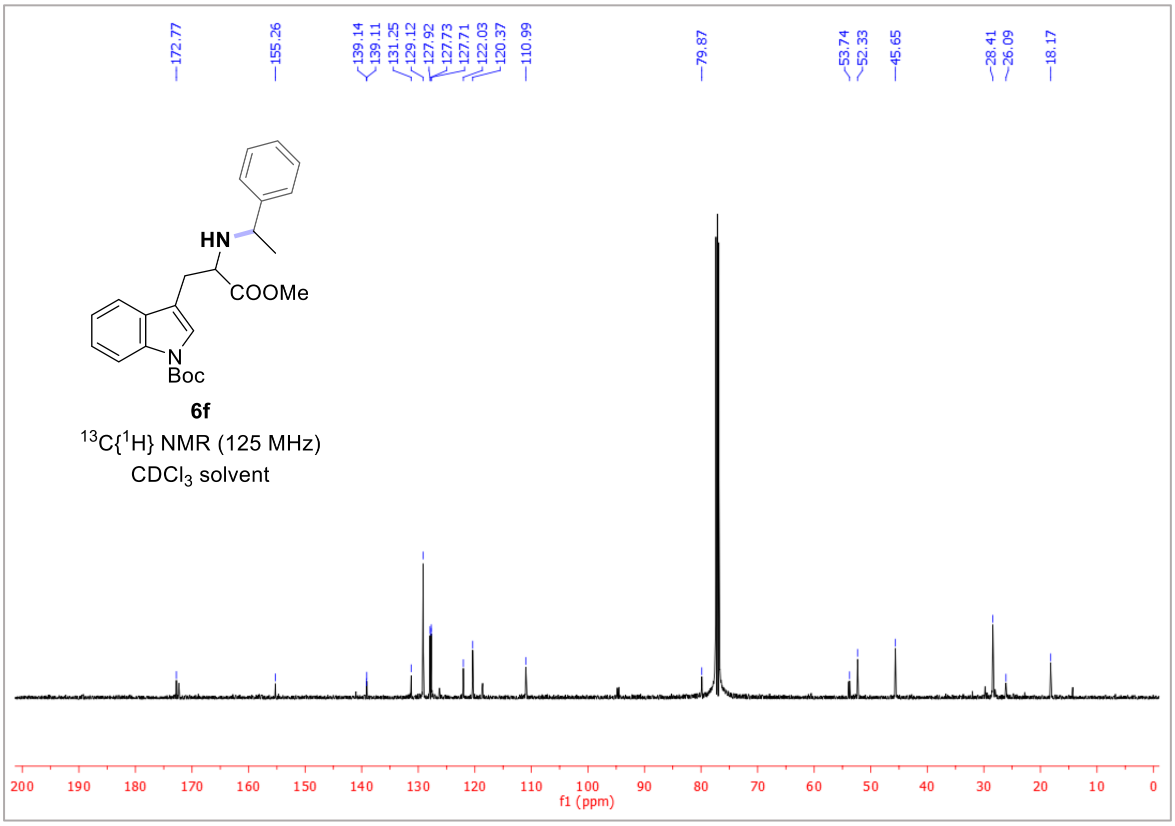
HN
COOMe
N
Boc
6f
^{13}C{^{1}H} NMR (125 MHz)
$CDCl_3$ solvent
f1 (ppm)

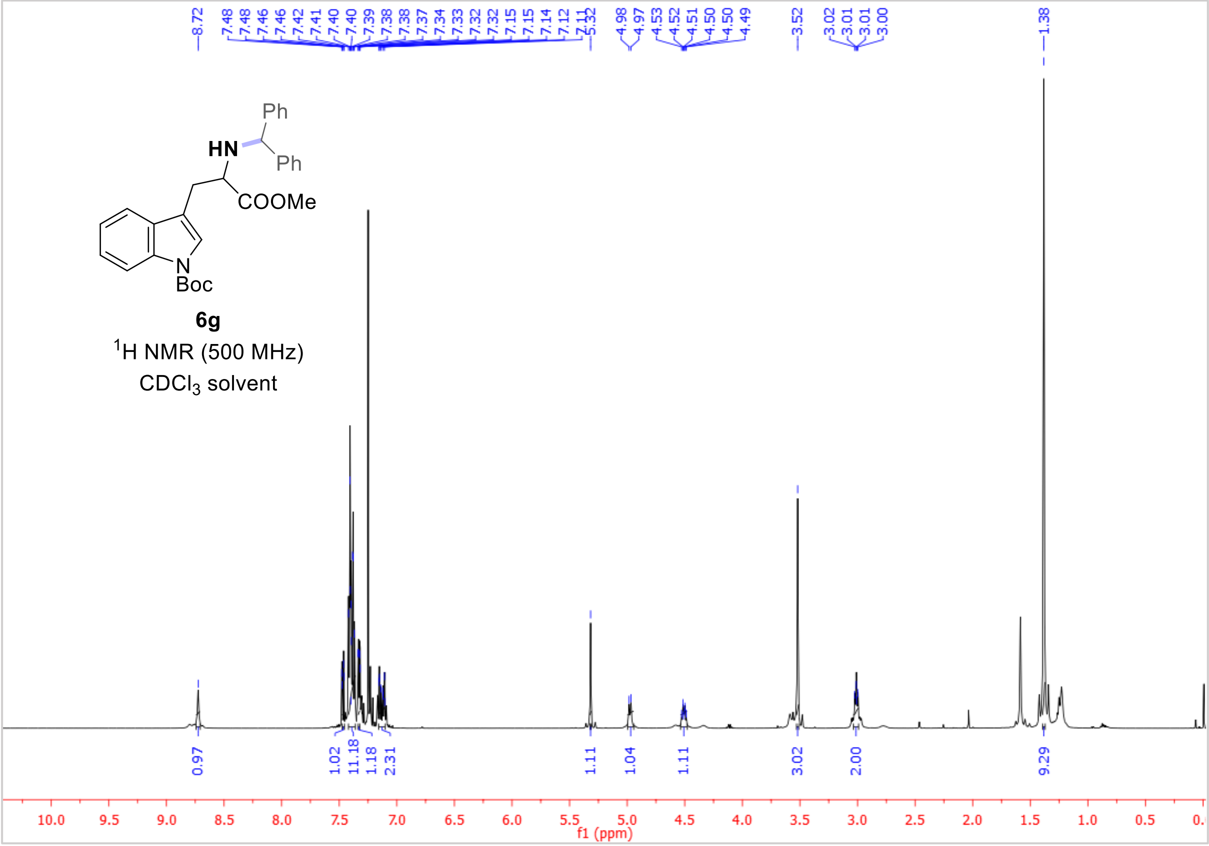
8.72
7.48
7.48
7.46
7.46
7.42
7.41
7.40
7.40
7.39
7.38
7.38
7.37
7.34
7.33
7.32
7.32
7.15
7.15
7.14
7.12
7.11
5.32
4.98
4.97
4.53
4.52
4.51
4.50
4.50
4.49
3.52
3.02
3.01
3.01
3.00
1.38
Ph
HN
Ph
COOMe
N
Boc
6g
^{1}H NMR (500 MHz)
$CDCl_3$ solvent
0.97
1.02
11.18
1.18
2.31
1.11
1.04
1.11
3.02
2.00
9.29
10.0 9.5 9.0 8.5 8.0 7.5 7.0 6.5 6.0 5.5 5.0 4.5 4.0 3.5 3.0 2.5 2.0 1.5 1.0 0.5 0.
f1 (ppm)

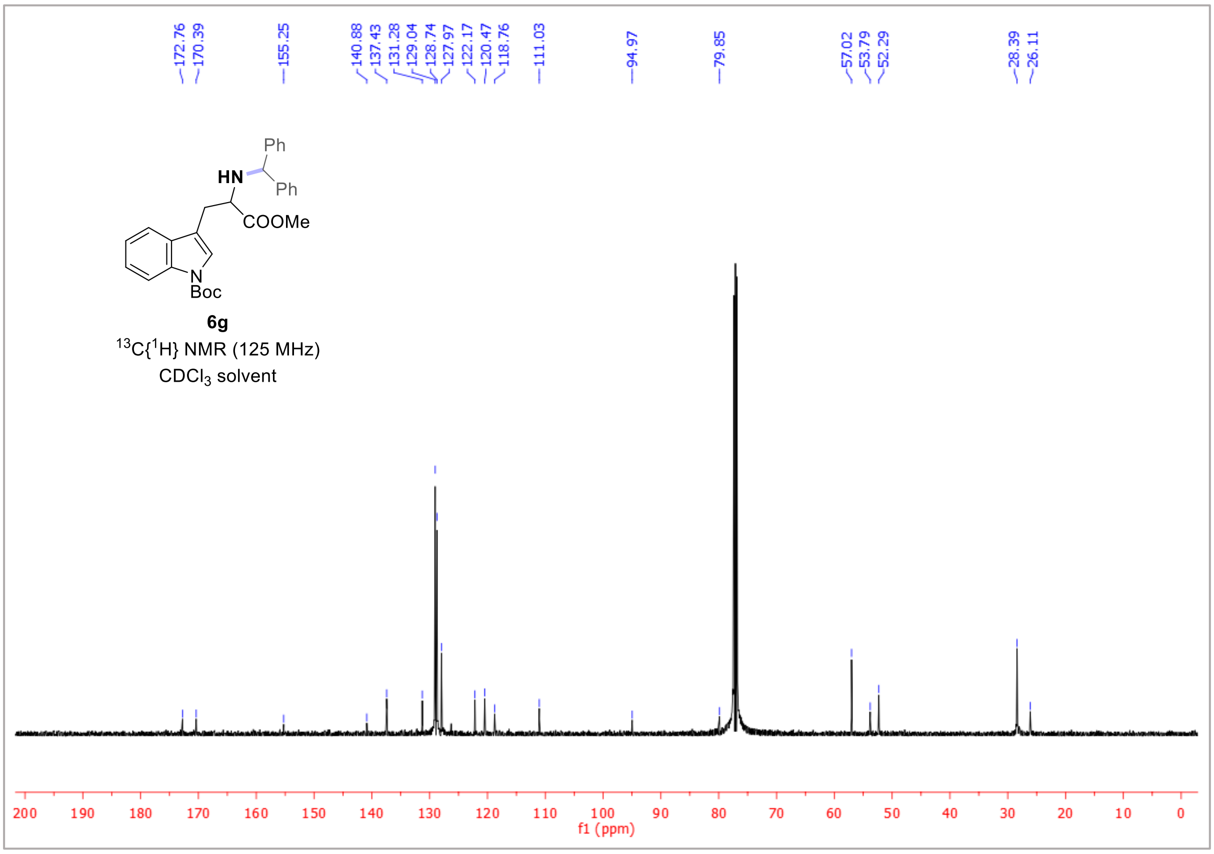
172.76
170.39
155.25
140.88
137.43
131.28
129.04
128.74
127.97
122.17
120.47
118.76
111.03
94.97
79.85
57.02
53.79
52.29
28.39
26.11
Ph
HN
Ph
COOMe
N
Boc
6g
$^{13}C\{^{1}H\}$ NMR (125 MHz)
$CDCl_3$ solvent
200 190 180 170 160 150 140 130 120 110 100 90 80 70 60 50 40 30 20 10 0
f1 (ppm)

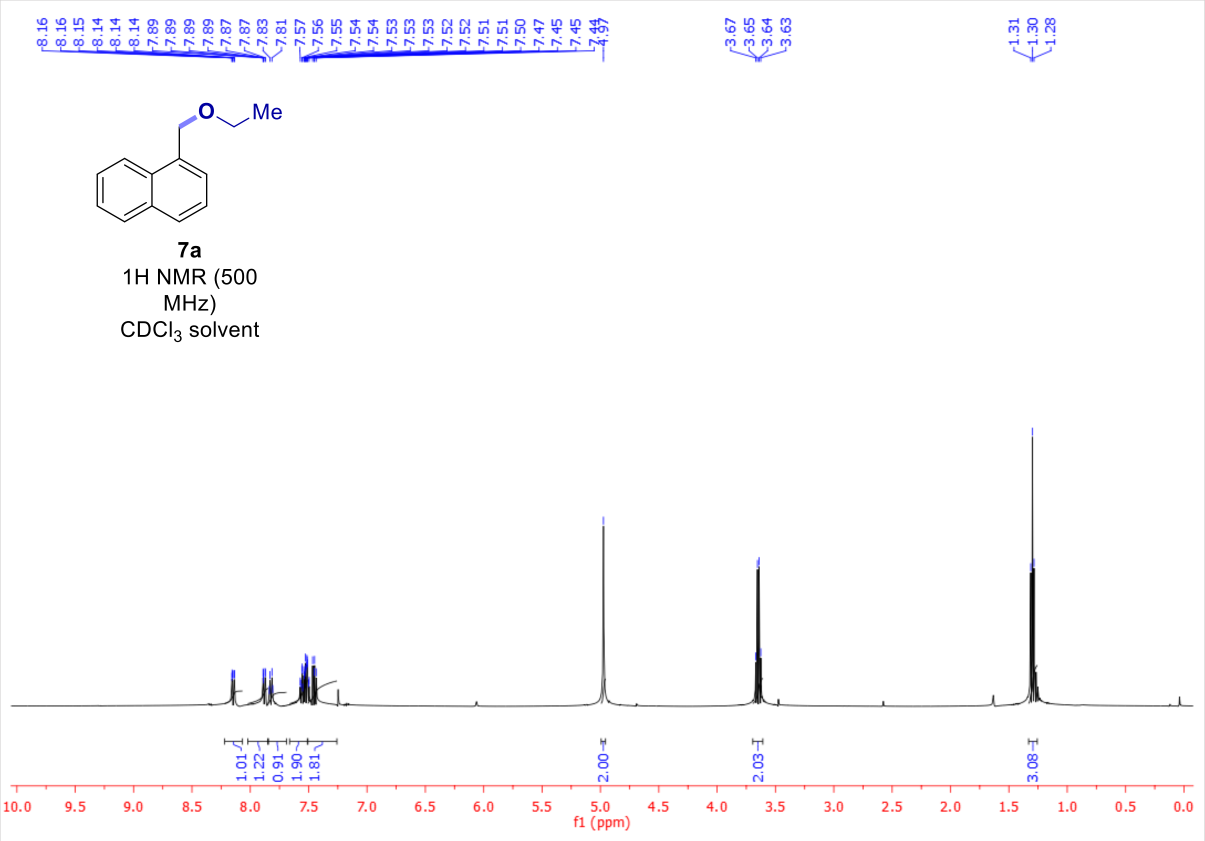

8.16
8.16
8.15
8.14
8.14
8.14
7.89
7.89
7.89
7.89
7.87
7.87
7.83
7.81
7.57
7.56
7.55
7.54
7.54
7.53
7.53
7.53
7.52
7.52
7.51
7.51
7.50
7.47
7.45
7.45
7.44
4.97
3.67
3.65
3.64
3.63
1.31
1.30
1.28
O
Me
7a
1H NMR (500 MHz)
$CDCl_3$ solvent
1.01
1.22
0.91
1.90
1.81
2.00
2.03
3.08
f1 (ppm)

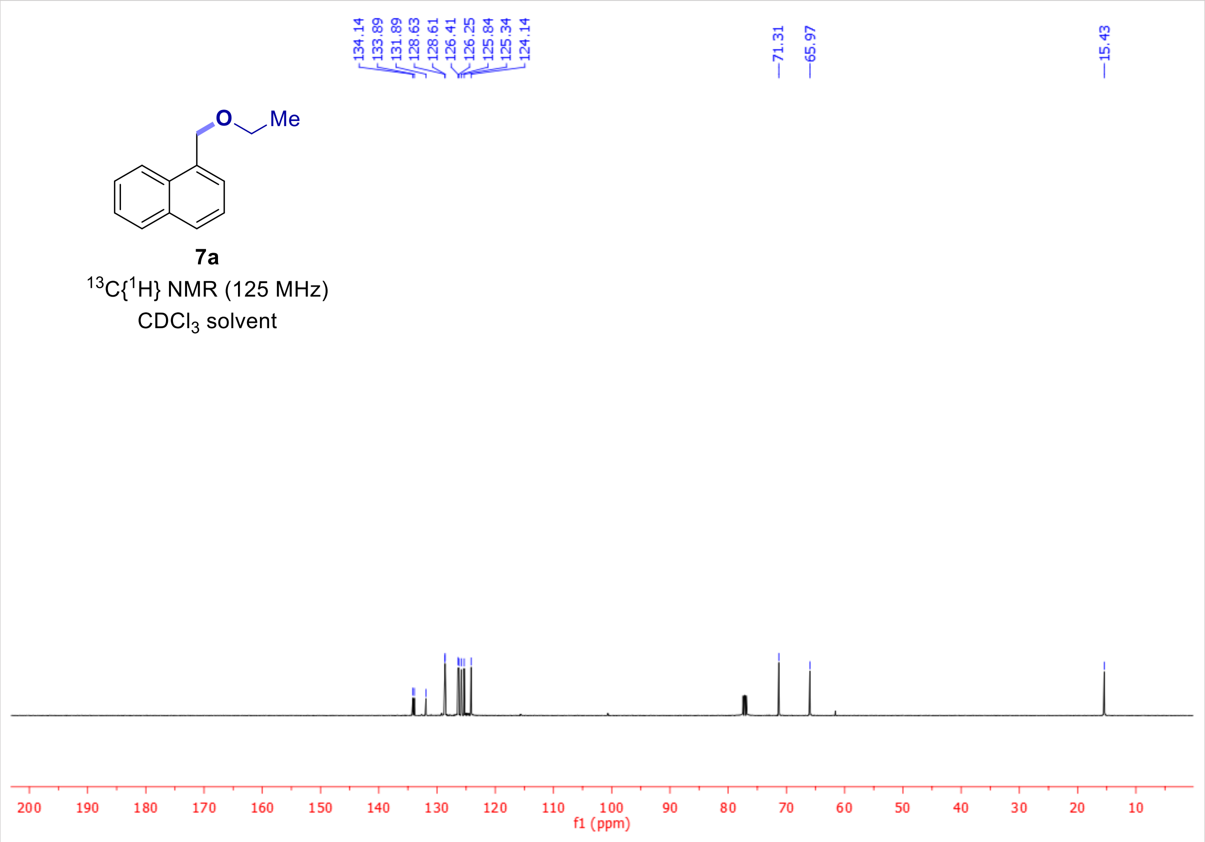

134.14
133.89
131.89
128.63
128.61
126.41
126.25
125.84
125.34
124.14
71.31
65.97
15.43
O
Me
7a
$^{13}C\{^1H\}$ NMR (125 MHz)
$CDCl_3$ solvent
f1 (ppm)

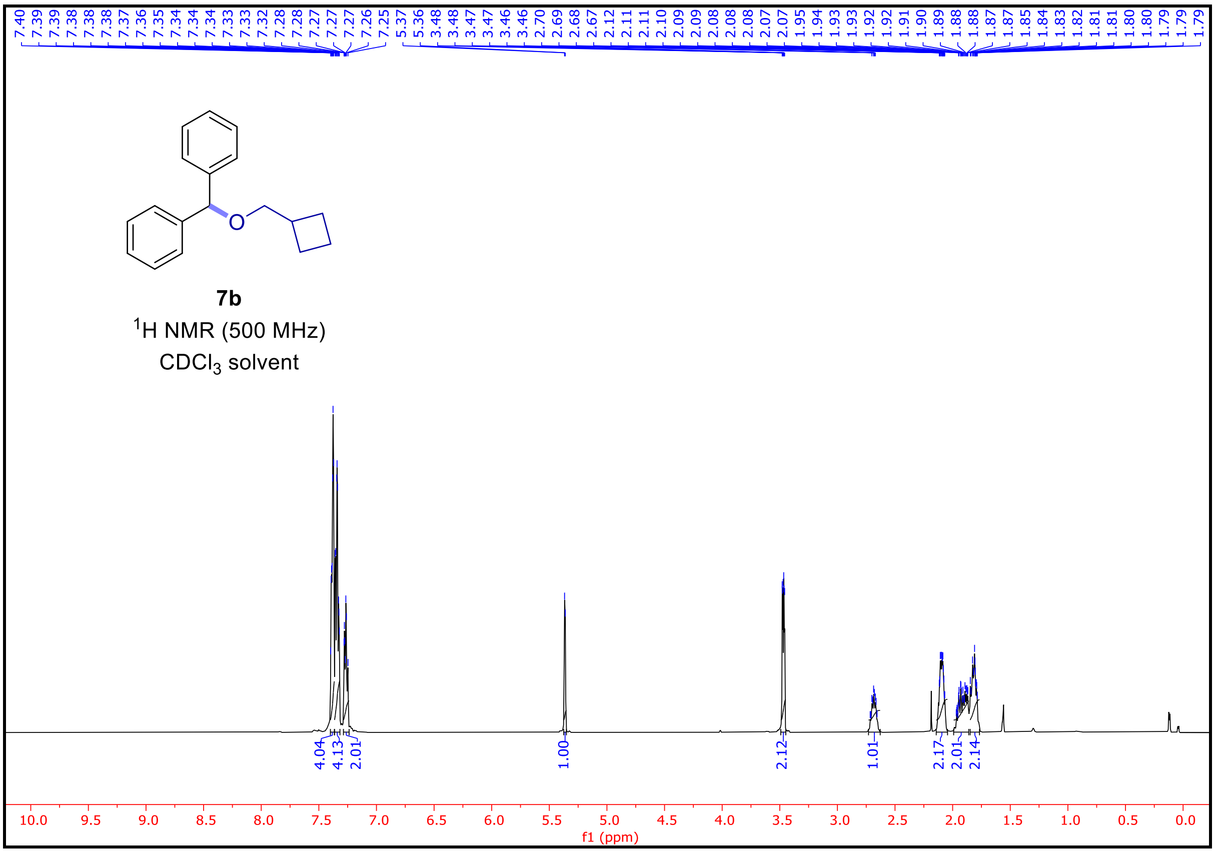
7.40 7.39 7.39 7.38 7.38 7.37 7.36 7.35 7.34 7.34 7.34 7.33 7.33 7.32 7.28 7.28 7.27 7.27 7.27 7.26 7.25 5.37 5.36 3.48 3.48 3.47 3.47 3.46 3.46 2.70 2.69 2.68 2.67 2.12 2.11 2.11 2.10 2.09 2.09 2.08 2.08 2.08 2.07 2.07 1.95 1.94 1.93 1.93 1.92 1.92 1.91 1.90 1.89 1.88 1.88 1.87 1.85 1.84 1.83 1.82 1.81 1.81 1.80 1.80 1.79 1.79 1.79
O
7b
1H NMR (500 MHz)
CDCl3 solvent
4.04 4.13 2.01 1.00 2.12 1.01 2.17 2.01 2.14
10.0 9.5 9.0 8.5 8.0 7.5 7.0 6.5 6.0 5.5 5.0 4.5 4.0 3.5 3.0 2.5 2.0 1.5 1.0 0.5 0.0
f1 (ppm)

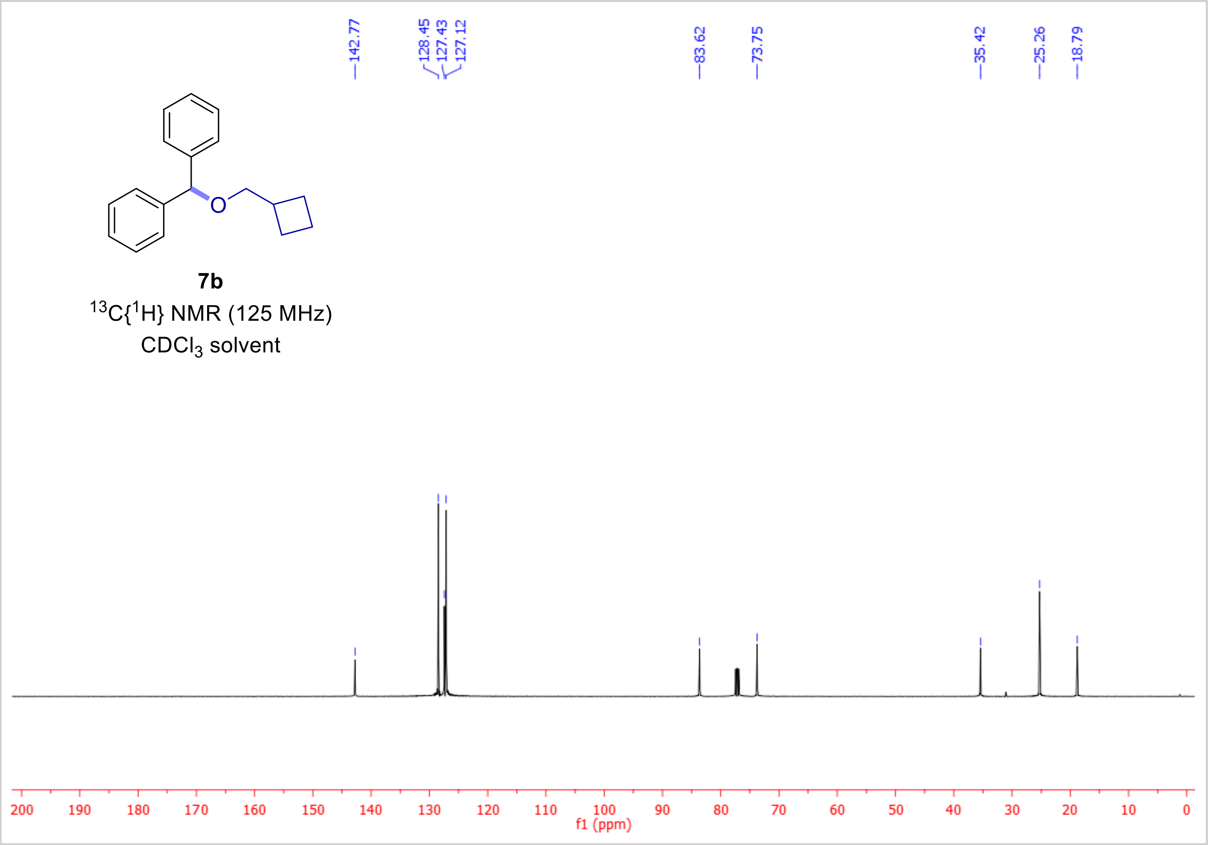
142.77
128.45 127.43 127.12
83.62
73.75
35.42
25.26
18.79
O
7b
13C{1H} NMR (125 MHz)
CDCl3 solvent
200 190 180 170 160 150 140 130 120 110 100 90 80 70 60 50 40 30 20 10 0
f1 (ppm)

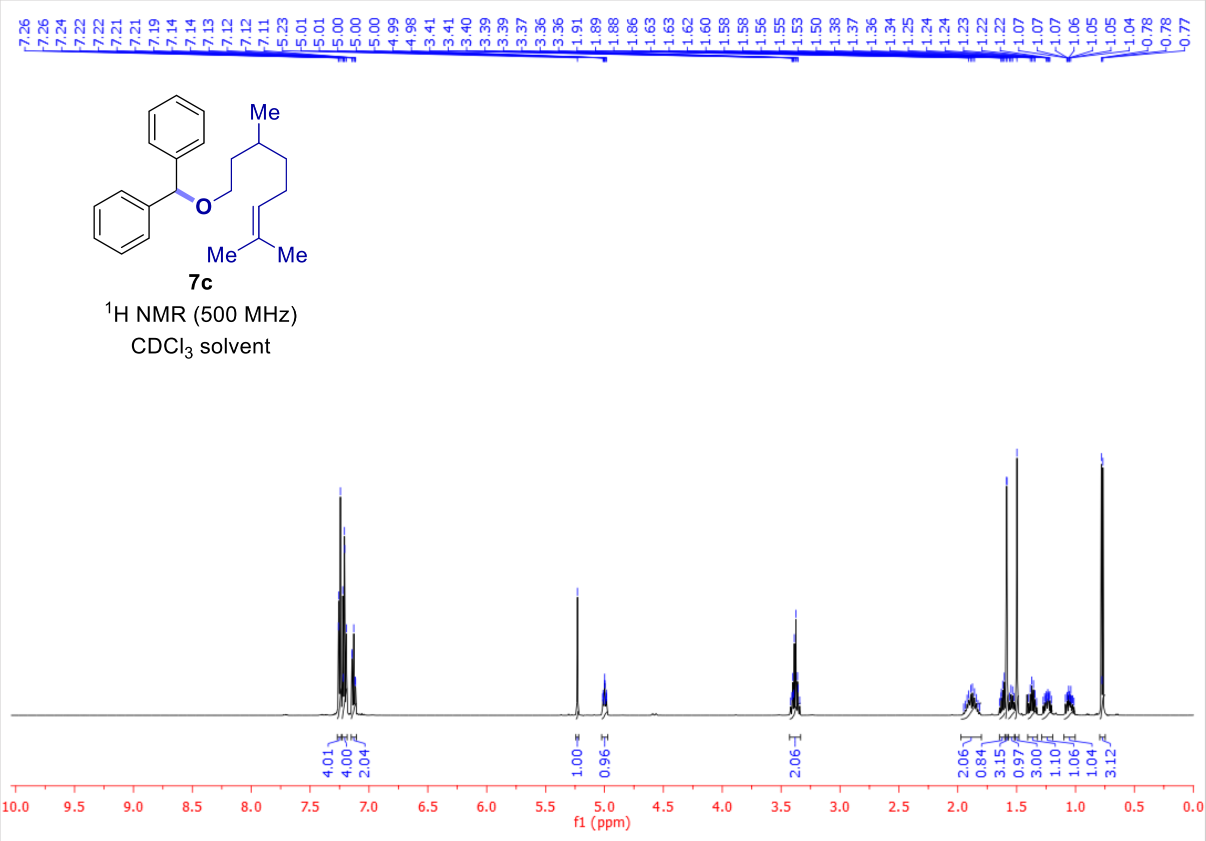
7c
^{1}H NMR (500 MHz)
$CDCl_3$ solvent
f1 (ppm)

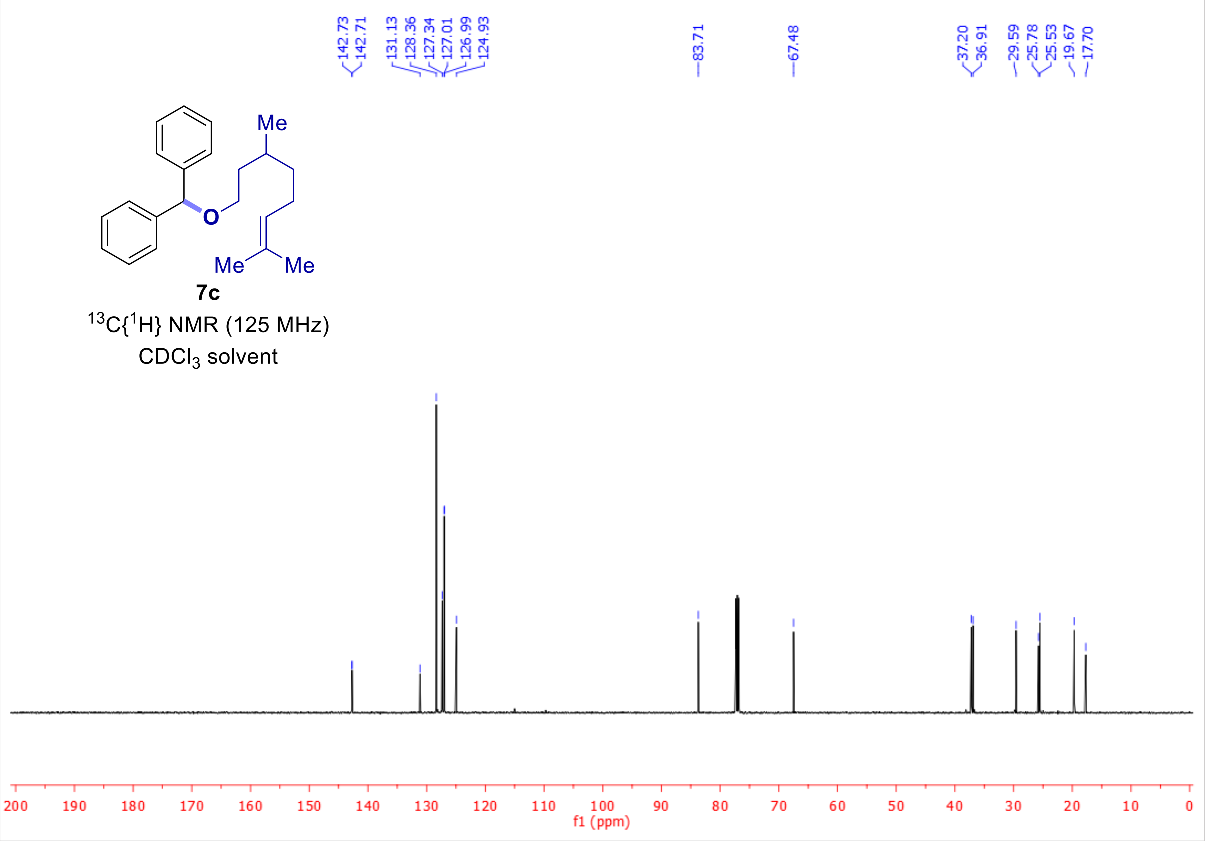
7c
$^{13}C\{^{1}H\}$ NMR (125 MHz)
$CDCl_3$ solvent
142.73
142.71
131.13
128.36
127.34
127.01
126.99
124.93
83.71
67.48
37.20
36.91
29.59
25.78
25.53
19.67
17.70
f1 (ppm)

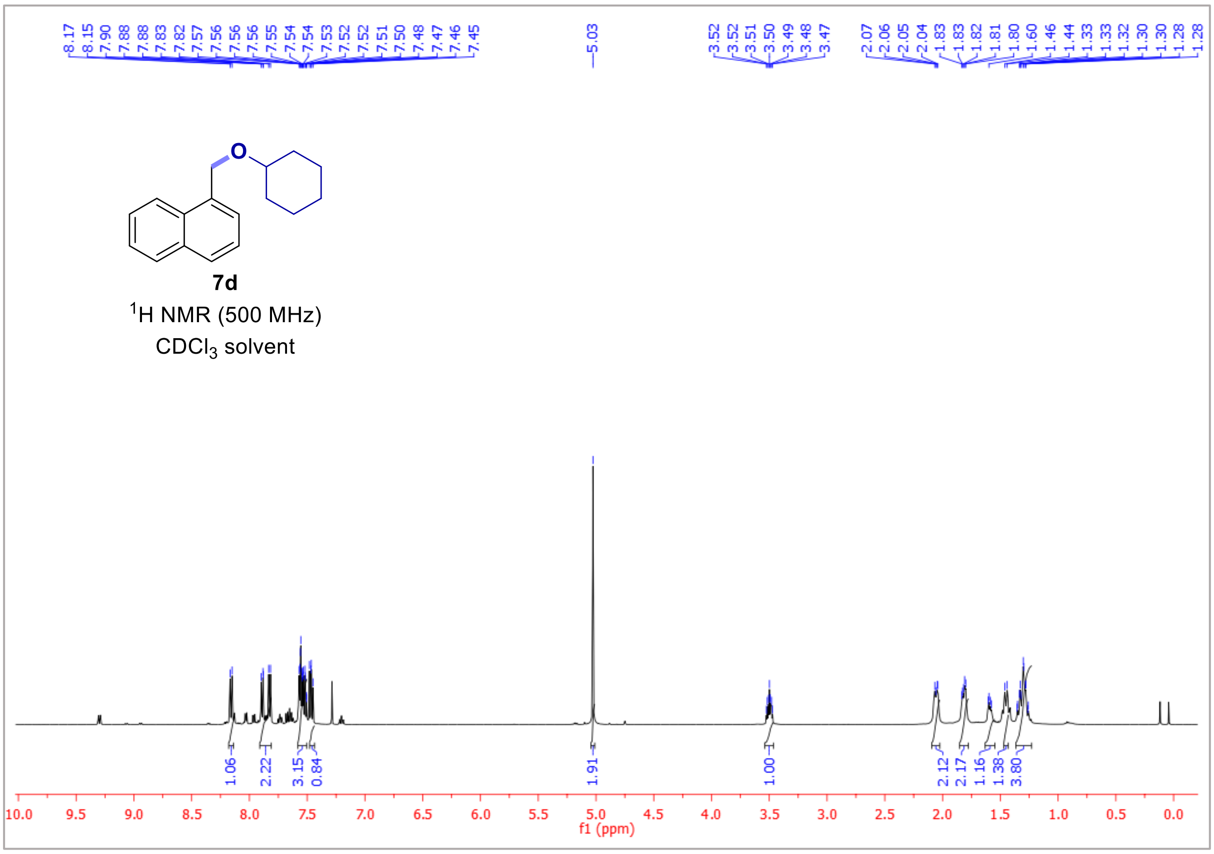
8.17
8.15
7.90
7.88
7.88
7.83
7.82
7.57
7.56
7.56
7.56
7.55
7.54
7.54
7.53
7.52
7.52
7.51
7.50
7.48
7.47
7.46
7.45
5.03
3.52
3.52
3.51
3.50
3.49
3.48
3.47
2.07
2.06
2.05
2.04
1.83
1.83
1.82
1.81
1.80
1.60
1.46
1.44
1.33
1.33
1.32
1.30
1.30
1.28
1.28
O
7d
^{1}H NMR (500 MHz)
$CDCl_3$ solvent
1.06
2.22
3.15
0.84
1.91
1.00
2.12
2.17
1.16
1.38
3.80
10.0 9.5 9.0 8.5 8.0 7.5 7.0 6.5 6.0 5.5 5.0 4.5 4.0 3.5 3.0 2.5 2.0 1.5 1.0 0.5 0.0
f1 (ppm)

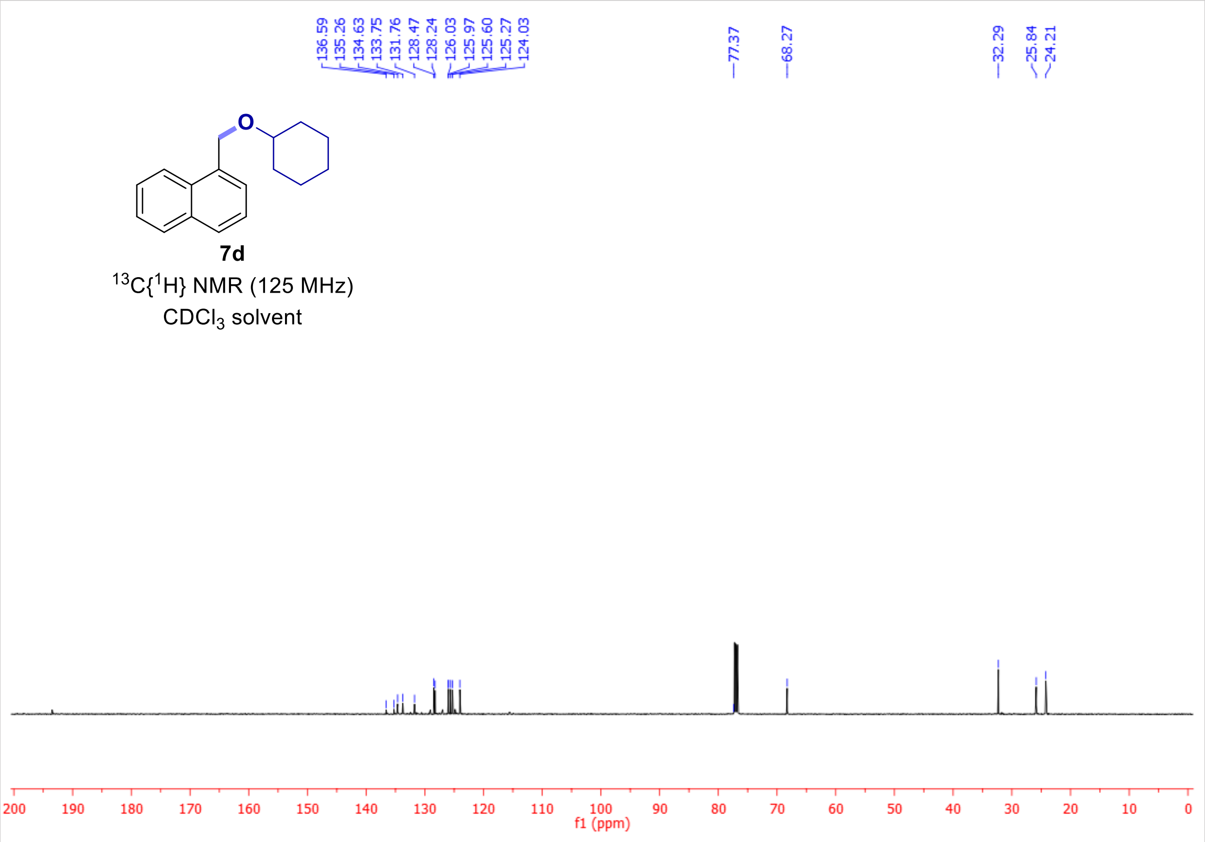
136.59
135.26
134.63
133.75
131.76
128.47
128.24
126.03
125.97
125.60
125.27
124.03
77.37
68.27
32.29
25.84
24.21
O
7d
^{13}C{^{1}H} NMR (125 MHz)
$CDCl_3$ solvent
200 190 180 170 160 150 140 130 120 110 100 90 80 70 60 50 40 30 20 10 0
f1 (ppm)

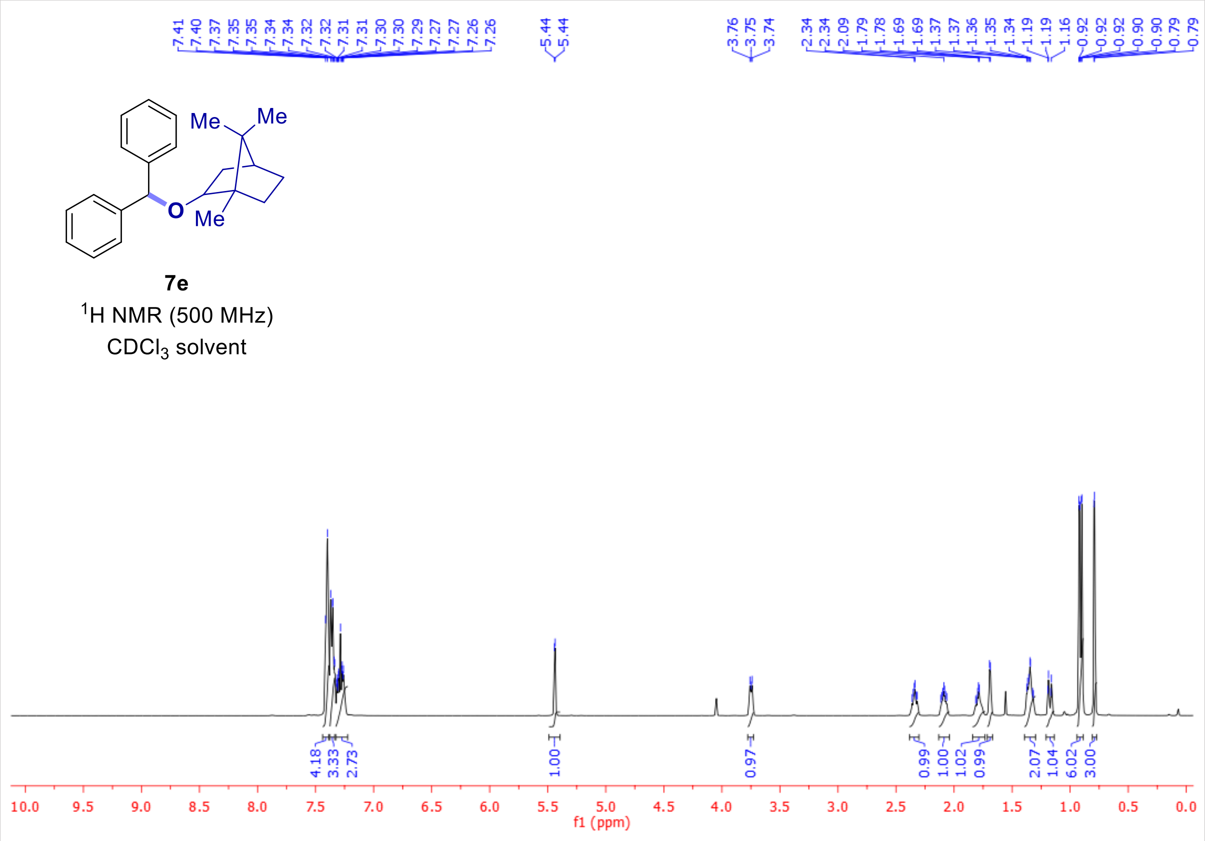
Me
Me
O
Me
7e
1H NMR (500 MHz)
CDCl3 solvent
f1 (ppm)

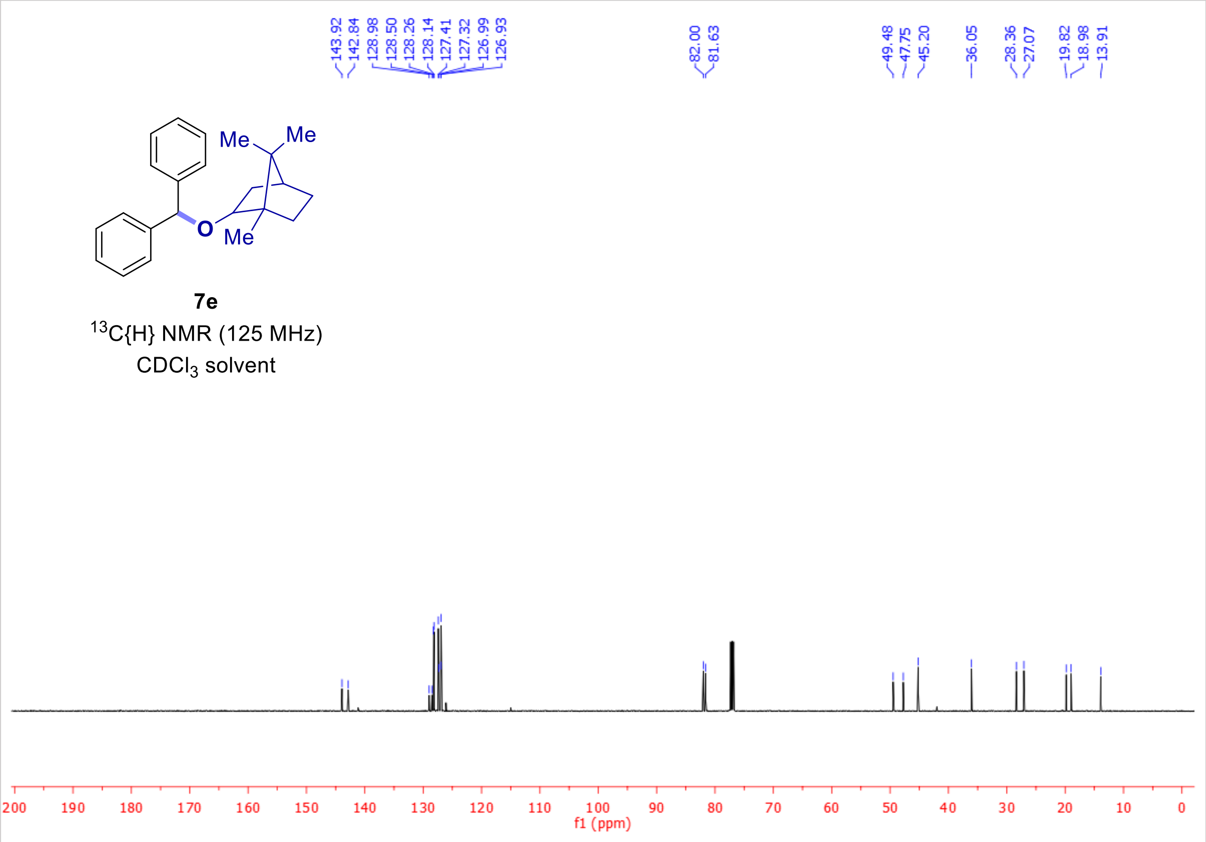
Me
Me
O
Me
7e
13C{H} NMR (125 MHz)
CDCl3 solvent
f1 (ppm)

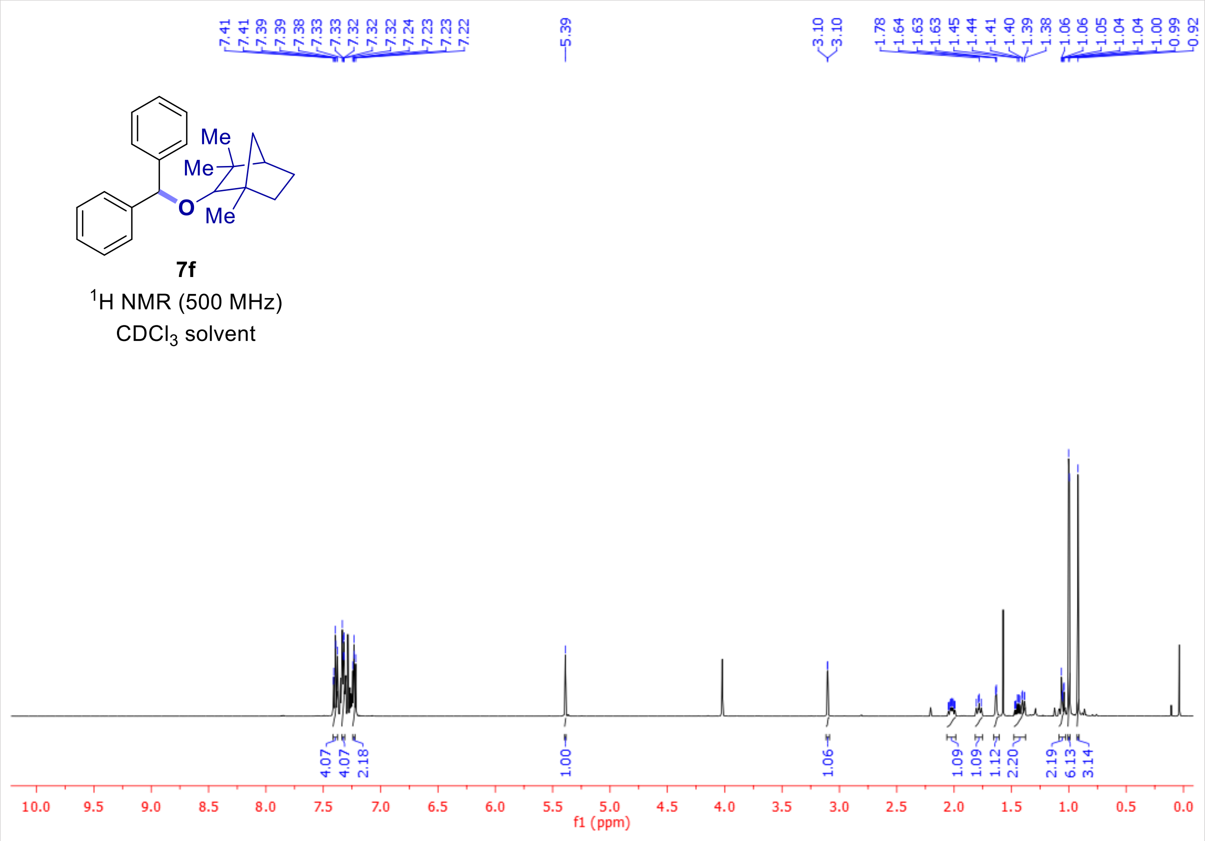
7.41
7.41
7.39
7.39
7.38
7.33
7.33
7.32
7.32
7.32
7.24
7.23
7.23
7.22
5.39
3.10
3.10
1.78
1.64
1.63
1.63
1.45
1.44
1.41
1.40
1.39
1.38
1.06
1.06
1.05
1.04
1.04
1.00
0.99
0.92
Me
Me
O
Me
7f
1H NMR (500 MHz)
CDCl3 solvent
4.07
4.07
2.18
1.00
1.06
1.09
1.09
1.12
2.20
2.19
6.13
3.14
10.0
9.5
9.0
8.5
8.0
7.5
7.0
6.5
6.0
5.5
5.0
4.5
4.0
3.5
3.0
2.5
2.0
1.5
1.0
0.5
0.0
f1 (ppm)

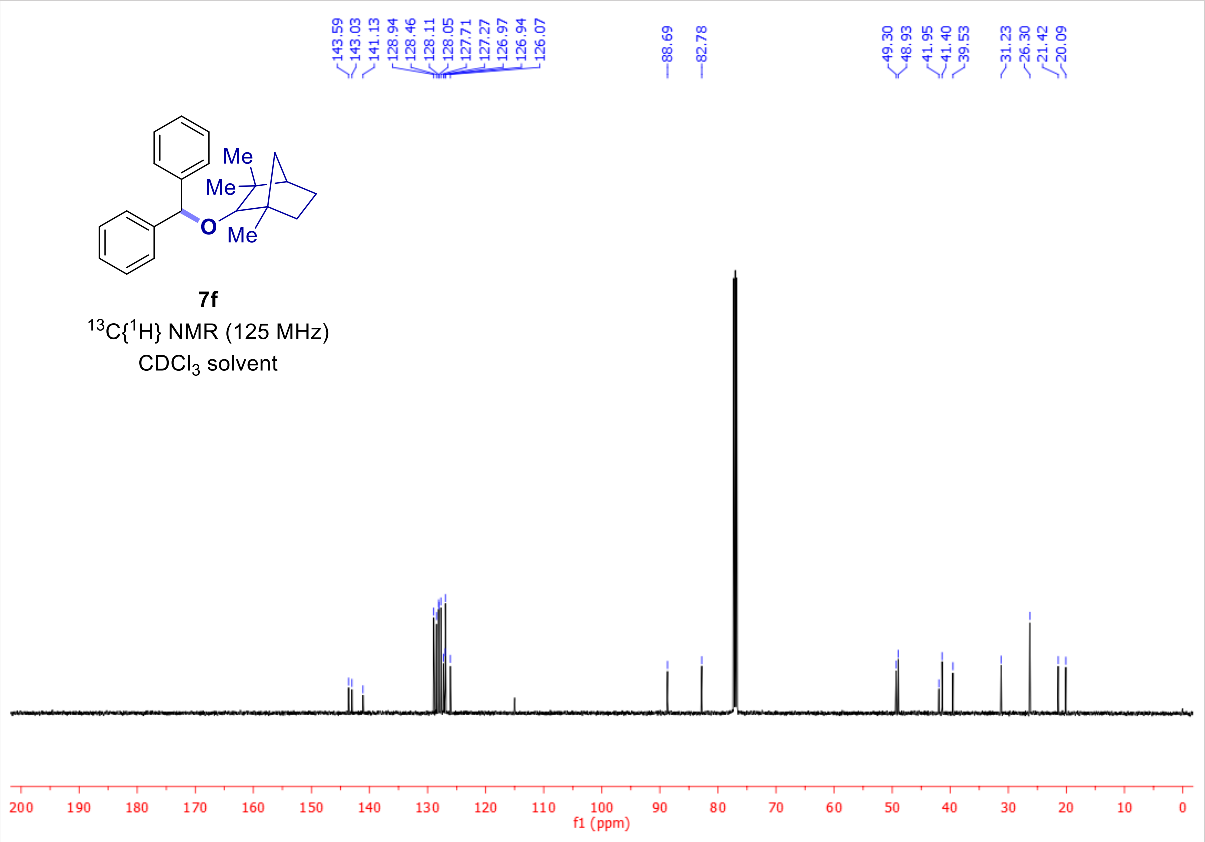
143.59
143.03
141.13
128.94
128.46
128.11
128.05
127.71
127.27
126.97
126.94
126.07
88.69
82.78
49.30
48.93
41.95
41.40
39.53
31.23
26.30
21.42
20.09
Me
Me
O
Me
7f
13C{1H} NMR (125 MHz)
CDCl3 solvent
200
190
180
170
160
150
140
130
120
110
100
90
80
70
60
50
40
30
20
10
0
f1 (ppm)

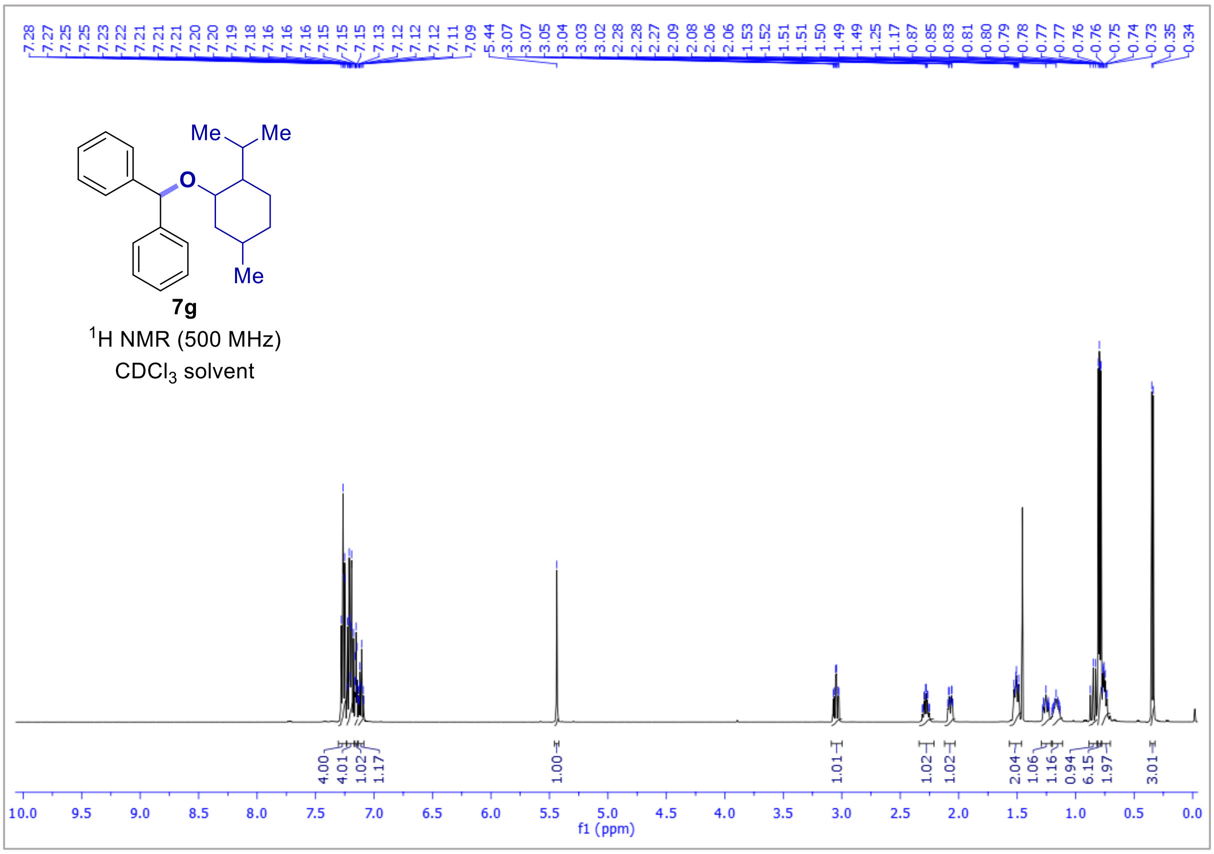
7.28 7.27 7.25 7.25 7.23 7.22 7.21 7.21 7.21 7.20 7.20 7.19 7.18 7.16 7.16 7.15 7.15 7.15 7.13 7.12 7.12 7.12 7.11 7.09 5.44 3.07 3.07 3.05 3.04 3.03 3.02 2.28 2.28 2.27 2.09 2.08 2.06 2.06 1.53 1.52 1.51 1.51 1.50 1.49 1.49 1.25 1.17 0.87 0.85 0.83 0.81 0.80 0.79 0.78 0.77 0.77 0.76 0.76 0.75 0.74 0.73 0.35 0.34
Me
Me
O
Me
7g
1H NMR (500 MHz)
CDCl3 solvent
4.00 4.01 1.02 1.17 1.00 1.01 1.02 1.02 2.04 1.06 1.16 0.94 6.15 1.97 3.01
f1 (ppm)

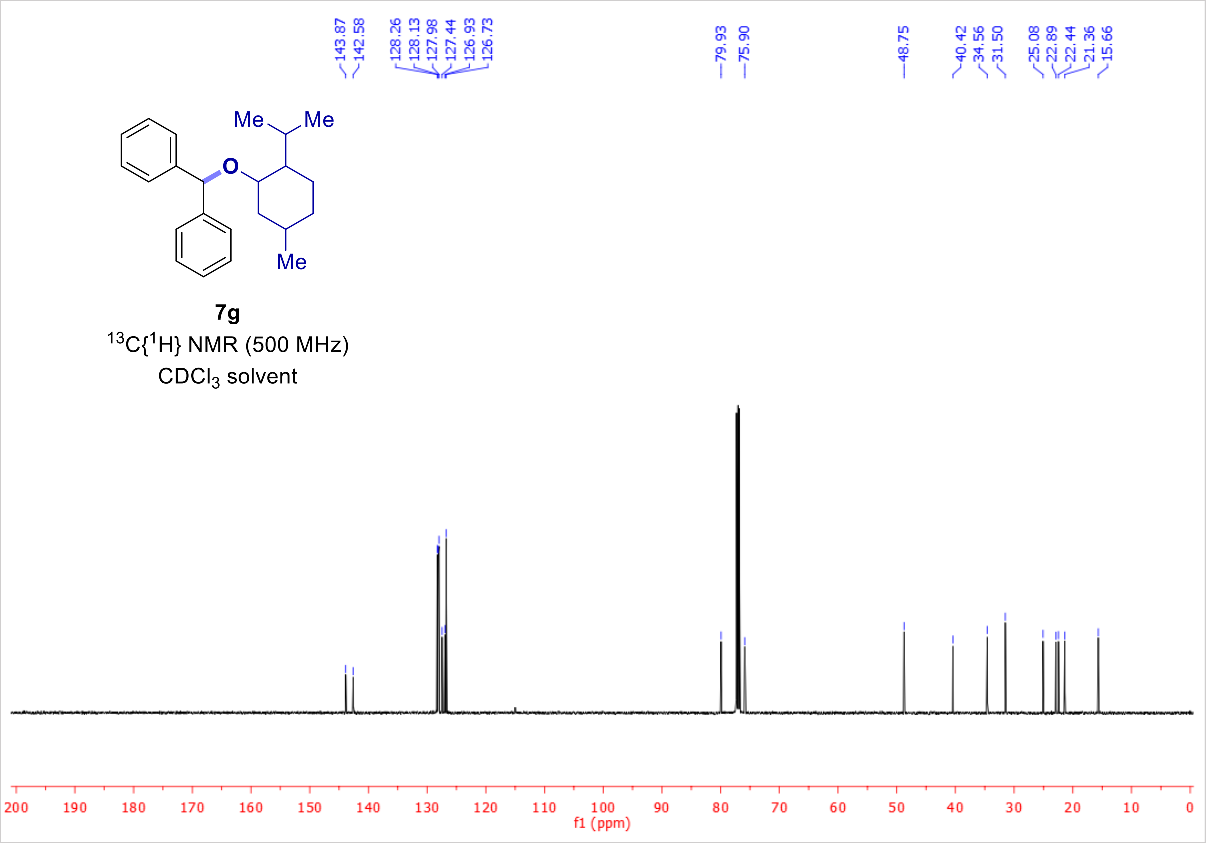
143.87 142.58 128.26 128.13 127.98 127.44 126.93 126.73 79.93 75.90 48.75 40.42 34.56 31.50 25.08 22.89 22.44 21.36 15.66
Me
Me
O
Me
7g
13C{1H} NMR (500 MHz)
CDCl3 solvent
f1 (ppm)

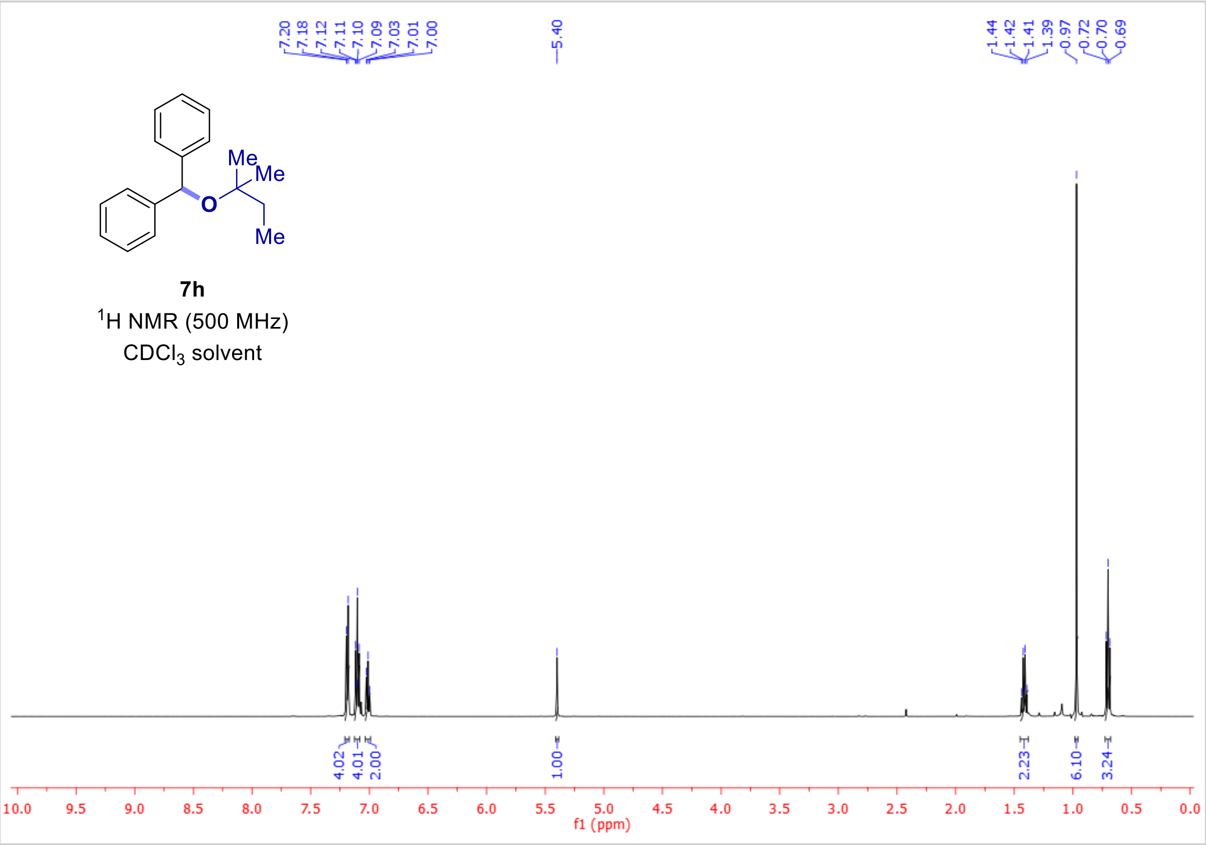
7.20
7.18
7.12
7.11
7.10
7.09
7.03
7.01
7.00
5.40
1.44
1.42
1.41
1.39
0.97
0.72
0.70
0.69
Me
Me
O
Me
7h
1H NMR (500 MHz)
CDCl3 solvent
4.02
4.01
2.00
1.00
2.23
6.10
3.24
10.0 9.5 9.0 8.5 8.0 7.5 7.0 6.5 6.0 5.5 5.0 4.5 4.0 3.5 3.0 2.5 2.0 1.5 1.0 0.5 0.0
f1 (ppm)

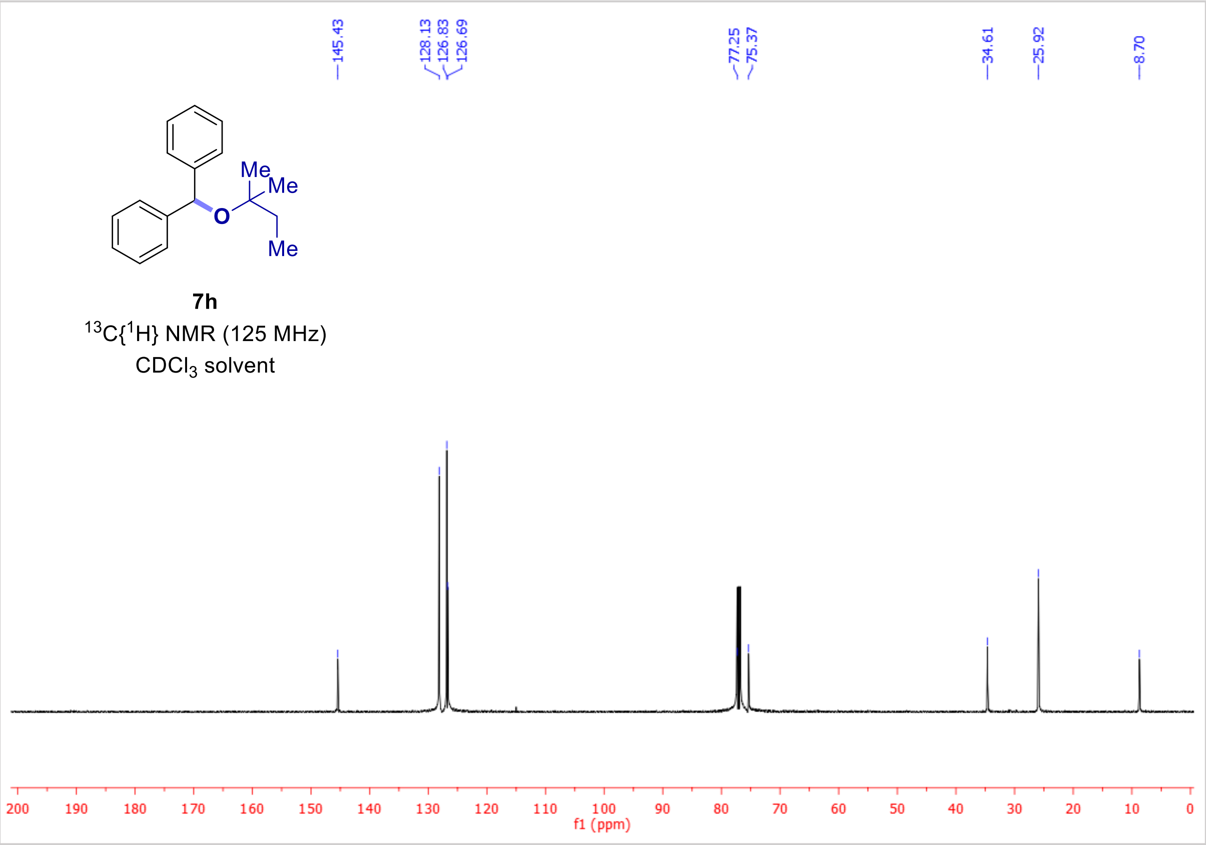
145.43
128.13
126.83
126.69
77.25
75.37
34.61
25.92
8.70
Me
Me
O
Me
7h
13C{1H} NMR (125 MHz)
CDCl3 solvent
200 190 180 170 160 150 140 130 120 110 100 90 80 70 60 50 40 30 20 10 0
f1 (ppm)

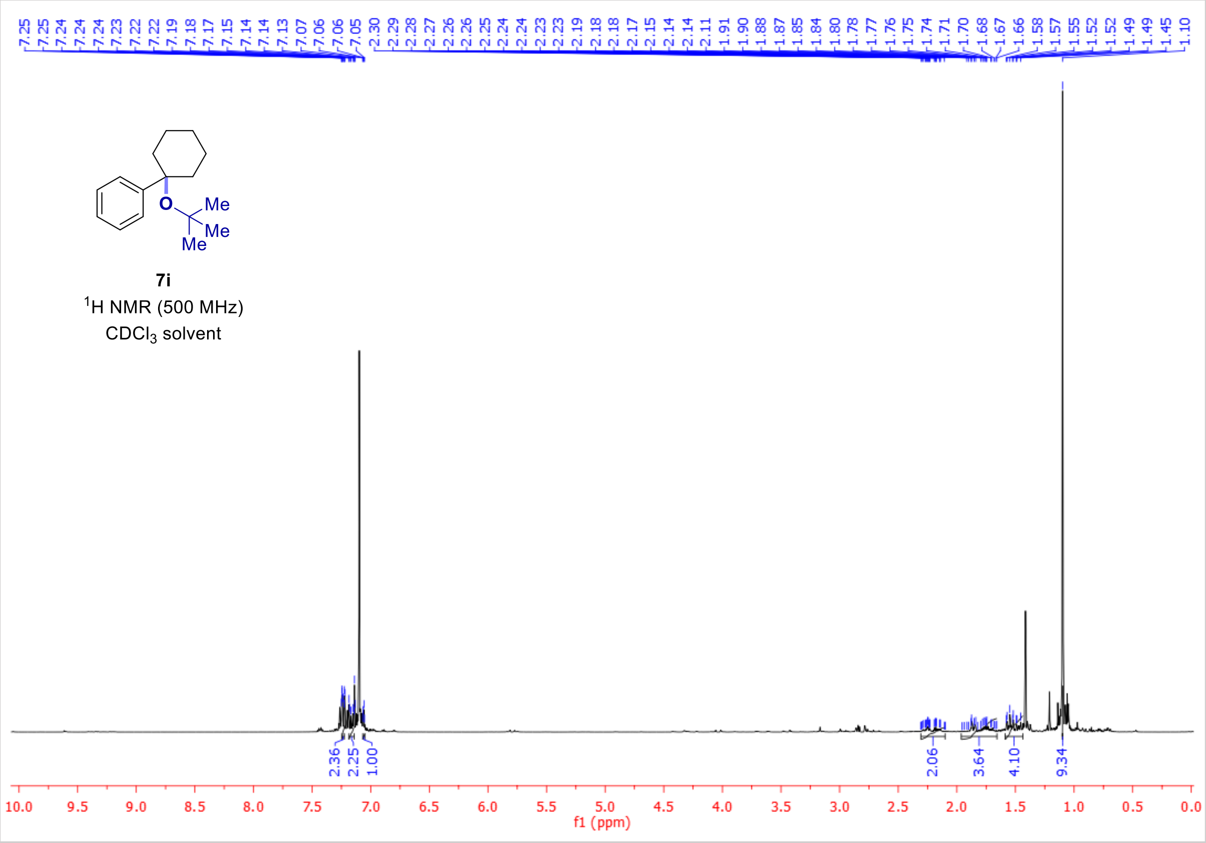

O
Me
Me
Me
7i
^{1}H NMR (500 MHz)
$CDCl_3$ solvent
2.36
2.25
1.00
2.06
3.64
4.10
9.34
f1 (ppm)

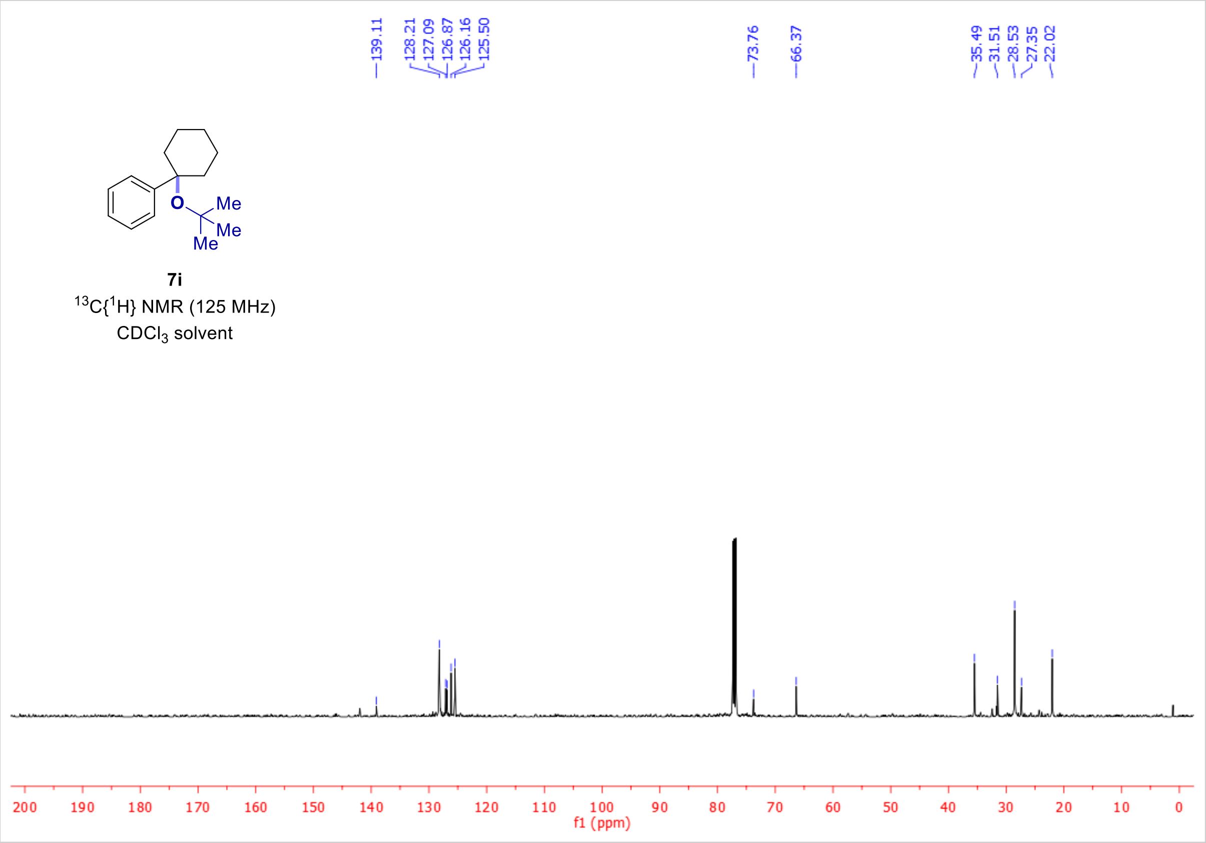

139.11
128.21
127.09
126.87
126.16
125.50
73.76
66.37
35.49
31.51
28.53
27.35
22.02
O
Me
Me
Me
7i
^{13}C{^{1}H} NMR (125 MHz)
$CDCl_3$ solvent
f1 (ppm)

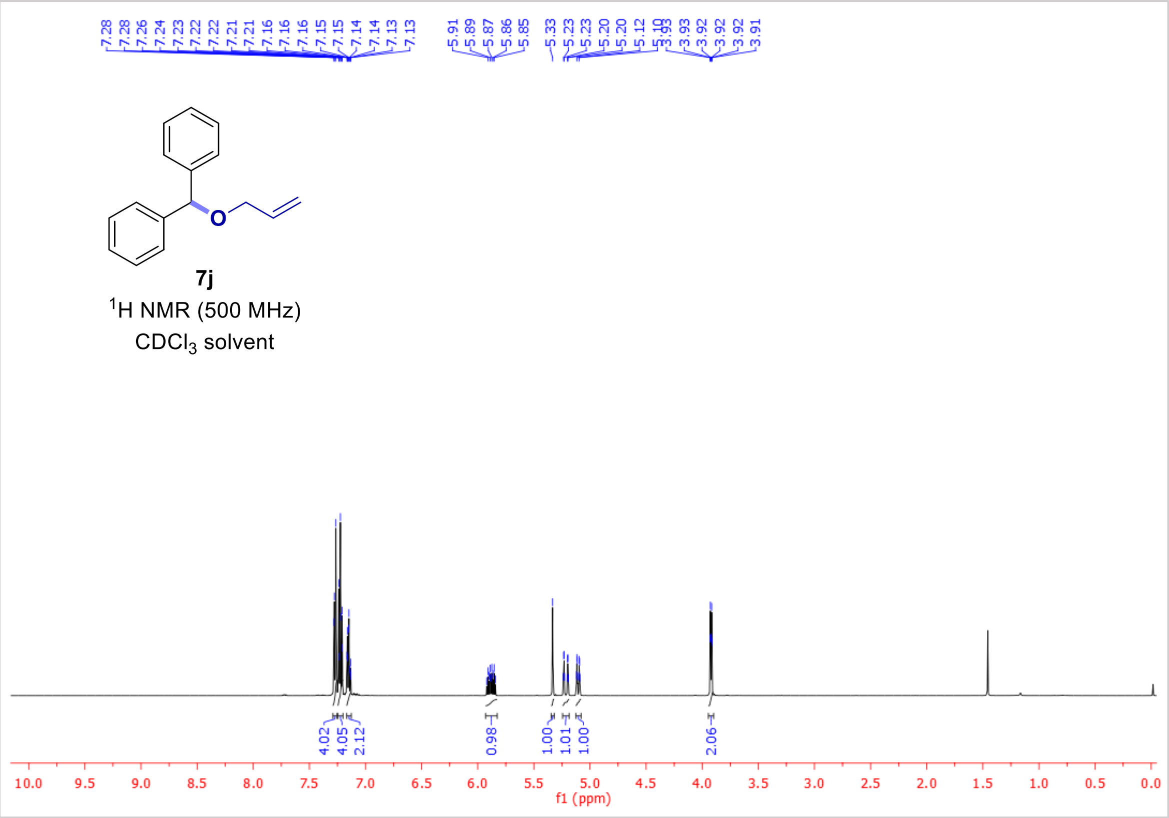
7.28 7.28 7.26 7.24 7.23 7.22 7.22 7.21 7.21 7.16 7.16 7.16 7.15 7.15 7.14 7.14 7.13 7.13
5.91 5.89 5.87 5.86 5.85
5.33 5.23 5.23 5.23 5.20 5.20 5.12 5.10 3.93 3.93 3.92 3.92 3.92 3.91
O
7j
1H NMR (500 MHz)
CDCl3 solvent
4.02 4.05 2.12 0.98 1.00 1.01 1.00 2.06
10.0 9.5 9.0 8.5 8.0 7.5 7.0 6.5 6.0 5.5 5.0 4.5 4.0 3.5 3.0 2.5 2.0 1.5 1.0 0.5 0.0
f1 (ppm)

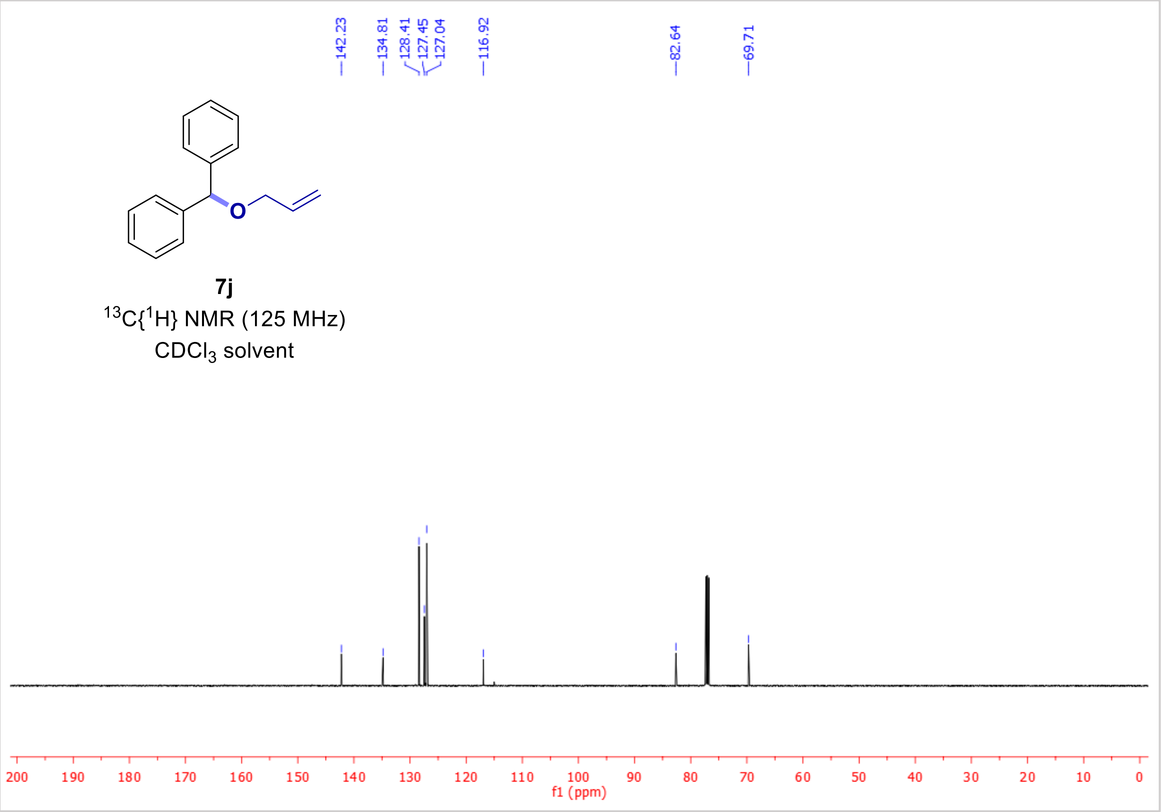
142.23
134.81
128.41 127.45 127.04
116.92
82.64
69.71
O
7j
13C{1H} NMR (125 MHz)
CDCl3 solvent
200 190 180 170 160 150 140 130 120 110 100 90 80 70 60 50 40 30 20 10 0
f1 (ppm)

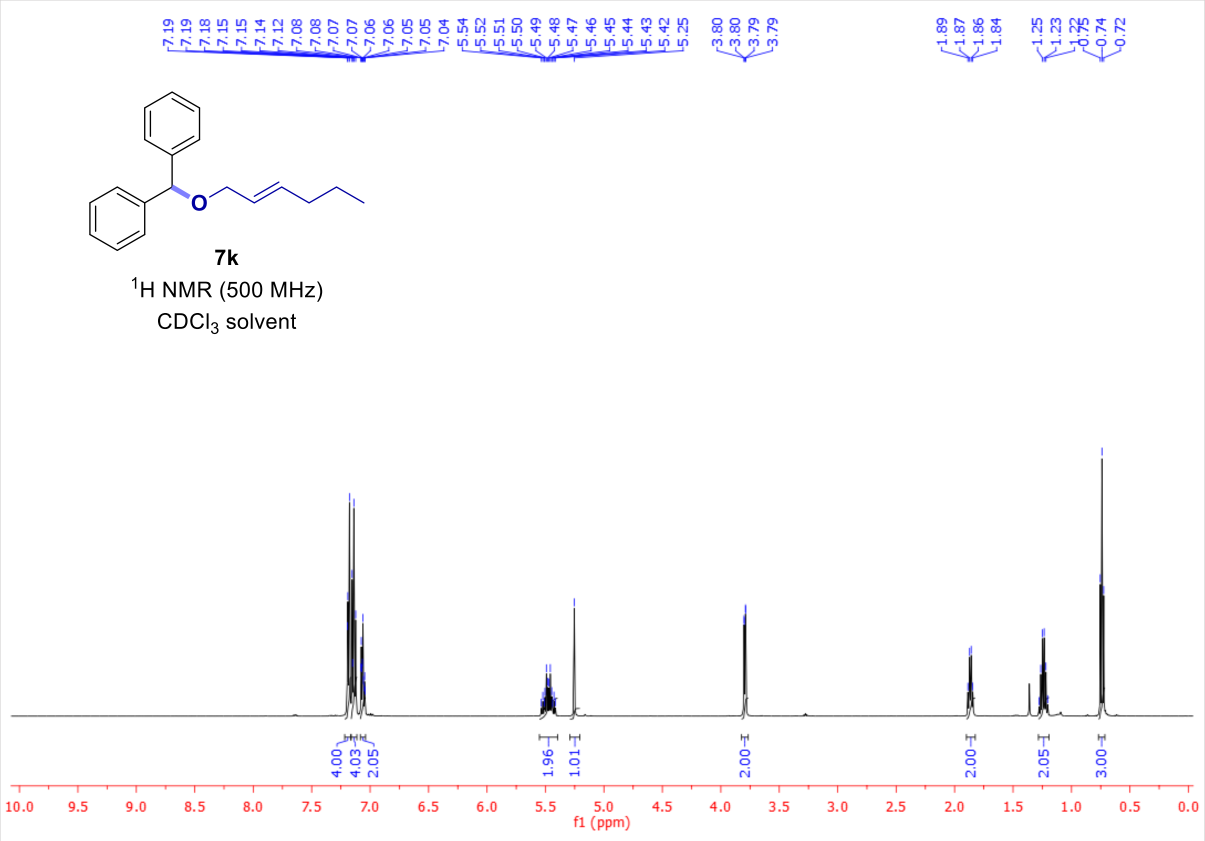
7.19 7.19 7.18 7.15 7.15 7.14 7.12 7.08 7.08 7.07 7.07 7.06 7.06 7.05 7.05 7.04 5.54 5.52 5.51 5.50 5.49 5.48 5.47 5.46 5.45 5.44 5.43 5.42 5.25 3.80 3.80 3.79 3.79 1.89 1.87 1.86 1.84 1.25 1.23 1.22 0.75 0.74 0.72
O
7k
1H NMR (500 MHz)
CDCl3 solvent
4.00 4.03 2.05 1.96 1.01 2.00 2.00 2.05 3.00
10.0 9.5 9.0 8.5 8.0 7.5 7.0 6.5 6.0 5.5 5.0 4.5 4.0 3.5 3.0 2.5 2.0 1.5 1.0 0.5 0.0
f1 (ppm)

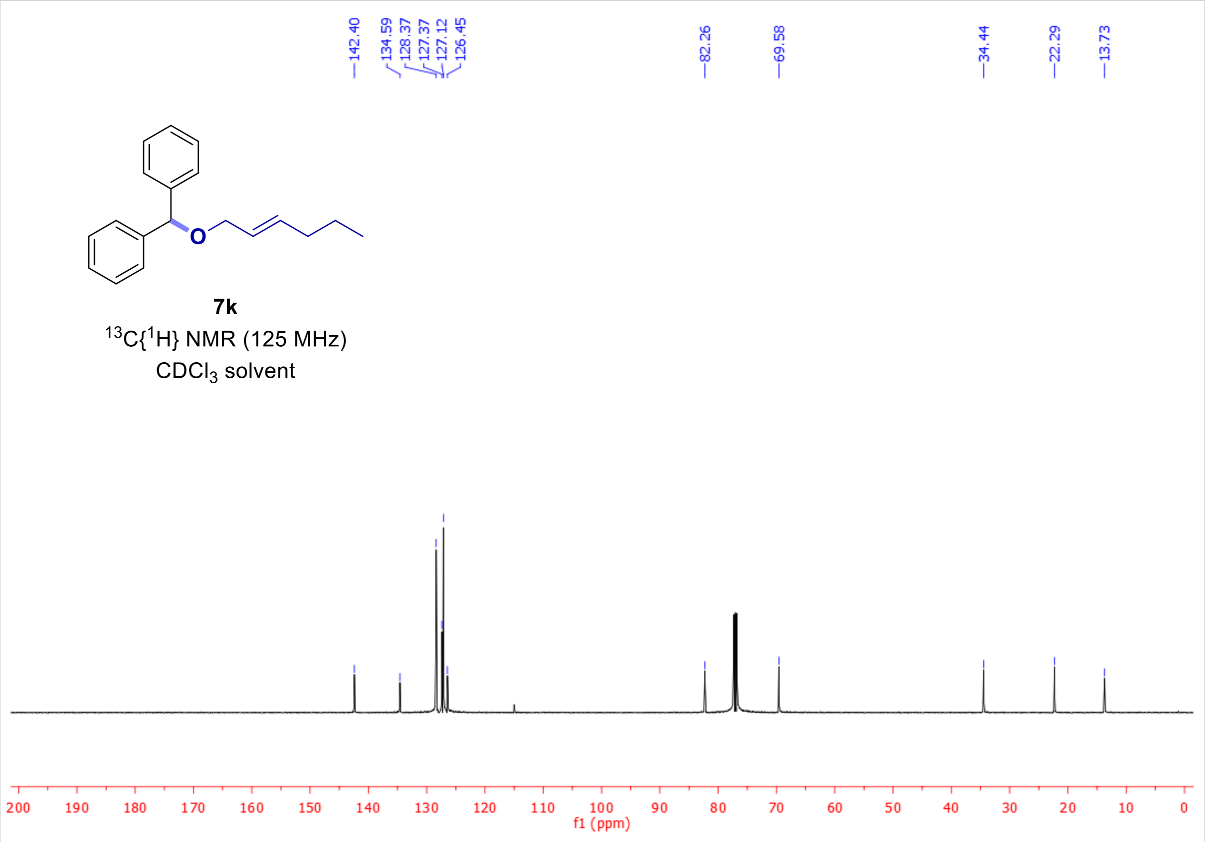
142.40 134.59 128.37 127.37 127.12 126.45 82.26 69.58 34.44 22.29 13.73
O
7k
13C{1H} NMR (125 MHz)
CDCl3 solvent
200 190 180 170 160 150 140 130 120 110 100 90 80 70 60 50 40 30 20 10 0
f1 (ppm)

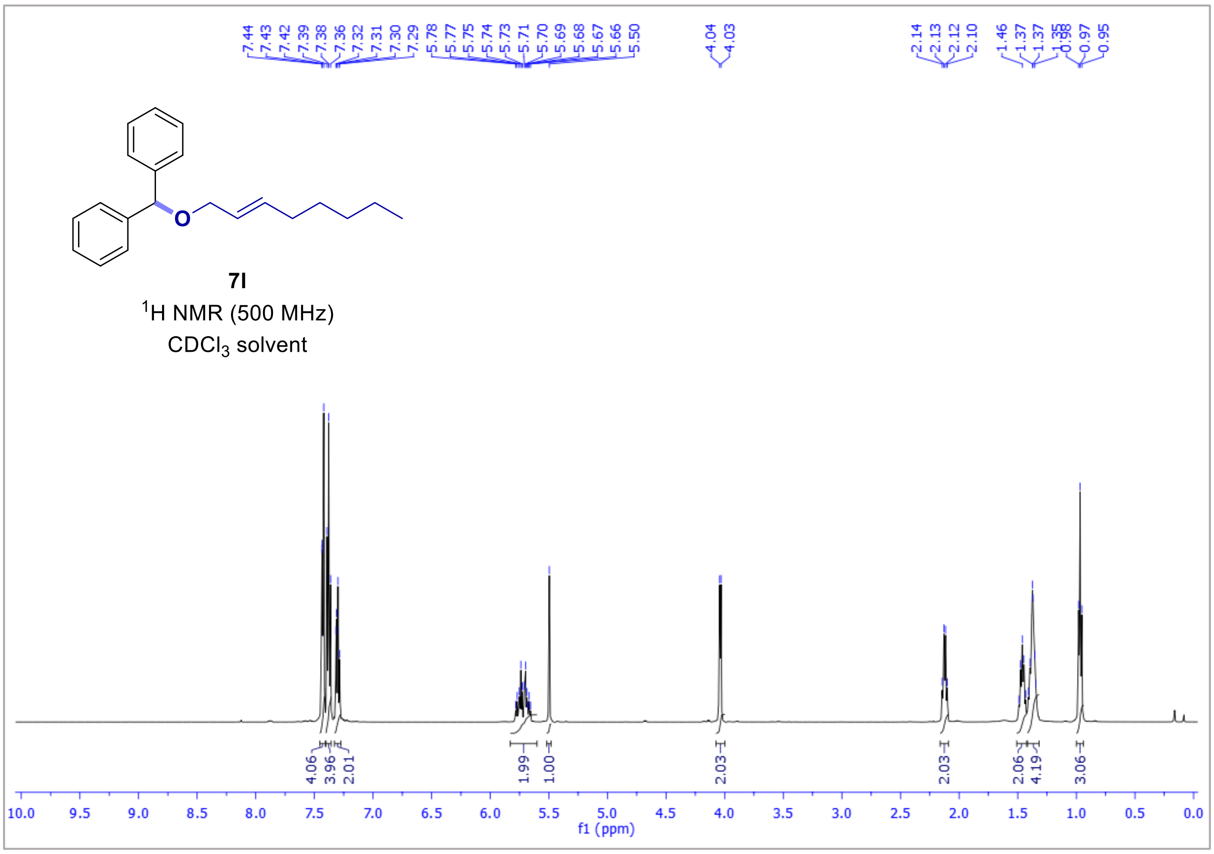

7.44 7.43 7.42 7.39 7.38 7.36 7.32 7.31 7.30 7.29 5.78 5.77 5.75 5.74 5.73 5.71 5.70 5.69 5.68 5.67 5.66 5.50 4.04 4.03 2.14 2.13 2.12 2.10 1.46 1.37 1.37 1.35 0.98 0.97 0.95
O
7l
^{1}H NMR (500 MHz)
$CDCl_3$ solvent
4.06 3.96 2.01 1.99 1.00 2.03 2.03 2.06 4.19 3.06
10.0 9.5 9.0 8.5 8.0 7.5 7.0 6.5 6.0 5.5 5.0 4.5 4.0 3.5 3.0 2.5 2.0 1.5 1.0 0.5 0.0
f1 (ppm)

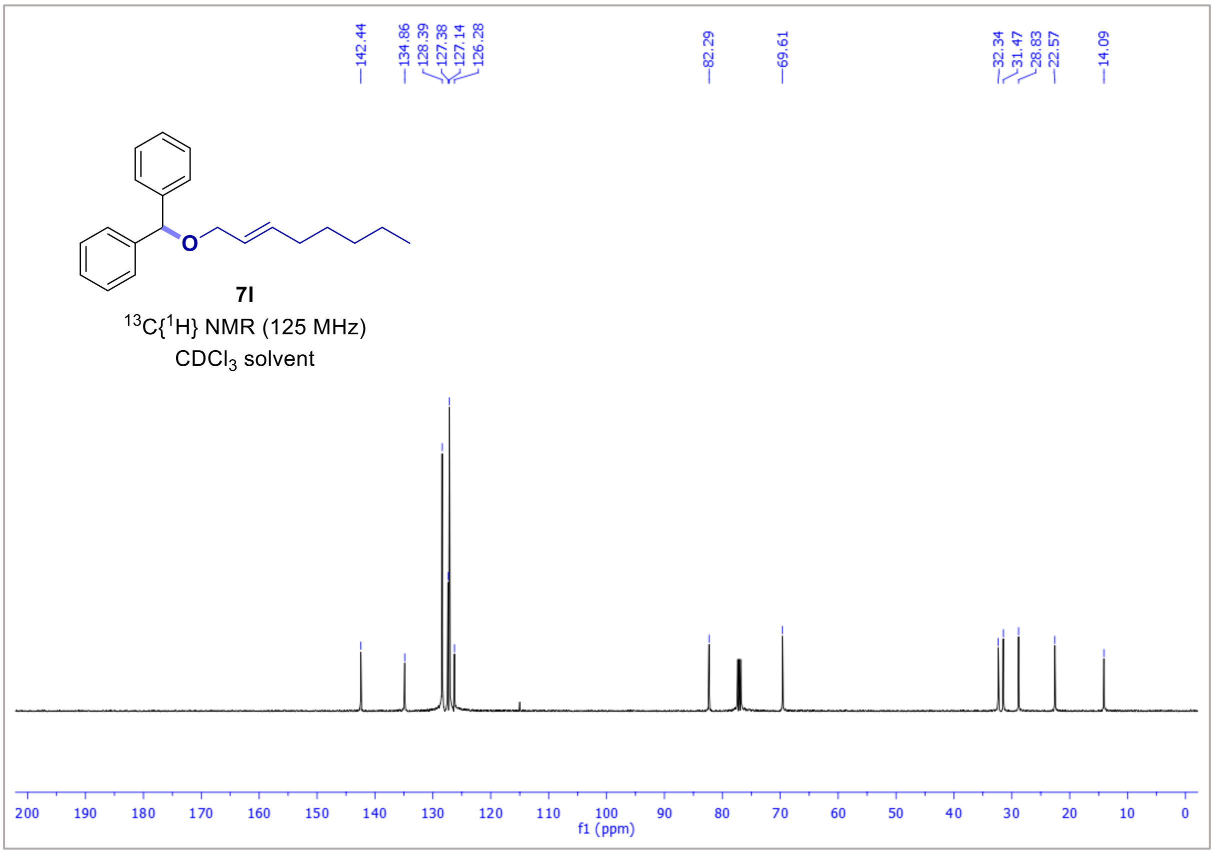

142.44 134.86 128.39 127.38 127.14 126.28 82.29 69.61 32.34 31.47 28.83 22.57 14.09
O
7l
^{13}C{^{1}H} NMR (125 MHz)
$CDCl_3$ solvent
200 190 180 170 160 150 140 130 120 110 100 90 80 70 60 50 40 30 20 10 0
f1 (ppm)

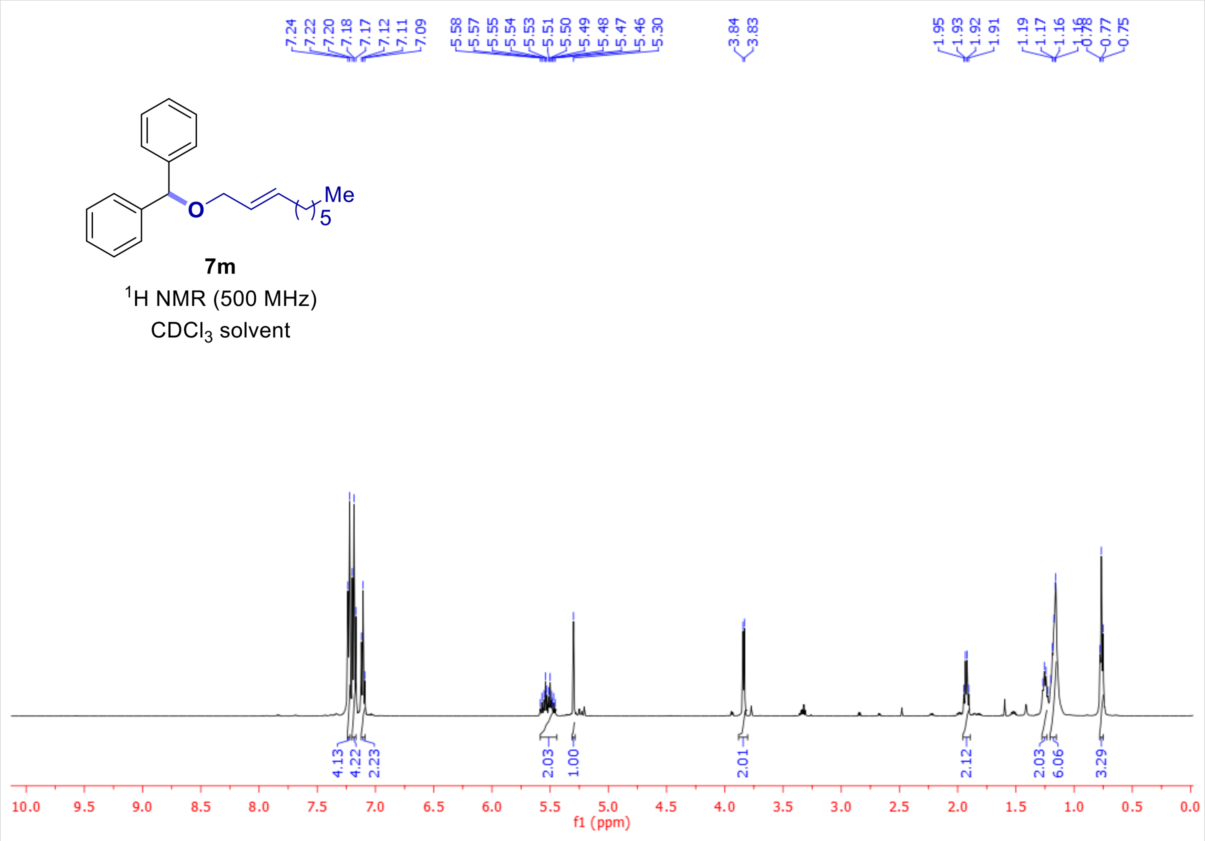

O
Me
5
7m
1H NMR (500 MHz)
CDCl3 solvent
7.24 7.22 7.20 7.18 7.17 7.12 7.11 7.09
5.58 5.57 5.55 5.54 5.53 5.51 5.50 5.49 5.48 5.47 5.46 5.30
3.84 3.83
1.95 1.93 1.92 1.91
1.19 1.17 1.16 1.16 0.78 0.77 0.75
4.13 4.22 2.23 2.03 1.00 2.01 2.12 2.03 6.06 3.29
f1 (ppm)

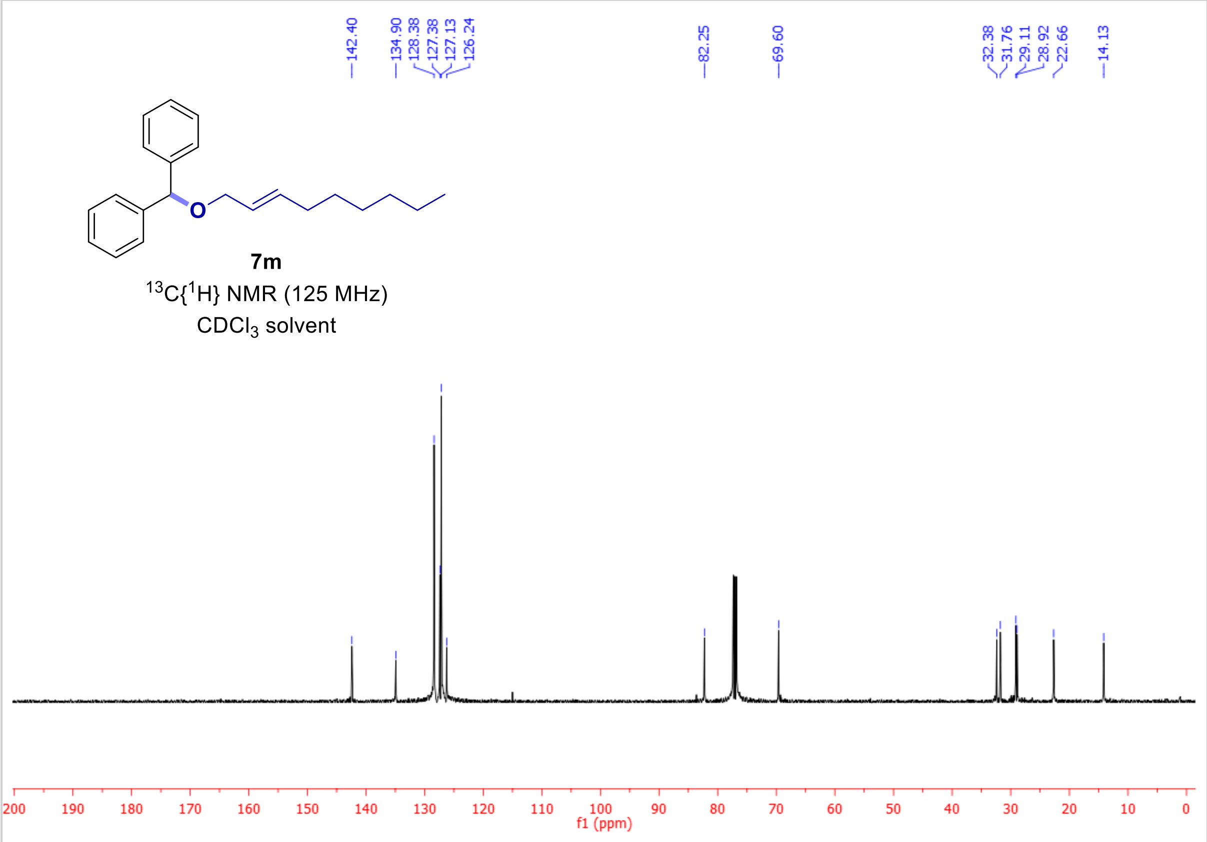

O
7m
13C{1H} NMR (125 MHz)
CDCl3 solvent
142.40
134.90 128.38 127.38 127.13 126.24
82.25
69.60
32.38 31.76 29.11 28.92 22.66
14.13
f1 (ppm)

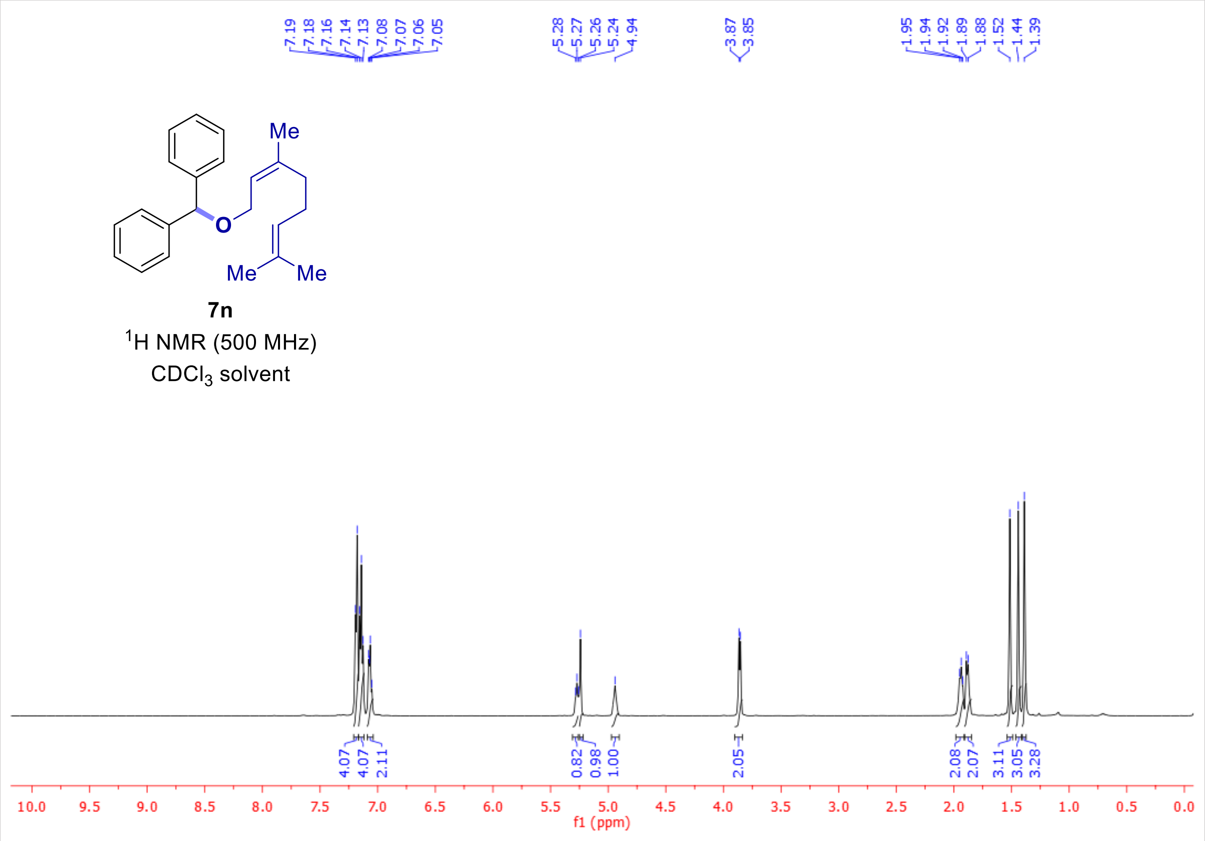
Me
O
Me
Me
7n
1H NMR (500 MHz)
CDCl3 solvent
f1 (ppm)

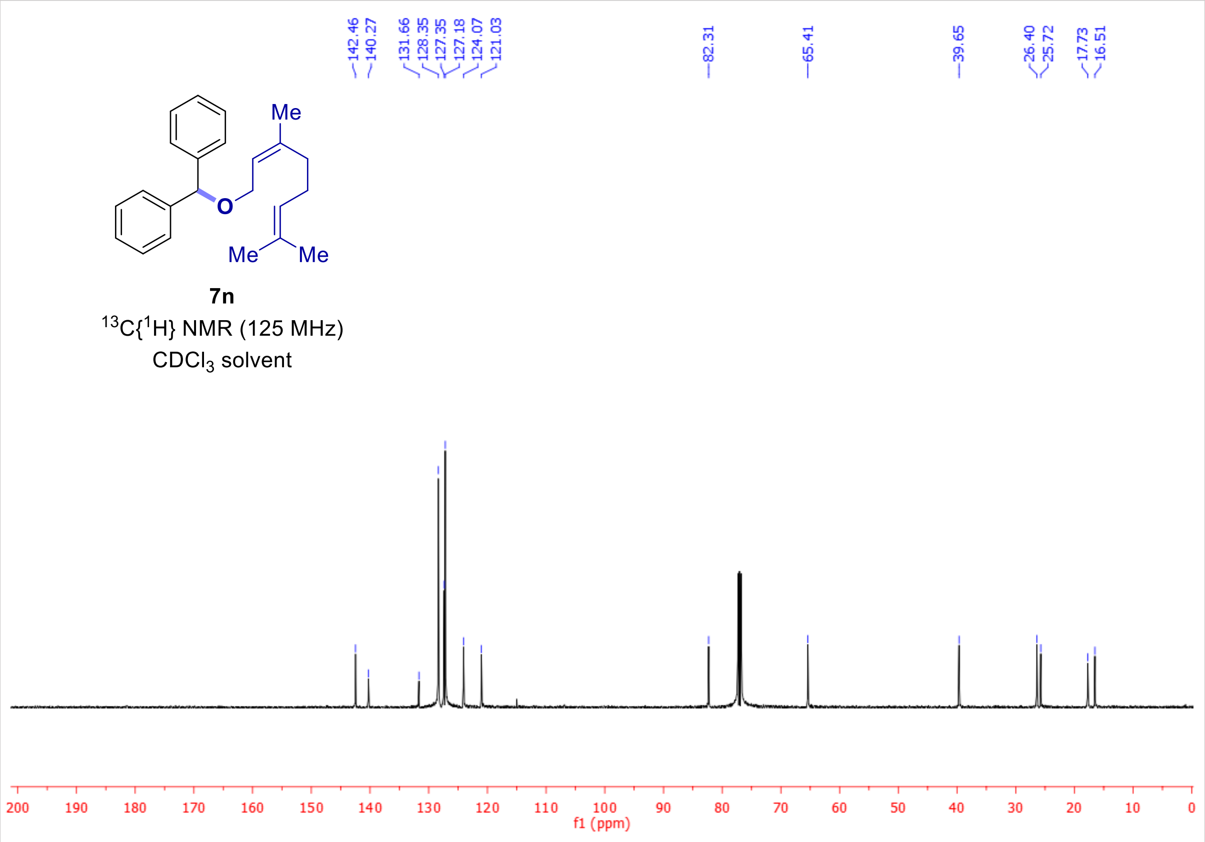
Me
O
Me
Me
7n
13C{1H} NMR (125 MHz)
CDCl3 solvent
f1 (ppm)

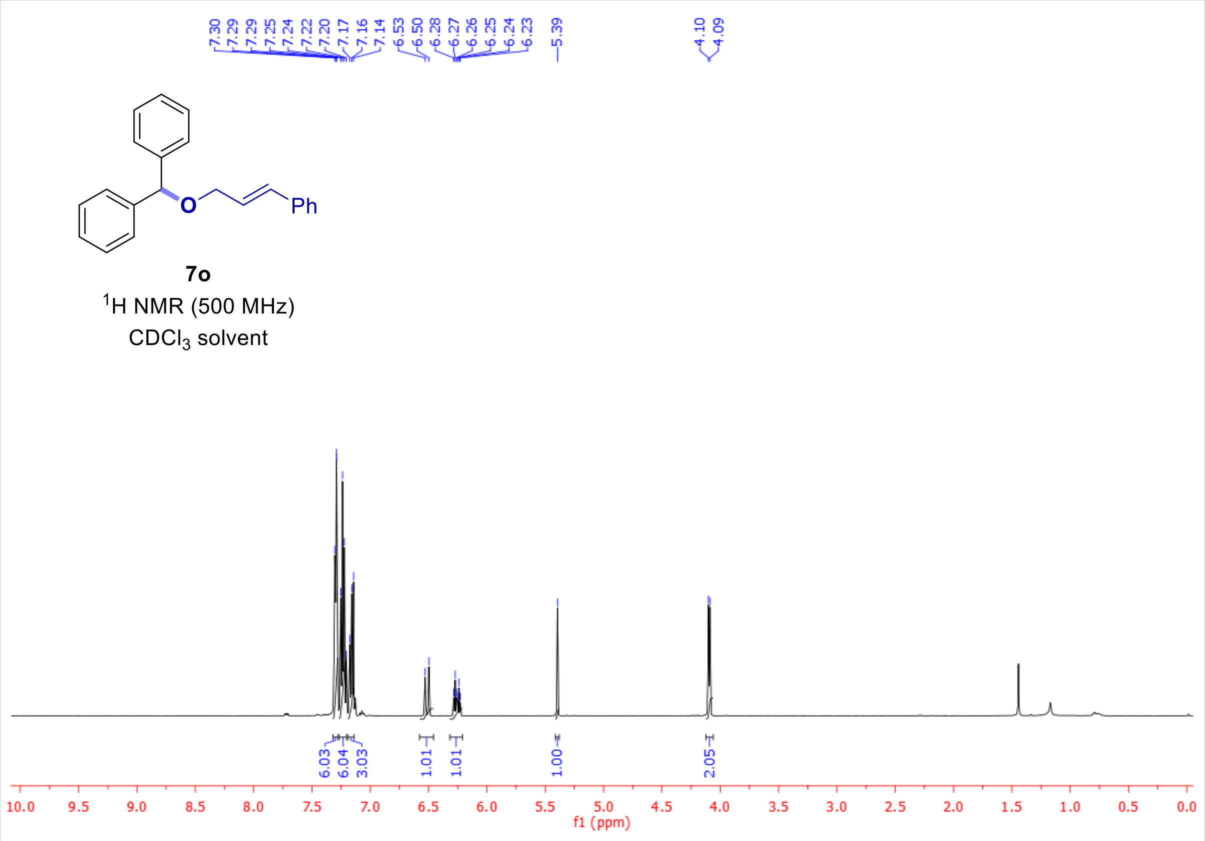
7.30
7.29
7.29
7.25
7.24
7.22
7.20
7.17
7.16
7.14
6.53
6.50
6.28
6.27
6.26
6.25
6.24
6.23
5.39
4.10
4.09
O
Ph
7o
^{1}H NMR (500 MHz)
$CDCl_3$ solvent
6.03
6.04
3.03
1.01
1.01
1.00
2.05
f1 (ppm)

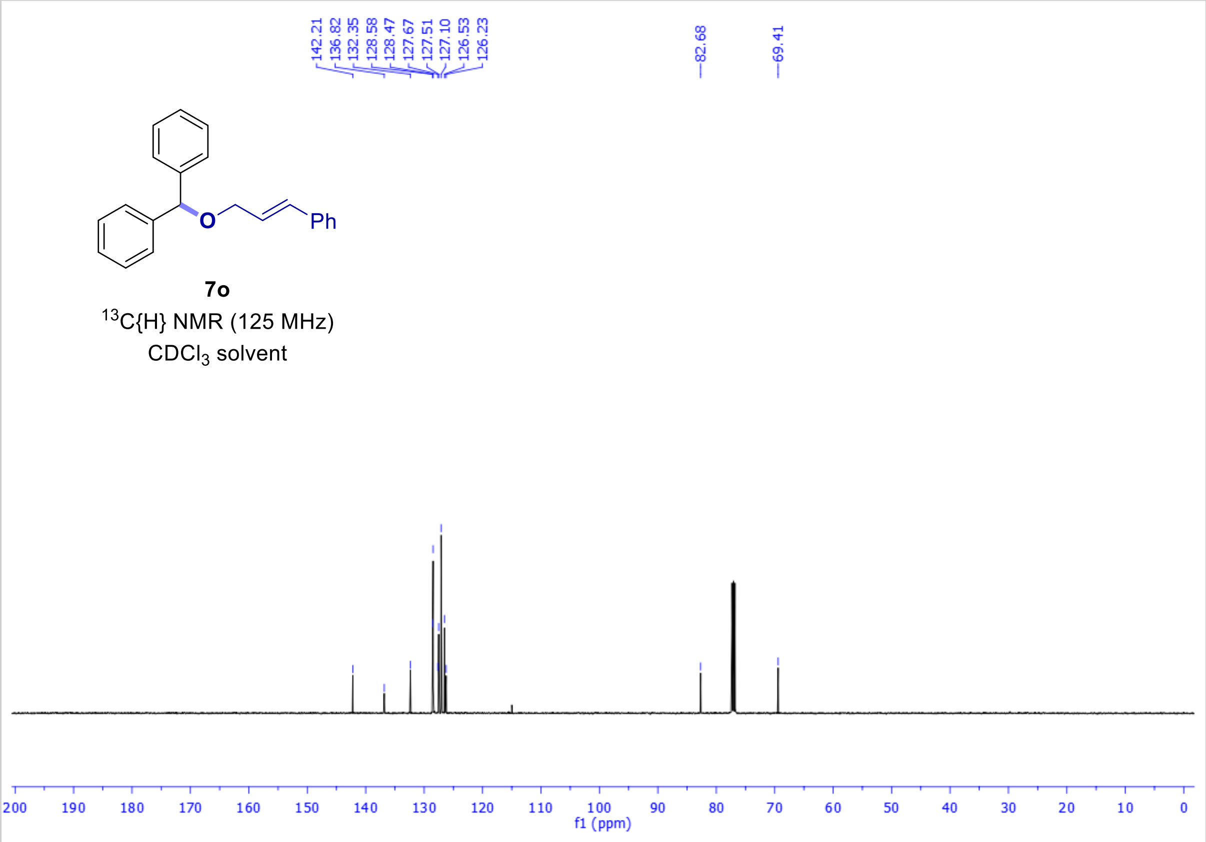
142.21
136.82
132.35
128.58
128.47
127.67
127.51
127.10
126.53
126.23
82.68
69.41
O
Ph
7o
^{13}C{H} NMR (125 MHz)
$CDCl_3$ solvent
f1 (ppm)

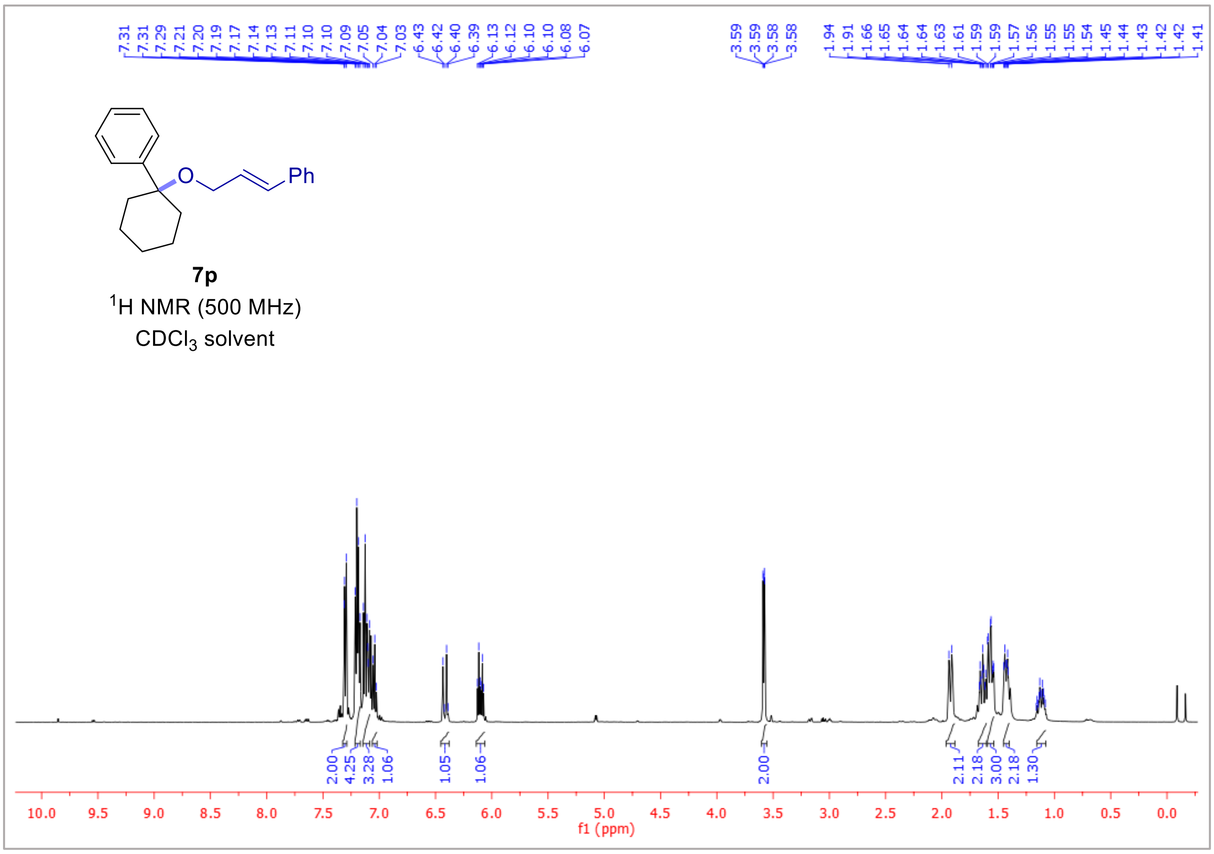
7.31 7.31 7.29 7.21 7.20 7.19 7.17 7.14 7.13 7.11 7.10 7.10 7.09 7.05 7.04 7.03 6.43 6.42 6.40 6.39 6.13 6.12 6.10 6.10 6.08 6.07
3.59 3.59 3.58 3.58
1.94 1.91 1.66 1.65 1.64 1.64 1.63 1.61 1.59 1.59 1.57 1.56 1.55 1.55 1.54 1.45 1.44 1.43 1.42 1.42 1.41
O
Ph
7p
1H NMR (500 MHz)
CDCl3 solvent
2.00 4.25 3.28 1.06 1.05 1.06 2.00 2.11 2.18 3.00 2.18 1.30
10.0 9.5 9.0 8.5 8.0 7.5 7.0 6.5 6.0 5.5 5.0 4.5 4.0 3.5 3.0 2.5 2.0 1.5 1.0 0.5 0.0
f1 (ppm)

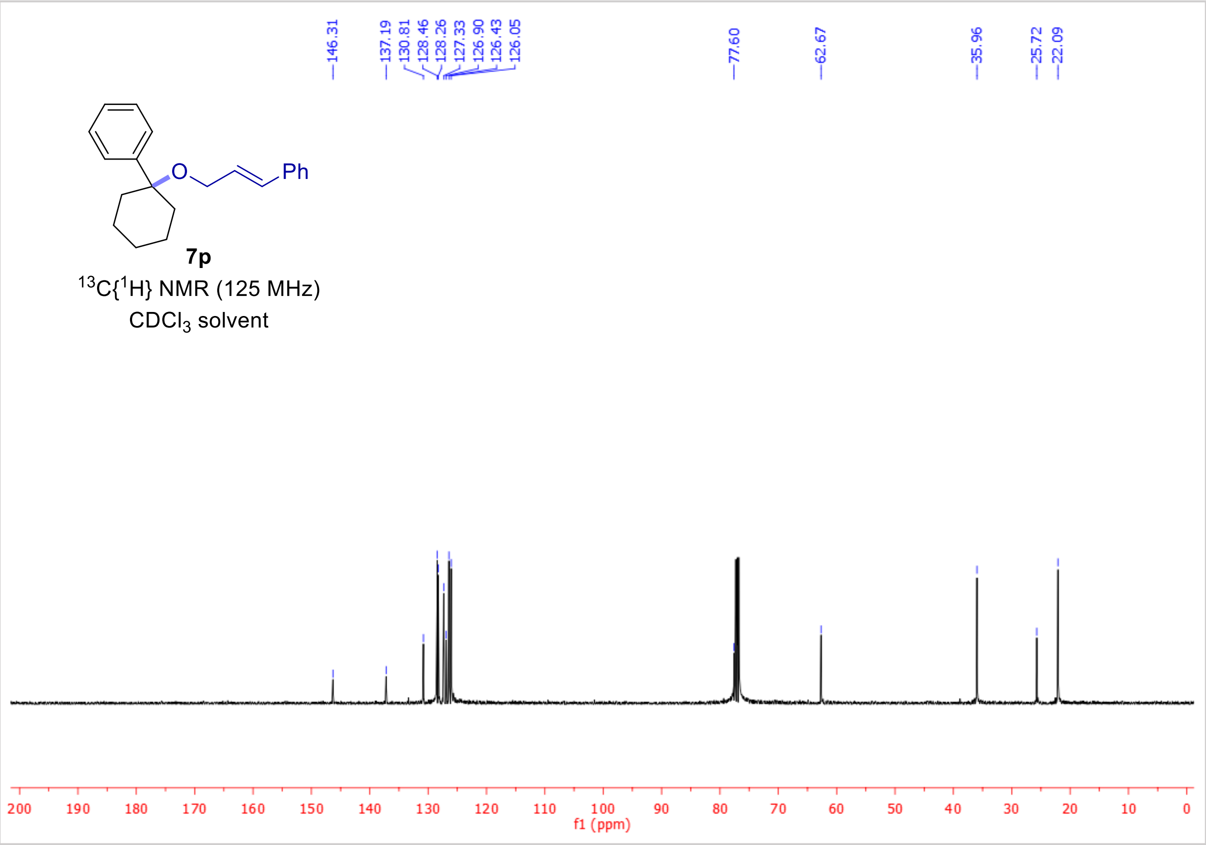
146.31
137.19 130.81 128.46 128.26 127.33 126.90 126.43 126.05
77.60
62.67
35.96
25.72 22.09
O
Ph
7p
13C{1H} NMR (125 MHz)
CDCl3 solvent
200 190 180 170 160 150 140 130 120 110 100 90 80 70 60 50 40 30 20 10 0
f1 (ppm)

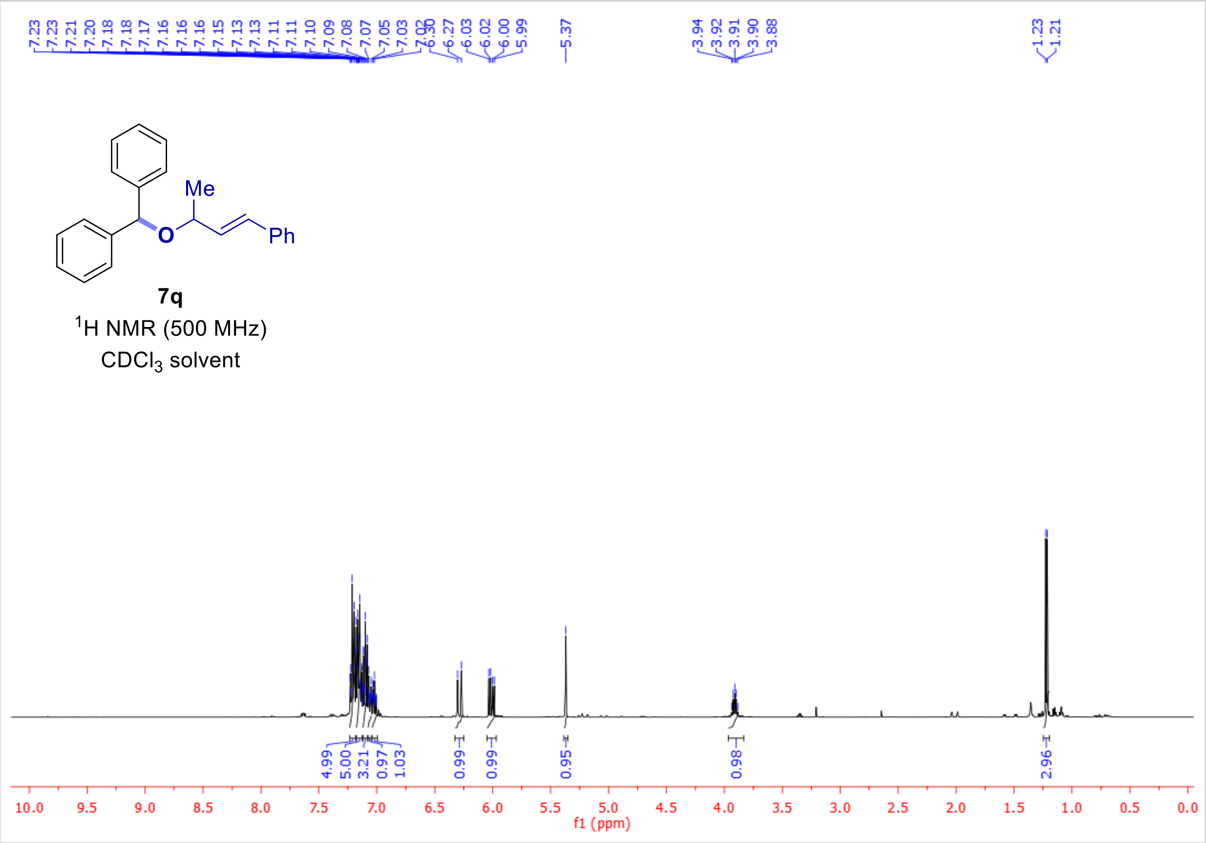
7.23 7.23 7.21 7.20 7.18 7.18 7.17 7.16 7.16 7.16 7.15 7.13 7.13 7.11 7.11 7.10 7.09 7.08 7.07 7.05 7.03 7.02 6.30 6.27 6.03 6.02 6.00 5.99
5.37
3.94 3.92 3.91 3.90 3.88
1.23 1.21
Me
O
Ph
7q
¹H NMR (500 MHz)
CDCl₃ solvent
4.99 5.00 3.21 0.97 1.03
0.99
0.99
0.95
0.98
2.96
10.0 9.5 9.0 8.5 8.0 7.5 7.0 6.5 6.0 5.5 5.0 4.5 4.0 3.5 3.0 2.5 2.0 1.5 1.0 0.5 0.0
f1 (ppm)

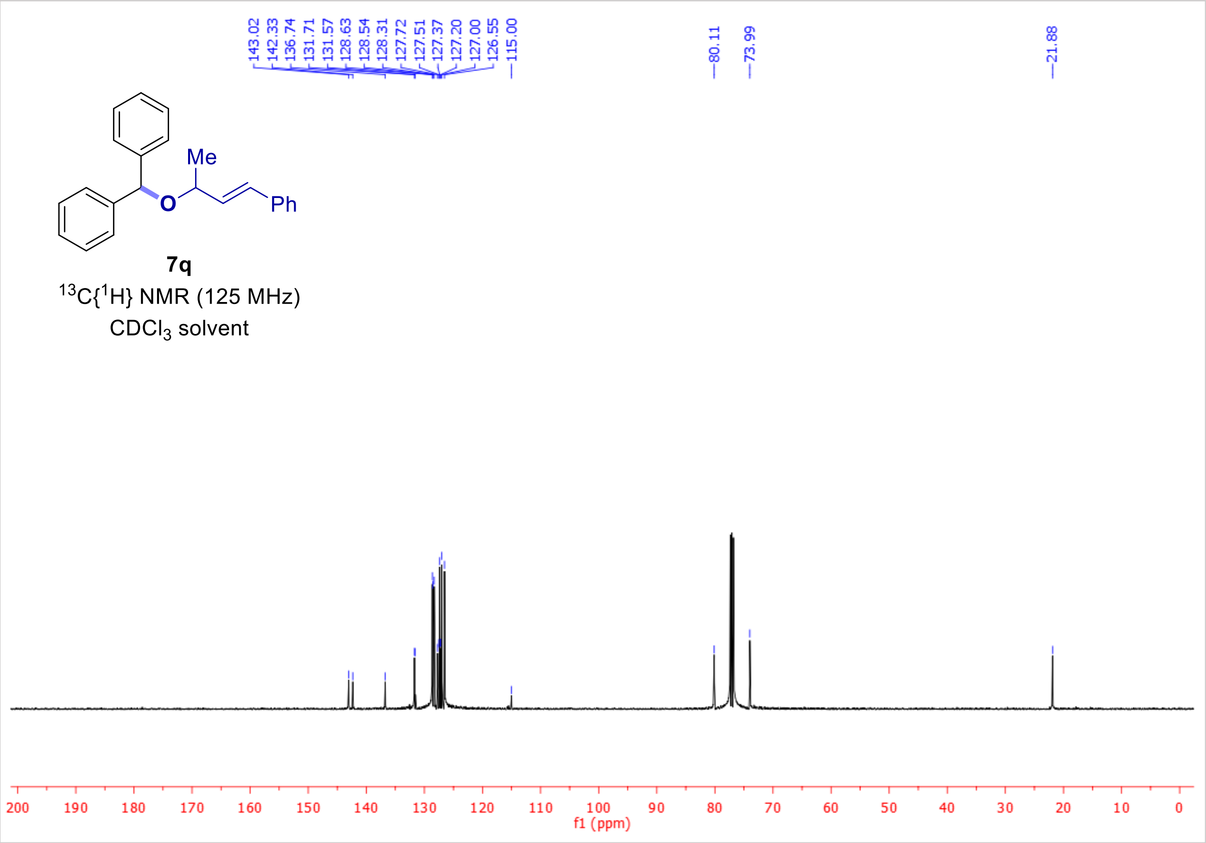
143.02 142.33 136.74 131.71 131.57 128.63 128.54 128.31 127.72 127.51 127.37 127.20 127.00 126.55 115.00
80.11
73.99
21.88
Me
O
Ph
7q
¹³C{¹H} NMR (125 MHz)
CDCl₃ solvent
200 190 180 170 160 150 140 130 120 110 100 90 80 70 60 50 40 30 20 10 0
f1 (ppm)

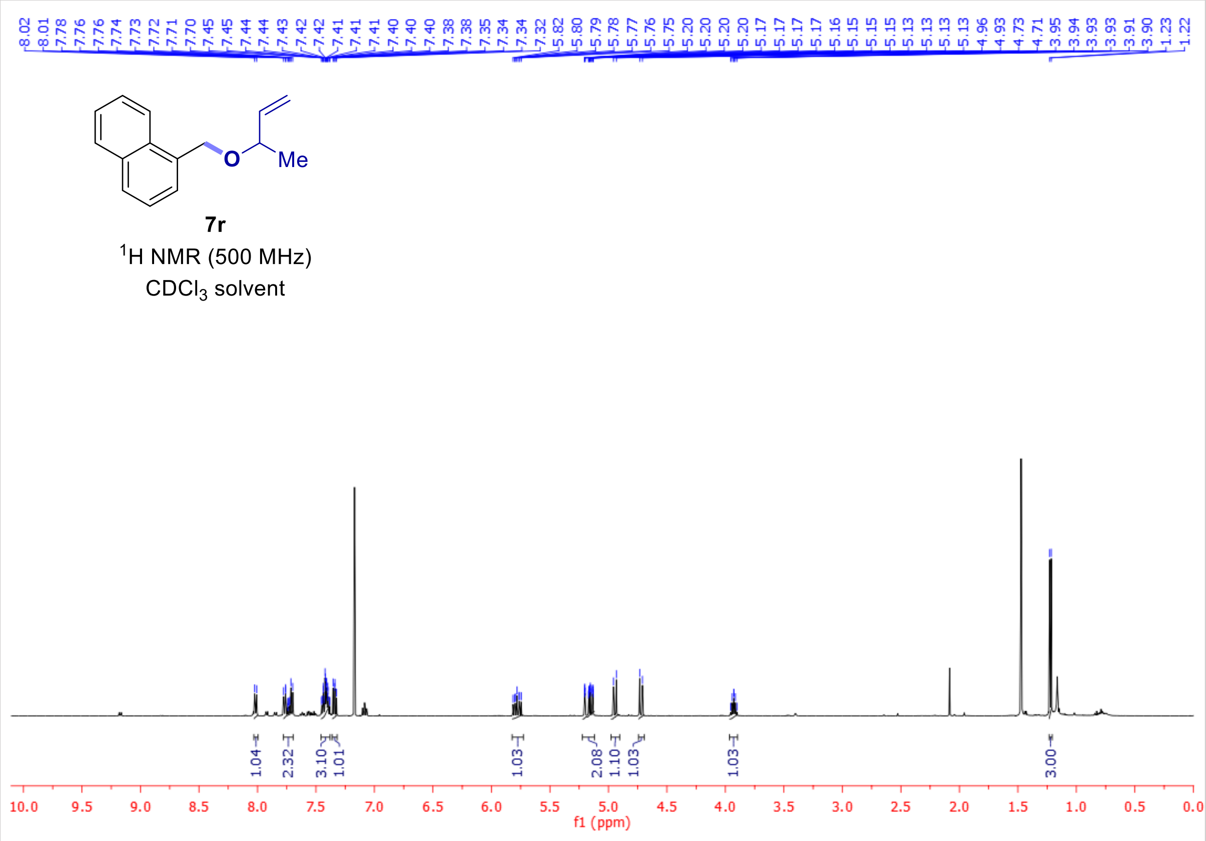
O
Me
7r
1H NMR (500 MHz)
CDCl3 solvent
f1 (ppm)

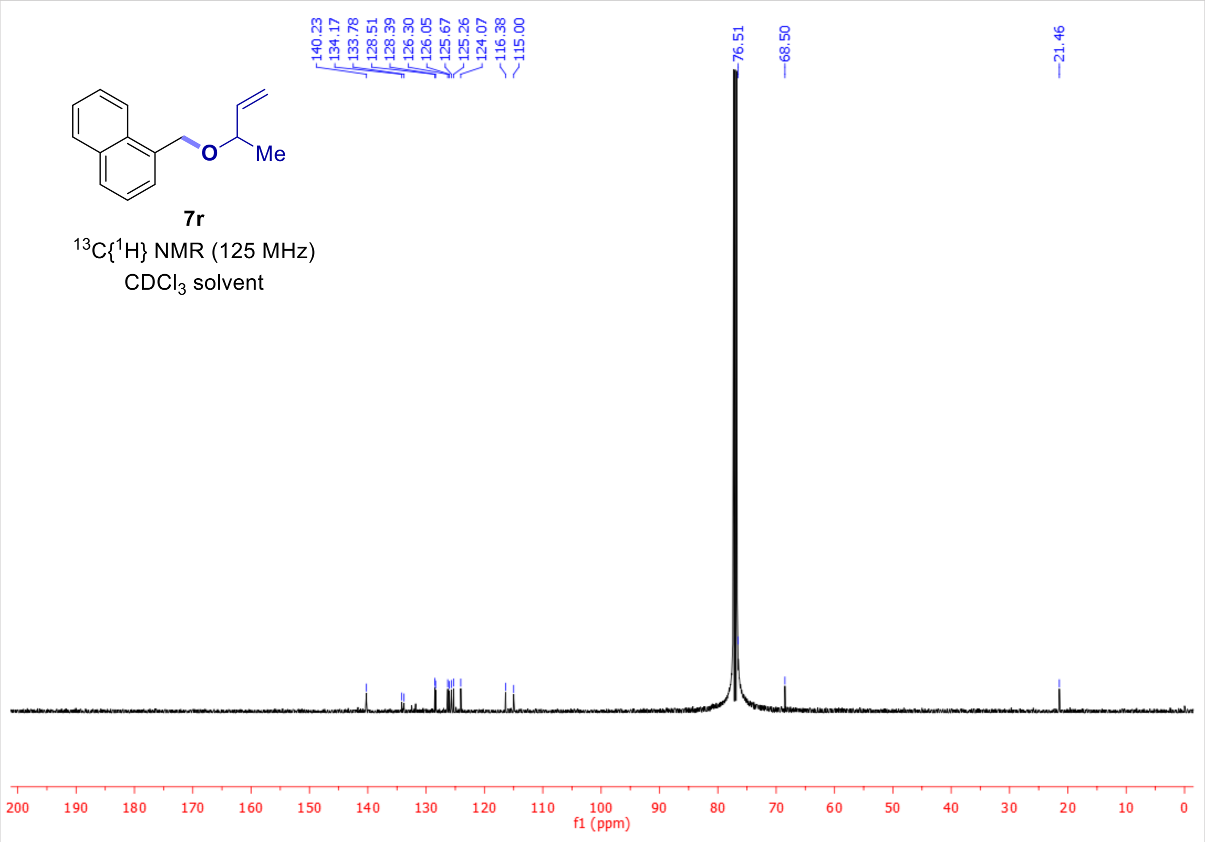
O
Me
7r
13C{1H} NMR (125 MHz)
CDCl3 solvent
140.23
134.17
133.78
128.51
128.39
126.30
126.05
125.67
125.26
124.07
116.38
115.00
76.51
68.50
21.46
f1 (ppm)

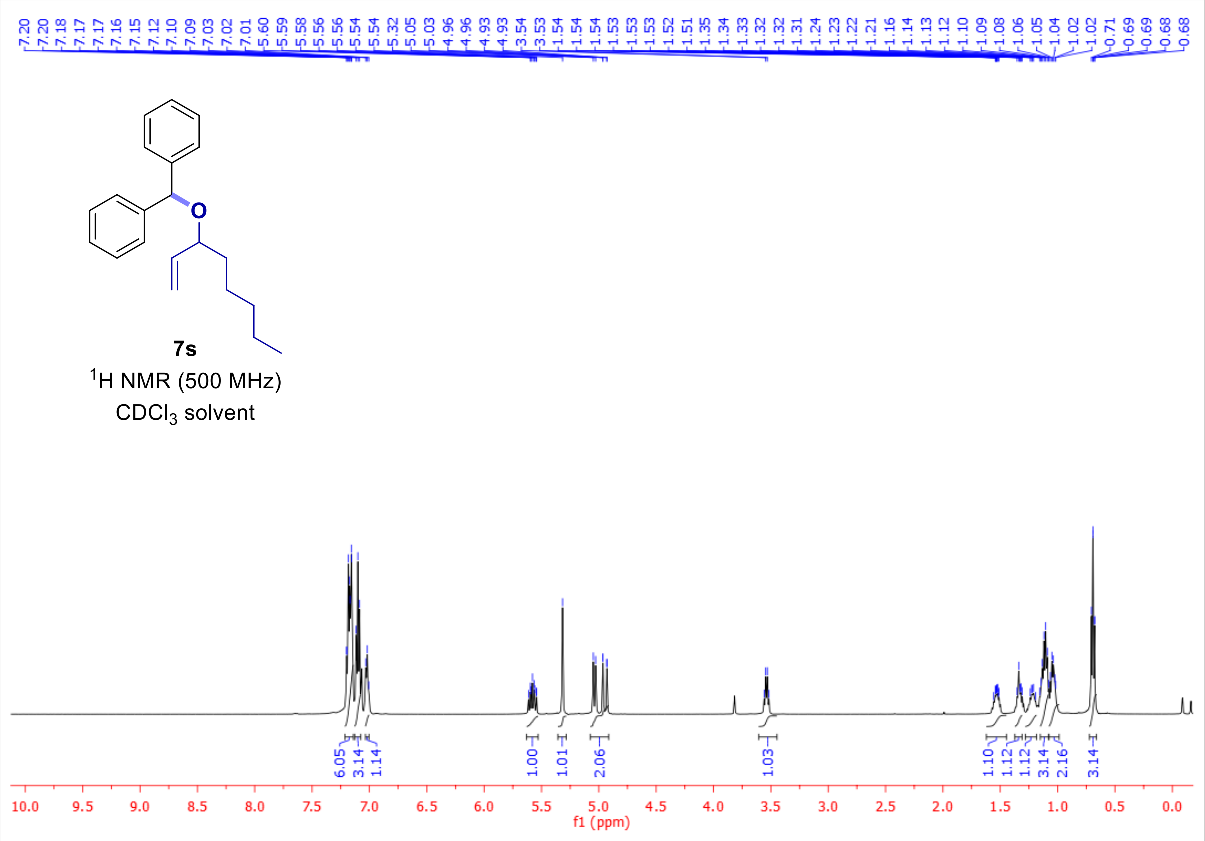
7.20 7.20 7.18 7.17 7.17 7.16 7.15 7.12 7.10 7.09 7.03 7.02 7.01 5.60 5.59 5.58 5.56 5.56 5.54 5.54 5.32 5.05 5.03 4.96 4.96 4.93 4.93 3.54 3.53 1.54 1.54 1.54 1.53 1.53 1.53 1.52 1.51 1.35 1.34 1.33 1.32 1.31 1.24 1.23 1.22 1.21 1.16 1.14 1.13 1.12 1.10 1.09 1.08 1.06 1.05 1.04 1.02 1.02 0.71 0.69 0.69 0.68 0.68
O
7s
1H NMR (500 MHz)
CDCl3 solvent
6.05 3.14 1.14 1.00 1.01 2.06 1.03 1.10 1.12 1.12 3.14 2.16 3.14
10.0 9.5 9.0 8.5 8.0 7.5 7.0 6.5 6.0 5.5 5.0 4.5 4.0 3.5 3.0 2.5 2.0 1.5 1.0 0.5 0.0
f1 (ppm)

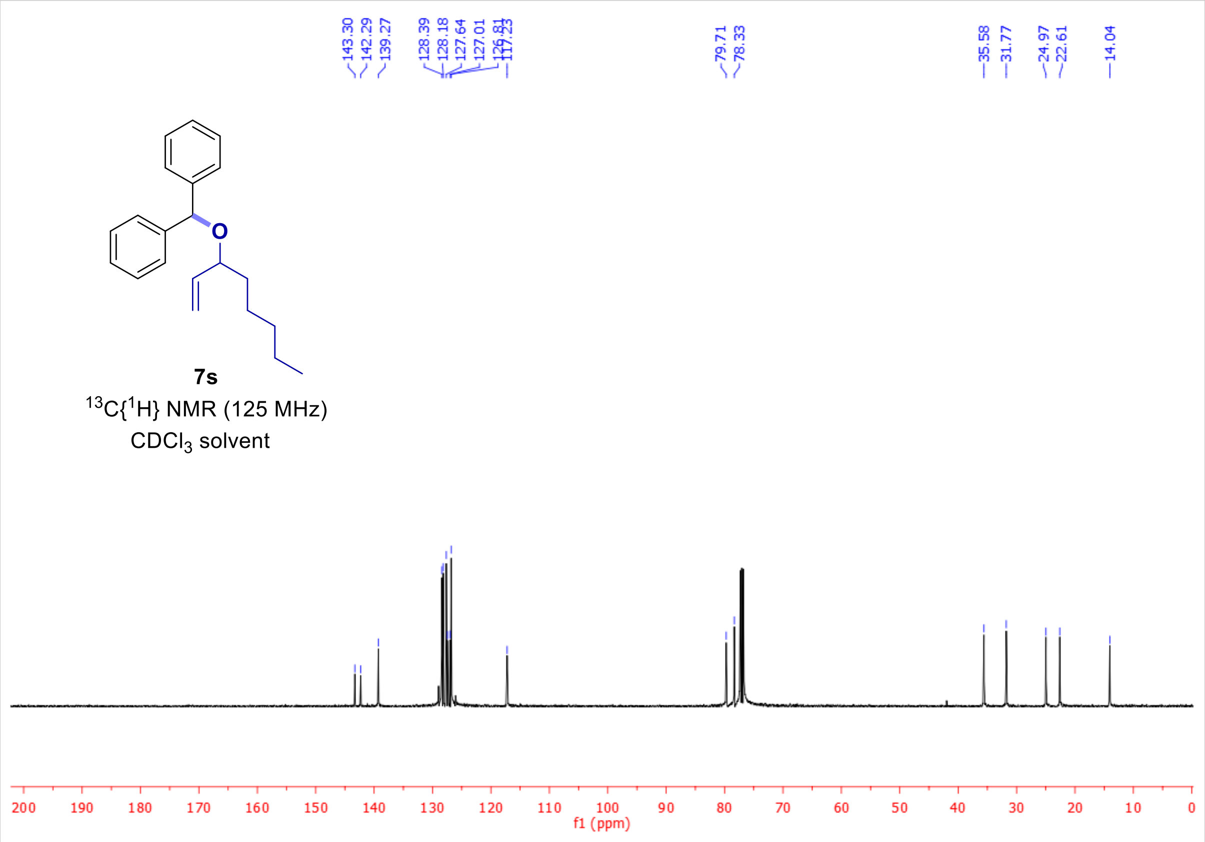
143.30 142.29 139.27 128.39 128.18 127.64 127.01 126.81 117.23 79.71 78.33 35.58 31.77 24.97 22.61 14.04
O
7s
13C{1H} NMR (125 MHz)
CDCl3 solvent
200 190 180 170 160 150 140 130 120 110 100 90 80 70 60 50 40 30 20 10 0
f1 (ppm)

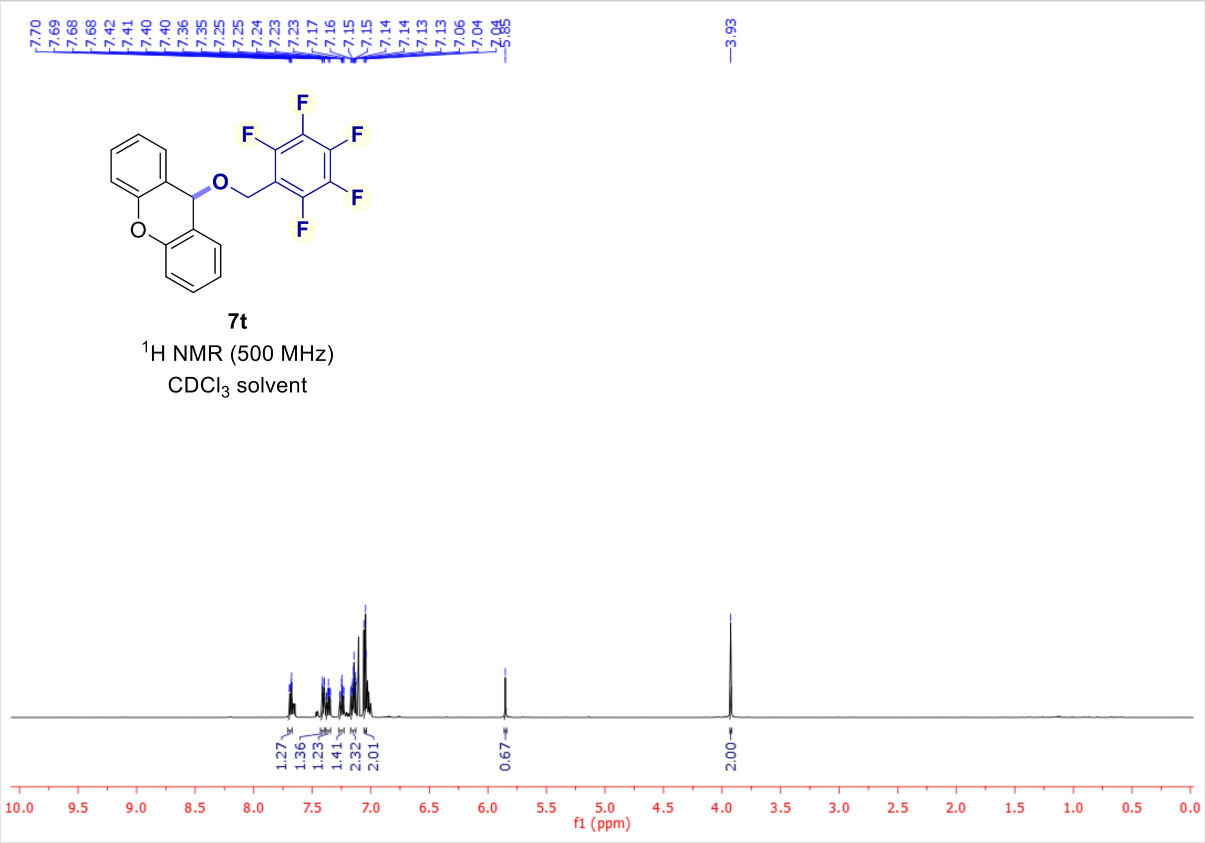
7.70
7.69
7.68
7.42
7.41
7.40
7.40
7.36
7.35
7.25
7.25
7.24
7.23
7.23
7.17
7.16
7.15
7.15
7.14
7.14
7.13
7.13
7.06
7.04
7.04
5.85
3.93
F
F
F
F
F
O
O
7t
1H NMR (500 MHz)
CDCl3 solvent
1.27
1.36
1.23
1.41
2.32
2.01
0.67
2.00
10.0 9.5 9.0 8.5 8.0 7.5 7.0 6.5 6.0 5.5 5.0 4.5 4.0 3.5 3.0 2.5 2.0 1.5 1.0 0.5 0.0
f1 (ppm)

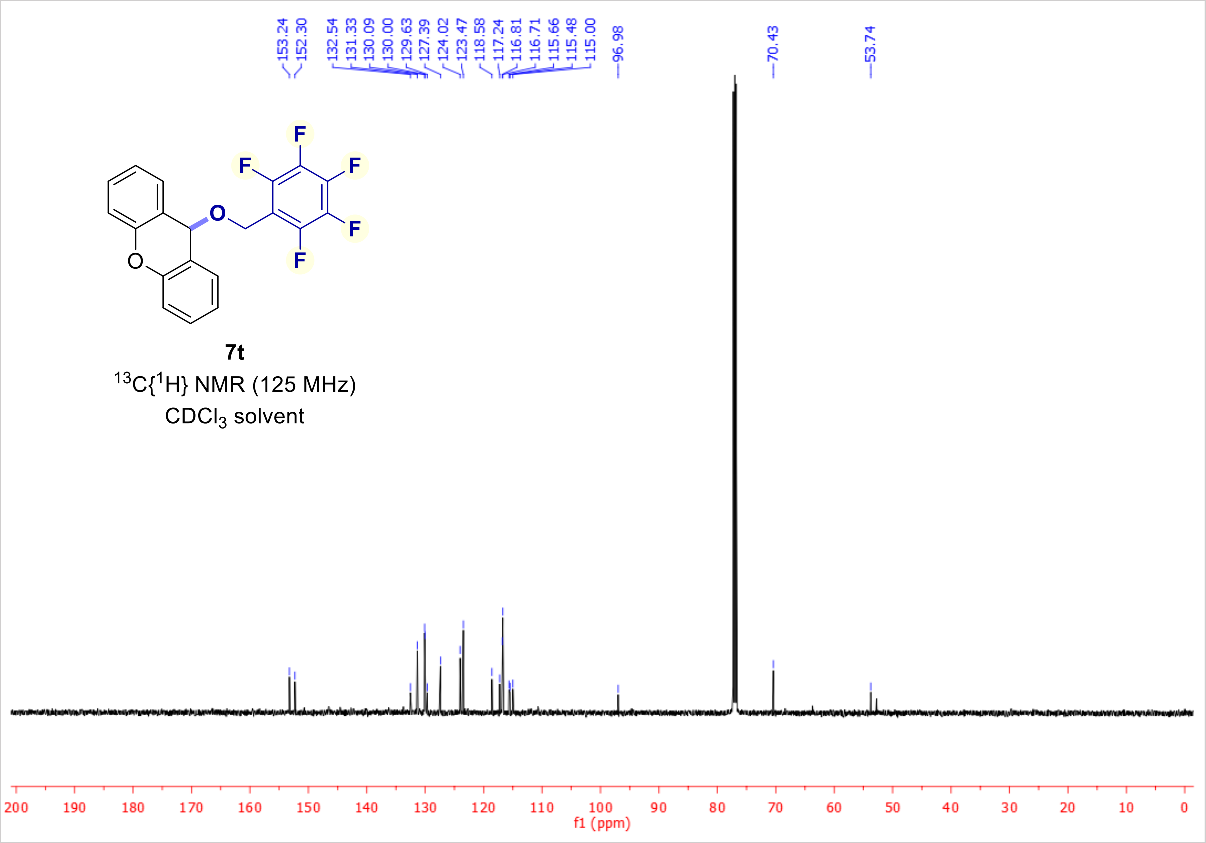
153.24
152.30
132.54
131.33
130.09
130.00
129.63
127.39
124.02
123.47
118.58
117.24
116.81
116.71
115.66
115.48
115.00
96.98
70.43
53.74
F
F
F
F
F
O
O
7t
13C{1H} NMR (125 MHz)
CDCl3 solvent
200 190 180 170 160 150 140 130 120 110 100 90 80 70 60 50 40 30 20 10 0
f1 (ppm)

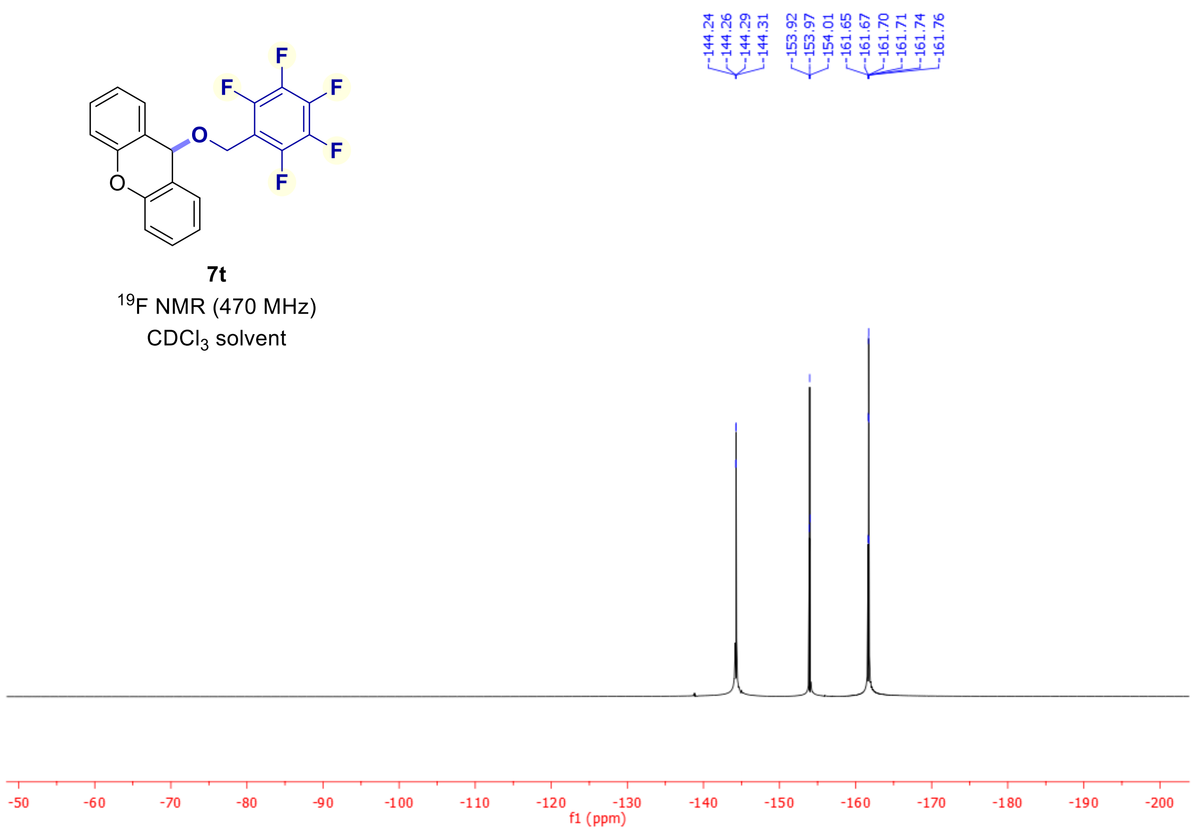

7t

^{19}F NMR (470 MHz)

$CDCl_3$ solvent

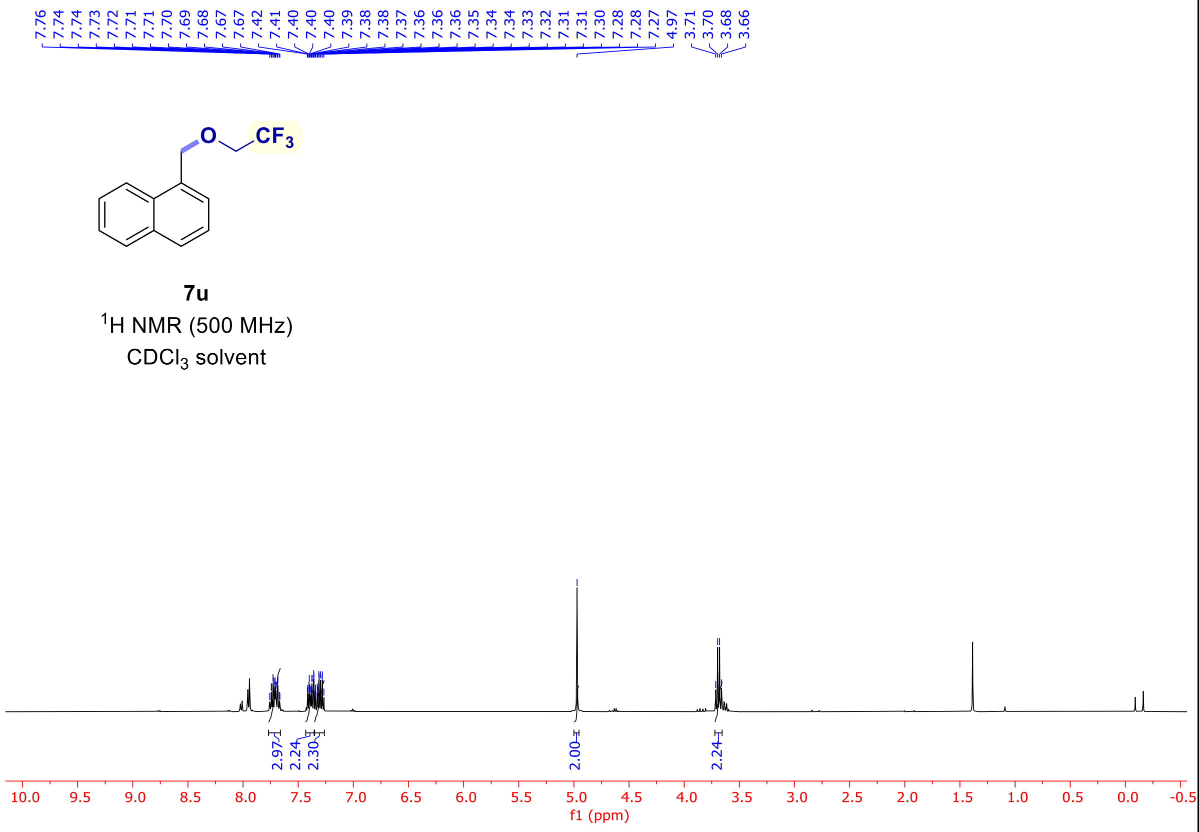

7u

^{1}H NMR (500 MHz)

$CDCl_3$ solvent

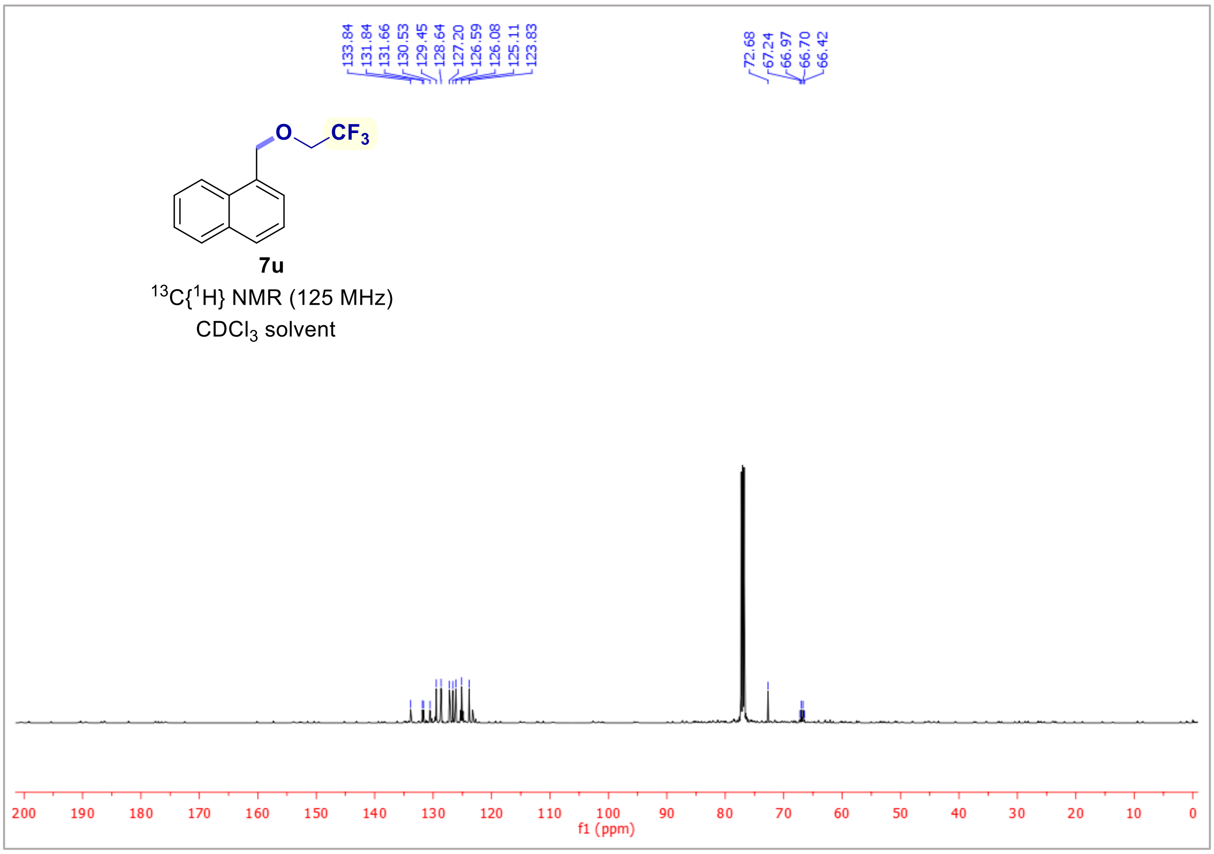
133.84
131.84
131.66
130.53
129.45
128.64
127.20
126.59
126.08
125.11
123.83
72.68
67.24
66.97
66.70
66.42
O
CF3
7u
13C{1H} NMR (125 MHz)
CDCl3 solvent
200
190
180
170
160
150
140
130
120
110
100
90
80
70
60
50
40
30
20
10
0
f1 (ppm)

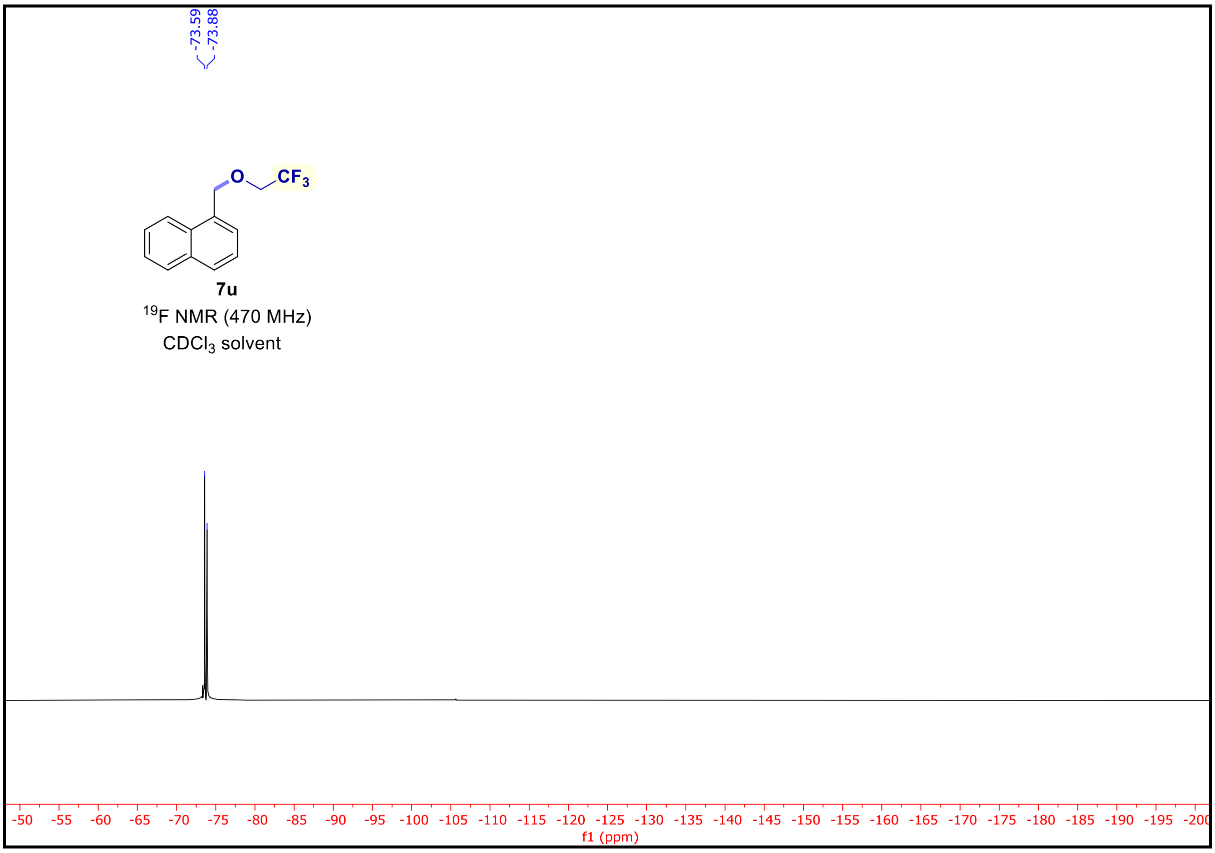
-73.59
-73.88
O
CF3
7u
19F NMR (470 MHz)
CDCl3 solvent
-50
-55
-60
-65
-70
-75
-80
-85
-90
-95
-100
-105
-110
-115
-120
-125
-130
-135
-140
-145
-150
-155
-160
-165
-170
-175
-180
-185
-190
-195
-200
f1 (ppm)

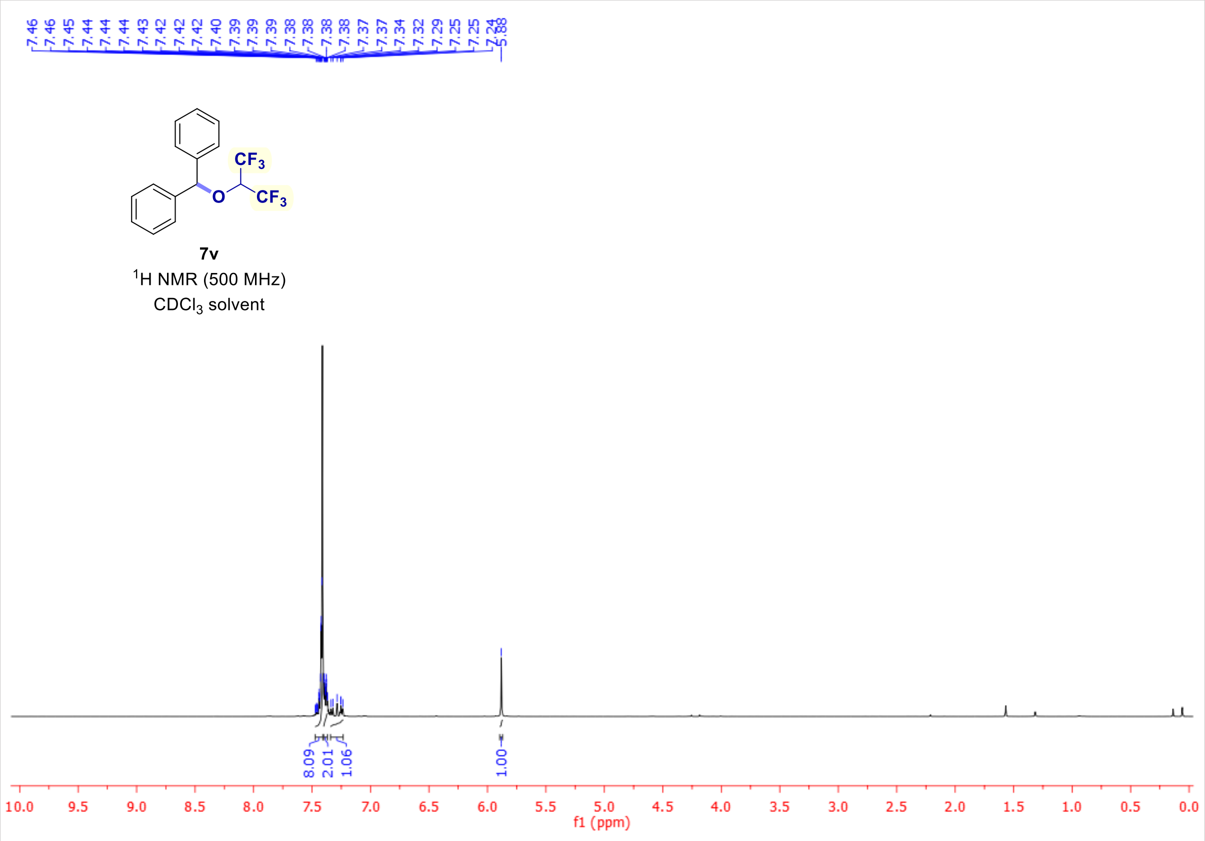
7.46
7.46
7.45
7.44
7.44
7.44
7.43
7.42
7.42
7.42
7.40
7.39
7.39
7.39
7.38
7.38
7.38
7.38
7.37
7.37
7.34
7.32
7.29
7.25
7.25
7.24
5.88
CF3
O
CF3
7v
1H NMR (500 MHz)
CDCl3 solvent
8.09
2.01
1.06
1.00
f1 (ppm)

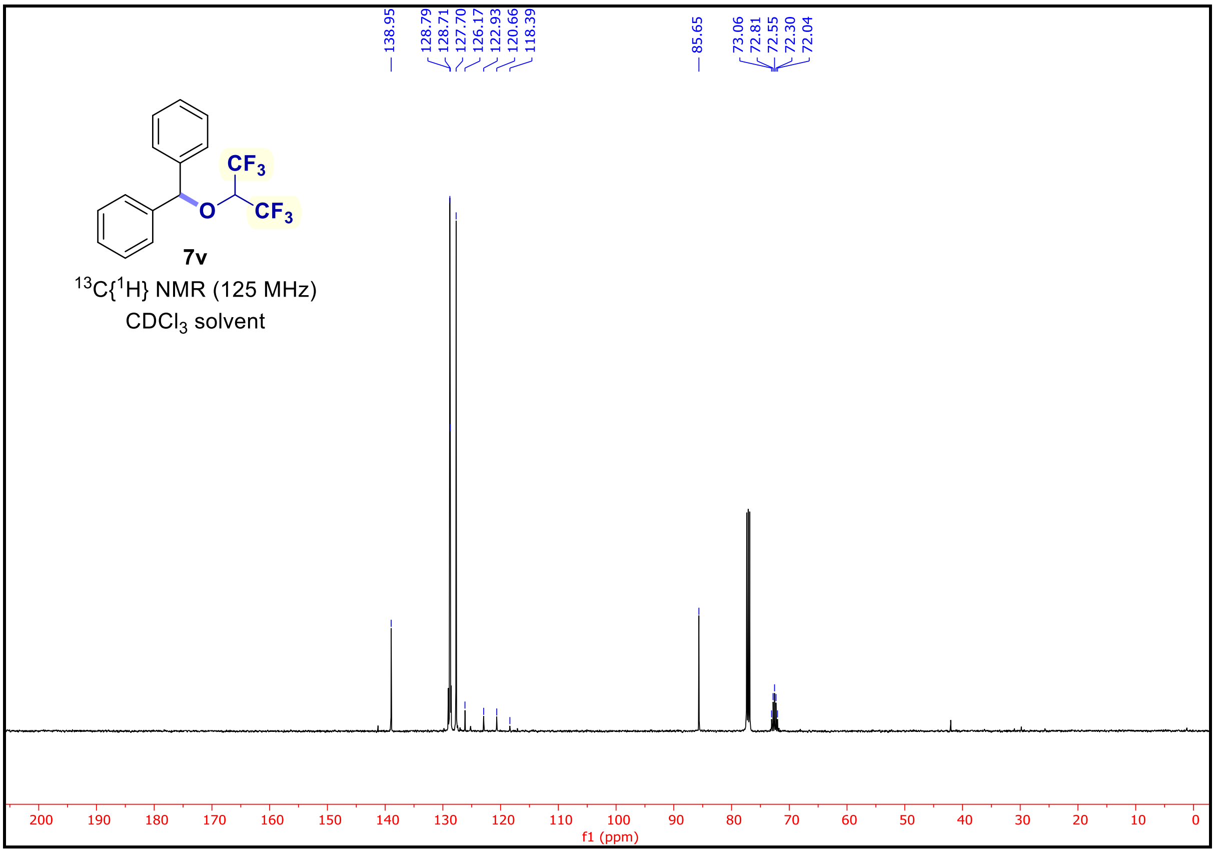
138.95
128.79
128.71
127.70
126.17
122.93
120.66
118.39
85.65
73.06
72.81
72.55
72.30
72.04
CF3
O
CF3
7v
13C{1H} NMR (125 MHz)
CDCl3 solvent
f1 (ppm)

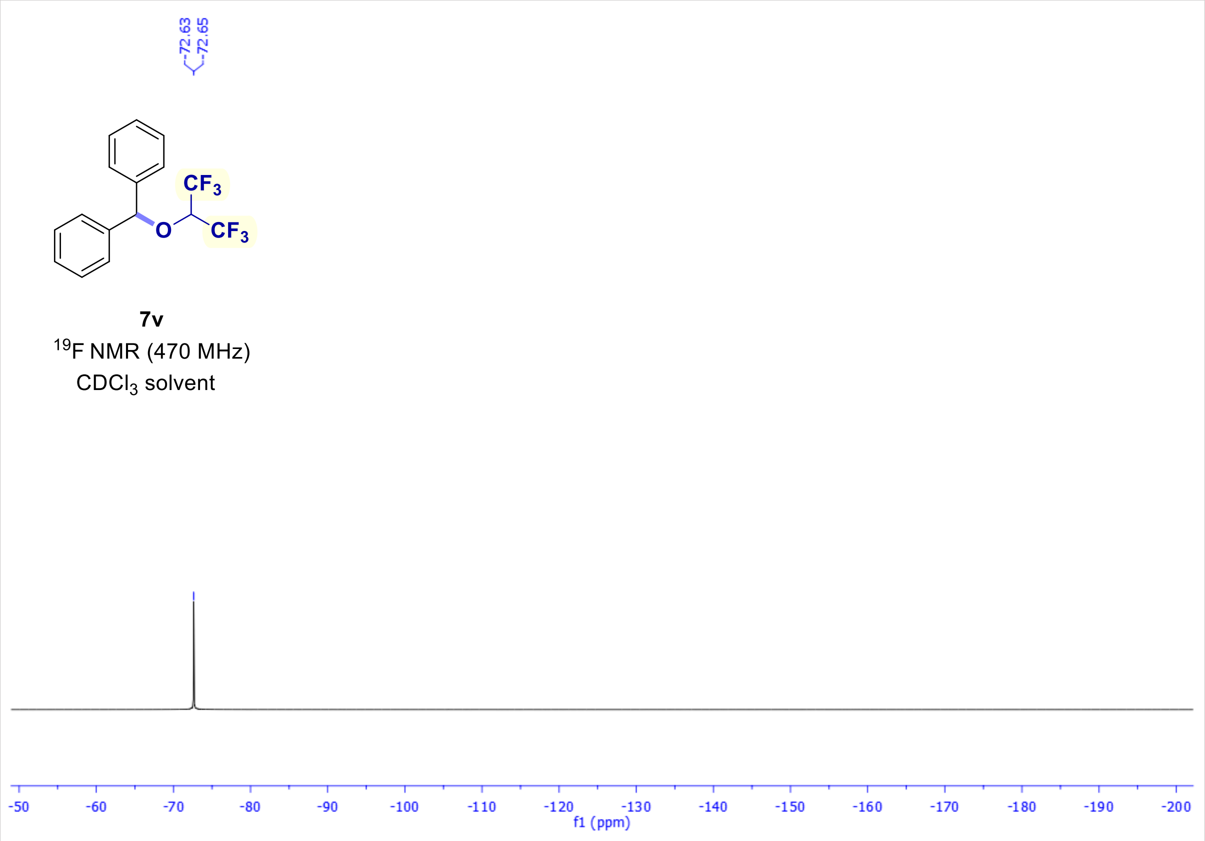

-72.63
-72.65
CF3
O
CF3
7v
19F NMR (470 MHz)
CDCl3 solvent
-50
-60
-70
-80
-90
-100
-110
-120
-130
-140
-150
-160
-170
-180
-190
-200
f1 (ppm)

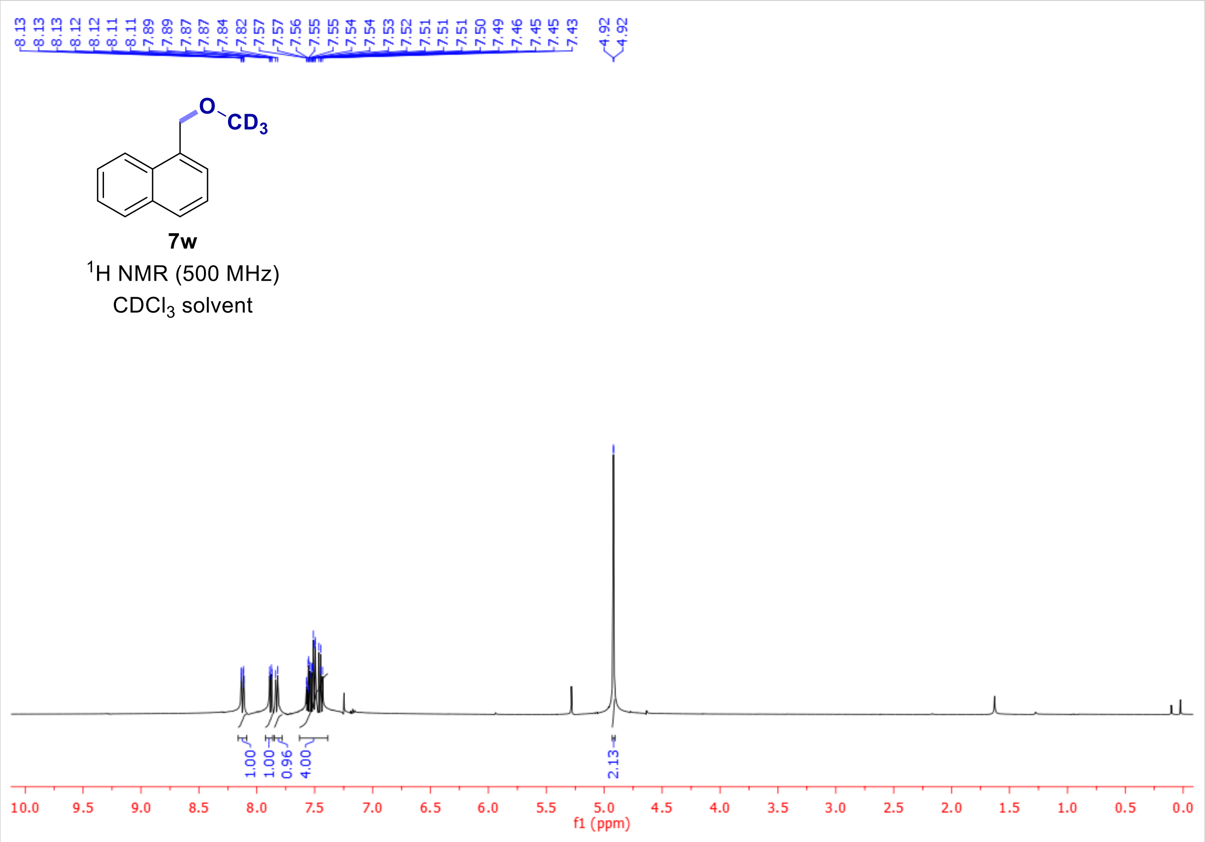

8.13
8.13
8.13
8.12
8.12
8.11
8.11
7.89
7.89
7.87
7.87
7.84
7.82
7.57
7.57
7.56
7.55
7.55
7.54
7.54
7.53
7.52
7.51
7.51
7.51
7.50
7.49
7.46
7.45
7.45
7.43
4.92
4.92
O
CD3
7w
1H NMR (500 MHz)
CDCl3 solvent
1.00
1.00
0.96
4.00
2.13
10.0
9.5
9.0
8.5
8.0
7.5
7.0
6.5
6.0
5.5
5.0
4.5
4.0
3.5
3.0
2.5
2.0
1.5
1.0
0.5
0.0
f1 (ppm)

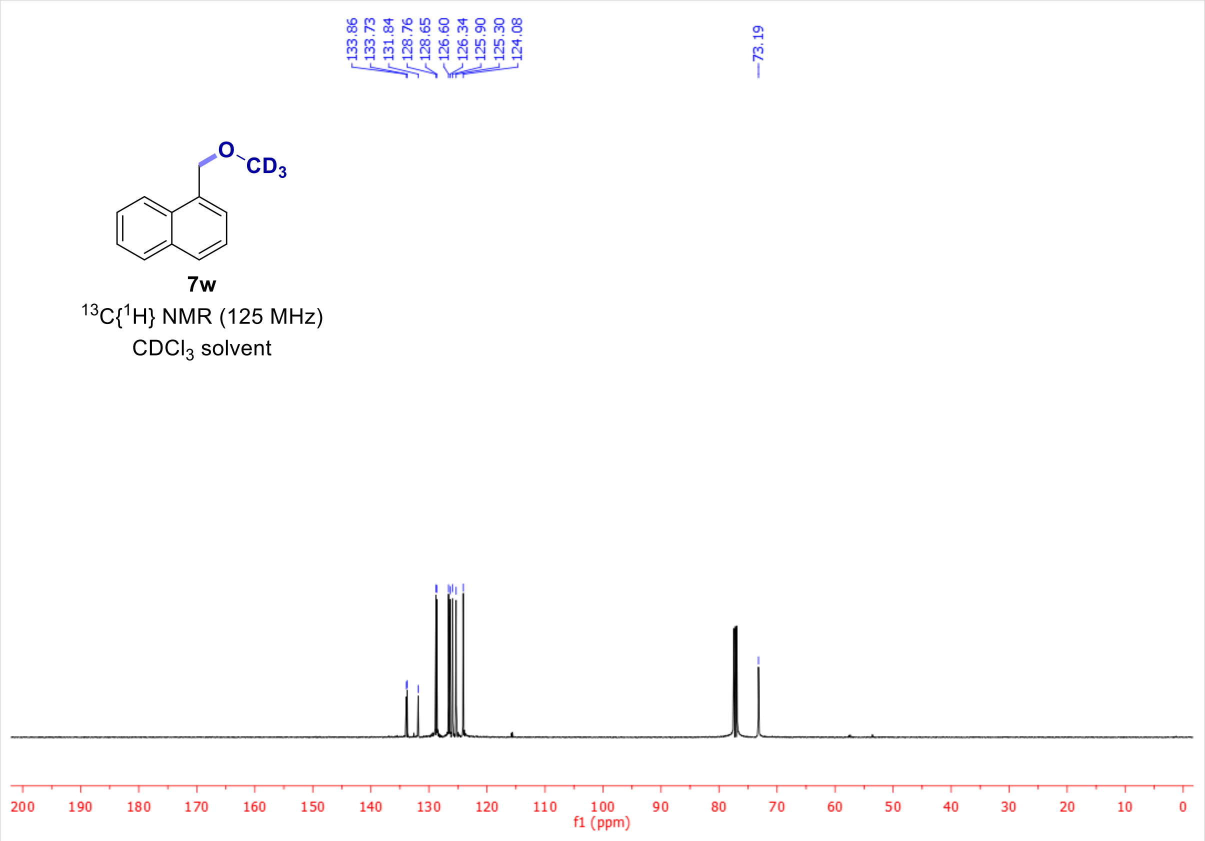
133.86
133.73
131.84
128.76
128.65
126.60
126.34
125.90
125.30
124.08
73.19
7w
$^{13}C\{^1H\}$ NMR (125 MHz)
$CDCl_3$ solvent
f1 (ppm)

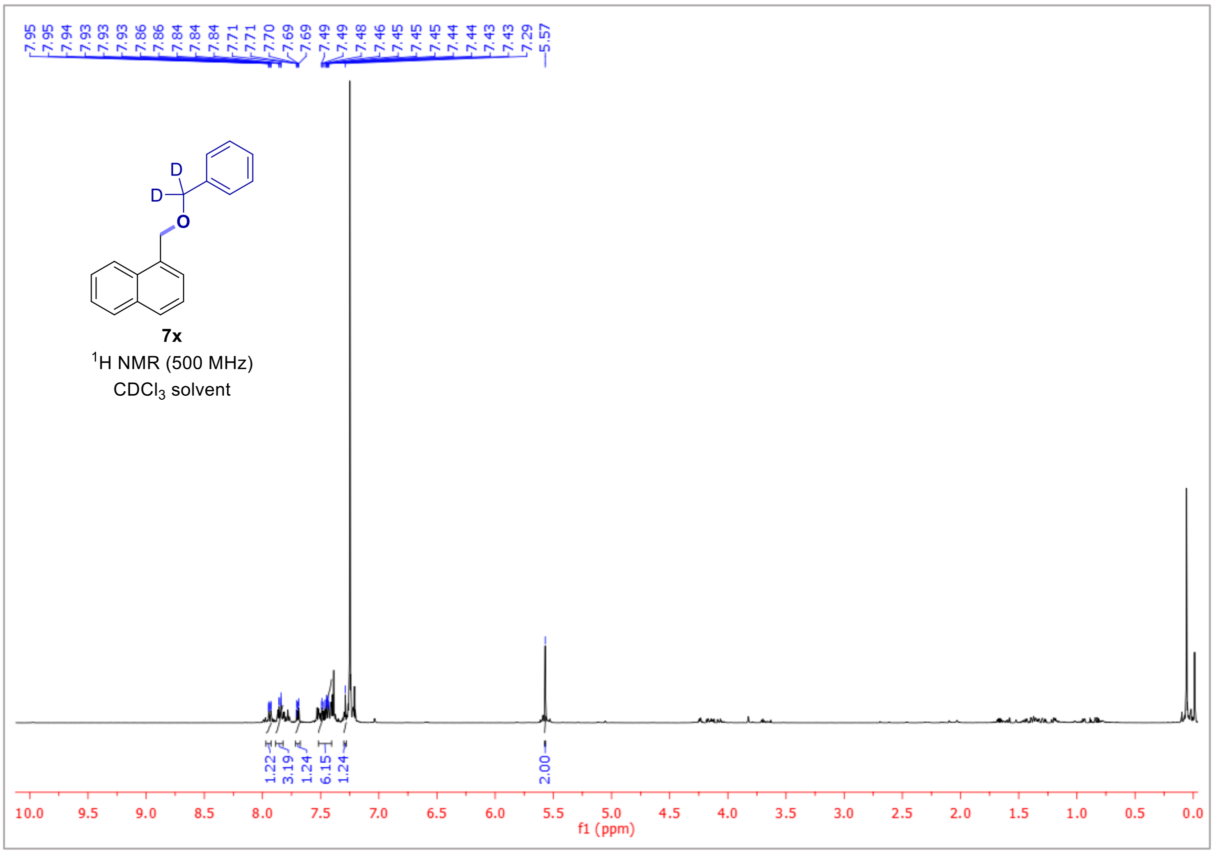
7.95
7.95
7.94
7.93
7.93
7.93
7.86
7.86
7.84
7.84
7.84
7.71
7.71
7.70
7.69
7.69
7.49
7.49
7.48
7.46
7.45
7.45
7.45
7.44
7.44
7.43
7.43
7.29
5.57
7x
^1H NMR (500 MHz)
$CDCl_3$ solvent
1.22
3.19
1.24
6.15
1.24
2.00
f1 (ppm)

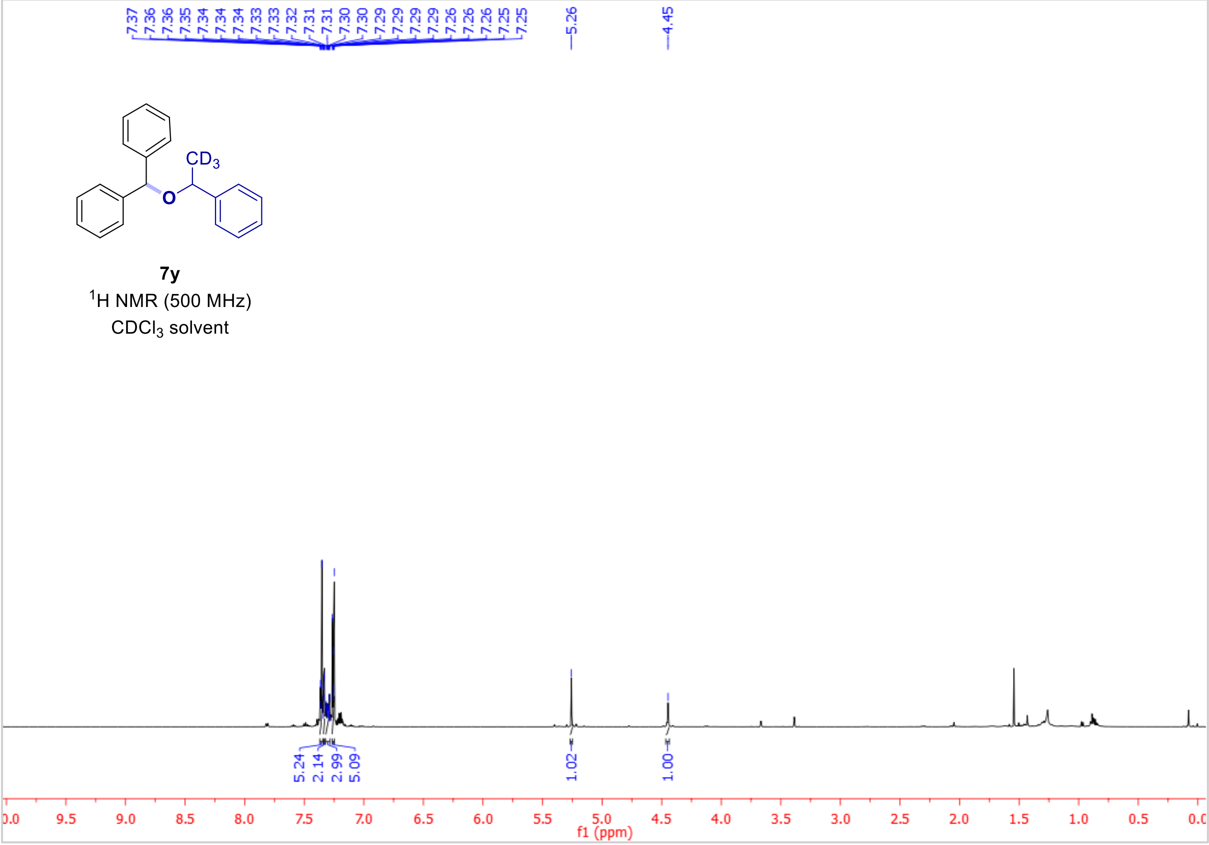
7.37
7.36
7.36
7.35
7.34
7.34
7.34
7.33
7.33
7.32
7.31
7.31
7.30
7.30
7.29
7.29
7.29
7.29
7.26
7.26
7.26
7.25
7.25
5.26
4.45
CD3
O
7y
1H NMR (500 MHz)
CDCl3 solvent
5.24
2.14
2.99
5.09
1.02
1.00
f1 (ppm)

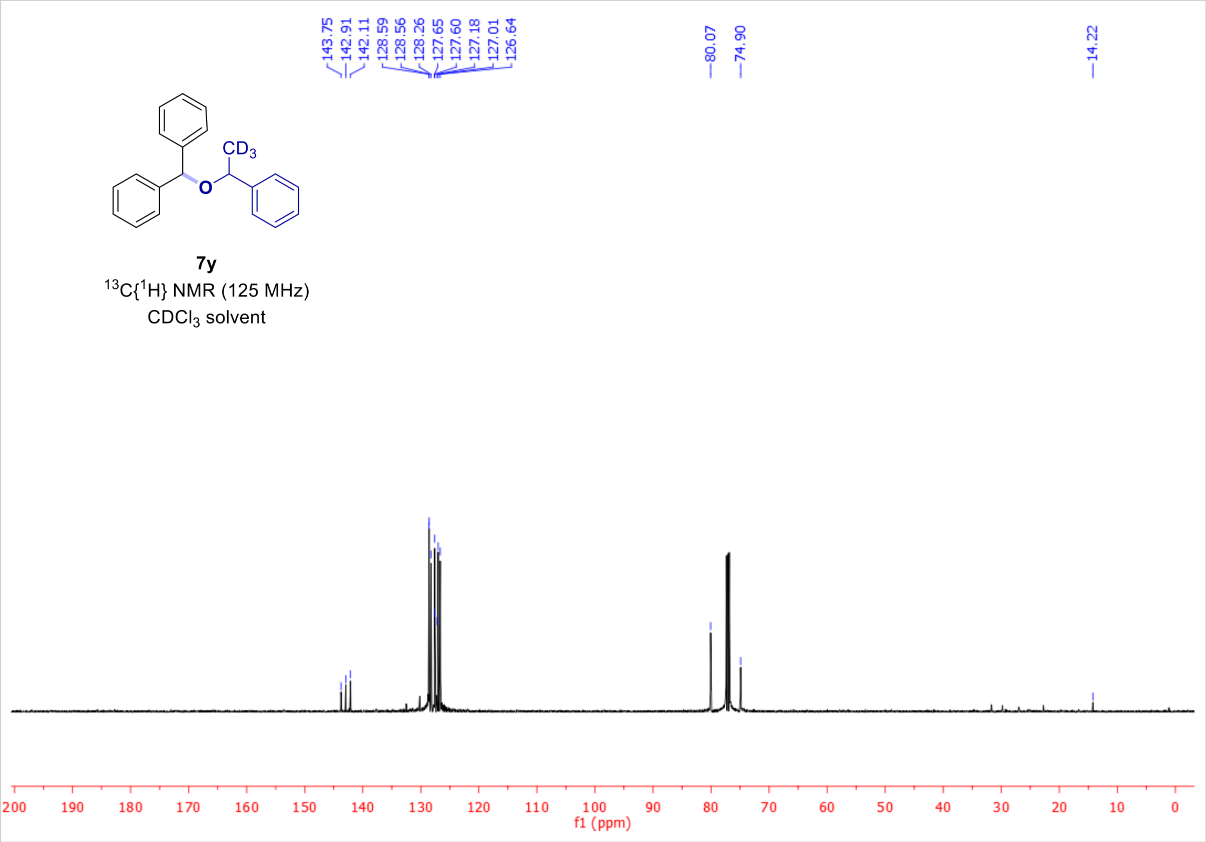
143.75
142.91
142.11
128.59
128.56
128.26
127.65
127.60
127.18
127.01
126.64
80.07
74.90
14.22
CD3
O
7y
13C{1H} NMR (125 MHz)
CDCl3 solvent
f1 (ppm)

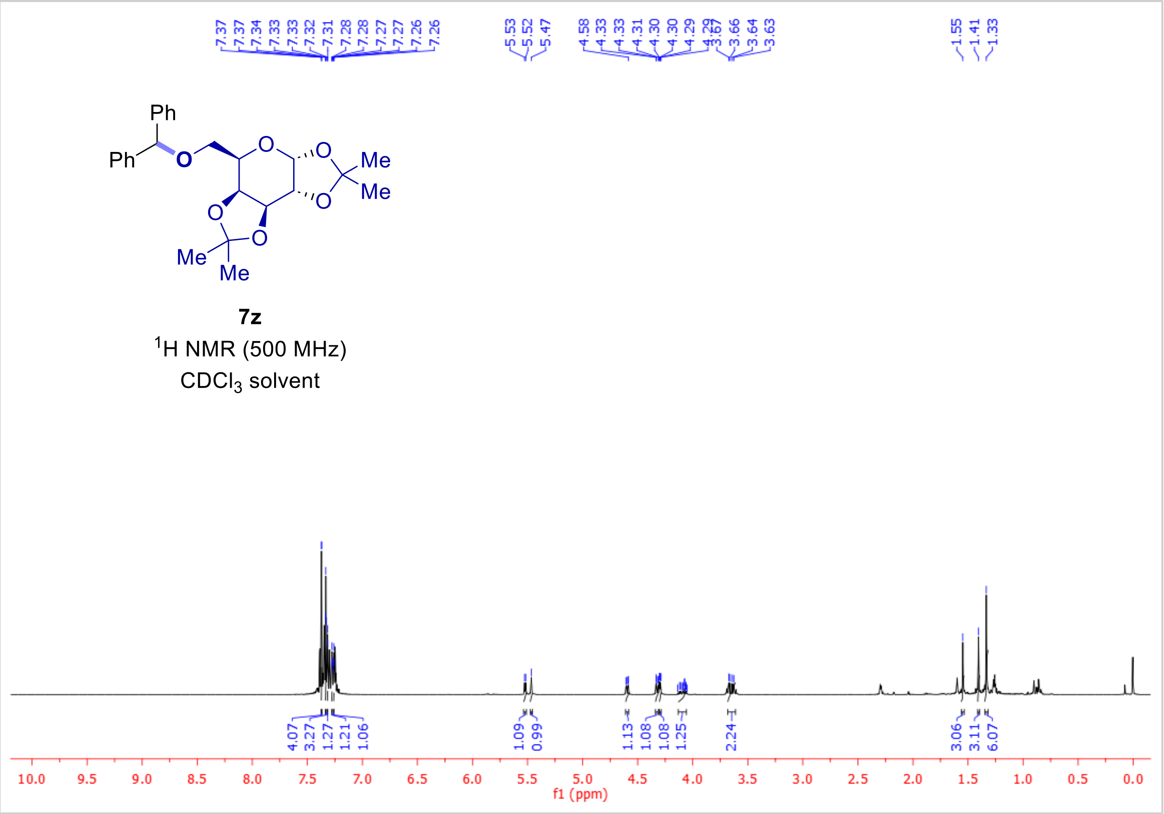
7.37
7.37
7.34
7.33
7.33
7.32
7.31
7.28
7.28
7.27
7.27
7.26
7.26
5.53
5.52
5.47
4.58
4.33
4.33
4.31
4.30
4.30
4.29
4.29
3.67
3.66
3.64
3.63
1.55
1.41
1.33
Ph
Ph
O
O
O
O
O
O
Me
Me
Me
Me
7z
1H NMR (500 MHz)
CDCl3 solvent
4.07
3.27
1.27
1.21
1.06
1.09
0.99
1.13
1.08
1.08
1.25
2.24
3.06
3.11
6.07
10.0
9.5
9.0
8.5
8.0
7.5
7.0
6.5
6.0
5.5
5.0
4.5
4.0
3.5
3.0
2.5
2.0
1.5
1.0
0.5
0.0
f1 (ppm)

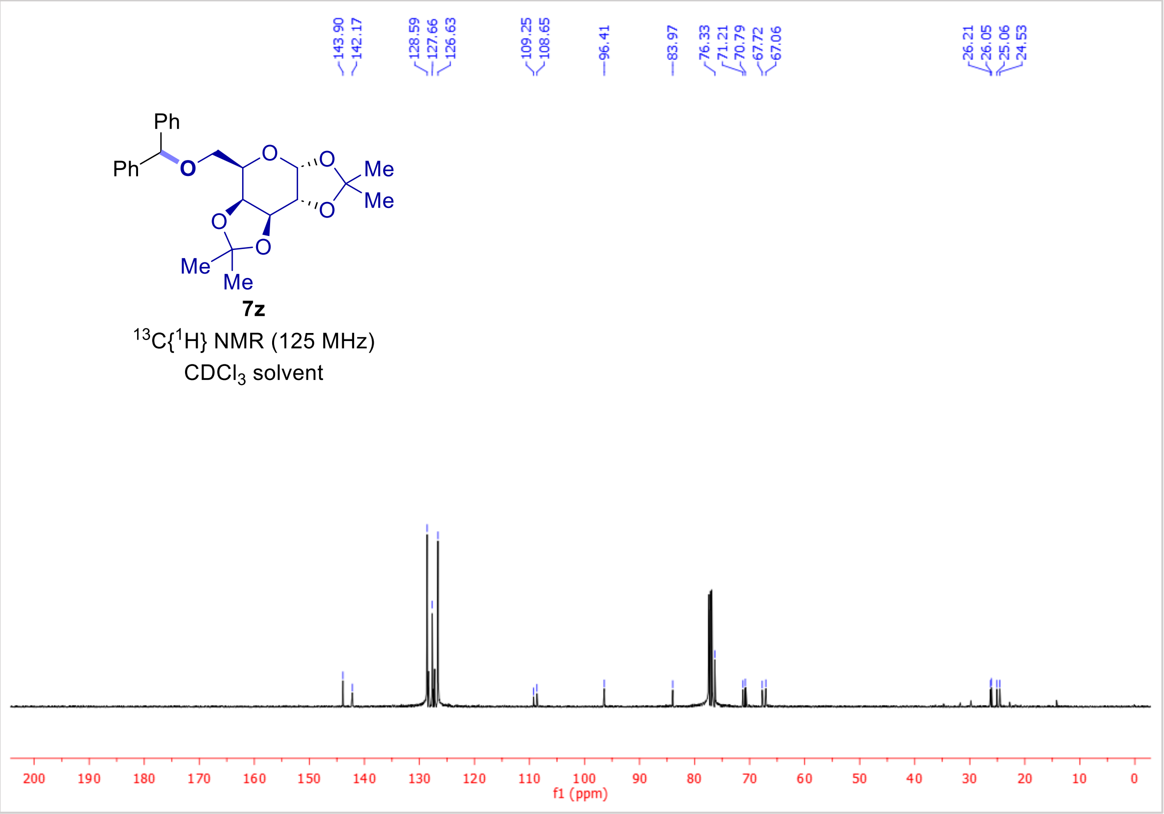
143.90
142.17
128.59
127.66
126.63
109.25
108.65
96.41
83.97
76.33
71.21
70.79
67.72
67.06
26.21
26.05
25.06
24.53
Ph
Ph
O
O
O
O
O
O
Me
Me
Me
Me
7z
13C{1H} NMR (125 MHz)
CDCl3 solvent
200
190
180
170
160
150
140
130
120
110
100
90
80
70
60
50
40
30
20
10
0
f1 (ppm)

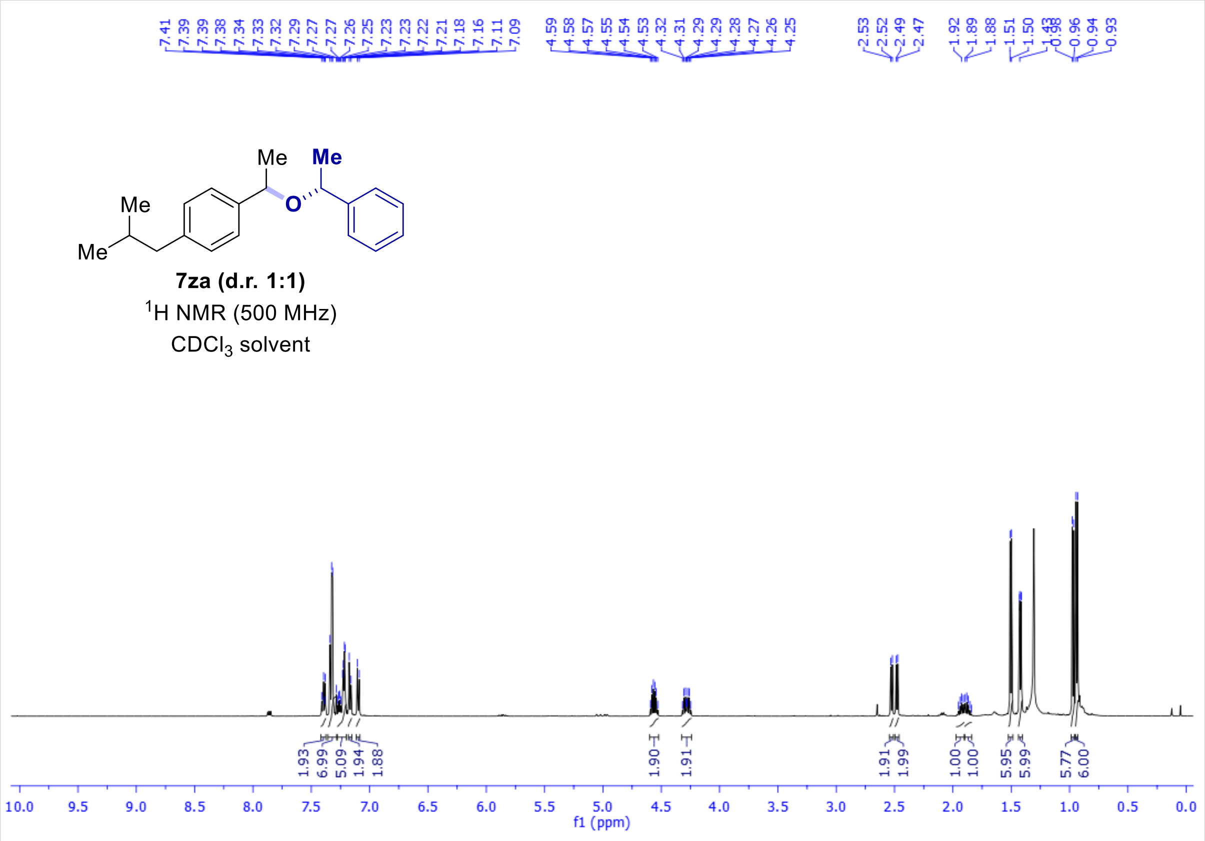
7.41 7.39 7.39 7.38 7.34 7.33 7.32 7.29 7.27 7.27 7.26 7.25 7.23 7.23 7.22 7.21 7.18 7.16 7.11 7.09
4.59 4.58 4.57 4.55 4.54 4.53 4.32 4.31 4.29 4.29 4.28 4.27 4.26 4.25
2.53 2.52 2.49 2.47
1.92 1.89 1.88 1.51 1.50 1.43 0.98 0.96 0.94 0.93
Me
Me
Me
O
Me
7za (d.r. 1:1)
1H NMR (500 MHz)
CDCl3 solvent
1.93 6.99 5.09 1.94 1.88
1.90 1.91
1.91 1.99
1.00 1.00
5.95 5.99
5.77 6.00
10.0 9.5 9.0 8.5 8.0 7.5 7.0 6.5 6.0 5.5 5.0 4.5 4.0 3.5 3.0 2.5 2.0 1.5 1.0 0.5 0.0
f1 (ppm)

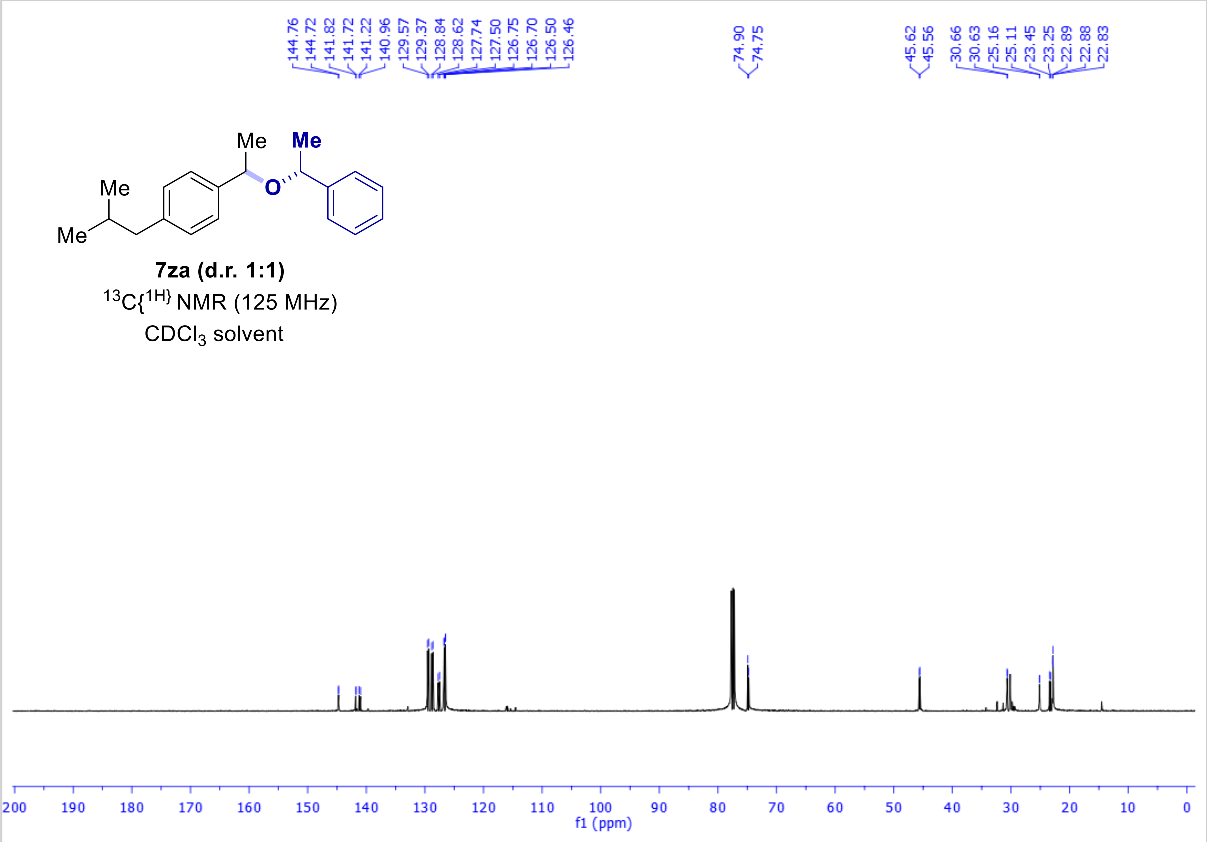
144.76 144.72 141.82 141.72 141.22 140.96 129.57 129.37 128.84 128.62 127.74 127.50 126.75 126.70 126.50 126.46
74.90 74.75
45.62 45.56 30.66 30.63 25.16 25.11 23.45 23.25 22.89 22.88 22.83
Me
Me
Me
O
Me
7za (d.r. 1:1)
13C{1H} NMR (125 MHz)
CDCl3 solvent
200 190 180 170 160 150 140 130 120 110 100 90 80 70 60 50 40 30 20 10 0
f1 (ppm)

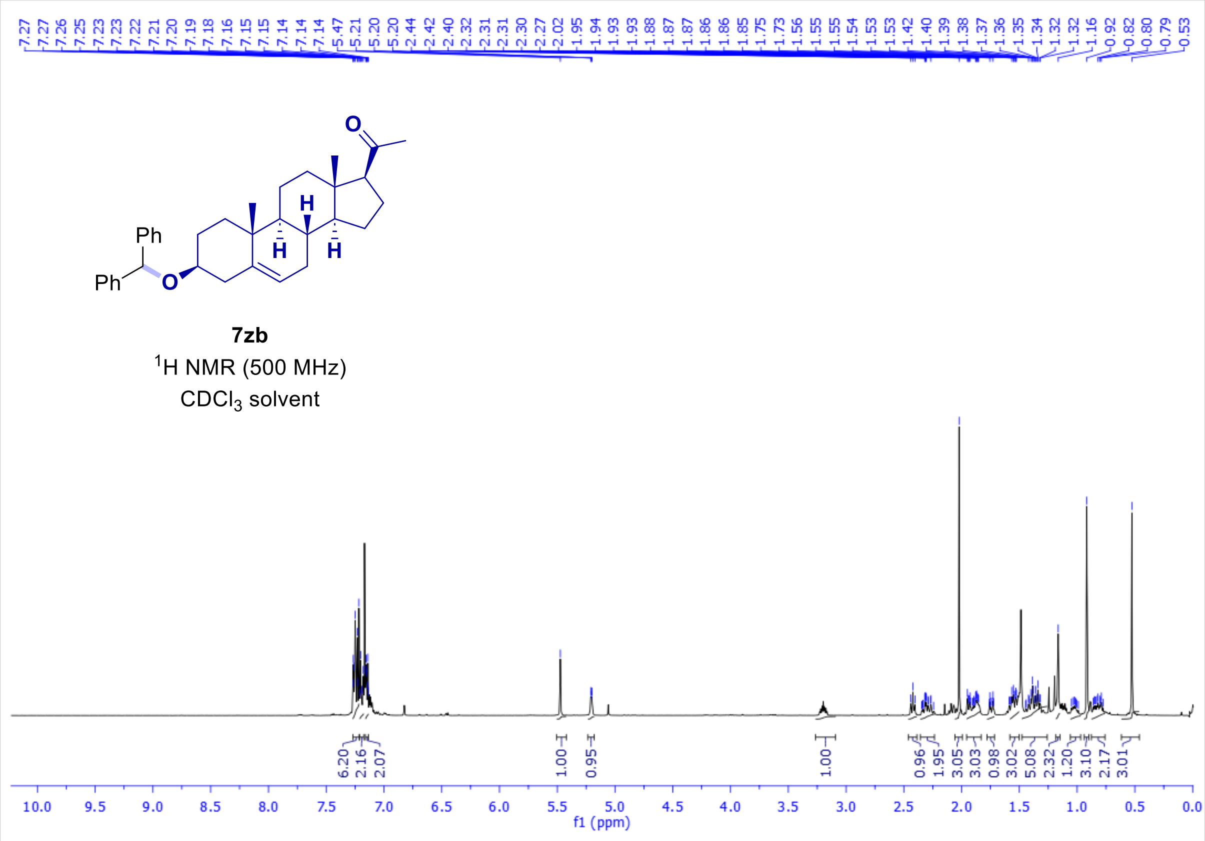
7zb
^{1}H NMR (500 MHz)
$CDCl_3$ solvent
f1 (ppm)

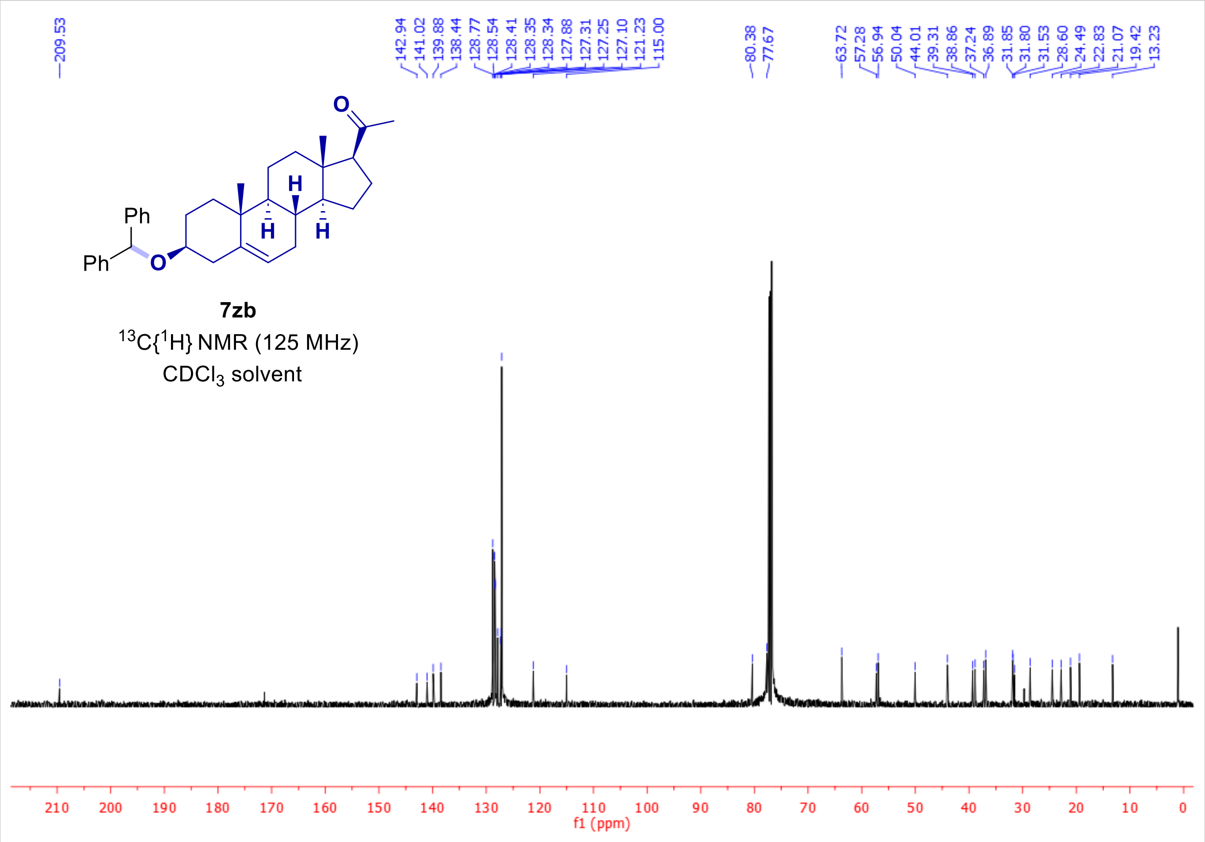
7zb
$^{13}C\{^{1}H\}$ NMR (125 MHz)
$CDCl_3$ solvent
f1 (ppm)

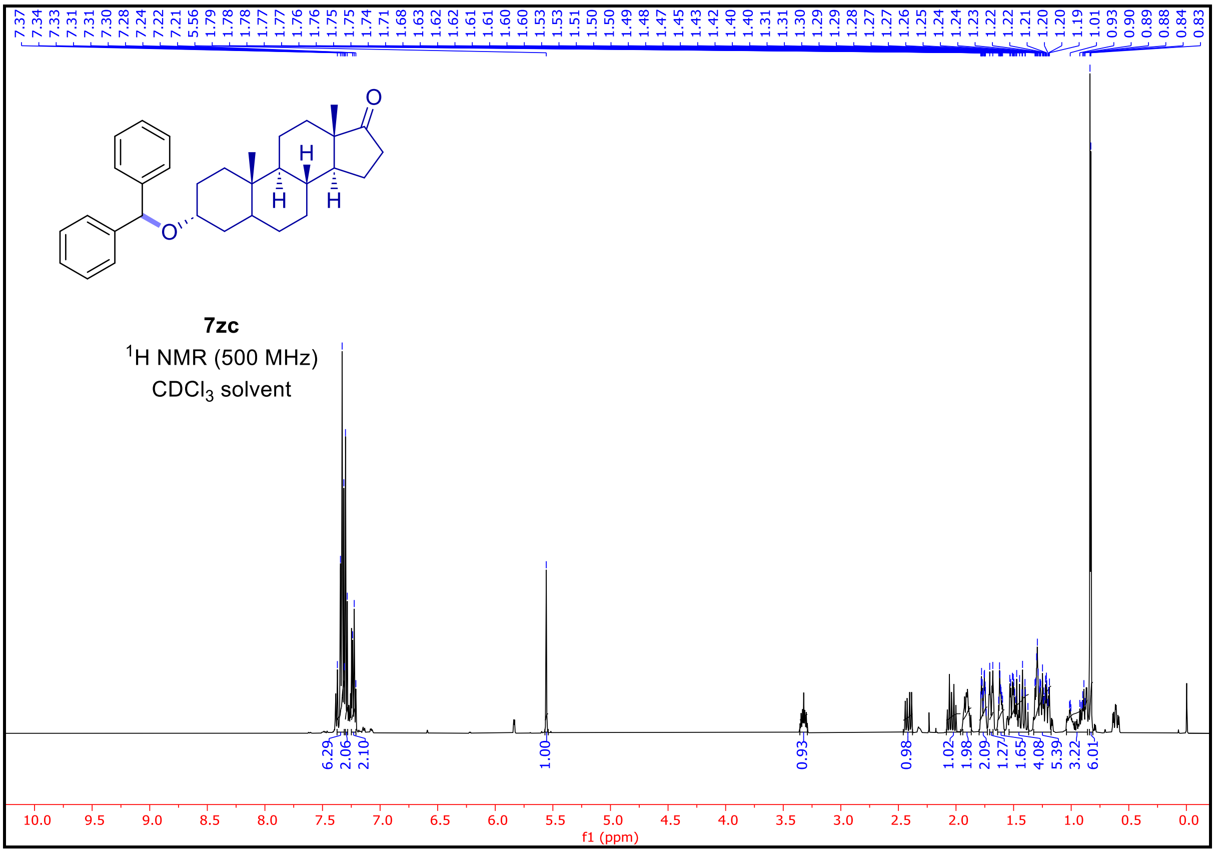
7.37 7.34 7.33 7.31 7.31 7.30 7.28 7.24 7.22 7.22 7.21 5.56 1.79 1.78 1.78 1.77 1.77 1.76 1.76 1.75 1.75 1.75 1.74 1.71 1.68 1.63 1.62 1.62 1.61 1.61 1.60 1.60 1.53 1.53 1.51 1.50 1.50 1.49 1.48 1.47 1.45 1.43 1.42 1.40 1.40 1.31 1.31 1.30 1.29 1.29 1.28 1.27 1.27 1.26 1.25 1.24 1.24 1.23 1.22 1.22 1.21 1.20 1.20 1.19 1.01 0.93 0.90 0.89 0.88 0.84 0.83
7zc
1H NMR (500 MHz)
CDCl3 solvent
6.29 2.06 2.10 1.00 0.93 0.98 1.02 1.98 2.09 1.27 1.65 4.08 5.39 3.22 6.01
10.0 9.5 9.0 8.5 8.0 7.5 7.0 6.5 6.0 5.5 5.0 4.5 4.0 3.5 3.0 2.5 2.0 1.5 1.0 0.5 0.0
f1 (ppm)

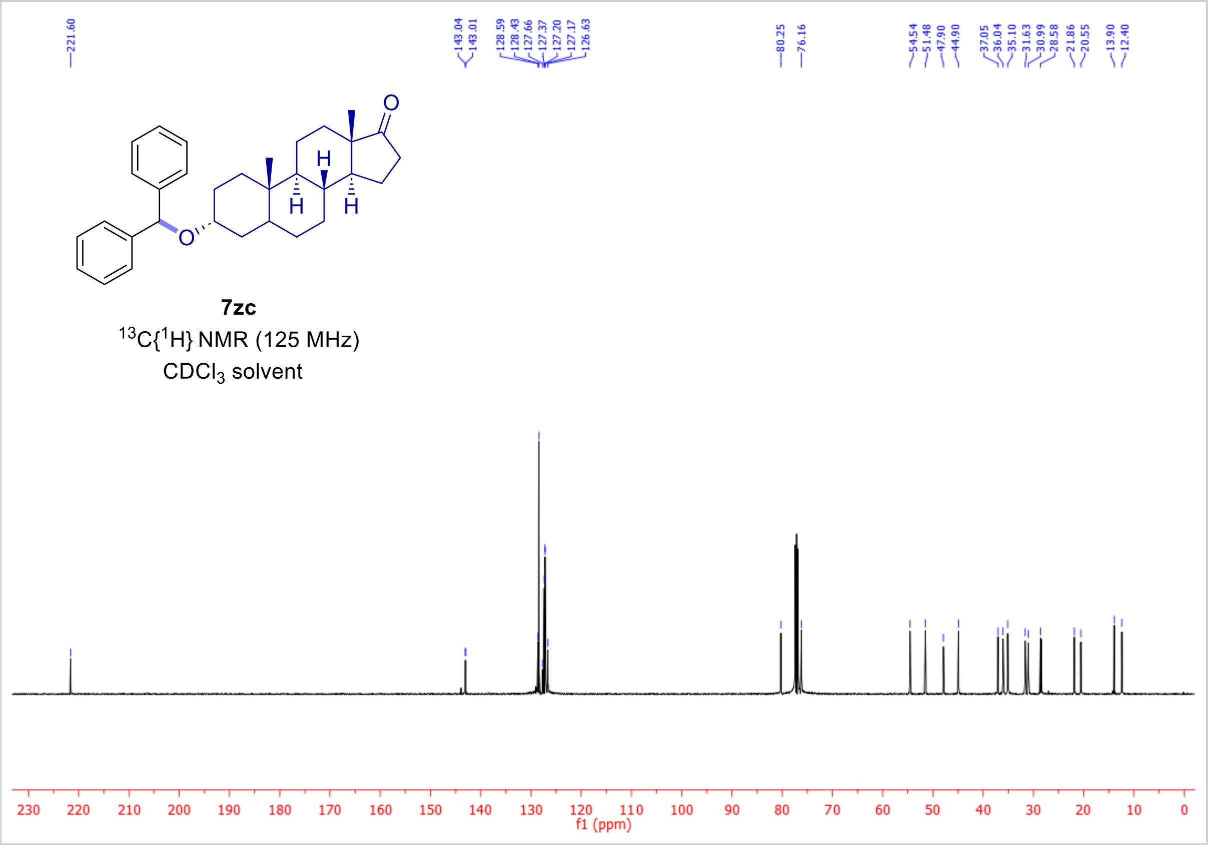
221.60
143.04 143.01
128.59 128.43 127.66 127.37 127.20 127.17 126.63
80.25 76.16
54.54 51.48 47.90 44.90
37.05 36.04 35.10 31.63 30.99 28.58 21.86 20.55 13.90 12.40
7zc
13C{1H} NMR (125 MHz)
CDCl3 solvent
230 220 210 200 190 180 170 160 150 140 130 120 110 100 90 80 70 60 50 40 30 20 10 0
f1 (ppm)

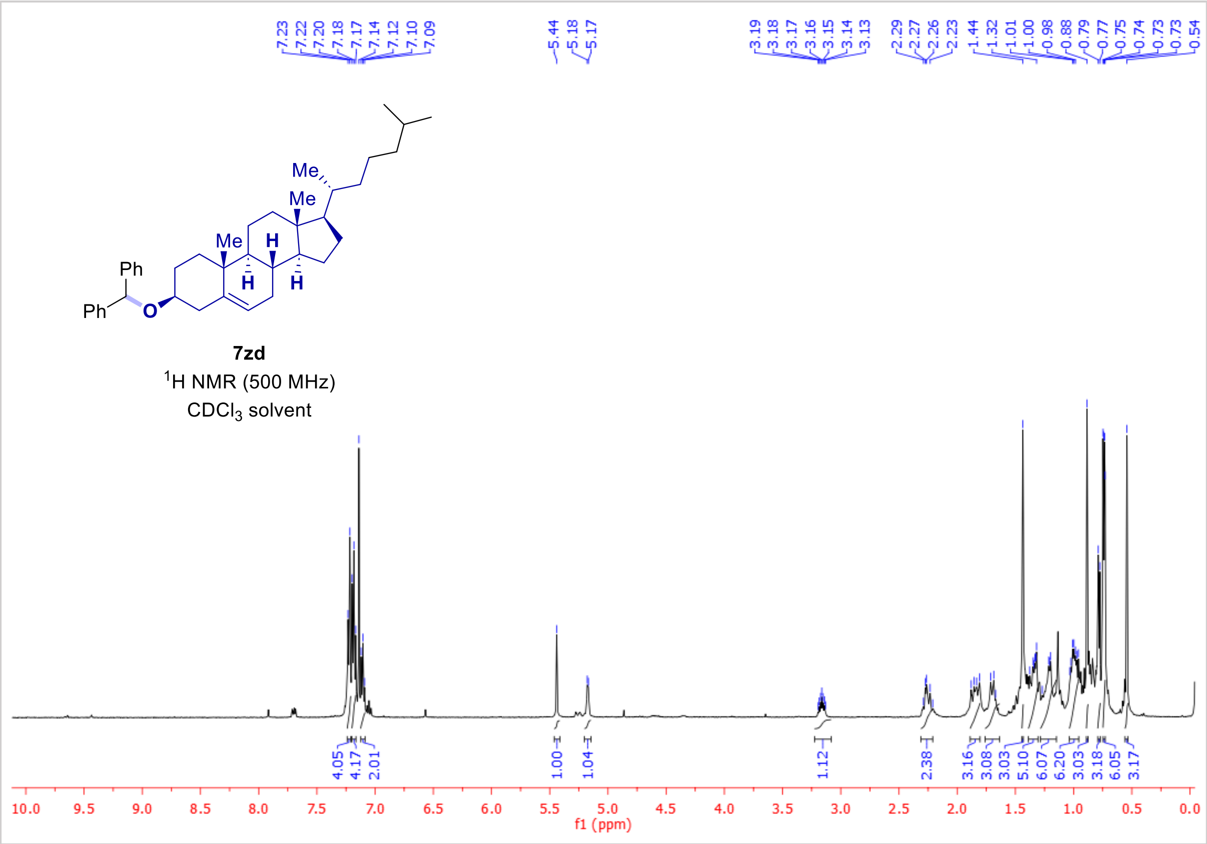
7.23 7.22 7.20 7.18 7.17 7.14 7.12 7.10 7.09
5.44 5.18 5.17
3.19 3.18 3.17 3.16 3.15 3.14 3.13
2.29 2.27 2.26 2.23
1.44 1.32 1.01 1.00 0.98 0.88 0.79 0.77 0.75 0.74 0.73 0.73 0.54
Me
Me
Me
H
H
H
Ph
Ph
O
7zd
1H NMR (500 MHz)
CDCl3 solvent
4.05 4.17 2.01 1.00 1.04 1.12 2.38 3.16 3.08 3.03 5.10 6.07 6.20 3.03 3.18 6.05 3.17
10.0 9.5 9.0 8.5 8.0 7.5 7.0 6.5 6.0 5.5 5.0 4.5 4.0 3.5 3.0 2.5 2.0 1.5 1.0 0.5 0.0
f1 (ppm)

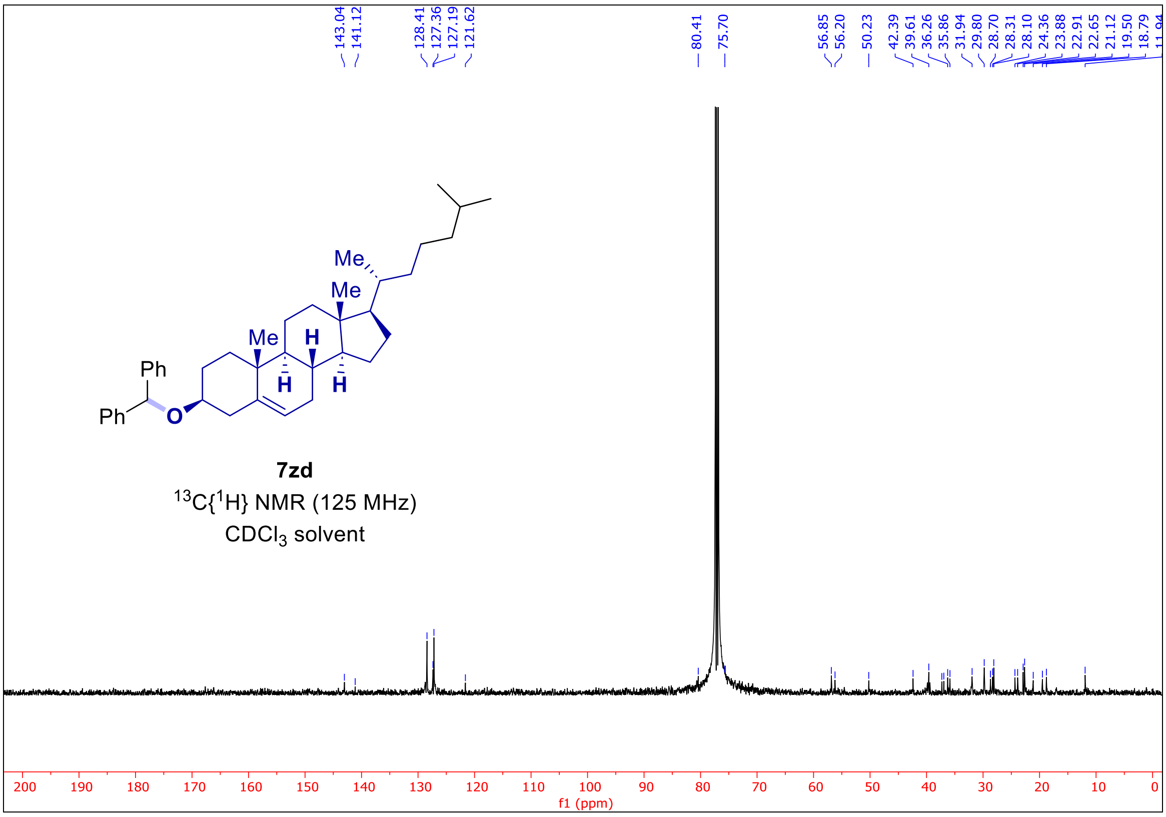
143.04 141.12
128.41 127.36 127.19 121.62
80.41 75.70
56.85 56.20 50.23
42.39 39.61 36.26 35.86 31.94 29.80 28.70 28.31 28.10 24.36 23.88 22.91 22.65 21.12 19.50 18.79 11.94
Me
Me
Me
H
H
H
Ph
Ph
O
7zd
13C{1H} NMR (125 MHz)
CDCl3 solvent
200 190 180 170 160 150 140 130 120 110 100 90 80 70 60 50 40 30 20 10 0
f1 (ppm)

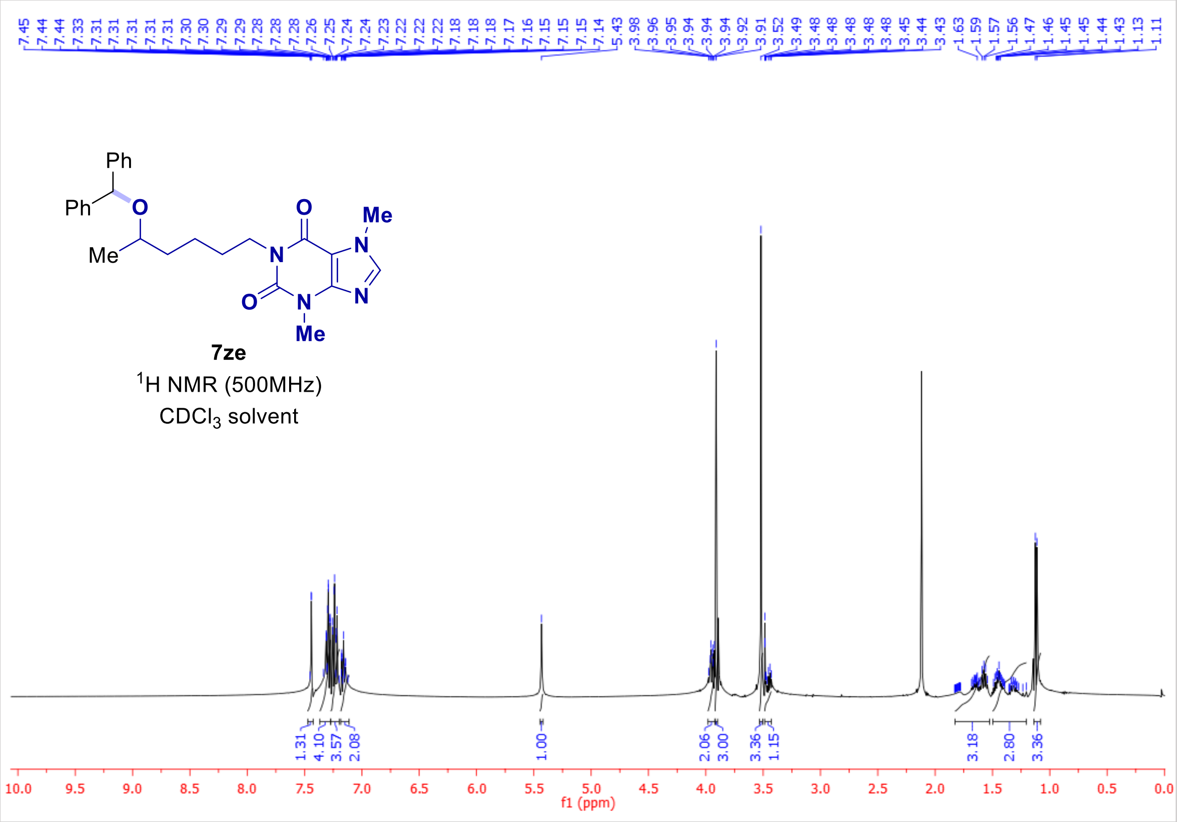
7.45 7.44 7.44 7.33 7.31 7.31 7.31 7.31 7.31 7.30 7.30 7.29 7.29 7.28 7.28 7.28 7.26 7.25 7.24 7.24 7.23 7.22 7.22 7.22 7.18 7.18 7.18 7.17 7.16 7.15 7.15 7.15 7.14 5.43 3.98 3.96 3.95 3.94 3.94 3.94 3.92 3.91 3.52 3.49 3.48 3.48 3.48 3.48 3.45 3.44 3.43 1.63 1.59 1.57 1.56 1.47 1.46 1.45 1.45 1.44 1.43 1.13 1.11
Ph
Ph
O
Me
O
Me
N
N
N
N
O
Me
7ze
1H NMR (500MHz)
CDCl3 solvent
1.31 4.10 3.57 2.08 1.00 2.06 3.00 3.36 1.15 3.18 2.80 3.36
10.0 9.5 9.0 8.5 8.0 7.5 7.0 6.5 6.0 5.5 5.0 4.5 4.0 3.5 3.0 2.5 2.0 1.5 1.0 0.5 0.0
f1 (ppm)

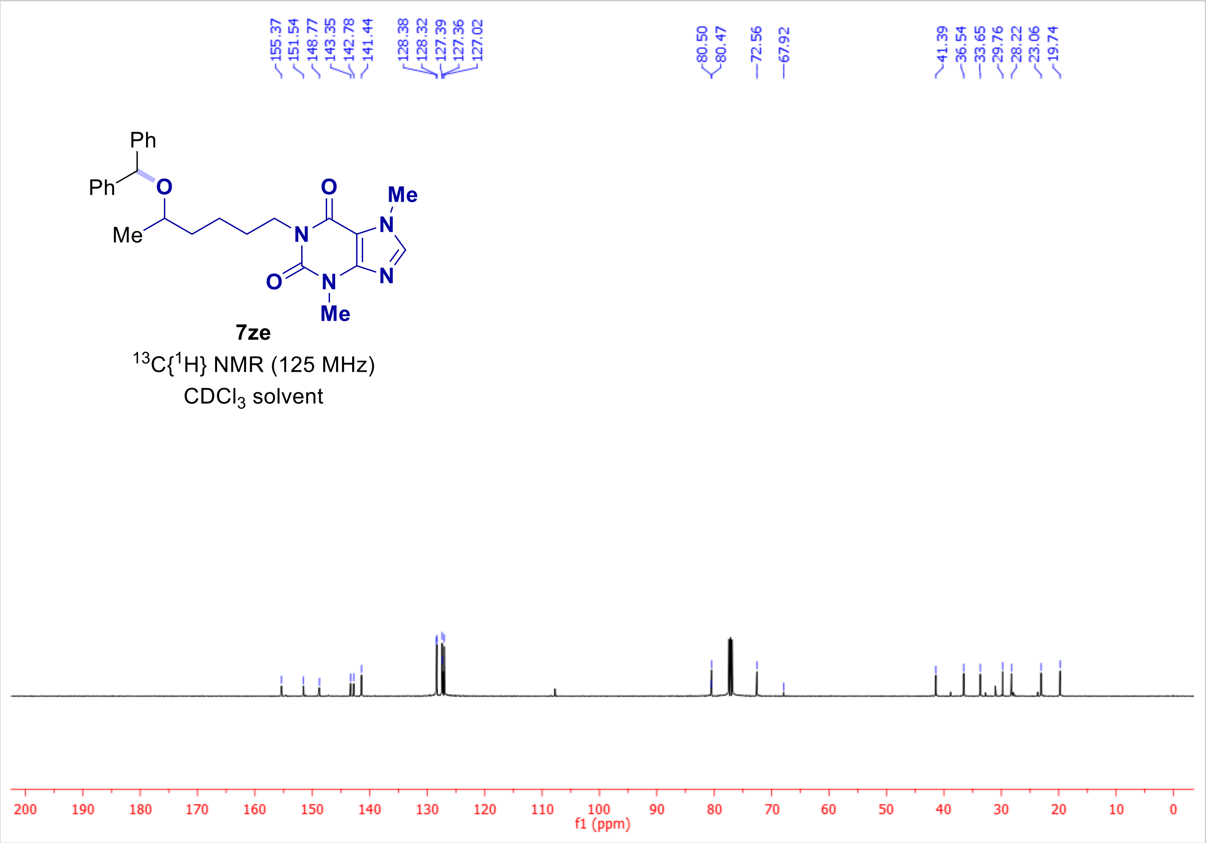
155.37 151.54 148.77 143.35 142.78 141.44 128.38 128.32 127.39 127.36 127.02 80.50 80.47 72.56 67.92 41.39 36.54 33.65 29.76 28.22 23.06 19.74
Ph
Ph
O
Me
O
Me
N
N
N
N
O
Me
7ze
13C{1H} NMR (125 MHz)
CDCl3 solvent
200 190 180 170 160 150 140 130 120 110 100 90 80 70 60 50 40 30 20 10 0
f1 (ppm)

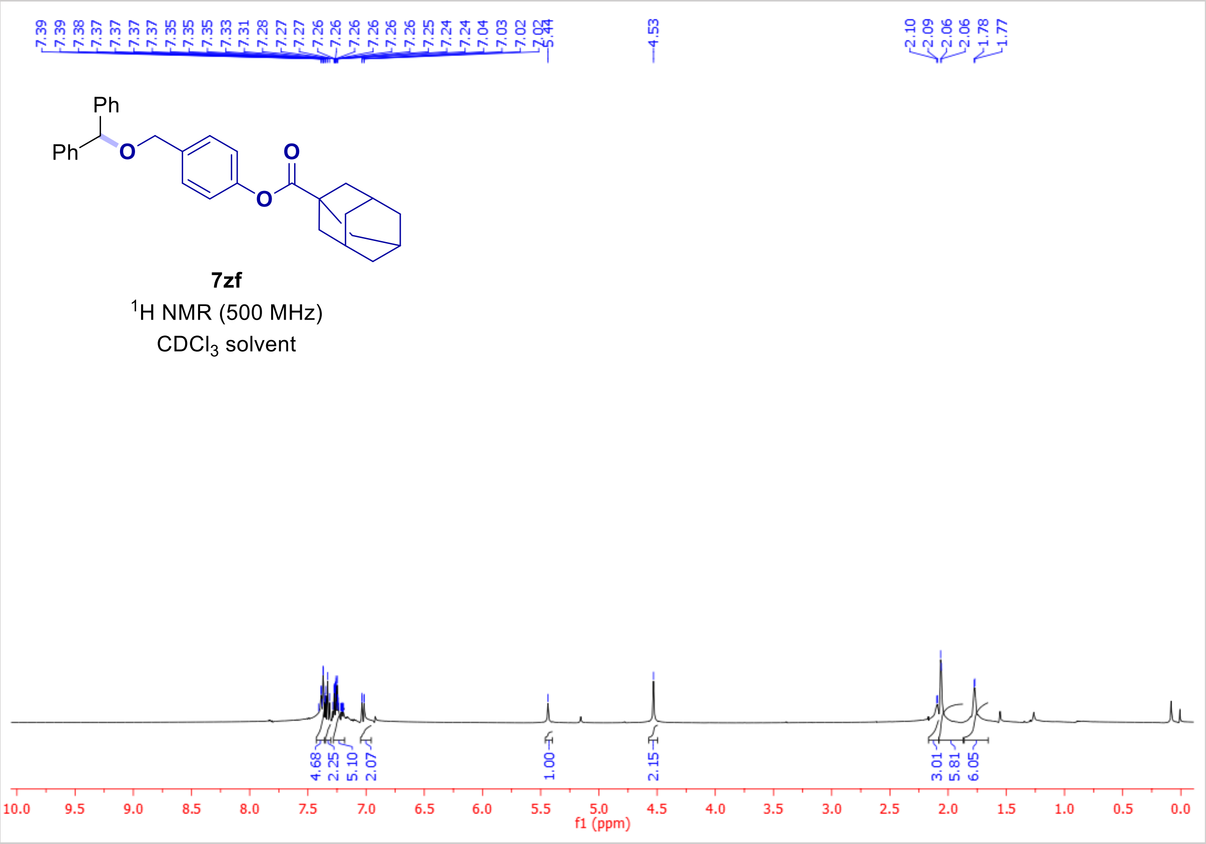
7.39
7.39
7.38
7.37
7.37
7.37
7.37
7.35
7.35
7.35
7.33
7.31
7.28
7.27
7.27
7.26
7.26
7.26
7.26
7.26
7.26
7.25
7.24
7.24
7.04
7.03
7.02
7.02
5.44
4.53
2.10
2.09
2.06
2.06
1.78
1.77
Ph
Ph
O
O
O
7zf
1H NMR (500 MHz)
CDCl3 solvent
4.68
2.25
5.10
2.07
1.00
2.15
3.01
5.81
6.05
10.0
9.5
9.0
8.5
8.0
7.5
7.0
6.5
6.0
5.5
5.0
4.5
4.0
3.5
3.0
2.5
2.0
1.5
1.0
0.5
0.0
f1 (ppm)

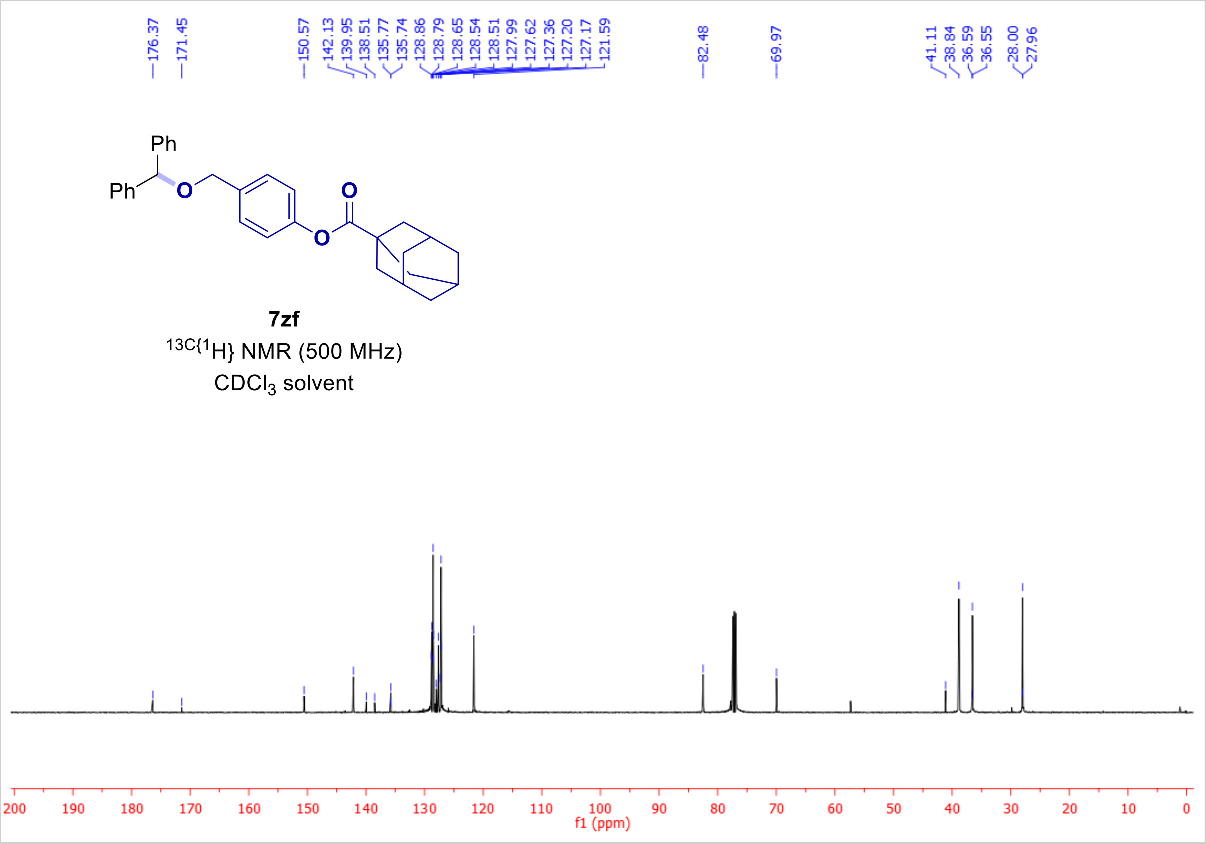
176.37
171.45
150.57
142.13
139.95
138.51
135.77
135.74
128.86
128.79
128.65
128.54
128.51
127.99
127.62
127.36
127.20
127.17
121.59
82.48
69.97
41.11
38.84
36.59
36.55
28.00
27.96
Ph
Ph
O
O
O
7zf
13C{1H} NMR (500 MHz)
CDCl3 solvent
200
190
180
170
160
150
140
130
120
110
100
90
80
70
60
50
40
30
20
10
0
f1 (ppm)

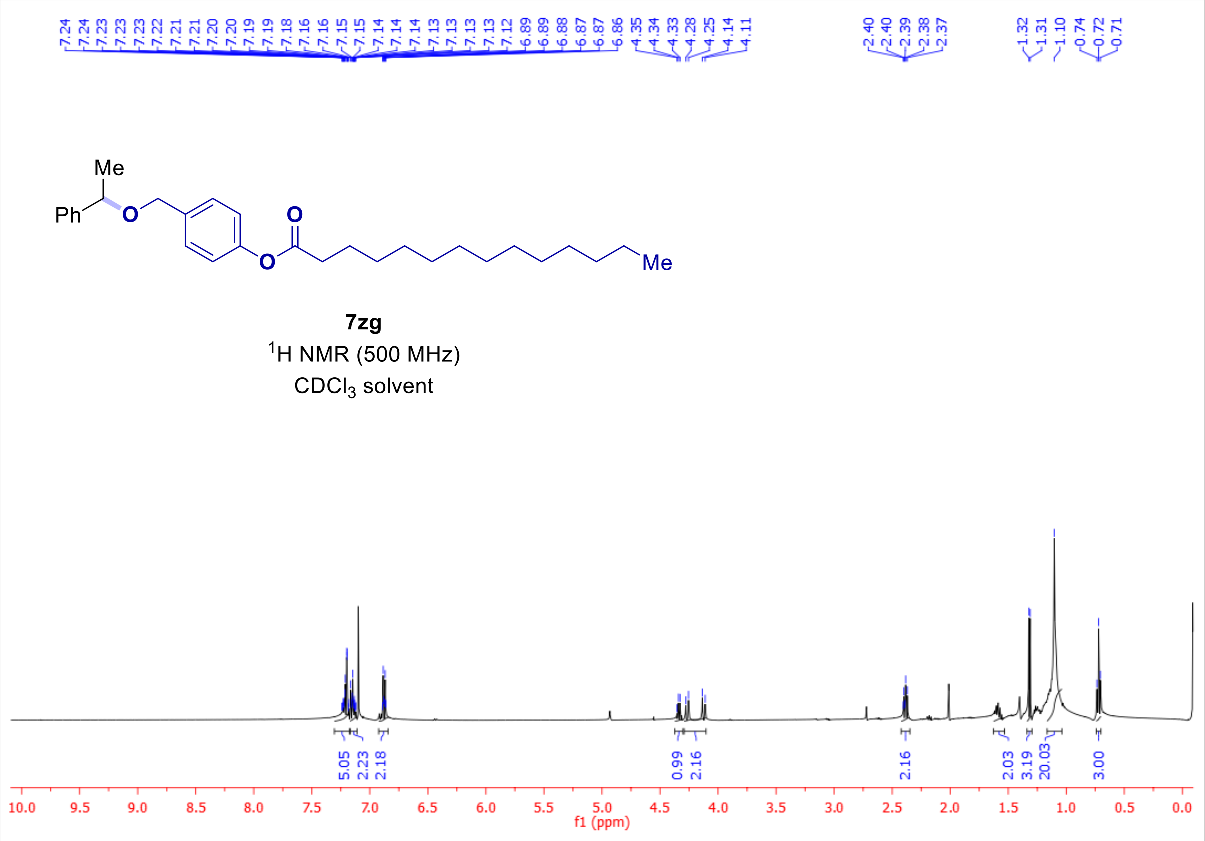
7.24
7.24
7.23
7.23
7.23
7.22
7.21
7.21
7.20
7.20
7.19
7.19
7.18
7.16
7.16
7.15
7.15
7.14
7.14
7.14
7.13
7.13
7.13
7.13
7.12
6.89
6.89
6.88
6.87
6.87
6.86
4.35
4.34
4.33
4.28
4.25
4.14
4.11
2.40
2.40
2.39
2.38
2.37
1.32
1.31
1.10
0.74
0.72
0.71
Me
Ph
O
O
O
Me
7zg
1H NMR (500 MHz)
CDCl3 solvent
5.05
2.23
2.18
0.99
2.16
2.16
2.03
3.19
20.03
3.00
f1 (ppm)

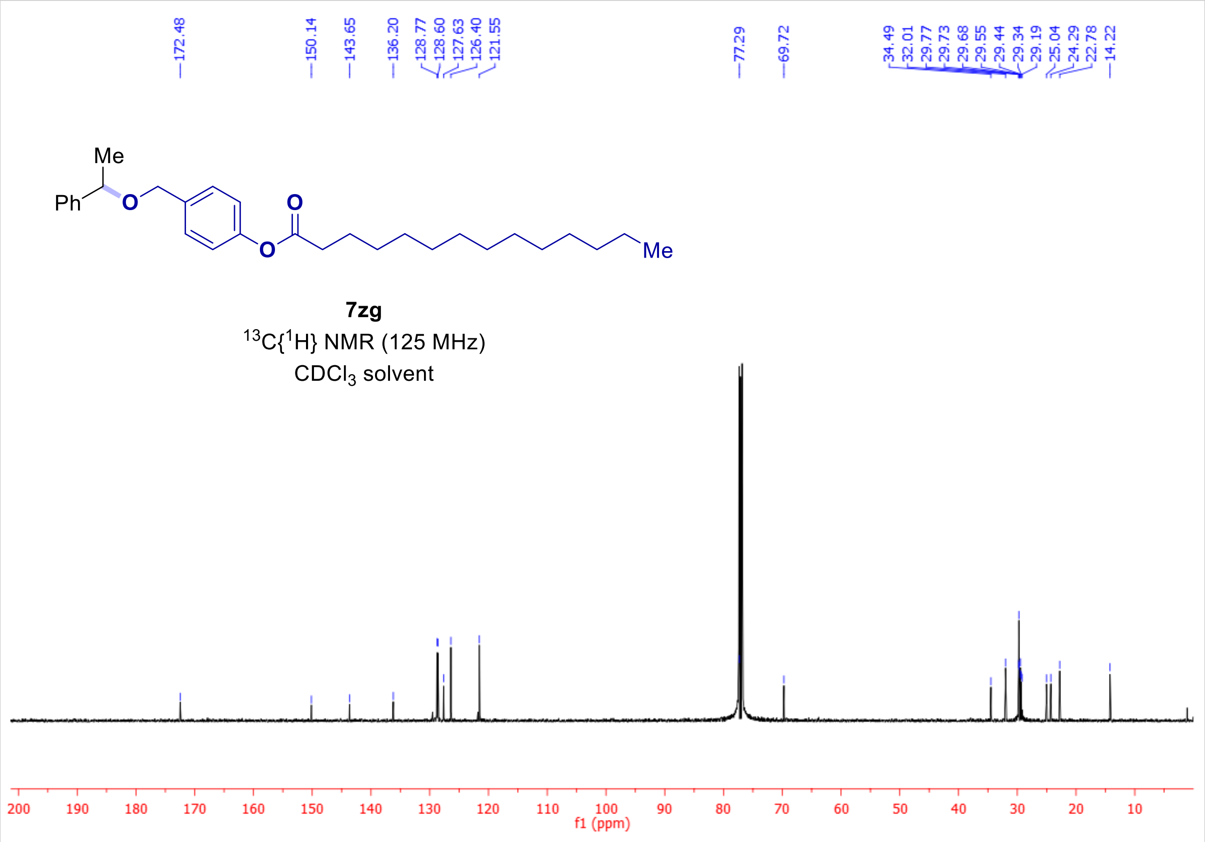
172.48
150.14
143.65
136.20
128.77
128.60
127.63
126.40
121.55
77.29
69.72
34.49
32.01
29.77
29.73
29.68
29.55
29.44
29.34
29.19
25.04
24.29
22.78
14.22
Me
Ph
O
O
O
Me
7zg
13C{1H} NMR (125 MHz)
CDCl3 solvent
f1 (ppm)

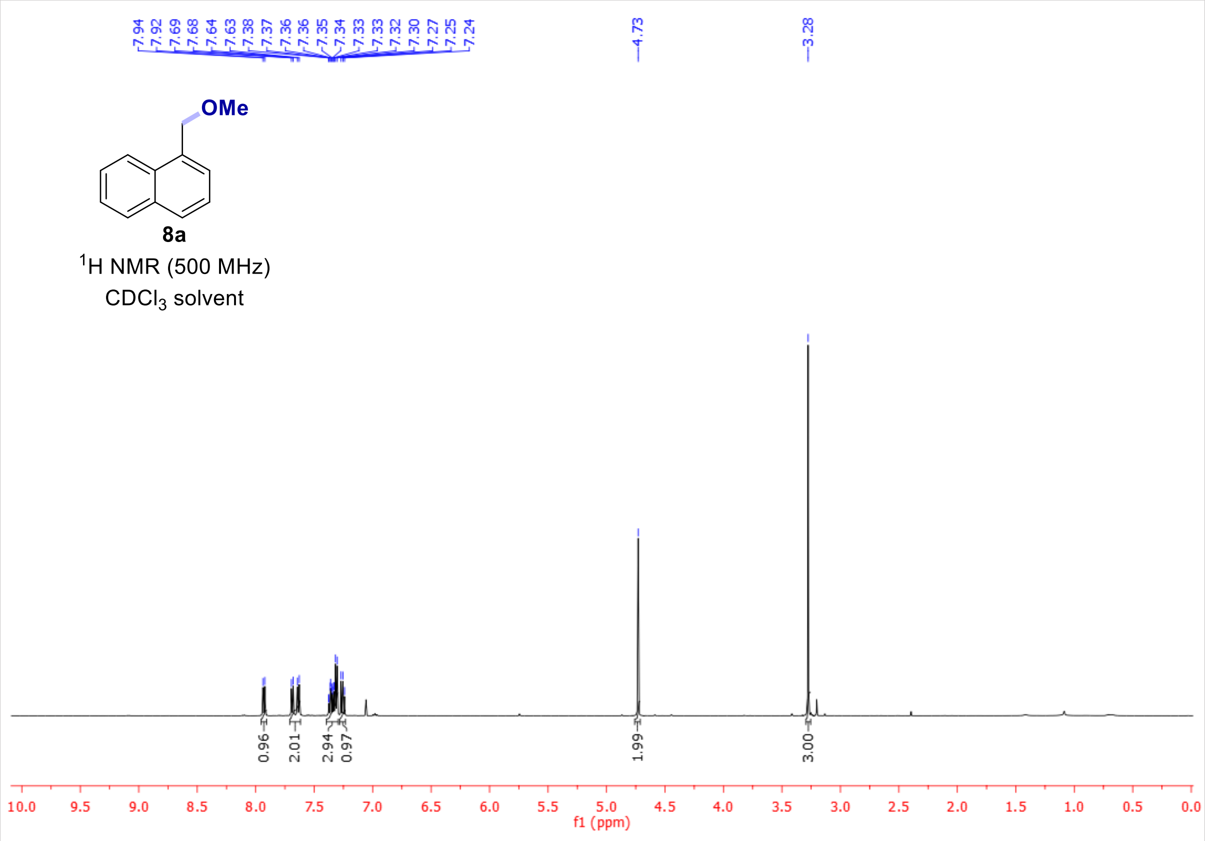
OMe
8a
^{1}H NMR (500 MHz)
$CDCl_3$ solvent
7.94 7.92 7.69 7.68 7.64 7.63 7.38 7.37 7.36 7.36 7.35 7.34 7.33 7.33 7.32 7.30 7.27 7.25 7.24
4.73
3.28
0.96 2.01 2.94 0.97 1.99 3.00
10.0 9.5 9.0 8.5 8.0 7.5 7.0 6.5 6.0 5.5 5.0 4.5 4.0 3.5 3.0 2.5 2.0 1.5 1.0 0.5 0.0
f1 (ppm)

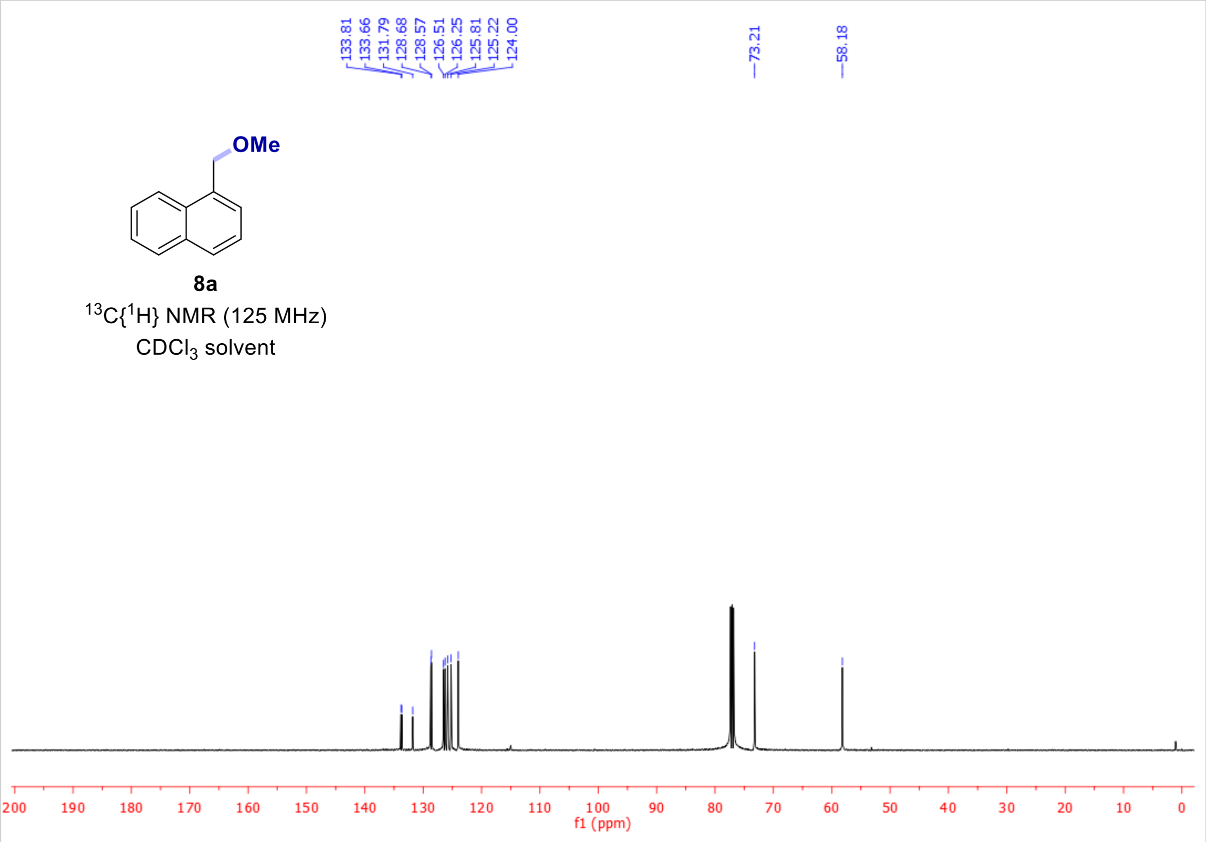
OMe
8a
^{13}C{^{1}H} NMR (125 MHz)
$CDCl_3$ solvent
133.81 133.66 131.79 128.68 128.57 126.51 126.25 125.81 125.22 124.00
73.21
58.18
200 190 180 170 160 150 140 130 120 110 100 90 80 70 60 50 40 30 20 10 0
f1 (ppm)

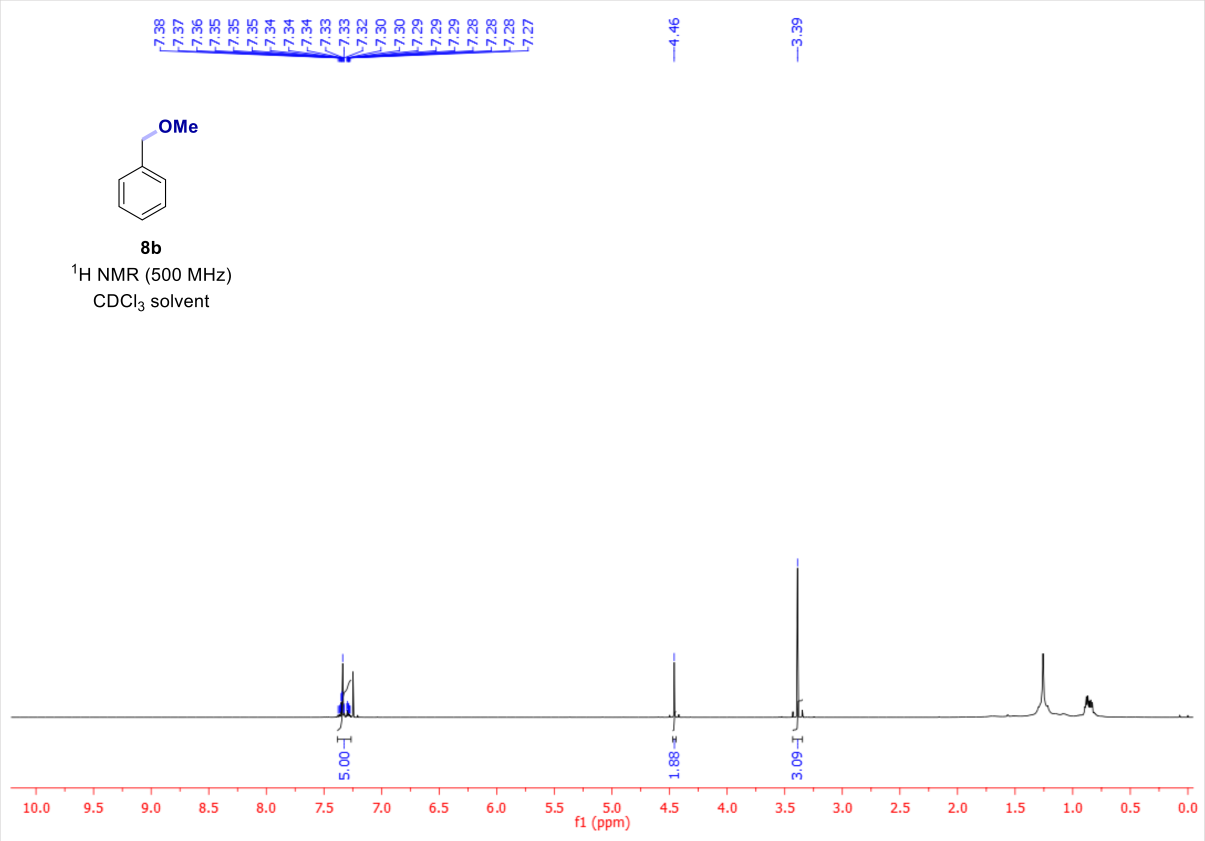
7.38
7.37
7.36
7.35
7.35
7.35
7.34
7.34
7.34
7.33
7.33
7.32
7.30
7.30
7.29
7.29
7.29
7.29
7.28
7.28
7.28
7.27
4.46
3.39
OMe
8b
^{1}H NMR (500 MHz)
$CDCl_3$ solvent
5.00
1.88
3.09
10.0
9.5
9.0
8.5
8.0
7.5
7.0
6.5
6.0
5.5
5.0
4.5
4.0
3.5
3.0
2.5
2.0
1.5
1.0
0.5
0.0
f1 (ppm)

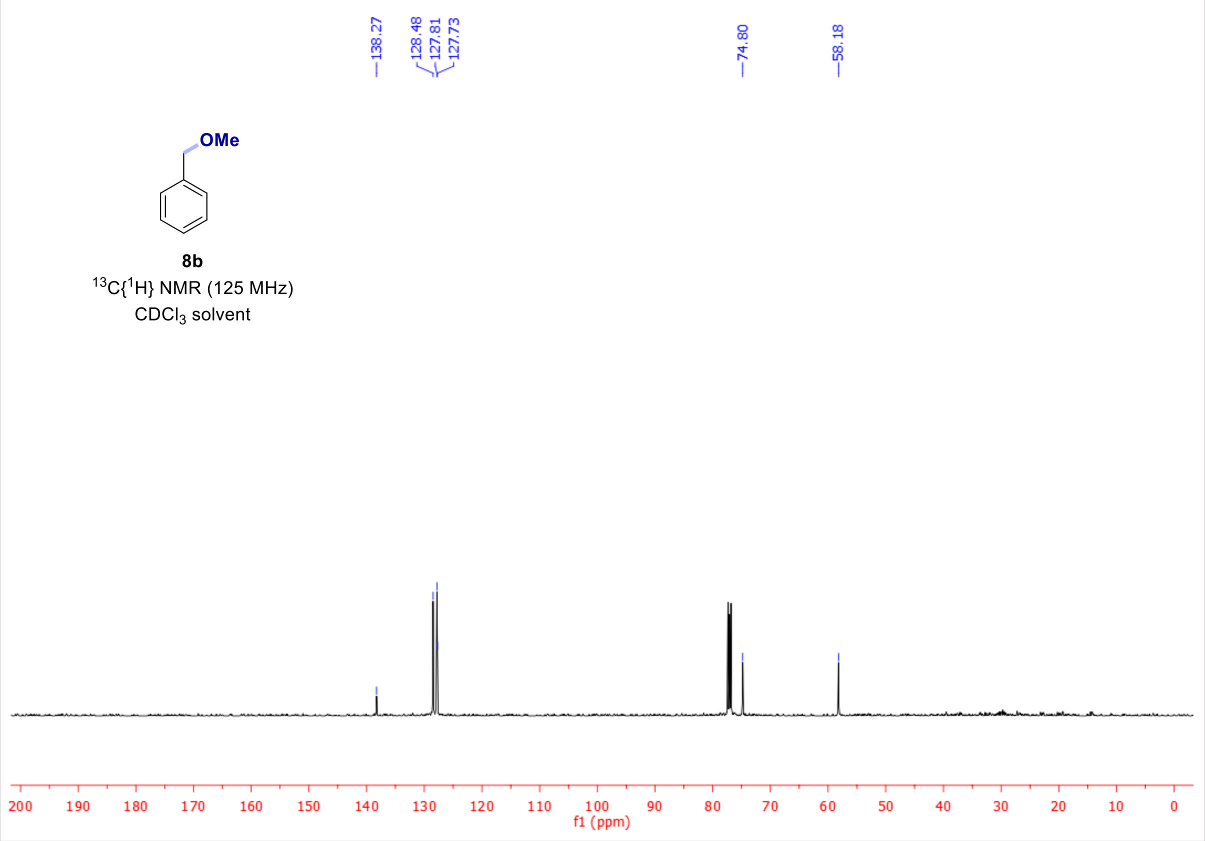
138.27
128.48
127.81
127.73
74.80
58.18
OMe
8b
$^{13}C\{^1H\}$ NMR (125 MHz)
$CDCl_3$ solvent
200
190
180
170
160
150
140
130
120
110
100
90
80
70
60
50
40
30
20
10
0
f1 (ppm)

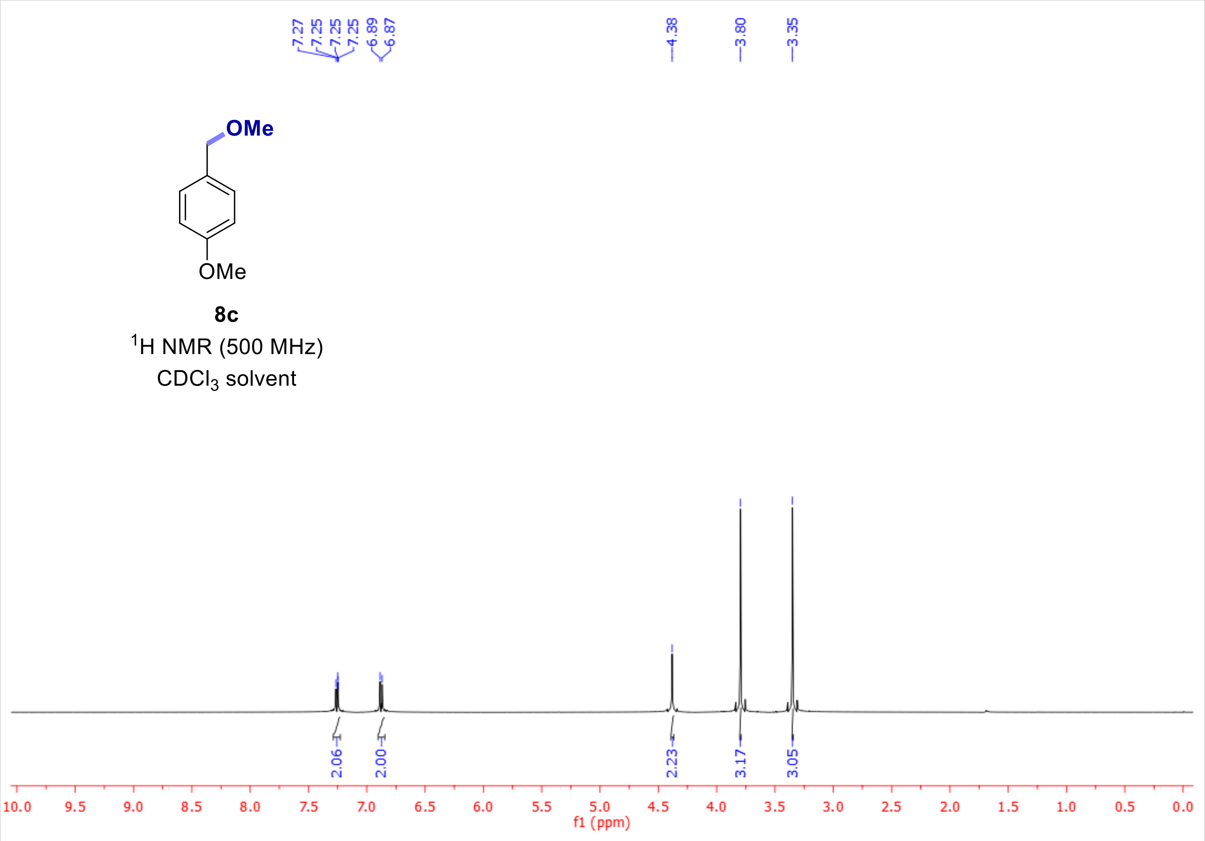
7.27
7.25
7.25
7.25
6.89
6.87
4.38
3.80
3.35
OMe
OMe
8c
1H NMR (500 MHz)
CDCl3 solvent
2.06
2.00
2.23
3.17
3.05
10.0 9.5 9.0 8.5 8.0 7.5 7.0 6.5 6.0 5.5 5.0 4.5 4.0 3.5 3.0 2.5 2.0 1.5 1.0 0.5 0.0
f1 (ppm)

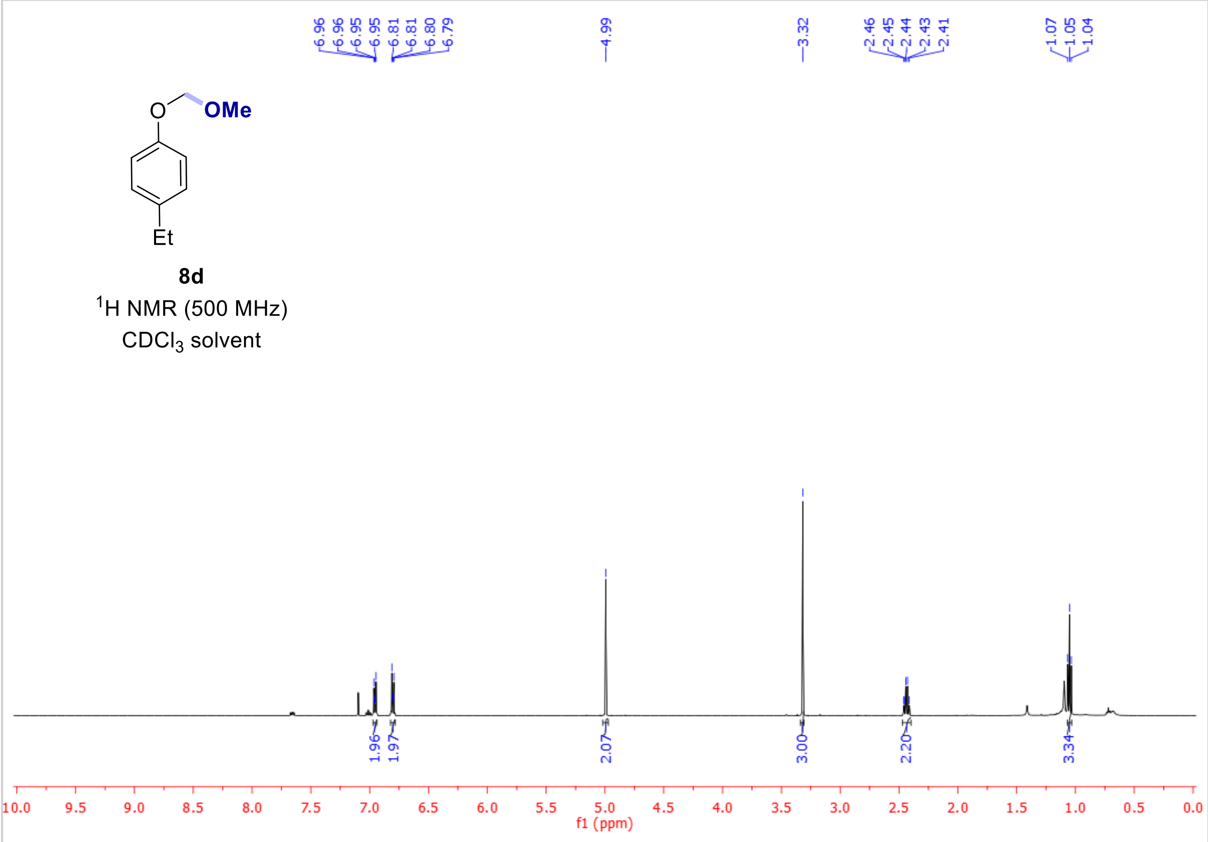
6.96
6.96
6.95
6.95
6.81
6.81
6.80
6.79
4.99
3.32
2.46
2.45
2.44
2.43
2.41
1.07
1.05
1.04
O
OMe
Et
8d
1H NMR (500 MHz)
CDCl3 solvent
1.96
1.97
2.07
3.00
2.20
3.34
10.0 9.5 9.0 8.5 8.0 7.5 7.0 6.5 6.0 5.5 5.0 4.5 4.0 3.5 3.0 2.5 2.0 1.5 1.0 0.5 0.0
f1 (ppm)

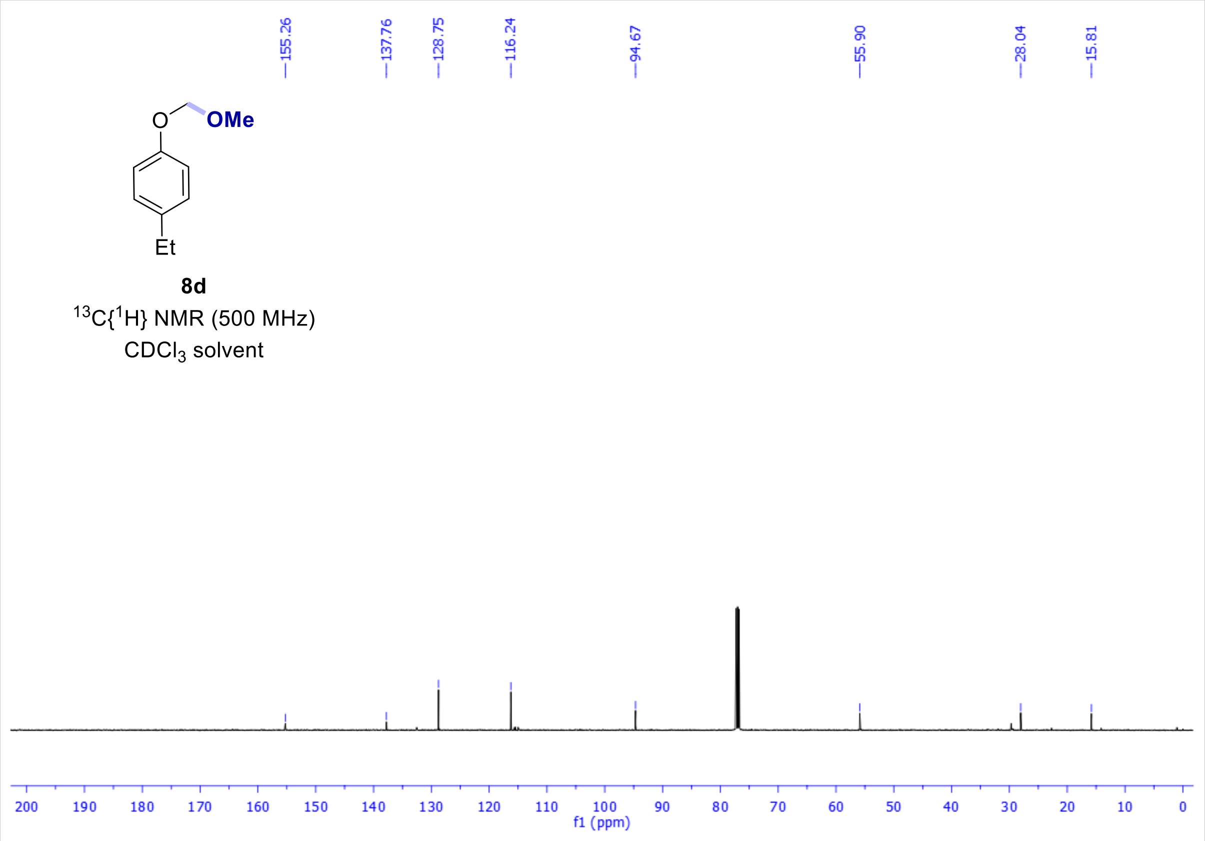

155.26
137.76
128.75
116.24
94.67
55.90
28.04
15.81
OMe
O
Et
8d
$^{13}C\{^1H\}$ NMR (500 MHz)
$CDCl_3$ solvent
200 190 180 170 160 150 140 130 120 110 100 90 80 70 60 50 40 30 20 10 0
f1 (ppm)

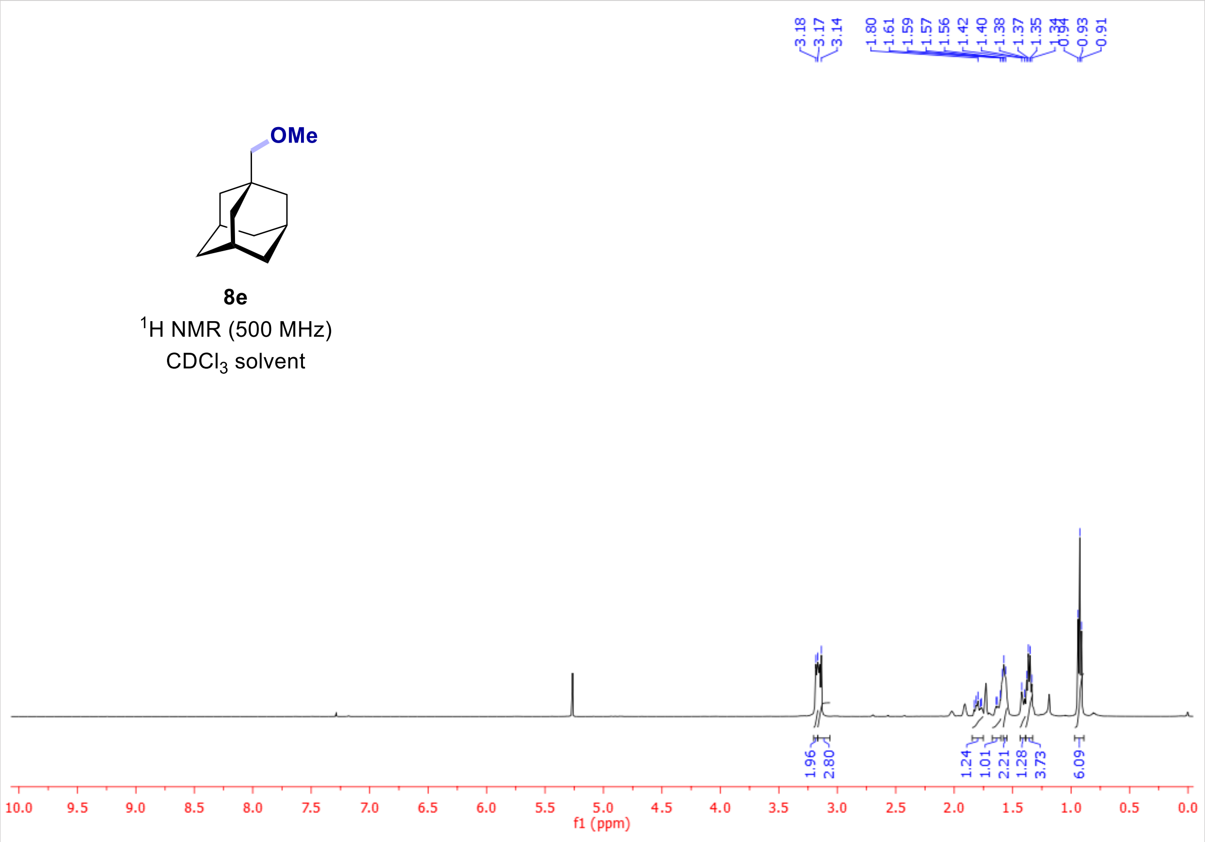

3.18
3.17
3.14
1.80
1.61
1.59
1.57
1.56
1.42
1.40
1.38
1.37
1.35
1.34
0.94
0.93
0.91
OMe
8e
^1H NMR (500 MHz)
$CDCl_3$ solvent
1.96
2.80
1.24
1.01
2.21
1.28
3.73
6.09
10.0 9.5 9.0 8.5 8.0 7.5 7.0 6.5 6.0 5.5 5.0 4.5 4.0 3.5 3.0 2.5 2.0 1.5 1.0 0.5 0.0
f1 (ppm)

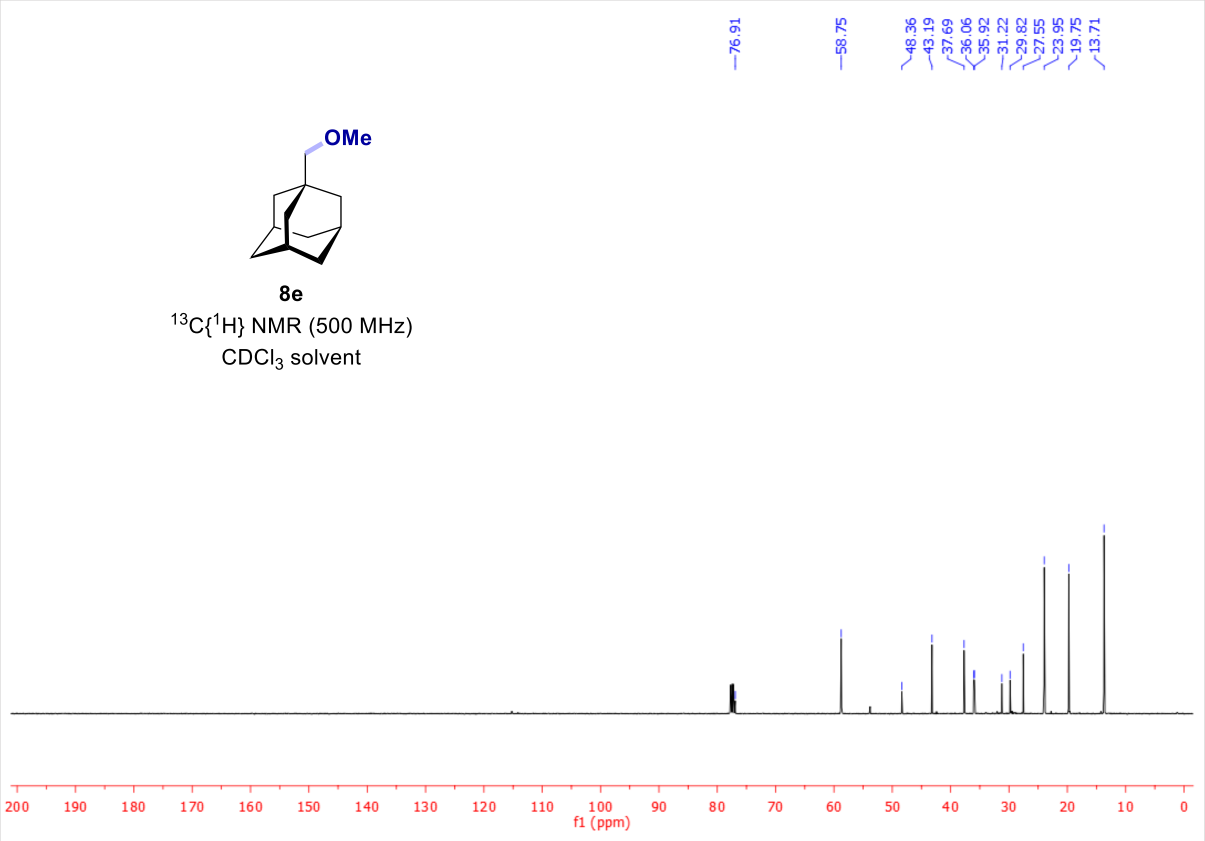
OMe
8e
13C{1H} NMR (500 MHz)
CDCl3 solvent
76.91
58.75
48.36
43.19
37.69
36.06
35.92
31.22
29.82
27.55
23.95
19.75
13.71
200 190 180 170 160 150 140 130 120 110 100 90 80 70 60 50 40 30 20 10 0
f1 (ppm)

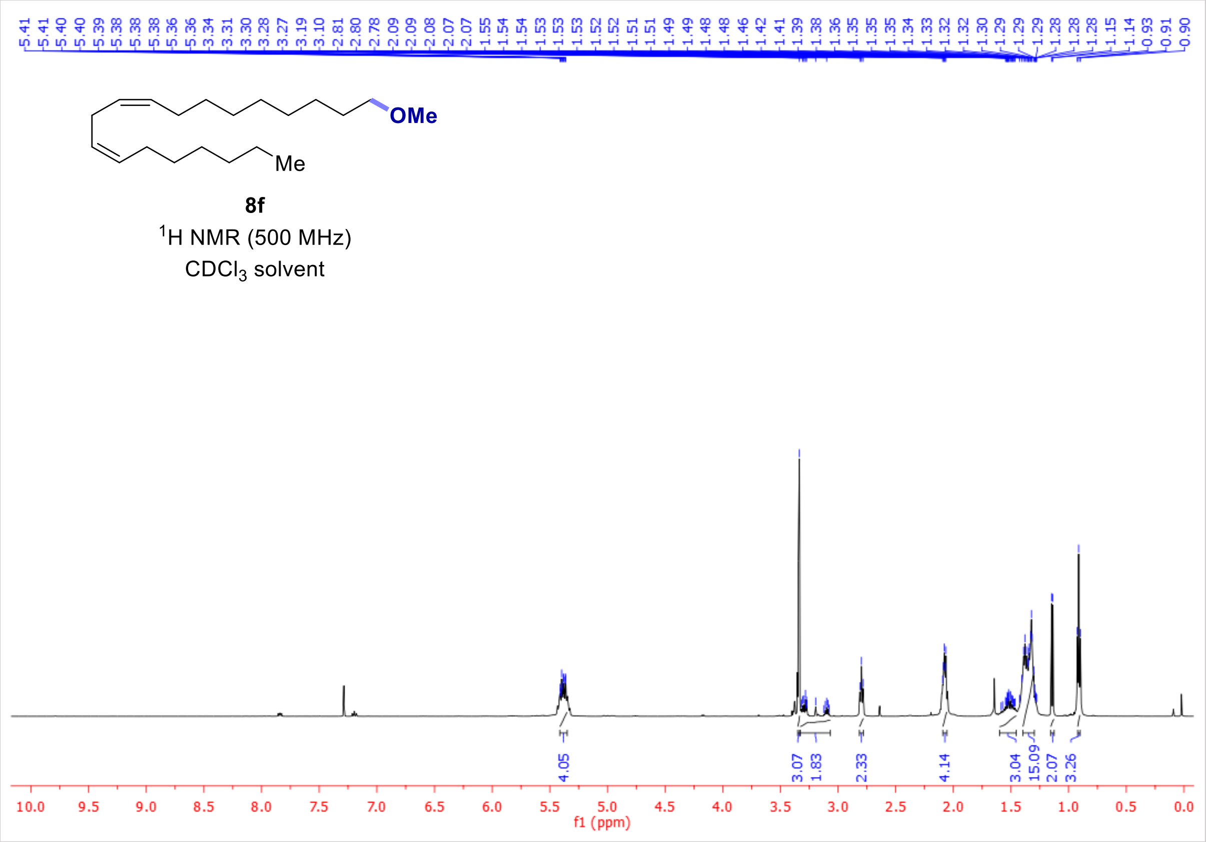
OMe
Me
8f
1H NMR (500 MHz)
CDCl3 solvent
f1 (ppm)

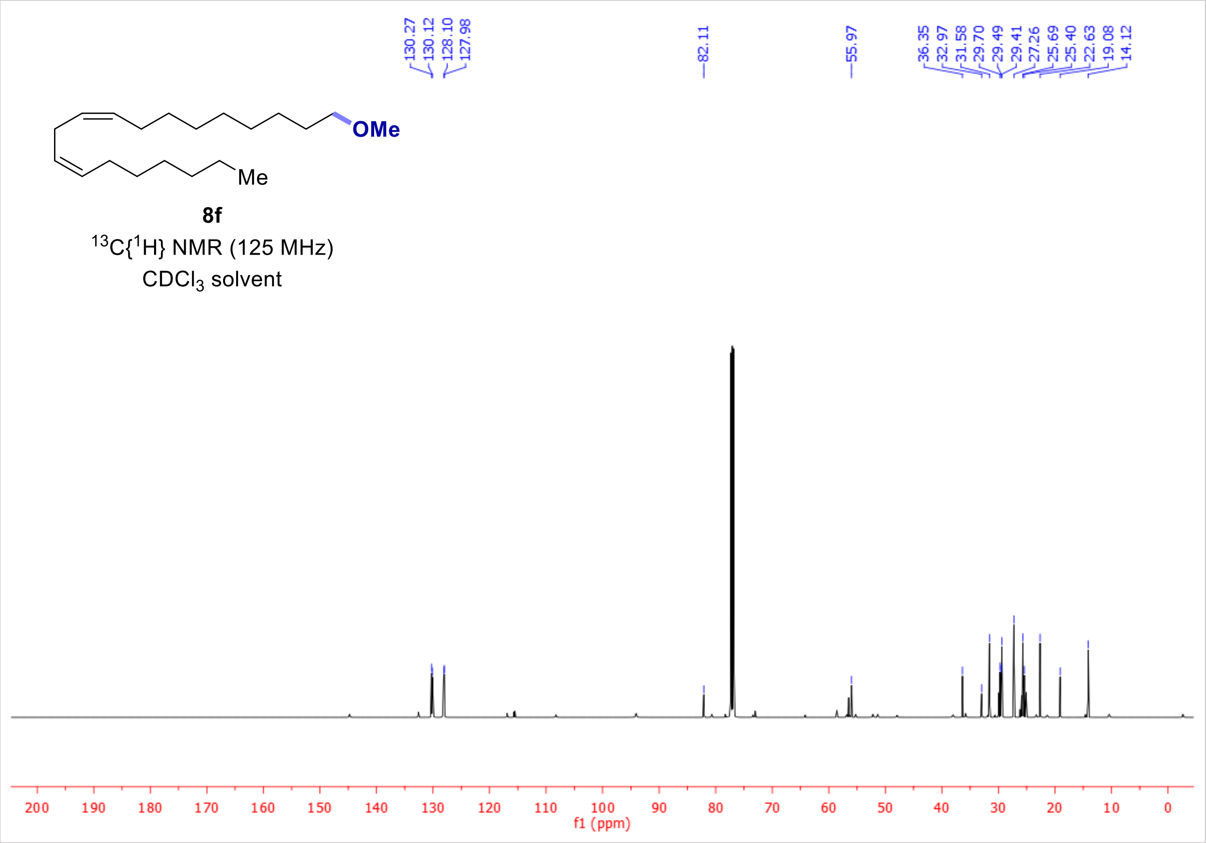
OMe
Me
8f
13C{1H} NMR (125 MHz)
CDCl3 solvent
130.27
130.12
128.10
127.98
82.11
55.97
36.35
32.97
31.58
29.70
29.49
29.41
27.26
25.69
25.40
22.63
19.08
14.12
f1 (ppm)

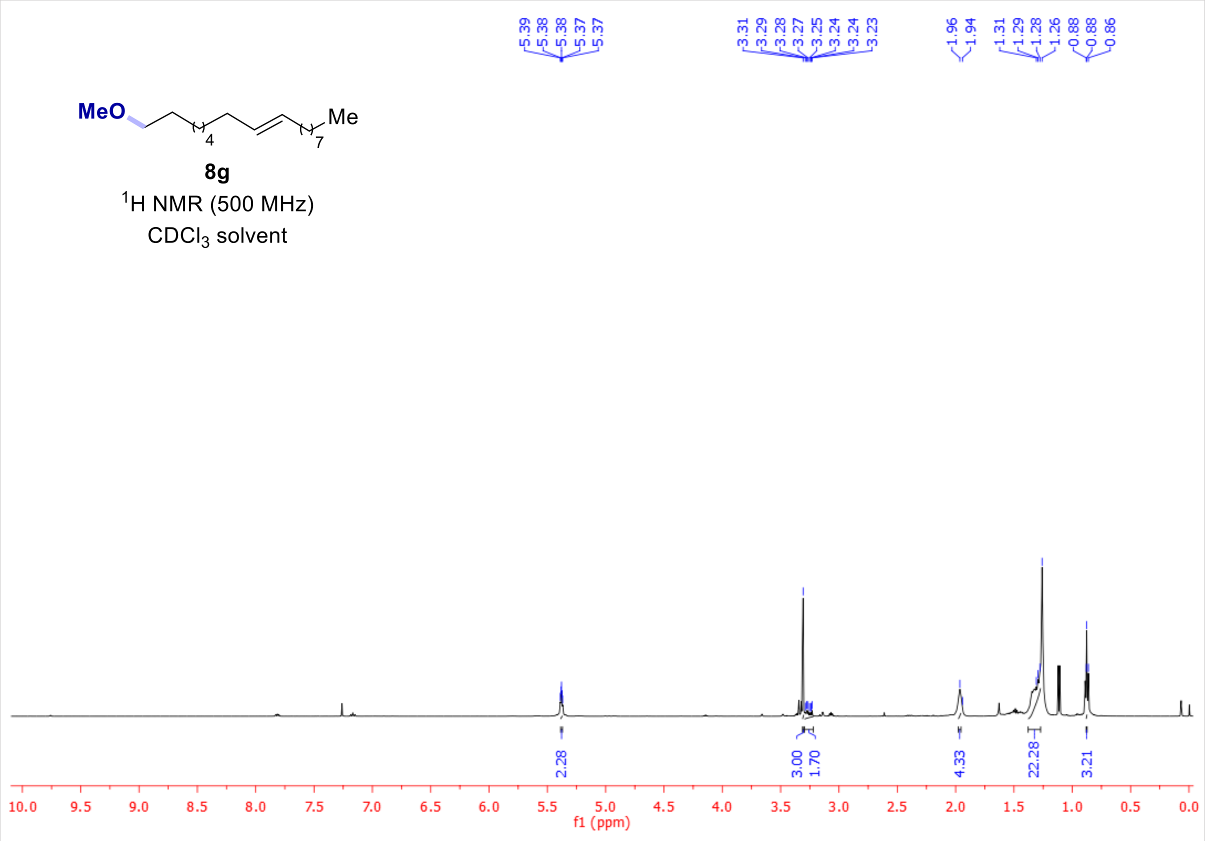
MeO
Me
4
7
8g
^{1}H NMR (500 MHz)
$CDCl_3$ solvent
5.39 5.38 5.38 5.37 5.37
3.31 3.29 3.28 3.27 3.25 3.24 3.24 3.23
1.96 1.94
1.31 1.29 1.28 1.26 0.88 0.88 0.86
2.28
3.00 1.70
4.33
22.28
3.21
10.0 9.5 9.0 8.5 8.0 7.5 7.0 6.5 6.0 5.5 5.0 4.5 4.0 3.5 3.0 2.5 2.0 1.5 1.0 0.5 0.0
f1 (ppm)

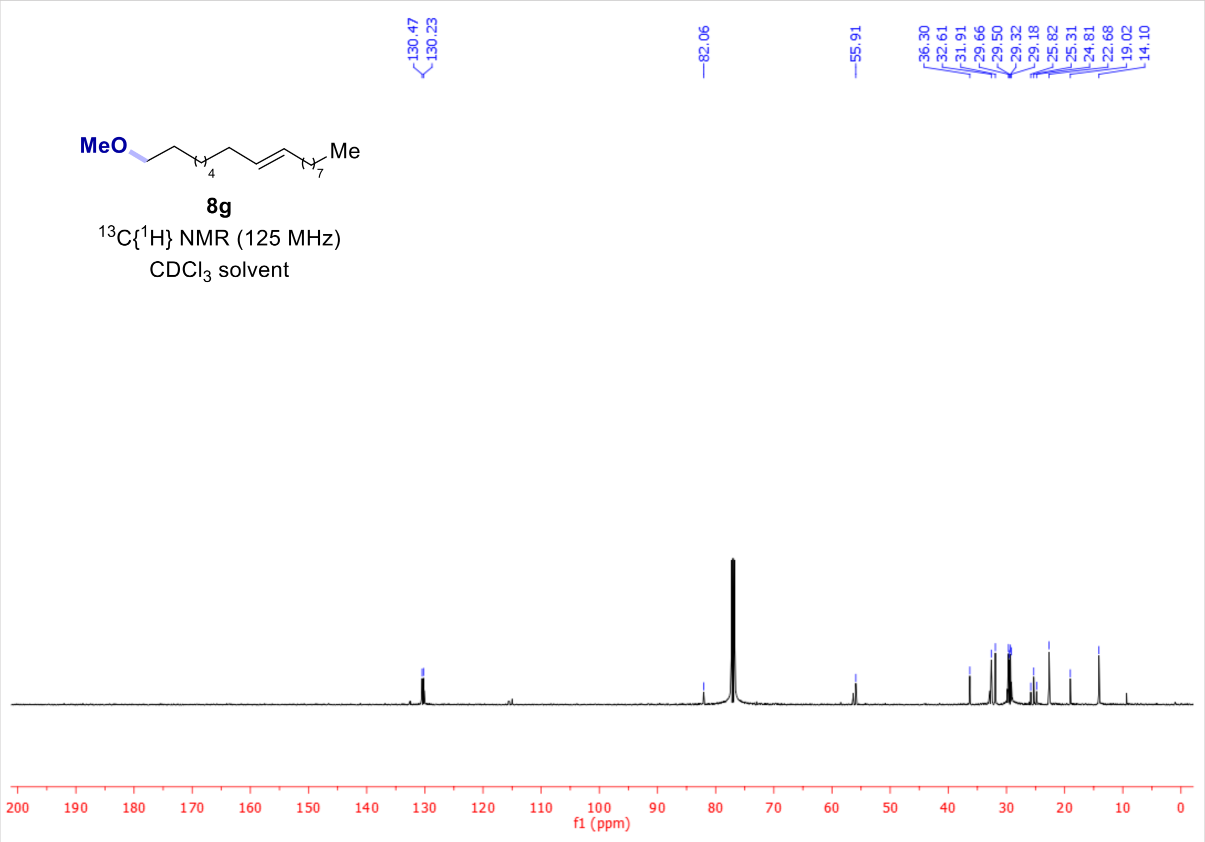
MeO
Me
4
7
8g
$^{13}C\{^1H\}$ NMR (125 MHz)
$CDCl_3$ solvent
130.47 130.23
82.06
55.91
36.30 32.61 31.91 29.66 29.50 29.32 29.18 25.82 25.31 24.81 22.68 19.02 14.10
200 190 180 170 160 150 140 130 120 110 100 90 80 70 60 50 40 30 20 10 0
f1 (ppm)

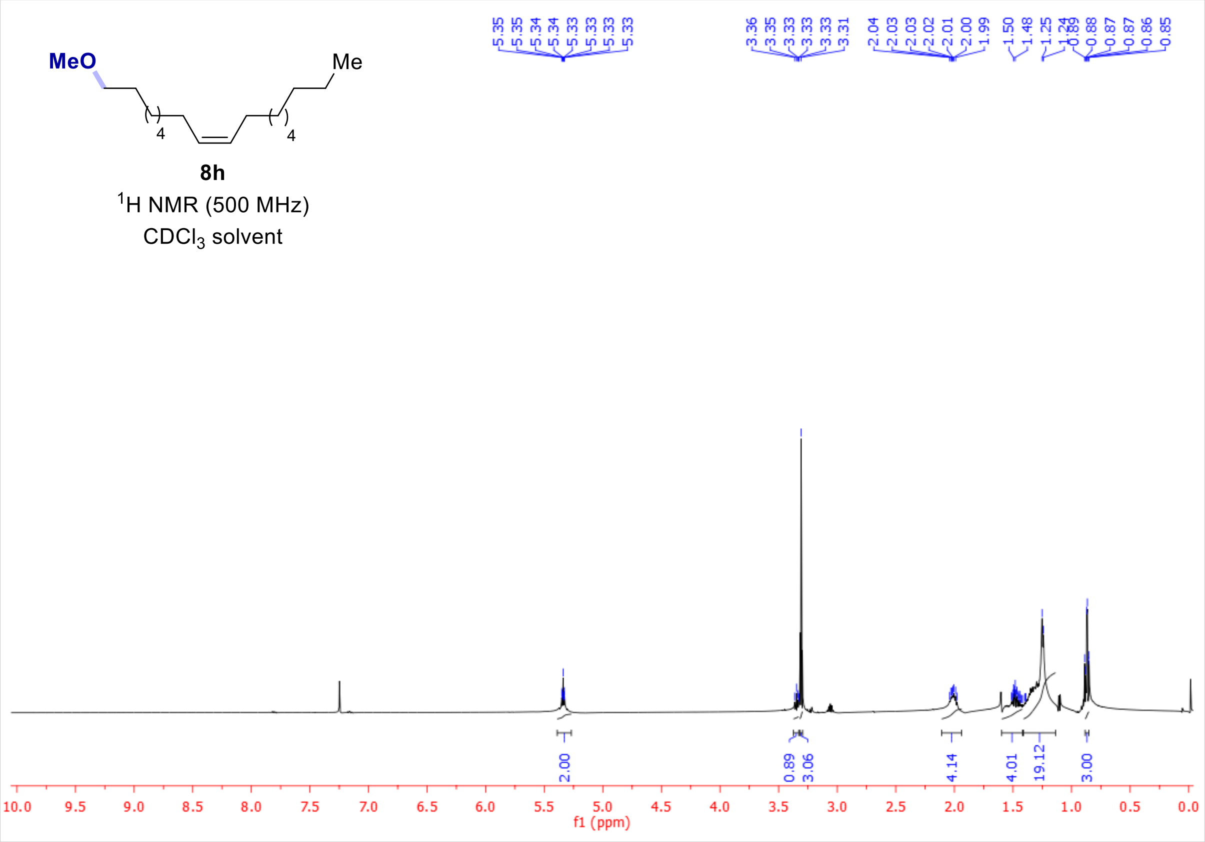
MeO
Me
4
4
8h
^{1}H NMR (500 MHz)
$CDCl_3$ solvent
f1 (ppm)

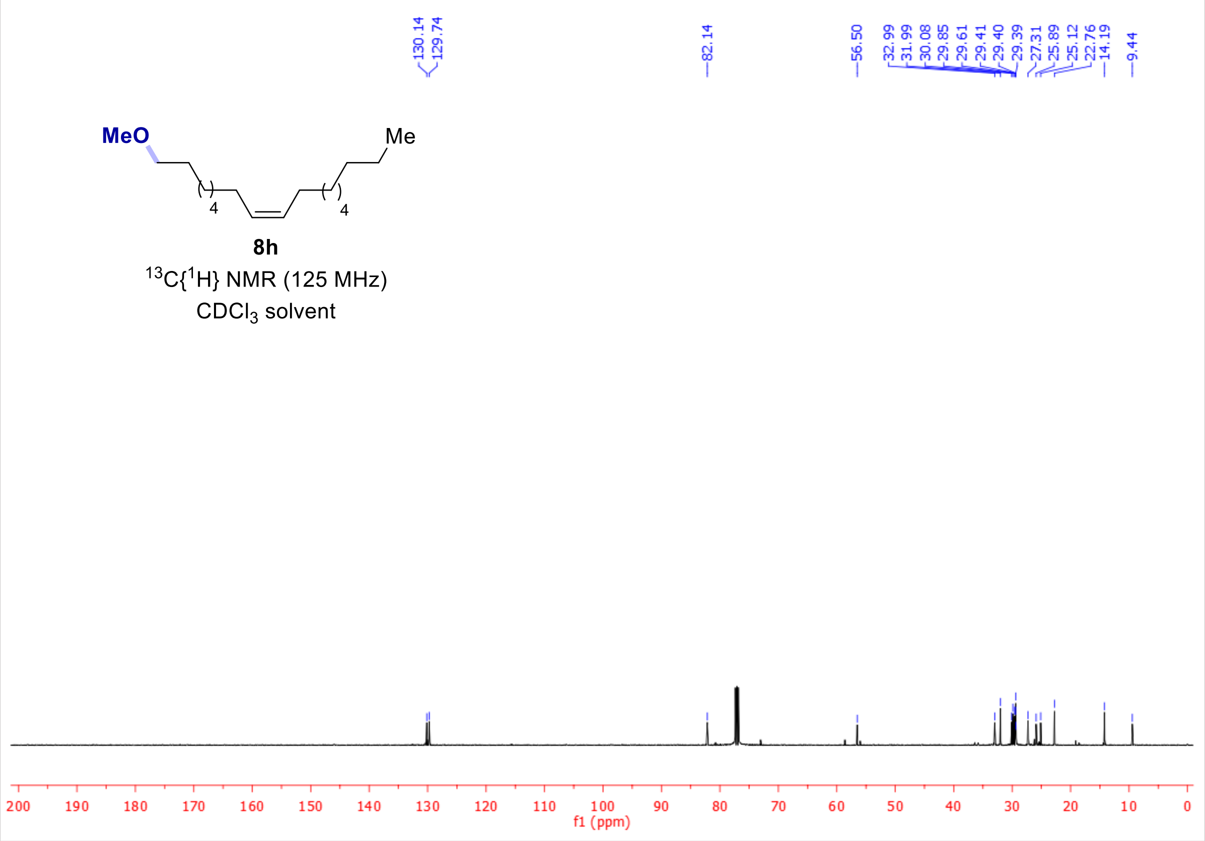
MeO
Me
4
4
8h
^{13}C{^{1}H} NMR (125 MHz)
$CDCl_3$ solvent
f1 (ppm)

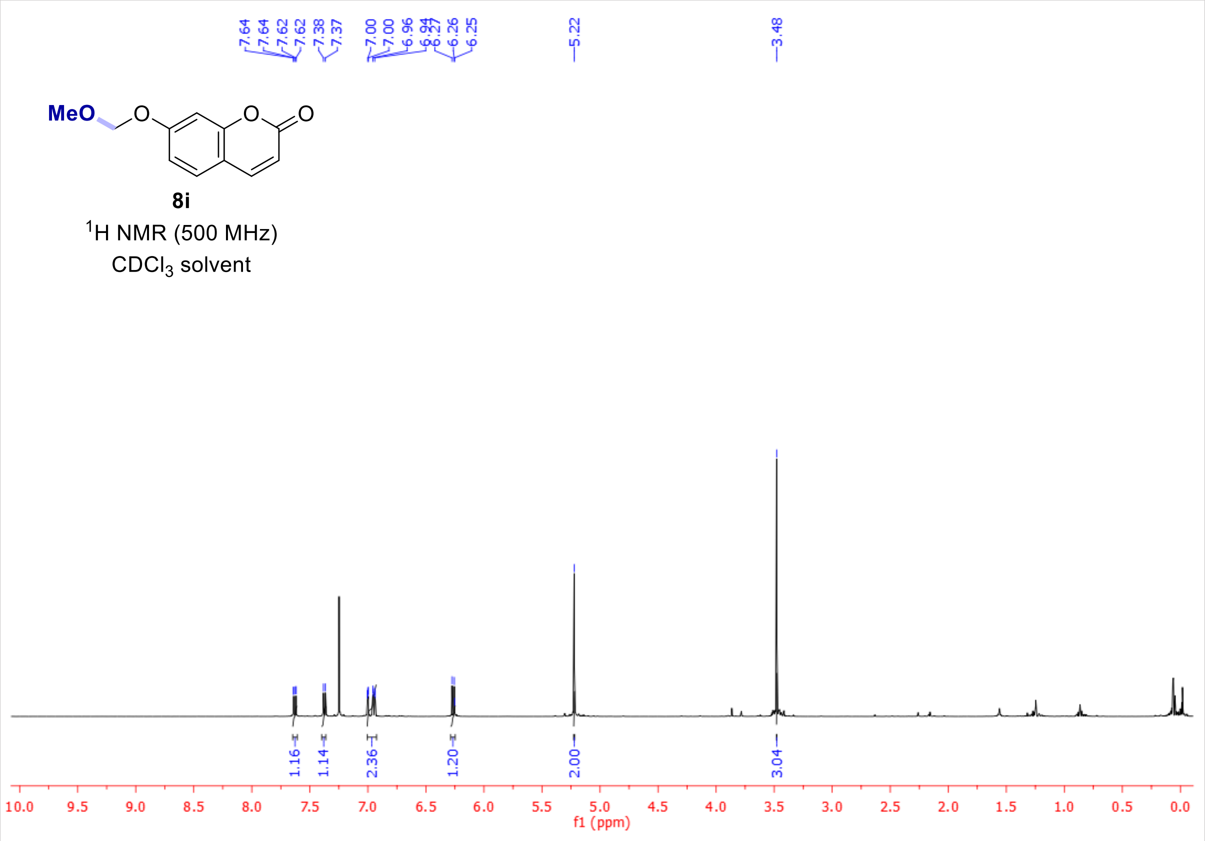
7.64
7.64
7.62
7.62
7.38
7.37
7.00
7.00
6.96
6.94
6.27
6.26
6.25
5.22
3.48
MeO
O
O
O
8i
¹H NMR (500 MHz)
CDCl₃ solvent
1.16
1.14
2.36
1.20
2.00
3.04
10.0
9.5
9.0
8.5
8.0
7.5
7.0
6.5
6.0
5.5
5.0
4.5
4.0
3.5
3.0
2.5
2.0
1.5
1.0
0.5
0.0
f1 (ppm)

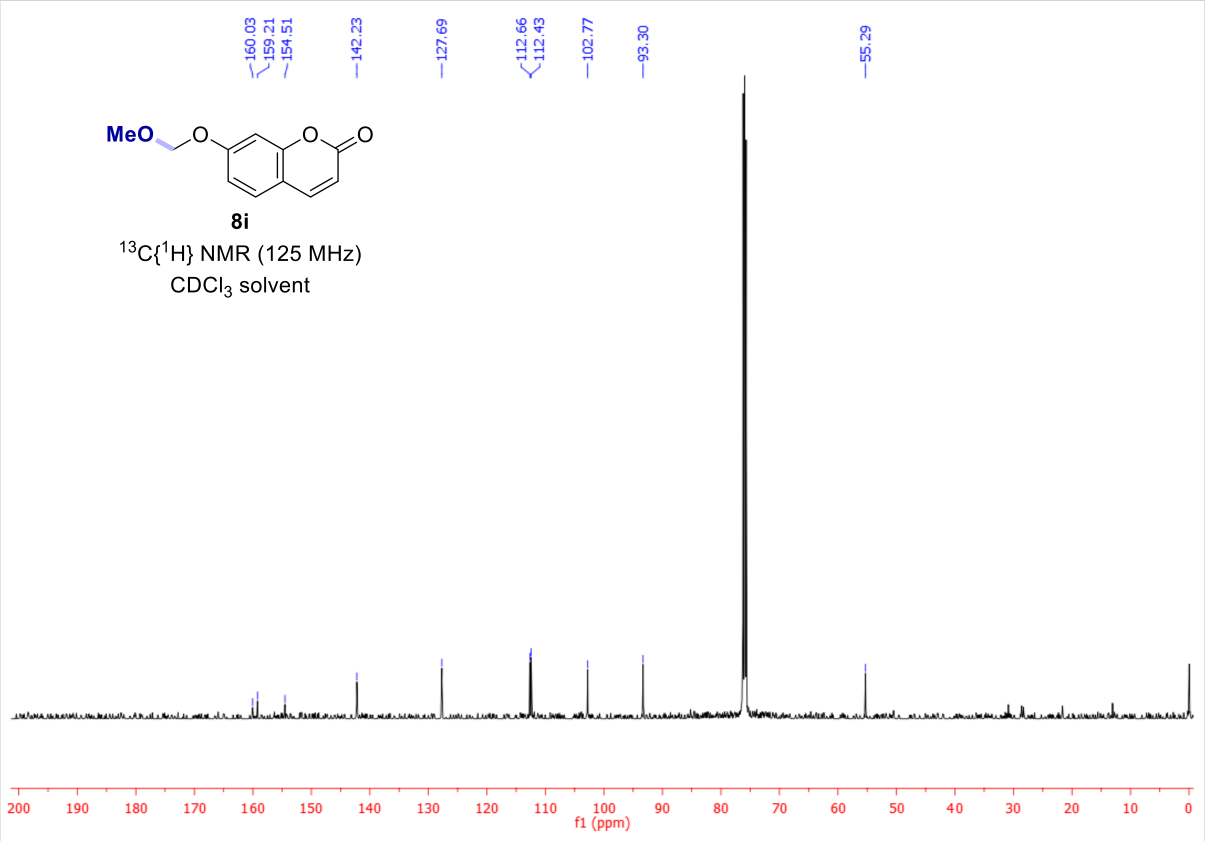
160.03
159.21
154.51
142.23
127.69
112.66
112.43
102.77
93.30
55.29
MeO
O
O
O
8i
¹³C{¹H} NMR (125 MHz)
CDCl₃ solvent
200
190
180
170
160
150
140
130
120
110
100
90
80
70
60
50
40
30
20
10
0
f1 (ppm)

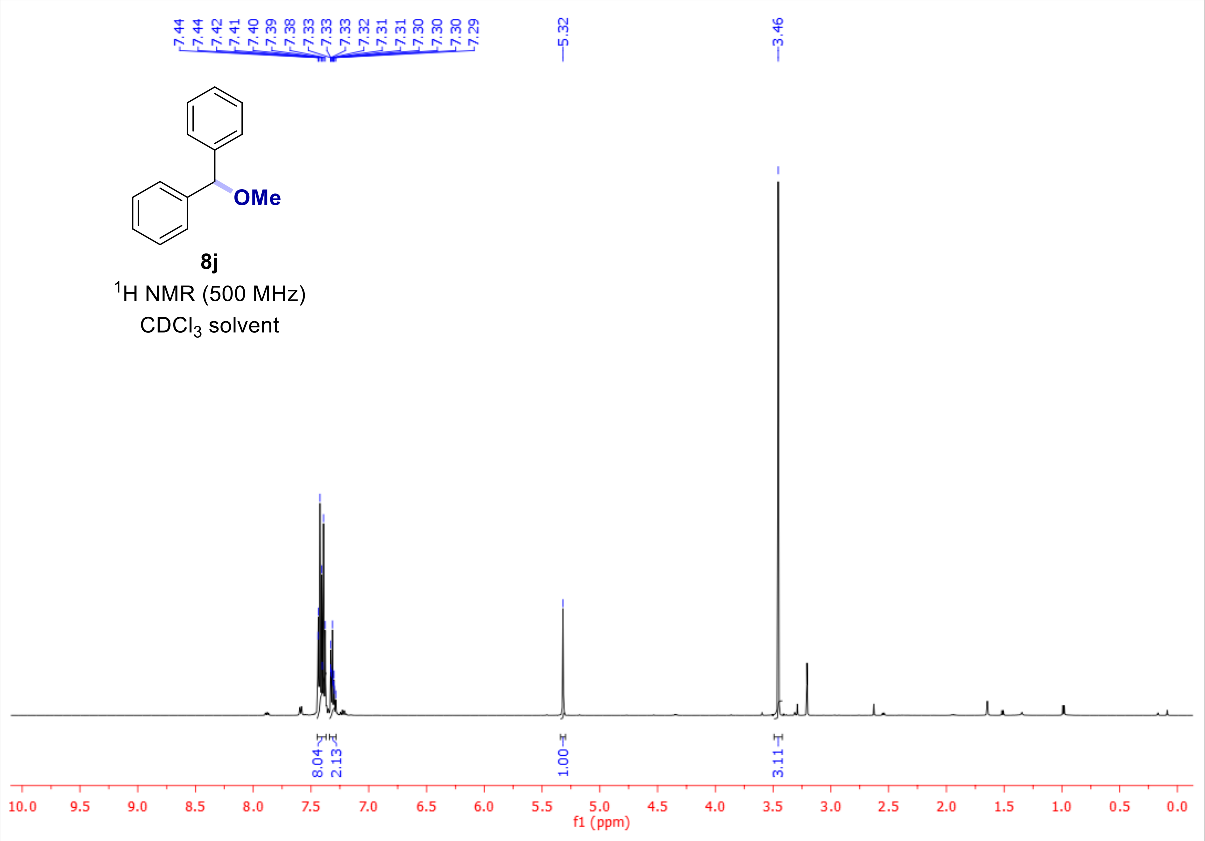
7.44
7.44
7.42
7.41
7.40
7.39
7.38
7.33
7.33
7.33
7.32
7.31
7.31
7.30
7.30
7.30
7.29
5.32
3.46
OMe
8j
1H NMR (500 MHz)
CDCl3 solvent
8.04
2.13
1.00
3.11
10.0 9.5 9.0 8.5 8.0 7.5 7.0 6.5 6.0 5.5 5.0 4.5 4.0 3.5 3.0 2.5 2.0 1.5 1.0 0.5 0.0
f1 (ppm)

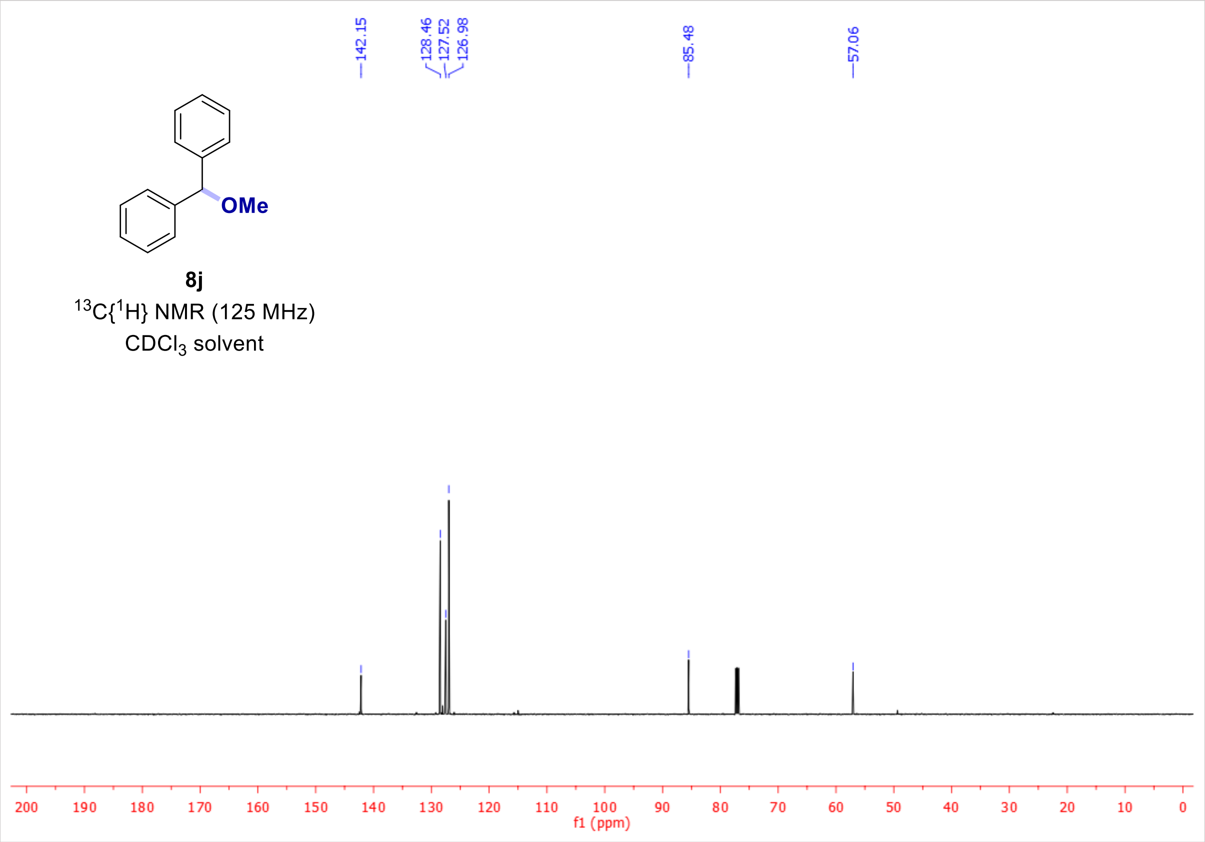
142.15
128.46
127.52
126.98
85.48
57.06
OMe
8j
13C{1H} NMR (125 MHz)
CDCl3 solvent
200 190 180 170 160 150 140 130 120 110 100 90 80 70 60 50 40 30 20 10 0
f1 (ppm)

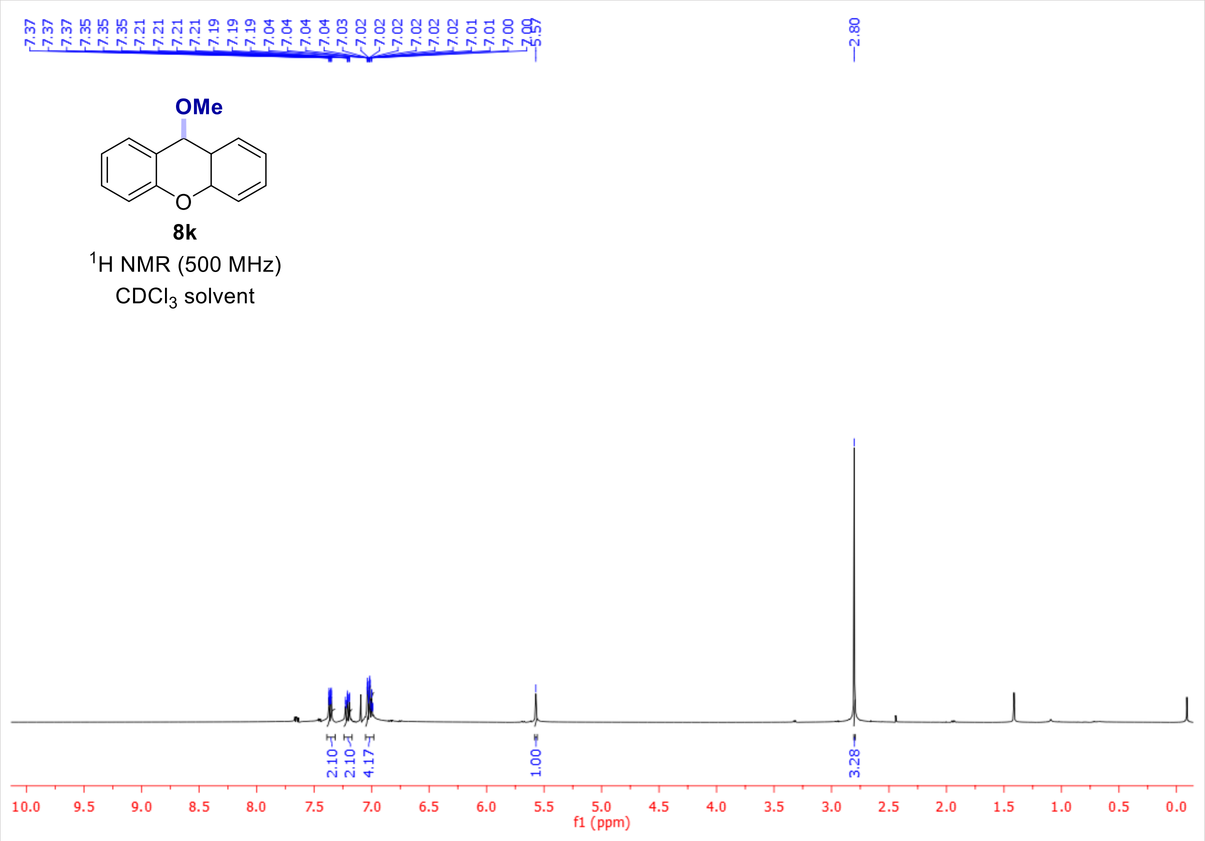
7.37
7.37
7.37
7.35
7.35
7.35
7.21
7.21
7.21
7.21
7.19
7.19
7.19
7.04
7.04
7.04
7.04
7.03
7.02
7.02
7.02
7.02
7.02
7.02
7.01
7.01
7.00
7.00
5.57
2.80
OMe
O
8k
1H NMR (500 MHz)
CDCl3 solvent
2.10
2.10
4.17
1.00
3.28
10.0
9.5
9.0
8.5
8.0
7.5
7.0
6.5
6.0
5.5
5.0
4.5
4.0
3.5
3.0
2.5
2.0
1.5
1.0
0.5
0.0
f1 (ppm)

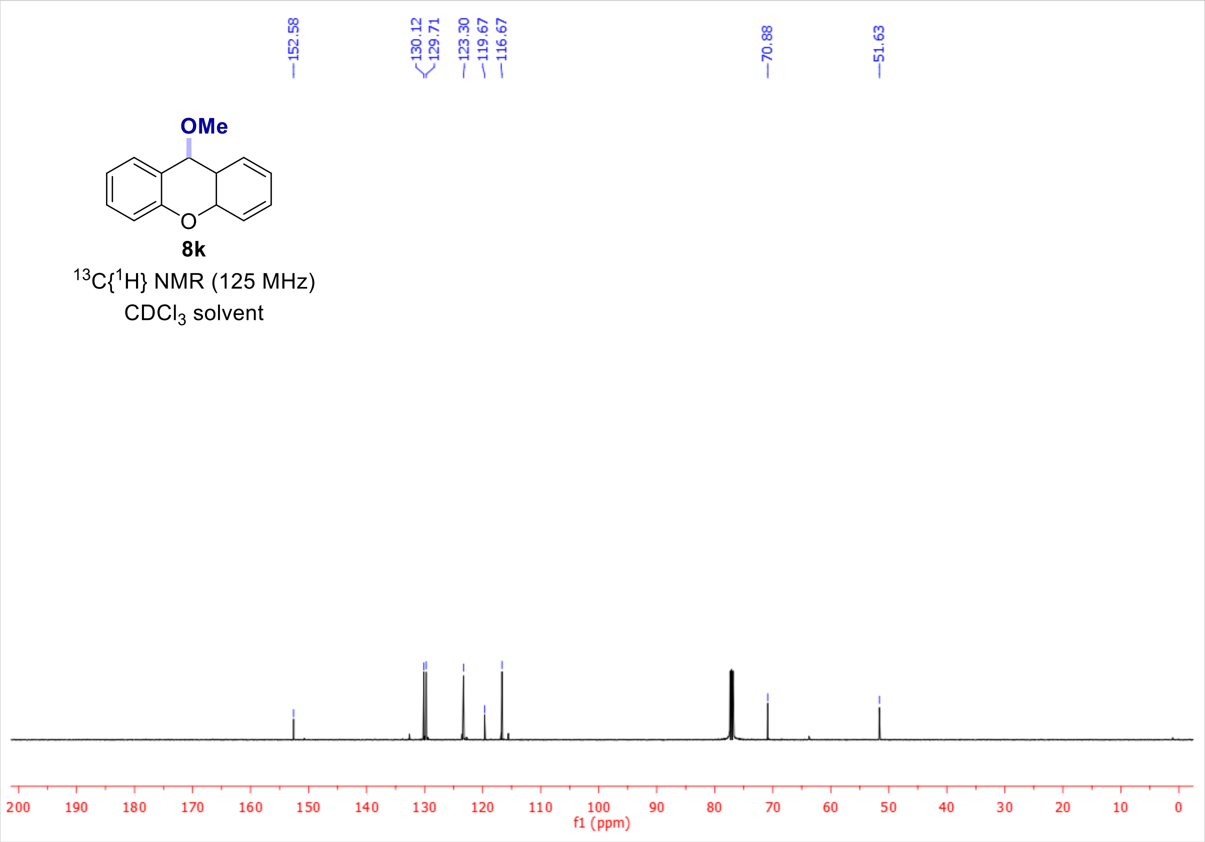
152.58
130.12
129.71
123.30
119.67
116.67
70.88
51.63
OMe
O
8k
13C{1H} NMR (125 MHz)
CDCl3 solvent
200
190
180
170
160
150
140
130
120
110
100
90
80
70
60
50
40
30
20
10
0
f1 (ppm)

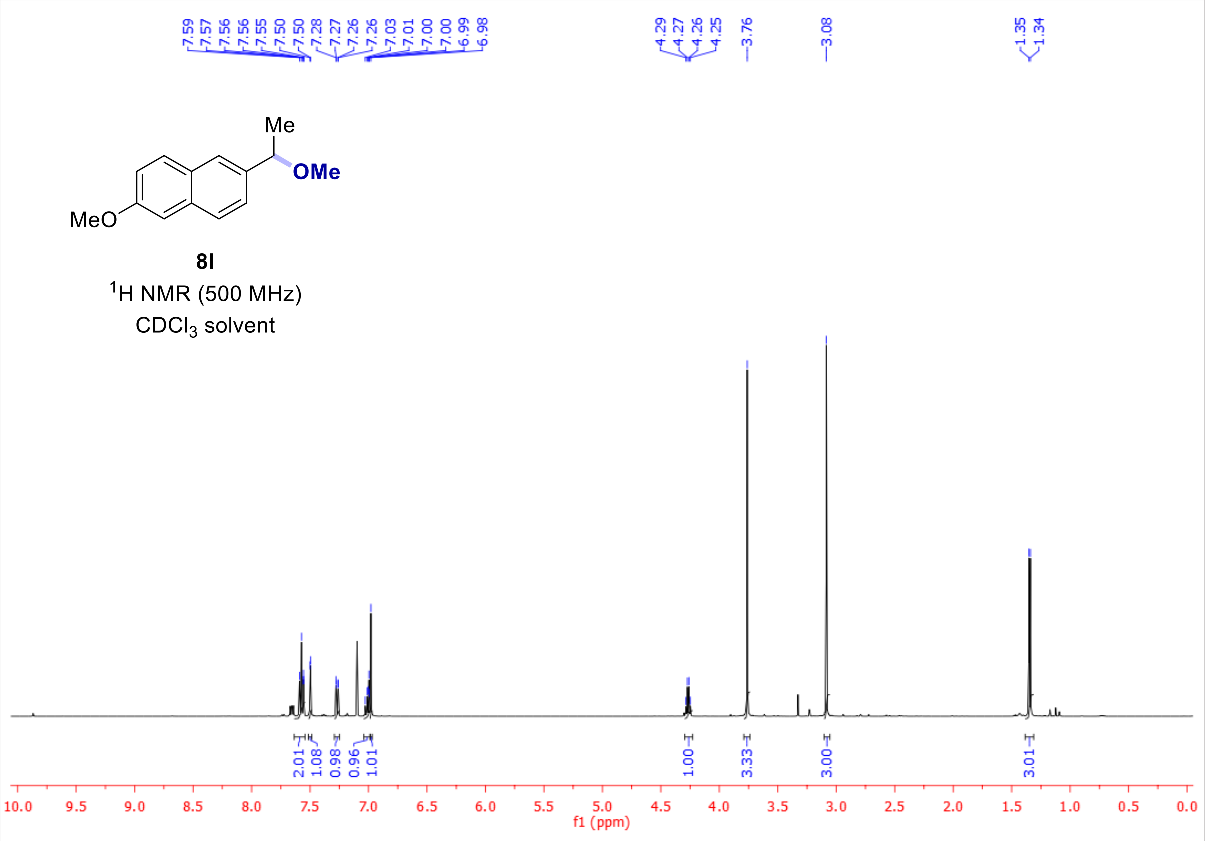
7.59 7.57 7.56 7.56 7.55 7.50 7.50 7.28 7.27 7.26 7.26 7.03 7.01 7.00 7.00 6.99 6.98
4.29 4.27 4.26 4.25
3.76
3.08
1.35 1.34
Me
OMe
MeO
8l
1H NMR (500 MHz)
CDCl3 solvent
2.01 1.08 0.98 0.96 1.01
1.00
3.33
3.00
3.01
f1 (ppm)

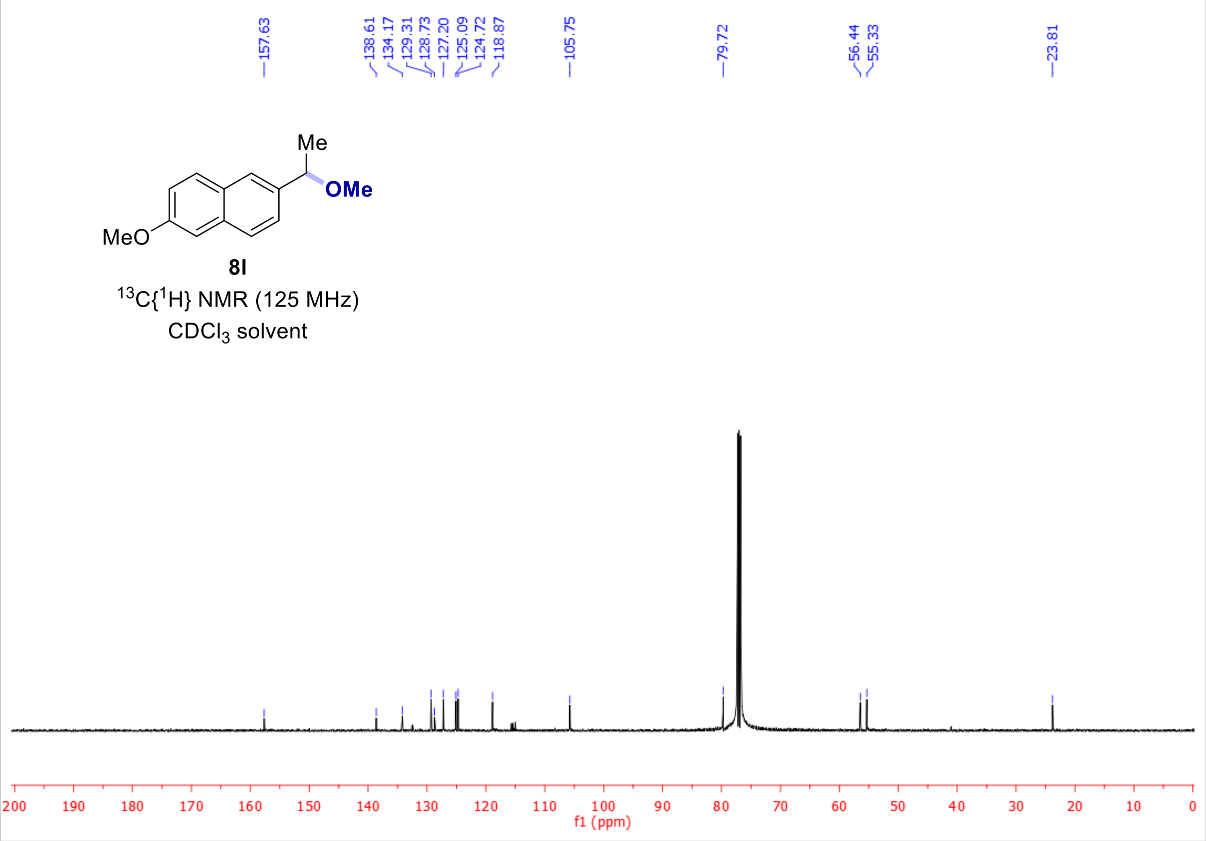
157.63
138.61 134.17 129.31 128.73 127.20 125.09 124.72 118.87
105.75
79.72
56.44 55.33
23.81
Me
OMe
MeO
8l
13C{1H} NMR (125 MHz)
CDCl3 solvent
f1 (ppm)

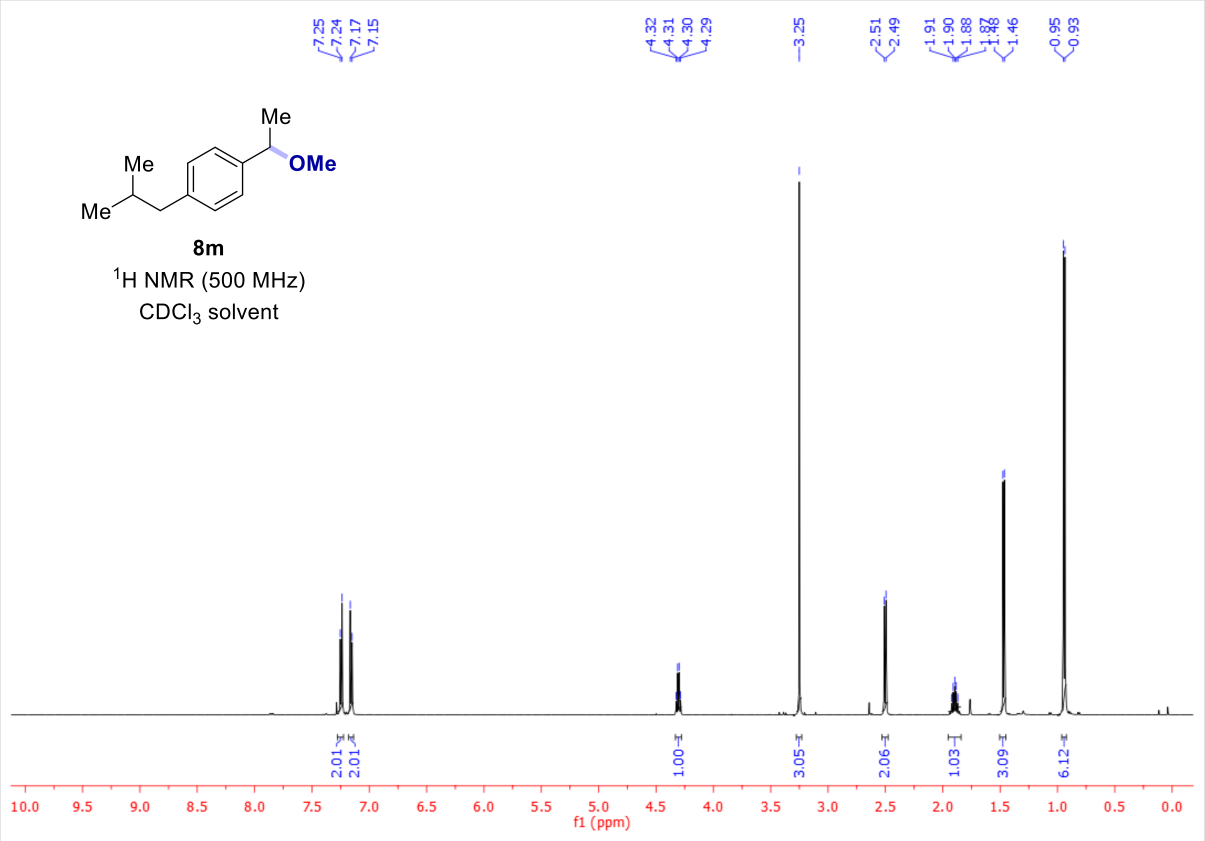

Me
OMe
Me
Me
8m
1H NMR (500 MHz)
CDCl3 solvent
7.25
7.24
7.17
7.15
4.32
4.31
4.30
4.29
3.25
2.51
2.49
1.91
1.90
1.88
1.87
1.48
1.46
0.95
0.93
2.01
2.01
1.00
3.05
2.06
1.03
3.09
6.12
f1 (ppm)

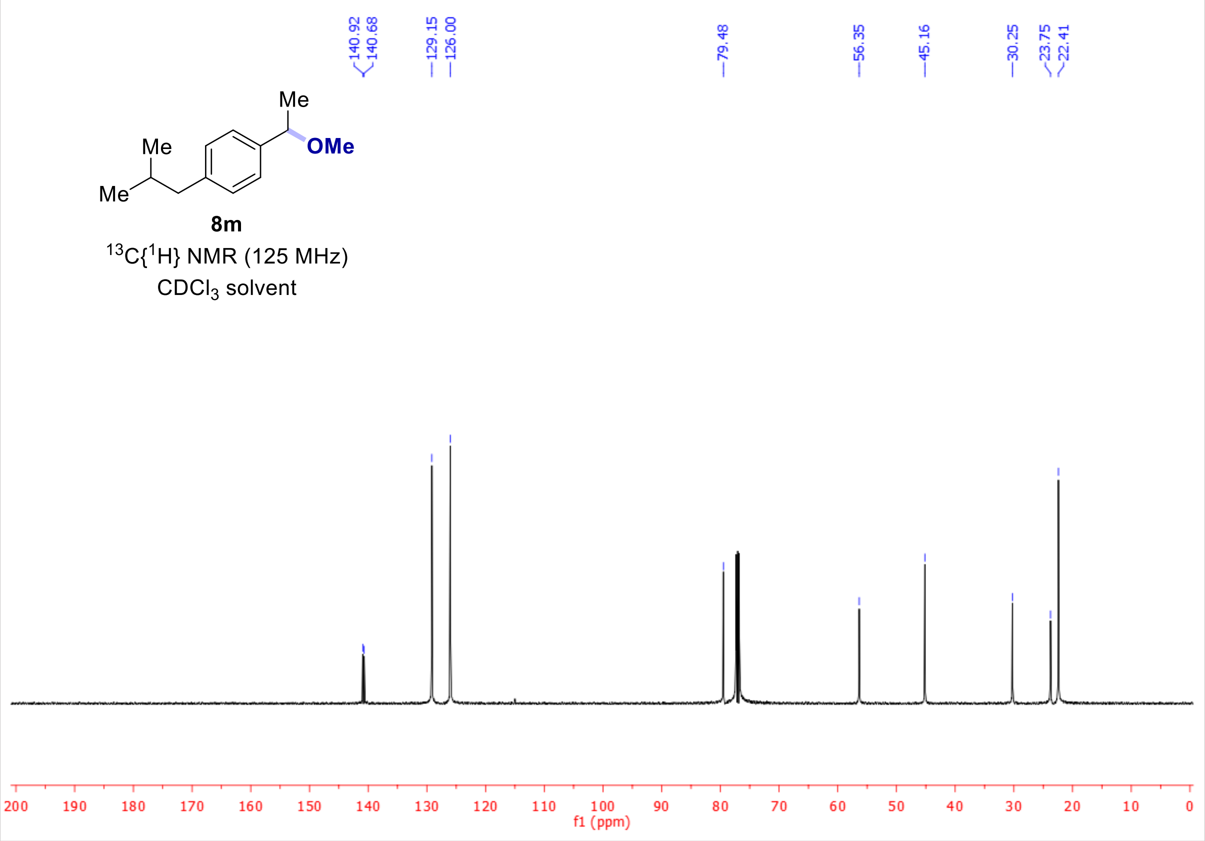

Me
OMe
Me
Me
8m
13C{1H} NMR (125 MHz)
CDCl3 solvent
140.92
140.68
129.15
126.00
79.48
56.35
45.16
30.25
23.75
22.41
f1 (ppm)

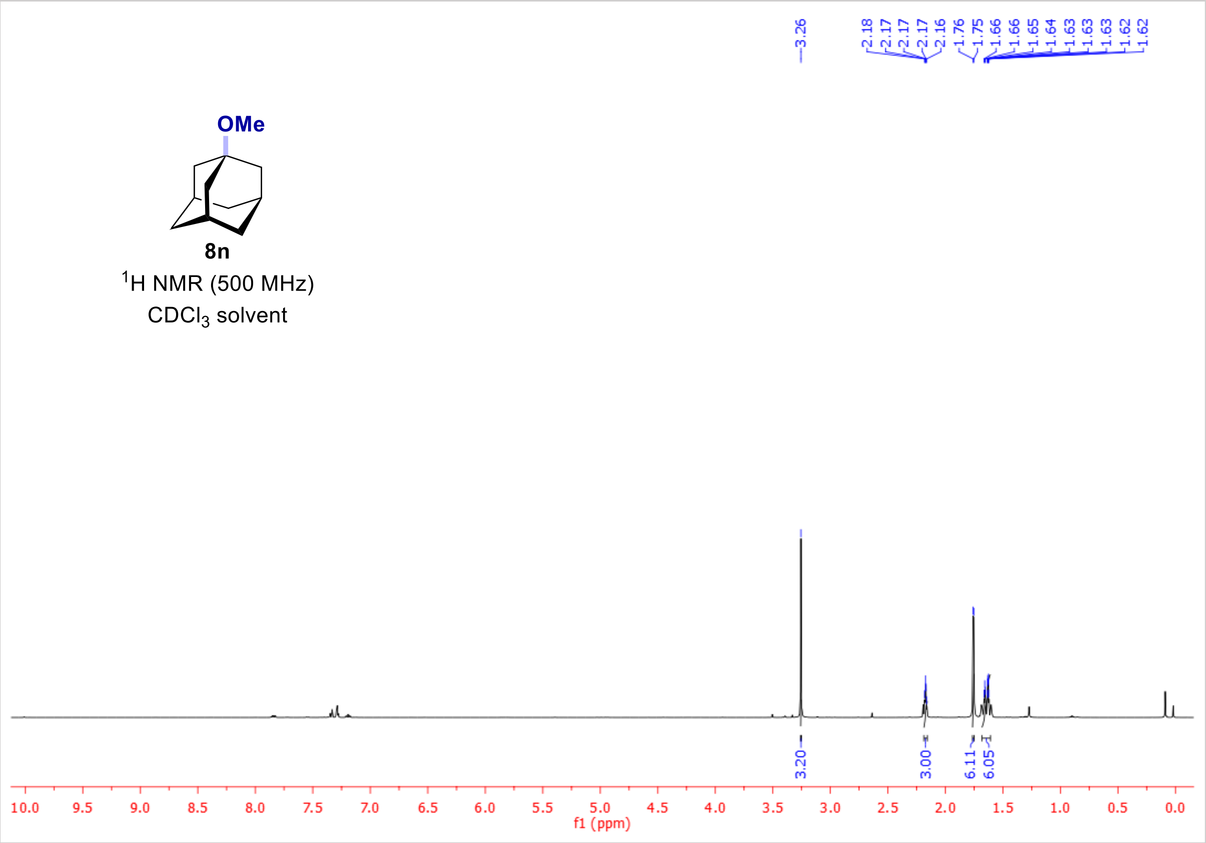
OMe
8n
^{1}H NMR (500 MHz)
$CDCl_3$ solvent
3.26
2.18
2.17
2.17
2.17
2.16
1.76
1.75
1.66
1.66
1.65
1.64
1.63
1.63
1.63
1.62
1.62
3.20
3.00
6.11
6.05
10.0 9.5 9.0 8.5 8.0 7.5 7.0 6.5 6.0 5.5 5.0 4.5 4.0 3.5 3.0 2.5 2.0 1.5 1.0 0.5 0.0
f1 (ppm)

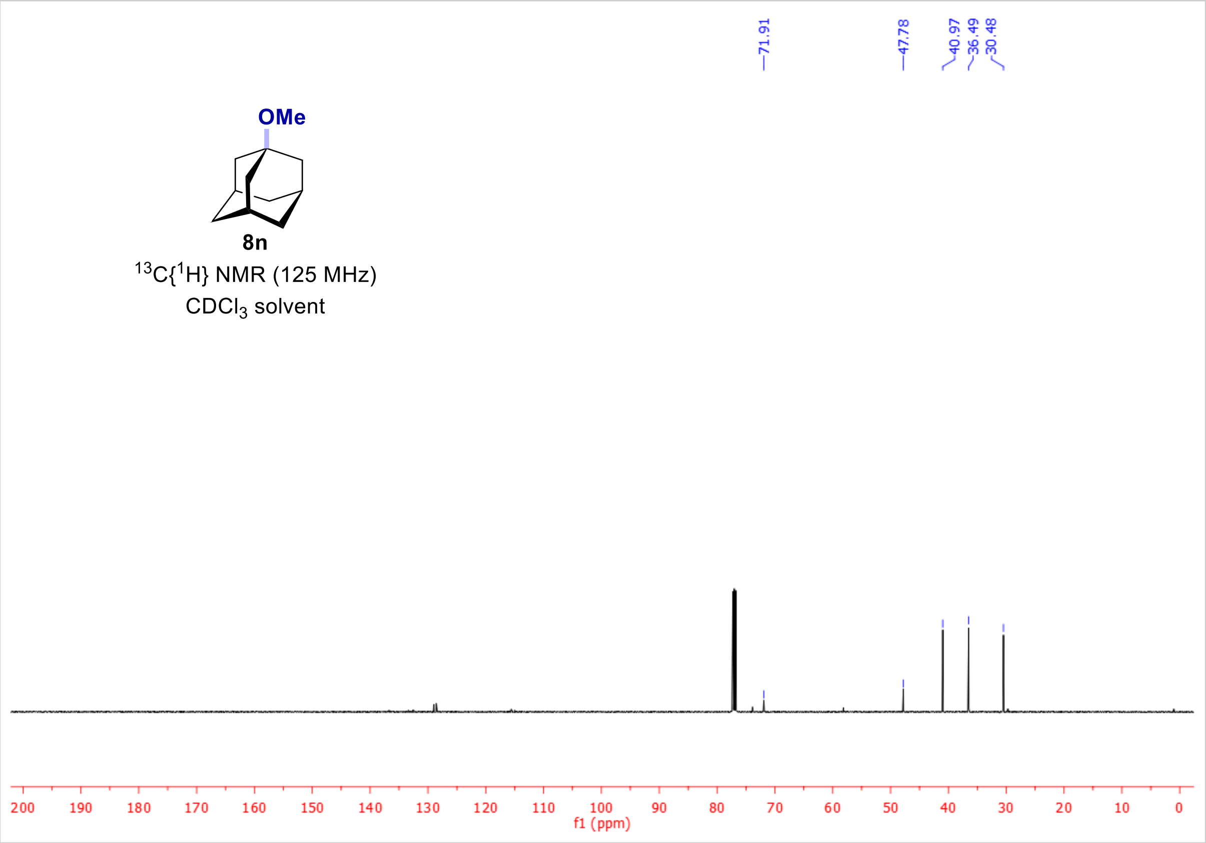
OMe
8n
^{13}C{^{1}H} NMR (125 MHz)
$CDCl_3$ solvent
71.91
47.78
40.97
36.49
30.48
200 190 180 170 160 150 140 130 120 110 100 90 80 70 60 50 40 30 20 10 0
f1 (ppm)

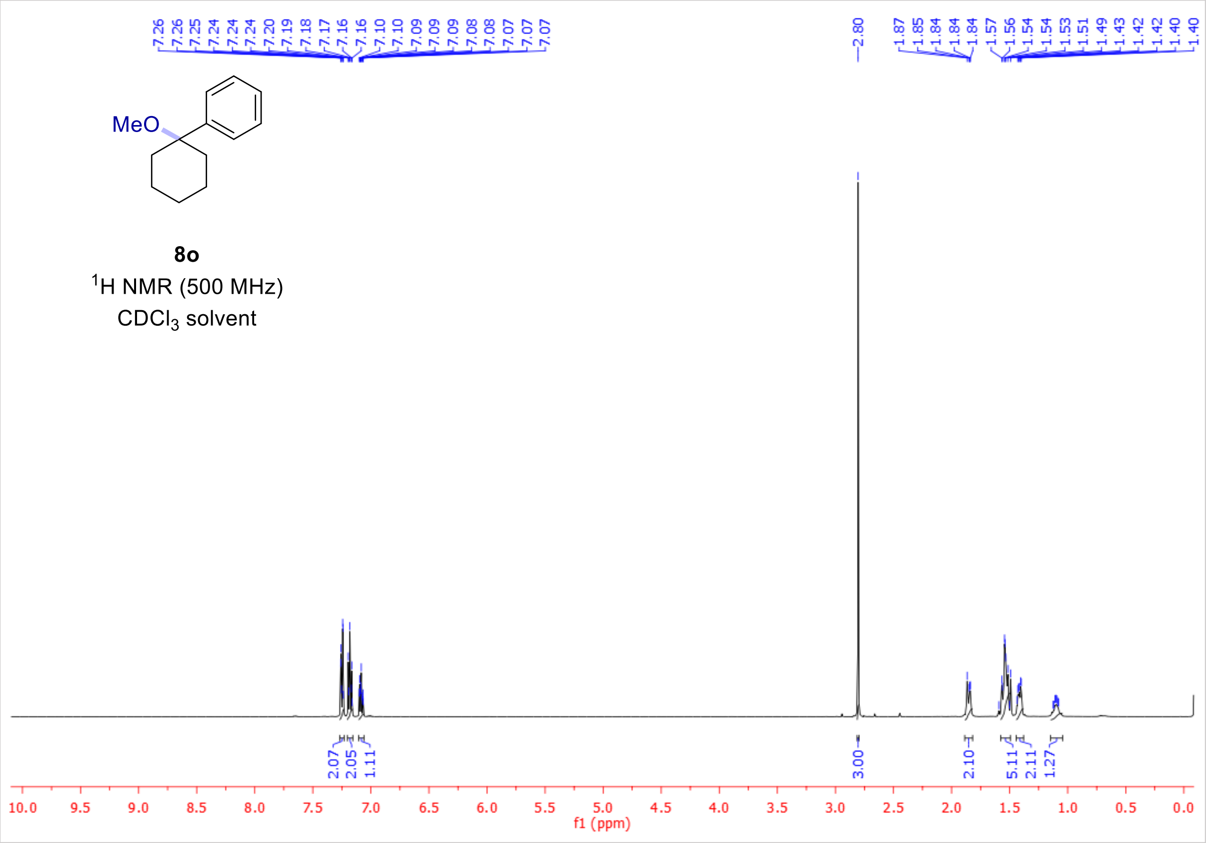
7.26
7.26
7.25
7.24
7.24
7.24
7.20
7.19
7.18
7.17
7.16
7.16
7.10
7.10
7.09
7.09
7.09
7.08
7.08
7.07
7.07
7.07
2.80
1.87
1.85
1.84
1.84
1.84
1.57
1.56
1.54
1.54
1.53
1.51
1.49
1.43
1.42
1.40
1.40
MeO
8o
^{1}H NMR (500 MHz)
$CDCl_3$ solvent
2.07
2.05
1.11
3.00
2.10
5.11
2.11
1.27
10.0
9.5
9.0
8.5
8.0
7.5
7.0
6.5
6.0
5.5
5.0
4.5
4.0
3.5
3.0
2.5
2.0
1.5
1.0
0.5
0.0
f1 (ppm)

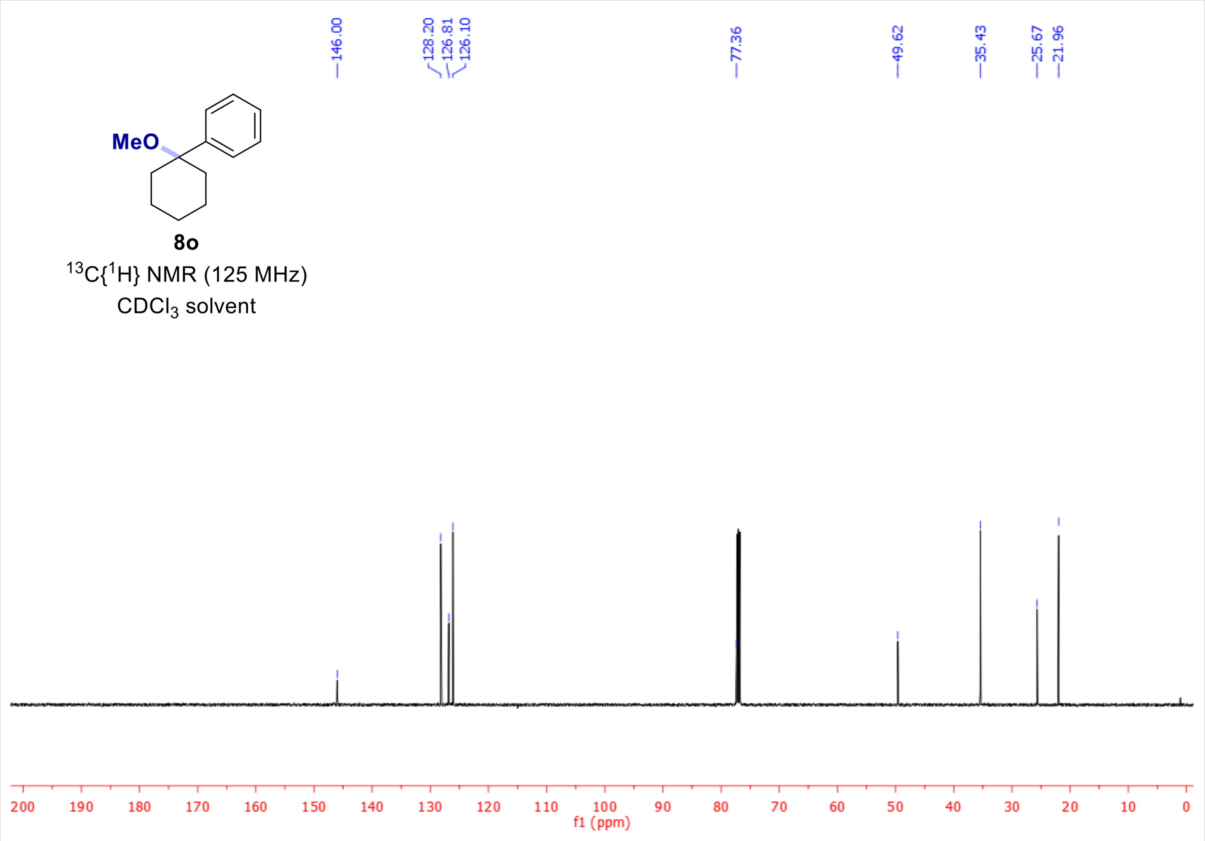
146.00
128.20
126.81
126.10
77.36
49.62
35.43
25.67
21.96
MeO
8o
$^{13}C\{^1H\}$ NMR (125 MHz)
$CDCl_3$ solvent
200 190 180 170 160 150 140 130 120 110 100 90 80 70 60 50 40 30 20 10 0
f1 (ppm)

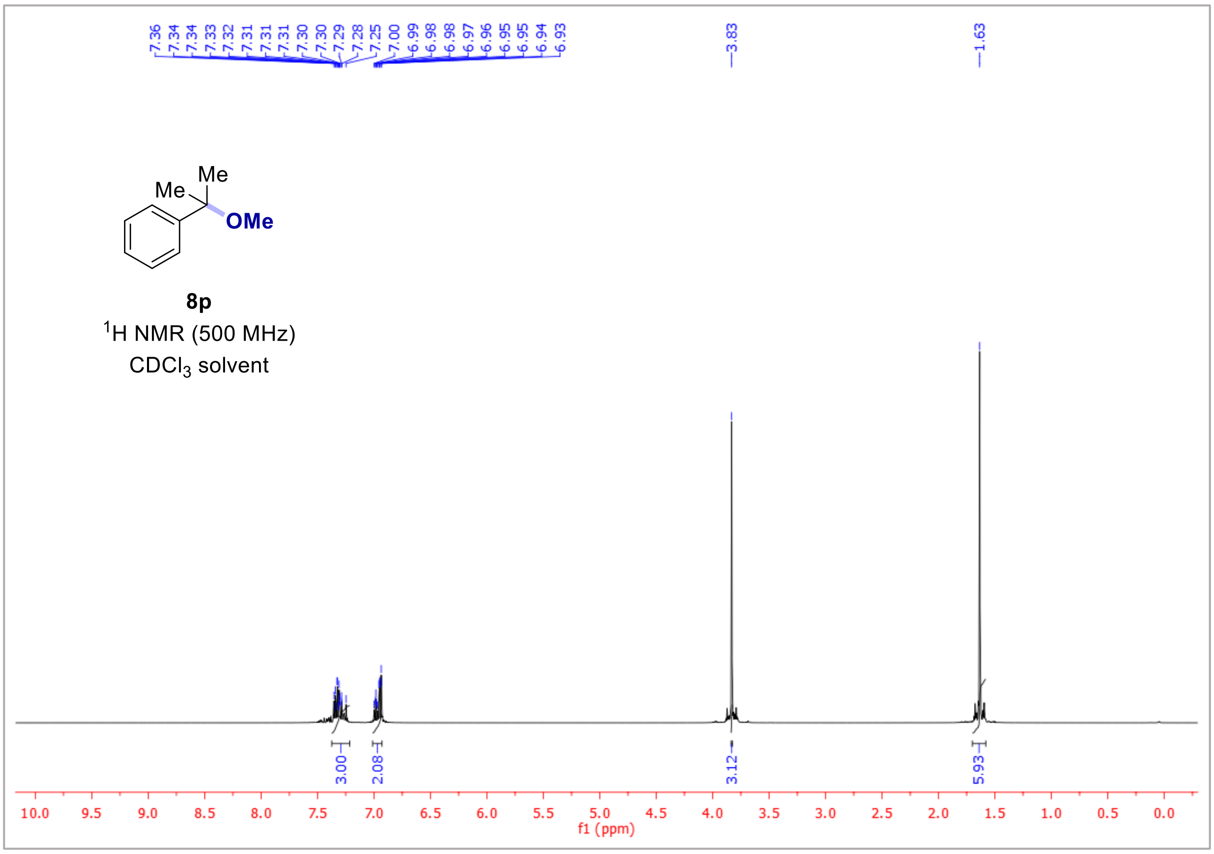
7.36 7.34 7.34 7.33 7.32 7.31 7.31 7.31 7.30 7.30 7.29 7.28 7.25 7.00 6.99 6.98 6.98 6.97 6.96 6.95 6.95 6.94 6.93
3.83
1.63
Me
Me
OMe
8p
^1H NMR (500 MHz)
$CDCl_3$ solvent
3.00
2.08
3.12
5.93
10.0 9.5 9.0 8.5 8.0 7.5 7.0 6.5 6.0 5.5 5.0 4.5 4.0 3.5 3.0 2.5 2.0 1.5 1.0 0.5 0.0
f1 (ppm)

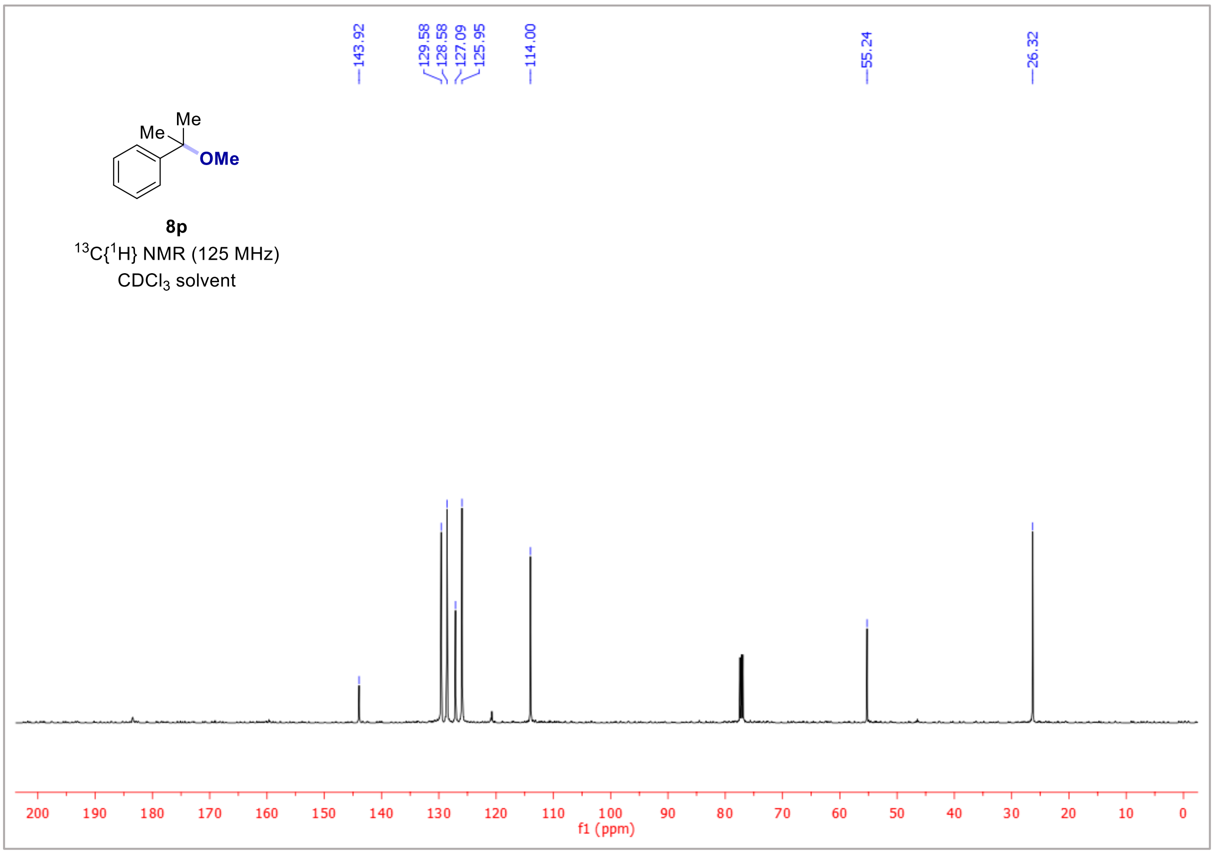
143.92
129.58
128.58
127.09
125.95
114.00
55.24
26.32
Me
Me
OMe
8p
13C{1H} NMR (125 MHz)
CDCl3 solvent
200 190 180 170 160 150 140 130 120 110 100 90 80 70 60 50 40 30 20 10 0
f1 (ppm)

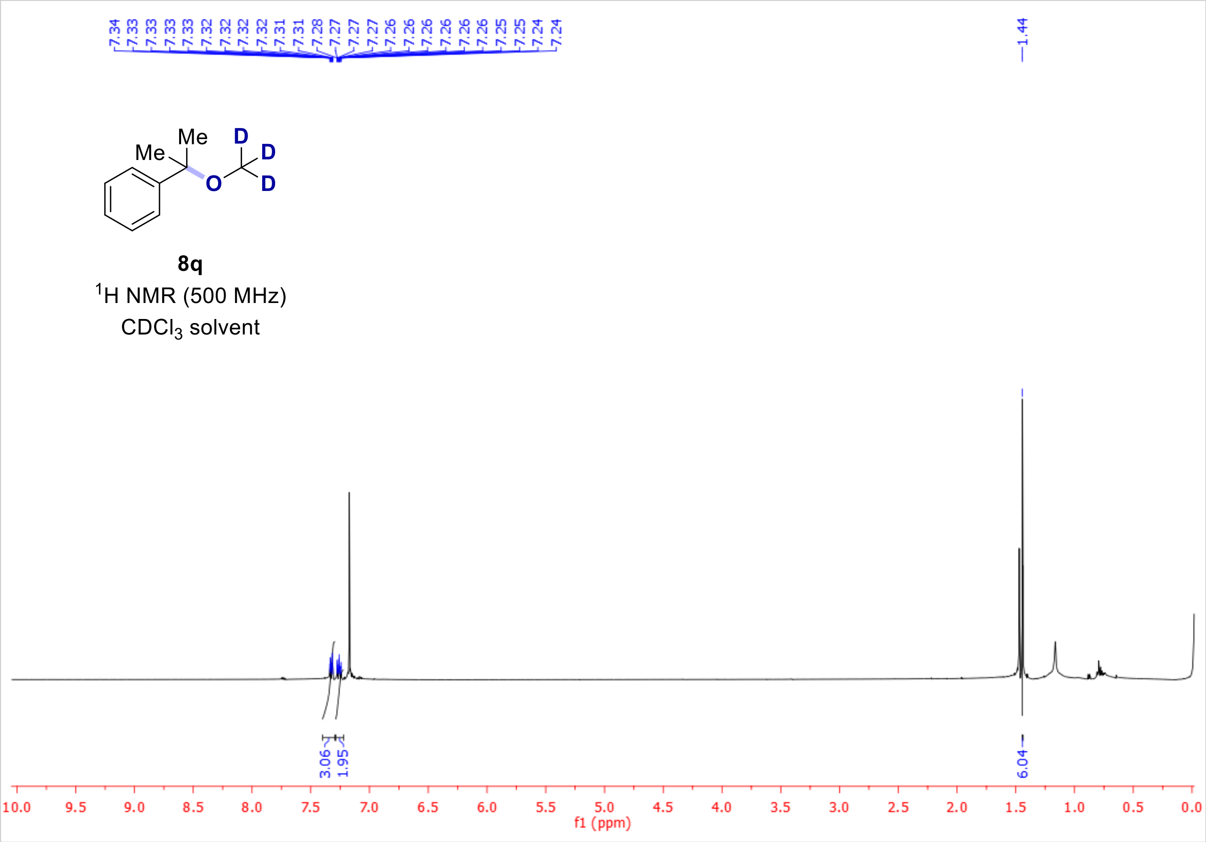
7.34 7.33 7.33 7.33 7.33 7.32 7.32 7.32 7.32 7.31 7.31 7.28 7.27 7.27 7.27 7.26 7.26 7.26 7.26 7.26 7.26 7.25 7.25 7.24 7.24
1.44
Me
Me
O
D
D
D
8q
1H NMR (500 MHz)
CDCl3 solvent
3.06
1.95
6.04
10.0 9.5 9.0 8.5 8.0 7.5 7.0 6.5 6.0 5.5 5.0 4.5 4.0 3.5 3.0 2.5 2.0 1.5 1.0 0.5 0.0
f1 (ppm)
